# A Common Small-Molecule Inhibitor Targeting Multiple Siglecs Associated with Immune Checkpoint Suppression of Cytotoxicity and Phagocytosis

**DOI:** 10.1021/jacs.6c15475

**Published:** 2026-09-29

**Authors:** Cinya Chung, Ruofan Li, Li-Chun Cheng, Chen-Yo Fan, Kuo-Shiang Liao, Chih-Chuan Kung, Chi-Huey Wong

**Affiliations:** † Genomics Research Center, Academia Sinica, 128 Academia Rd., Sec, 2, Taipei 11529, Taiwan; ‡ Department of Chemistry, 4356 The Scripps Research Institute, 10550 North Torrey Pines Rd., La Jolla, California 92037, United States

## Abstract

Immune checkpoints are key regulators of the immune system that cancer cells exploit to evade immune surveillance. While these pathways have been targeted with antibody therapies such as PD-1/PD-L1 inhibitors, other inhibitory receptors, like sialic acid-binding immunoglobulin-type lectins (Siglecs), remain clinically underexplored. Among the many Siglecs on immune cells that interact with sialosides on cancer cells, Siglec-9 and Siglec-10 are prominently expressed on macrophages within the tumor microenvironment, functioning as key inhibitory immune checkpoints that suppress macrophage-mediated phagocytosis and cytotoxicity. Similarly, Siglec-7 and Siglec-9 are expressed on NK cells, acting as primary immune checkpoints that inhibit cellular cytotoxicity. Since all Siglecs recognize sialic acids, we sought to identify a common small-molecule inhibitor to target these receptors for immunotherapy. We first chemoenzymatically synthesized more than 60 glycans, including multiantennary glycans with terminal sialic acids, and assembled them onto a glycan microarray to screen for high-affinity binders to these Siglecs. We identified several strong binders in these array assays; among them, a truncated glycolyl sialoside (R3) was found to strongly inhibit the interaction of Siglec-10 with HER2-low MCF-7 cells, substantially enhancing macrophage-mediated phagocytosis and cytotoxicity. Notably, R3 also interacts significantly with other inhibitory immune checkpoint receptors, including Siglec-5, -7, -9, -11, and -15, thereby relieving Siglec-mediated suppression and enhancing NK cell-mediated cytotoxicity in cell-based assays. Structural studies are underway to investigate the binding mode of R3 with these Siglecs to guide further optimization toward a common small-molecule inhibitor targeting multiple inhibitory glyco-immune checkpoints for cancer immunotherapy.

## Graphical abstract

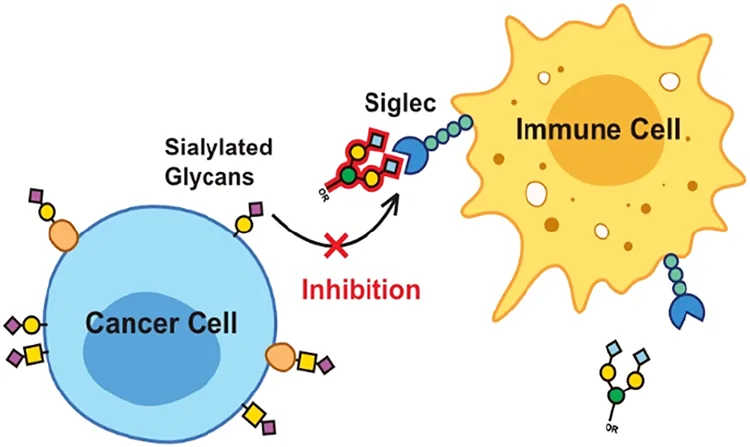

## Introduction

Immunotherapy has become an important way to fight cancer by enhancing the body’s natural immune response. It works by targeting regulatory pathways in T cells to enhance the immune system’s ability to recognize and destroy cancer cells. When immune cells are activated to destroy targets, certain cell-surface receptors may interact with ligands on the target cells to inhibit this cytotoxic activity, a process called immune checkpoint inhibition.^1,2^ Normally, immune checkpoint proteins like PD-1 (programmed cell death protein 1)^3^ and CTLA-4 (cytotoxic T-lymphocyte-associated protein 4)^4^ help prevent the immune system from attacking normal cells. However, cancer cells can exploit these checkpoints to evade immune surveillance. Immune checkpoint inhibitors block these proteins, thereby reactivating T cells against tumors. For example, nivolumab and pembrolizumab are anti-PD-1 antibodies approved for treating melanoma, lung cancer, and several other malignancies.^5^ Similarly, ipilimumab, an anti-CTLA-4 antibody, has been used in the treatment of advanced melanoma.^6^

While the PD-1 and CTLA-4 pathways have been successfully targeted with antibody therapies,^1,7^ other immune checkpoint inhibitory receptors like sialic acid-binding immunoglobulin-type lectins (Siglecs) have been extensively studied but have not been well explored clinically.^8–12^ Siglecs are a family of cell-surface receptors predominantly expressed on immune cells, where they recognize sialic acid-containing glycans on host or tumor cells. Many Siglecs transmit inhibitory signals through intracellular ITIM (immunoreceptor tyrosine-based inhibitory motifs) or ITIM-like motifs that recruit tyrosine phosphatases such as SHP-1 and SHP-2, thereby suppressing immune activation,^13^ whereas activating Siglecs contain positively charged amino acid residues within their transmembrane domains that associate with ITAM-bearing adaptor proteins such as DAP12. The mechanism of immune check point regulation is used in our immune system to maintain self-tolerance but can be exploited by tumors to engage Siglecs and evade immune responses.

Key inhibitory members include Siglec-7 and Siglec-9, which are prominently expressed on NK cells and suppress NK-mediated cytotoxicity.^14–16^ Another member, Siglec-15, is an emerging immune checkpoint target currently under clinical investigation.^17–19^ Furthermore, Siglec-9, Siglec-10, and Siglec-11 are highly expressed on tumor-associated macrophages, where they function to inhibit phagocytosis.^9,20,21^ Siglec-10 is also expressed on hematopoietic cells, B and T cells, monocytes and eosinophils.^9,22^ CD24, also known as heat stable antigen (HSA), is a highly glycosylated glycosylphosphatidylinositol (GPI)-anchored protein^23,24^ which is recognized by Siglec-10 as a ligand and overexpressed on tumor cells such as ovarian cancer, breast cancer, nonsmall cell lung carcinoma, prostate and pancreatic cancers^25^ and shown to enhance the metastatic potential of malignant cells.^26^ The Siglec-10/CD24 interaction mediates immune suppression, which dampens danger-associated molecular pattern (DAMP)-induced inflammatory responses.^20,27^ Recent studies also suggest that CD24 is an innate immune checkpoint for tumor immune escape and is a promising target for cancer immunotherapy.^9,28^ The interaction of CD24-bearing tumors with Siglec-10 on macrophages inhibits phagocytosis and increases tumor growth and survival.^9^ Surface labeling of sialic acid residues on human CD24 by sodium periodate/^3^H-NaBH_4_ method revealed the presence of sialic acid residues on specific domains^29^ and removal of sialic acids from placental CD24 by *V. cholerae* neuraminidase showed a significant reduction in Siglec-10 binding by ∼ 60%.^30^ While FACS data showed interactions of Siglec-10 with wild-type MCF-7 cells, there was no apparent binding of Siglec-10 to neuraminidase-treated ΔCD24 MCF-7 cells.^9^

The immune checkpoint inhibition caused by interaction of Siglecs on immune cells, especially Siglec-7 and Siglec-9 on NK cells and Siglec-9 and Siglec-10 on macrophages, and sialyl glycans on cancer cells was shown to reduce the efficacy (effector function) of therapeutic antibodies via suppression of antibody-dependent cellular cytotoxicity (ADCC) and antibody-dependent cellular phagocytosis (ADCP).^9,10,31^ To block potential Siglec-mediated suppression of antibody-mediated effector functions, we have extensively studied the binding specificity of Siglecs with glycan arrays^32^ and evaluated the impact of Siglecs on therapeutic antibodies.^33^ We found that the ADCC activity of NK cells against pancreatic cancer was mainly from the subpopulation of NKG2D-expressing NK cells and the activity was enhanced in the presence of antibody against Siglec-9, a major Siglec on NK cells. We also found that the ADCP activity was enhanced by the antibody against Siglec-10, a major Siglec on macrophages. In addition, replacing the terminal N-acetylneuraminic acid residue of natural Siglec ligands with N-glycolylneuraminic acid would generally enhance Siglec binding and a truncated, unnatural α-2,3-linked glycolyl sialoglycan (R3) was found to be a potent binder of Siglec-10 with an apparent dissociation constant (*K*_*D*_^app^) around 50 nM on glycan array.^32^

Siglecs expressed on various immune cells can act as activating or inhibitory immune checkpoints to regulate immune responses through interaction with sialic acid-linked glycans on target cells. For example, immune cells with cytotoxic activity include cytotoxic T cells (CD8+ T cells). NK cells, monocytes, macrophages, and dendritic cells, and Siglec-7/9 on NK cells and Siglec-9/10 on macrophages are key inhibitory immune checkpoints of cytotoxicity. While Siglec-10 is expressed on various immune cells, mostly on B cells to help self-tolerance, its high expression on tumor associated macrophages along with Siglec-9/11 often promotes immune suppression of macrophage-mediated phagocytosis and cytotoxicity. Since Siglec-7/9/10 are the major players of immune check point inhibition and all recognize sialylated N-acetyllactosamine as a common motif,^10^ this study aims to develop a common small-molecule inhibitor of these Siglecs, thereby blocking their binding to cancer cells and enhancing cytotoxicity and phagocytosis as well as ADCC and ADCP in cancer immunotherapy. We thought developing a common small-molecule inhibitor of multiple Siglec-mediated inhibitory immune checkpoints would be a more cost-effective approach than use of multiple biologics, such as antibodies or bispecific-like constructs, to target specific immune checkpoints.

## Results

### Synthesis of a Library of Glycans for Assembly onto a Glycan Microarray and Screening for Binding to Siglec-10

In our previous study,^32^ we identified a promising unnatural glycan that can potentially be used to block the Siglec-10/CD24 interaction. The glycan candidate, R3, is a biantennary pentasaccharide with two terminal α2,3-glycolylneuraminic acids (Neu5Gcs) (Figure 1a). The binding of Siglec-10 to R3 on the glycan array was relatively strong, with an apparent dissociation constant (*K*_*D*_^app^) of 0.050 ± 0.011 μM.^32^ In order to identify a better candidate, in this study, we first prepared a panel of 60 glycans and assembled them as a glycan microarray, including bi- and triantennary N-glycans with LacNAc repeats terminated with N-acetylneuraminic acid (Neu5Ac) and N-glycolylneuraminic acid (Neu5Gc) for comparison of their binding to Siglec-10 (Figure S1). It was found that, in general, multiantennary glycans with terminal Neu5Gc exhibited stronger binding to Siglec-10 than the corresponding Neu5Ac derivatives and R3. However, since R3 is an unnatural glycan with smaller molecular weight and is a strong inhibitor of Siglec-10, we decided to use R3 as a lead structure and synthesized derivatives of R3 to compare their binding affinity toward Siglec-10 (Figure 1) (see detailed synthetic procedures in Supporting Information). As shown in Figure 1, the synthesis of the Gal_2_Man core was accomplished with NIS-promoted glycosylation between the thioglycoside donor I^34^ and mannosyl acceptor II^35^ to provide the fully protected trisaccharide, followed by Zemplén deacetylation to give diol III in 82% overall yield. Removal of the benzyl groups by hydrogenolysis produced the linker attached Gal2Man trisaccharide R1, which was used as a substrate for enzymatic α2,3-siaylation. Thus, treatment of R1 with bacterial α2,3-sialyltransferase from *Vibrio sp*. JT-FAJ-16 in the presence of Mg^2+^ and CMP-Neu5Ac or CMP-Neu5Gc afforded α2,3-sialosides R2 and R3, respectively, in satisfactory yields.

**Figure 1:**
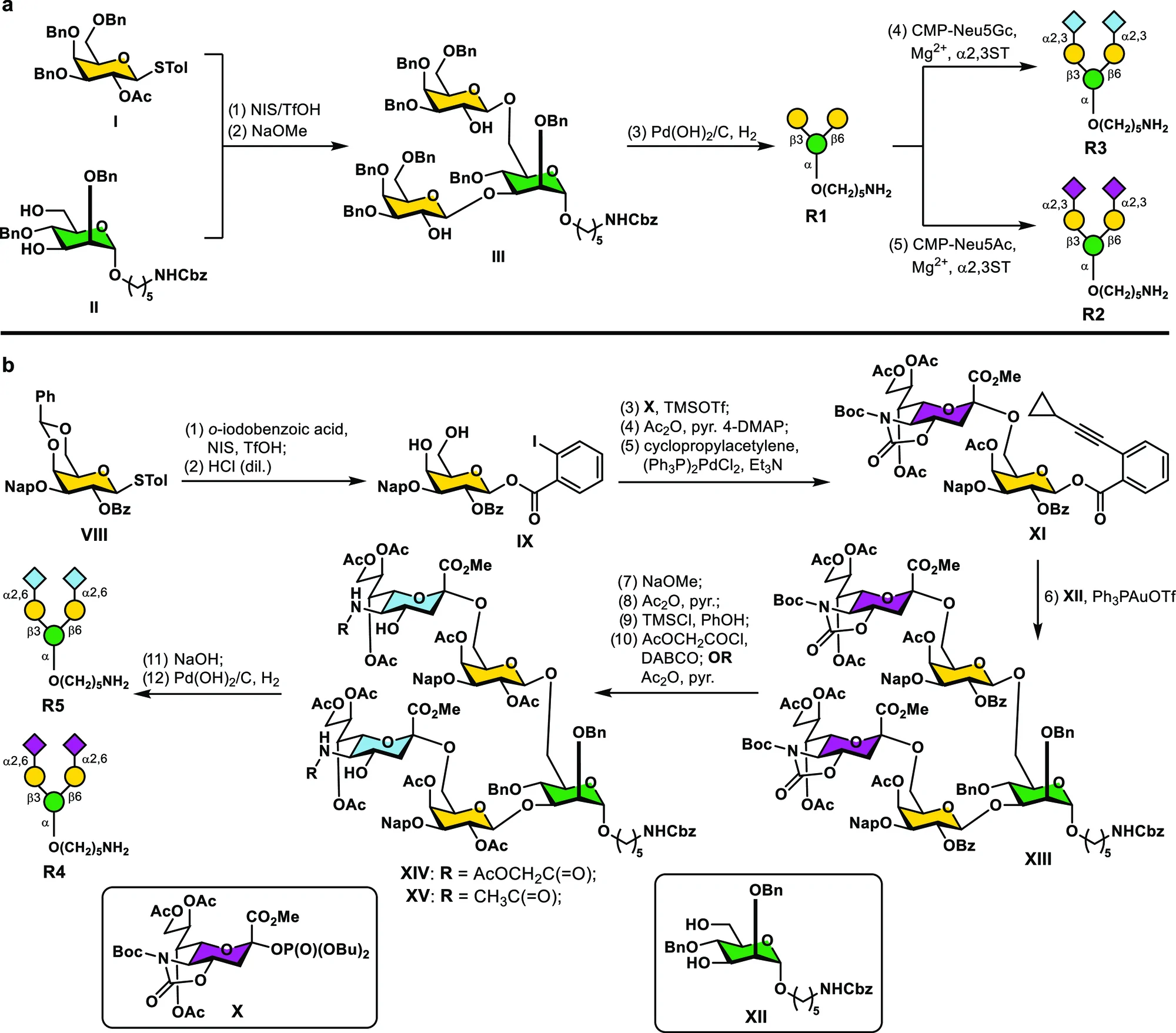
Synthesis of galactoside R1 and its derivatives R2–R5 (a) Reagents and conditions: (1) **I** (2.5 equiv), **II** (1.0 equiv), NIS (2.5 equiv), TfOH (0.5 equiv), 4Å MS, CH_2_Cl_2_, −40 → 0 °C, 1 h; (2) NaOMe (0.5 equiv), MeOH, r.t., 24 h, 82% overall; (3) Pd­(OH)_2_/C, H_2_ (balloon), THF:H_2_O:BuOH:AcOH (6:3:1:0.1, *v*/*v*/*v*/*v*), 24 h, 82%; (4) CMP-Neu5Gc (2.2 equiv), MgCl_2_ (20 mM), Tris·HCl (pH 7.5, 100 mM), α2,3-sialyltransferase (JT-FAJ-16), 89%; (5) CMP-Neu5Ac (2.2 equiv), MgCl_2_ (20 mM), Tris·HCl (pH 7.5, 100 mM), α2,3-sialyltransferase (JT-FAJ-16), 95%. (b) Reagents and conditions: (1) *o*-iodobenzoic acid (2.0 equiv), NIS (2.0 equiv), TfOH (0.2 equiv), 4Å MS, CH_2_Cl_2_, −40 → 0 °C, 1 h; (2) THF:*i*-PrOH:conc.HCl (20:50:1, *v*/*v*/*v*), 55 °C, 6 h, 73%; (3) **X** (1.1 equiv), TMSOTf (1.1 equiv), 4Å MS, CH_2_Cl_2_, −78 → −25 °C, 1 h; (4) Ac_2_O (3.0 equiv), pyridine (5.0 equiv), 4-DMAP (0.5 equiv), CH_2_Cl_2_, r.t., 24 h, 70% overall; (5) (Ph_3_P)_2_PdCl_2_ (0.1 equiv), CuI (0.2 equiv), cyclopropylacetylene (5.0 equiv), Et_3_N:DMF (3:1, *v*/*v*), r.t., 24 h, 88%; (6) **XII** (1.0 equiv), **XI-ABz** (2.5 equiv), Ph_3_PAuOTf (1.0 equiv), 4Å MS, CH_2_Cl_2_, 0 °C, 30 min, 52%; (7) NaOMe (10 equiv), MeOH, r.t., 24 h; (8) Ac_2_O:pyridine (1:3, *v*/*v*), r.t., 24 h; (9) TMSCl:PhOH (neat, 1:3, mol/mol), −78 → −20 °C, 1 h; (10) AcOCH_2_COCl (3.0 equiv), DABCO (3.0 equiv), CH_2_Cl_2_, 0 °C → r.t., 12 h; or Ac_2_O:pyridine (1:3, *v*/*v*), r.t., 12 h, 48% overall for **XIV**, 52% overall for **XV**; (11) NaOMe:MeOH:H_2_O (1:5:10, v/v/v), r.t., 48 h; (12) Pd­(OH)_2_/C, H_2_ (balloon), THF:H_2_O:BuOH:AcOH (6:3:1:0.1, *v*/*v*/*v*/*v*), 24 h, 75% overall for **R5**, 83% overall for **R4**.

Unfortunately, trisaccharide R1 could not be enzymatically α2,6-sialylated, as Gal_2_Man was not a substrate of any α2,6-sialyltransferase tested. Therefore, chemical synthesis was employed for the construction of α2,6-sialodes as outlined in Figure 1b. Fully protected sialyl galactosyl donor XII was synthesized from reported building blocks VIII^36^ and X^37^ in five steps. Coupling of O-alkynylbenozate donor VIII and mannosyl acceptor XII under the influence of Au­(I) catalyst smoothly afforded pentasaccharide intermediate XIII with complete β-stereoselectivity secured by the neighboring group participation effect and confirmed by NMR characterization, which served as a common precursor to both α2,6-Neu5Ac and α2,6-Neu5Gc sialosides. Sequential carbonate removal (NaOMe), peracetylation (Ac_2_O, pyridine), Boc removal (TMSCl, PhOH) followed by either N-acetylation (Ac_2_O, pyridine) or N-glycolylation (AcOCH_2_COCl, DABCO) delivered XV and XIV, respectively, in a protected form. Final deacetylation and debenzylation successfully produced α2,6-sialosides R4 and R5 in good yields.

### Preparation of R1-R5 as a Glycan Microarray For Identification of Siglec-10 Inhibitors

We prepared a glycan array of R1-R5, including the sialosides with 2,3- and 2,6-linkages and compared their binding affinities toward Siglec-10 (Figure 2b). Results showed that, for unsialylated R1, there was no binding detected. Glycans with terminal Neu5Gc (**R3** and **R5**) showed higher affinity compared to the glycans with terminal Neu5Ac (**R2**, **R4**), suggesting that Siglec-10 had stronger binding preference toward unnatural Neu5Gc glycans with 2,3-linkage over the 2,6-linkage and other Neu5Ac derivatives.

**Figure 2:**
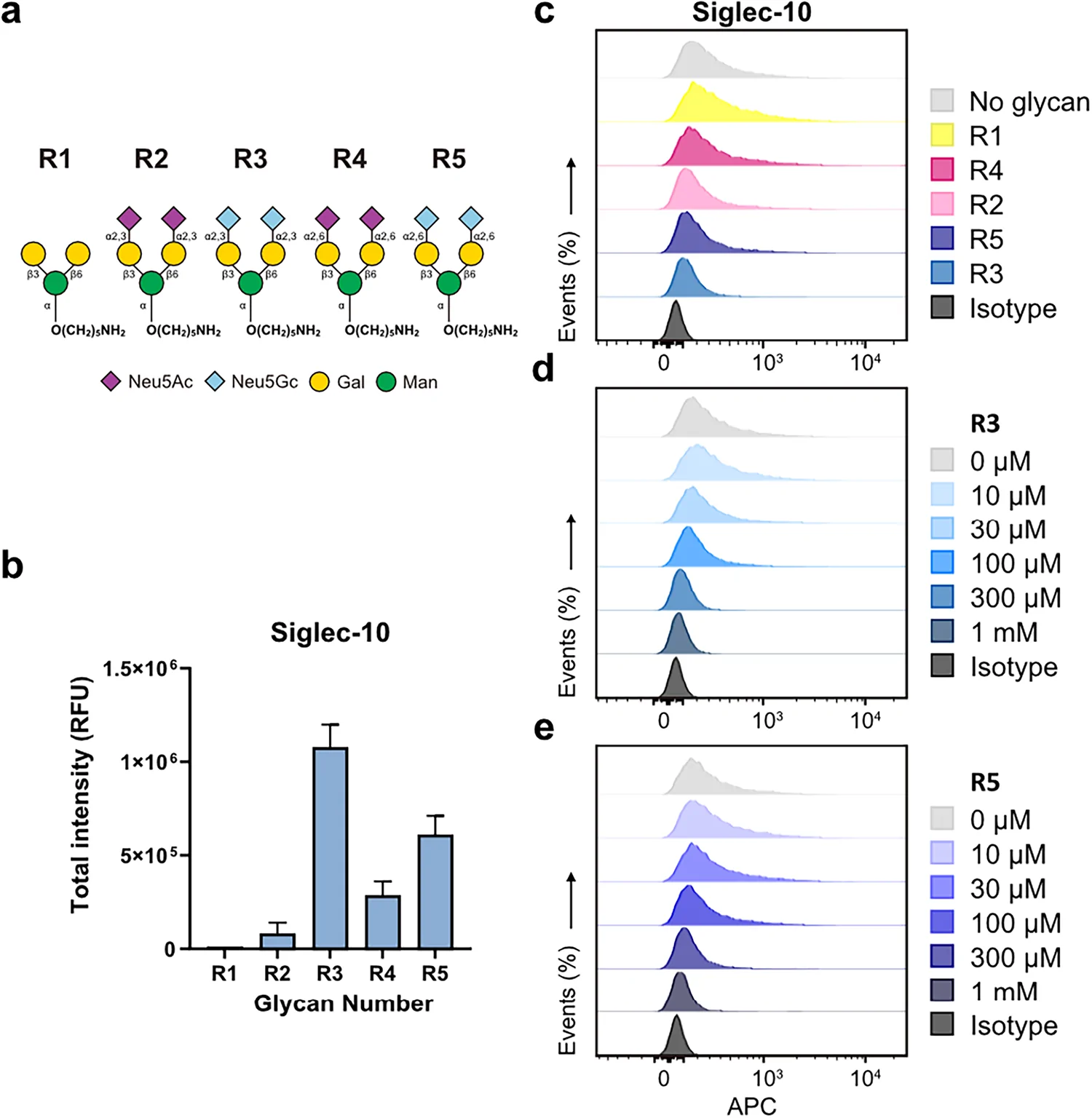
Siglec-10 binding to R3 and derivatives. (a) Structures of glycans R1 to R5. (b) Glycan array results of Siglec-10- Fc binding to glycans R1 to R5. Data are mean ± SD (c) Flow cytometry results of Siglec-10 binding to MCF-7 in the presence of R1 to R5 (200 μM), respectively. (d) Siglec-10 binding to MCF-7 at different R3 concentrations. (e) Siglec-10 binding to MCF-7 at different R5 concentrations.

We then tested the blocking ability of the synthetic glycans toward Siglec-10 interacting with HER2-low MCF-7 cancer cells via flow cytometry. In Figure 2c, all glycans were added respectively at the same concentration (200 μM). While the unsialylated R1 showed no apparent interference, the Neu5Gc R3 and R5 were then prepared at different concentrations for the binding assay (Figure 2d,e). With higher binding affinity to Siglec-10, **R3** showed significant blocking ability at a lower concentration (300 μM) compared to **R5** (1 mM). The results suggested that by adding the Neu5Gc-terminated glycans (**R3** or **R5**), the binding of Siglec-10 to MCF-7 cells was successfully interrupted, meaning that **R3** or **R5** could successfully compete with Siglec-10 ligands on cancer cell surfaces, including CD24. We also compared the binding results of Siglec-10 to wild type MCF-7 (WT) or CD24-knockout MCF-7 (CD24-KO). We found that Siglec-10 was still capable of binding to CD24-KO MCF-7, even without CD24 expression (Figure S3). This pointed out the variety of sialoglycan ligands of Siglecs present on cancer cells, and that using a glycan inhibitor such as **R3** or **R5** could be a good strategy to target inhibitory immune checkpoints.

### N-Glycan Profiling of HER2-low MCF-7 Cancer Cells by Mass Spectrometry Analysis

According to our glycan microarray data and flow cytometry results, Neu5Gc-glycan **R3** or **R5** is a better ligand for Siglec-10 binding than the corresponding glycans with terminal Neu5Ac. However, human cells normally lack the enzyme cytidine-5′-monophosphate-N-acetylneuraminic acid hydroxylase (CMAH) to convert Neu5Ac to Neu5Gc,^38^ hence humans do not express glycans terminated with Neu5Gc. Low traces of Neu5Gc found in humans were assumed to come from dietary acquisition.^39^ The higher levels of Neu5Gc-containing glycans found in human cancer cells^40^ suggest the potential of using Neu5Gc-glycoconjugates as cancer biomarkers.^41^

The N-glycan profiling of MCF-7 cells was performed using mass spectrometry. MCF-7 cells were collected, lysed, and the lysate was treated with PNGase F to collect the N-glycans. According to the mass spectrometry results (Figure S4), most of the compositions were high mannose glycans (∼70%), accompanied by various complex-type N-glycans (bi-, tri-, tetraantennary) with low percentage of sialic acid-containing glycans (less than 10%) (Table S1). Most sialic acids detected were Neu5Ac, and among the few that contained Neu5Gc had only one Neu5Gc residue. The overall sialic acid composition in total lysate of MCF-7 cells was further analyzed with MALDI-ISD (Figure S5). Results showed that most sialic acids were Neu5Ac (over 97%), with traces of Neu5Gc. To analyze the glycosidic linkages of sialic acids, the relative intensity ratios (RIR) of diagnostic ions were calculated.^42^ RIR value (0.62) was obtained as the signal intensities of *m*/*z* 290.1 and 308.1 divided by the intensity at *m*/*z* 366.1 for Neu5Gc, indicating a mixture of α-2,3 and α-2,6.

### Evaluation of R3 and Related Structures as Inhibitors of Other Siglecs

Because the natural sialoside ligands recognized by Siglec-10 are structurally similar to those recognized by other Siglecs associated with immune checkpoint inhibition, notably Siglec-7/9 on human natural killer (NK) cells (which inhibits cytotoxicity) and Siglec-9/11 on macrophages (which inhibits phagocytosis), we evaluated the interactions of **R2**–**R5** with a broader panel of Siglecs using a glycan microarray (Figure 3). Based on our pentasaccharide scaffold, substituting Neu5Ac (**R2**, **R4**) with Neu5Gc (**R3**, **R5**) dramatically increased binding toward Siglec-7, -10, and -11. For instance, Siglec-10 binding to the α2,3-linked ligand increased over 7-fold when transitioning from Neu5Ac (**R2**) to Neu5Gc (**R3**), consistent with previous reports demonstrating that Siglec-10 exhibits distinct terminal epitope- and branch-dependent preferences, displaying significant avidity differences across specific N-glycan structural isomers and their Neu5Gc-containing counterparts.^43^ Similarly, Siglec-11 exhibited near-zero binding to Neu5Ac constructs (**R2**, **R4**) but bound robustly to Neu5Gc derivatives (**R3**, **R5**). Although humans do not endogenously synthesize Neu5Gc, human CD33-related Siglecs retain binding pockets capable of accommodating, and often favoring, the additional hydroxyl group on the *N*-glycolyl moiety of Neu5Gc via hydrogen bonding.^44^

**Figure 3:**
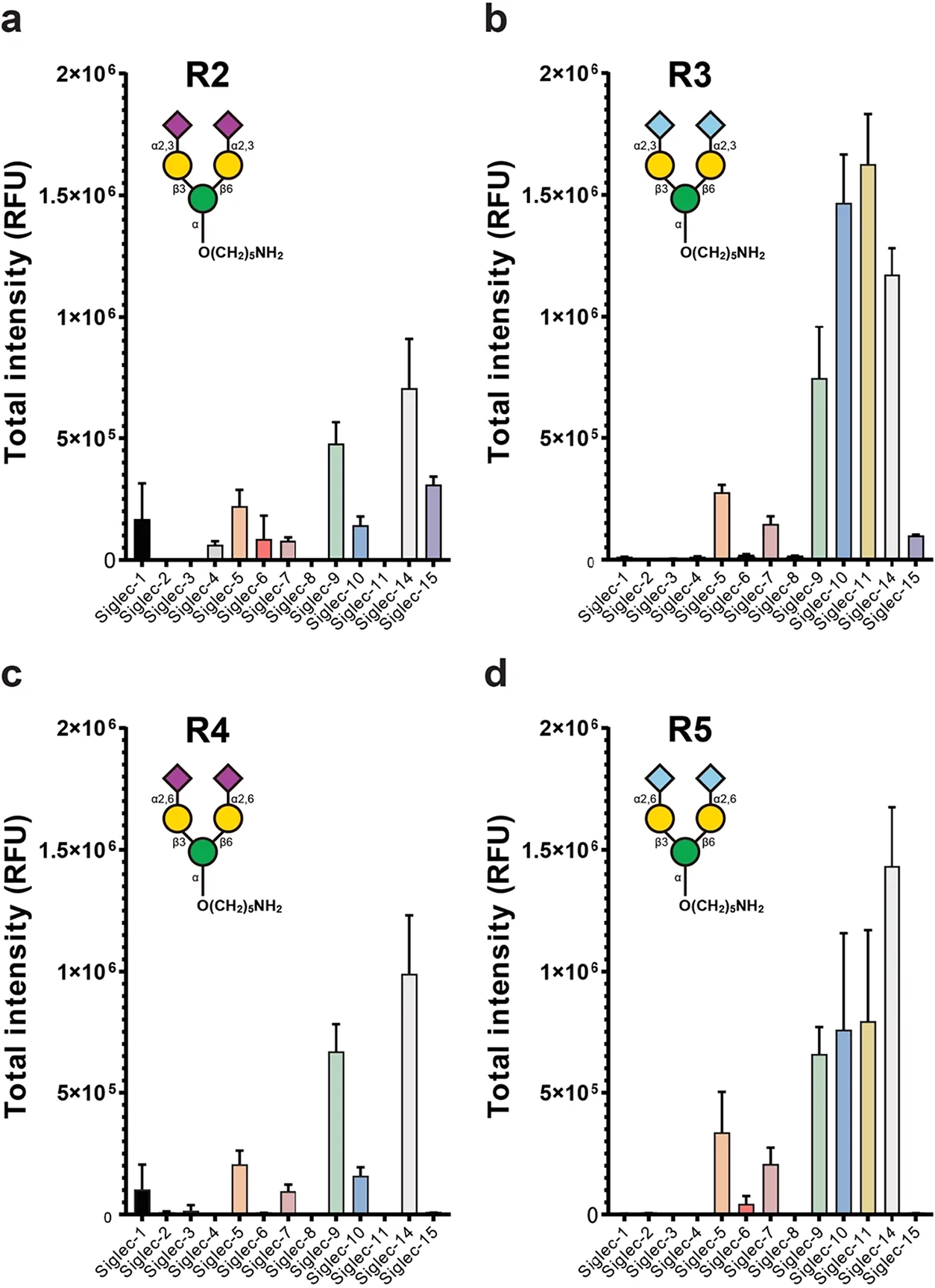
Human siglecs binding toward (a) R2, (b) R3, (c) R4, and (d) R5. Recombinant human siglecs: Siglec-1, 2, 3, 4, 5, 6, 7, 8, 9, 10, 11, 14, and 15. Siglec concentration: 5 μg/mL. Data are mean ± SD.

While Siglec-7 and -9 traditionally demonstrate strong preference for Neu5Ac-terminated epitopes, the structural tolerance and enhanced binding for Neu5Gc modifications across certain Siglec family members suggest that unnatural glycans terminated with Neu5Gc could serve as a handle to modulate binding selectivity between Siglec-7, -9, and -10. Siglec-9 displayed high affinity toward all four synthetic candidates (**R2-R5**) with minimal linkage bias, aligning with literature reports showing that Siglec-9 broadly recognizes both α2,3- and α2,6-sialoglycans.^45^ Specifically, Siglec-9 demonstrated robust binding to both α2,3- and α2,6-linked Neu5Gc, with slightly enhanced binding toward **R3**. A direct comparison of **R3** (α2,3-linked Neu5Gc) and **R5** (α2,6-linked Neu5Gc) binding to Siglec-10 revealed a clear preference for the α2,3-linkage; although canonical natural ligands of Siglec-10 typically favor α2,6-linkages,^45^ this altered specificity is likely attributable to the truncated, unnatural biantennary architecture of the synthetic scaffold. Collectively, these results demonstrate that major inhibitory checkpoints, including Siglec-5, -7, -9, -10, and -11, all interact strongly with **R3**. Because Siglec-7 and -9 on NK cells^15,46,47^ and Siglec-9, -10, and -11 on macrophages^34,35^ function as primary inhibitory checkpoints suppressing immune effector functions, their robust recognition of **R3** highlights its potential as a common immune checkpoint blocker to enhance antitumor immunity and antibody-mediated cell killing in immunotherapy.

### Enhanced Phagocytosis and Cytotoxicity Against MCF-7 Cells in the Presence of R3

To test whether the glycan blocker **R3** could disrupt inhibitory immune checkpoint signaling during phagocytosis, differentiated human macrophages were cocultured with MCF-7 cells in the presence or absence of **R3** (Figure 4). After 2 h of coculture, we found that **R3** enhanced phagocytic activity across all naïve and differentiated macrophage subsets compared to the PBS control. Anti-inflammatory M2-like macrophages^48,49^ (induced by IL-4 or IL-10) were affected most significantly by **R3**, with average phagocytosis rates enhanced by 56.2 and 137.9%, respectively, in the presence of **R3** (Figures 4d and 4e). Unactivated M0-like macrophages were also influenced by **R3**, yielding an 82.2% enhancement in phagocytosis (Figure 4b). Conversely, pro-inflammatory M1-like macrophages (induced by LPS and IFN-γ), which already possessed high baseline phagocytic activity, were less enhanced by **R3** among the four subsets (+22.7%, Figure 4c), reflecting their activated pro-inflammatory phenotype.^50,51^

**Figure 4:**
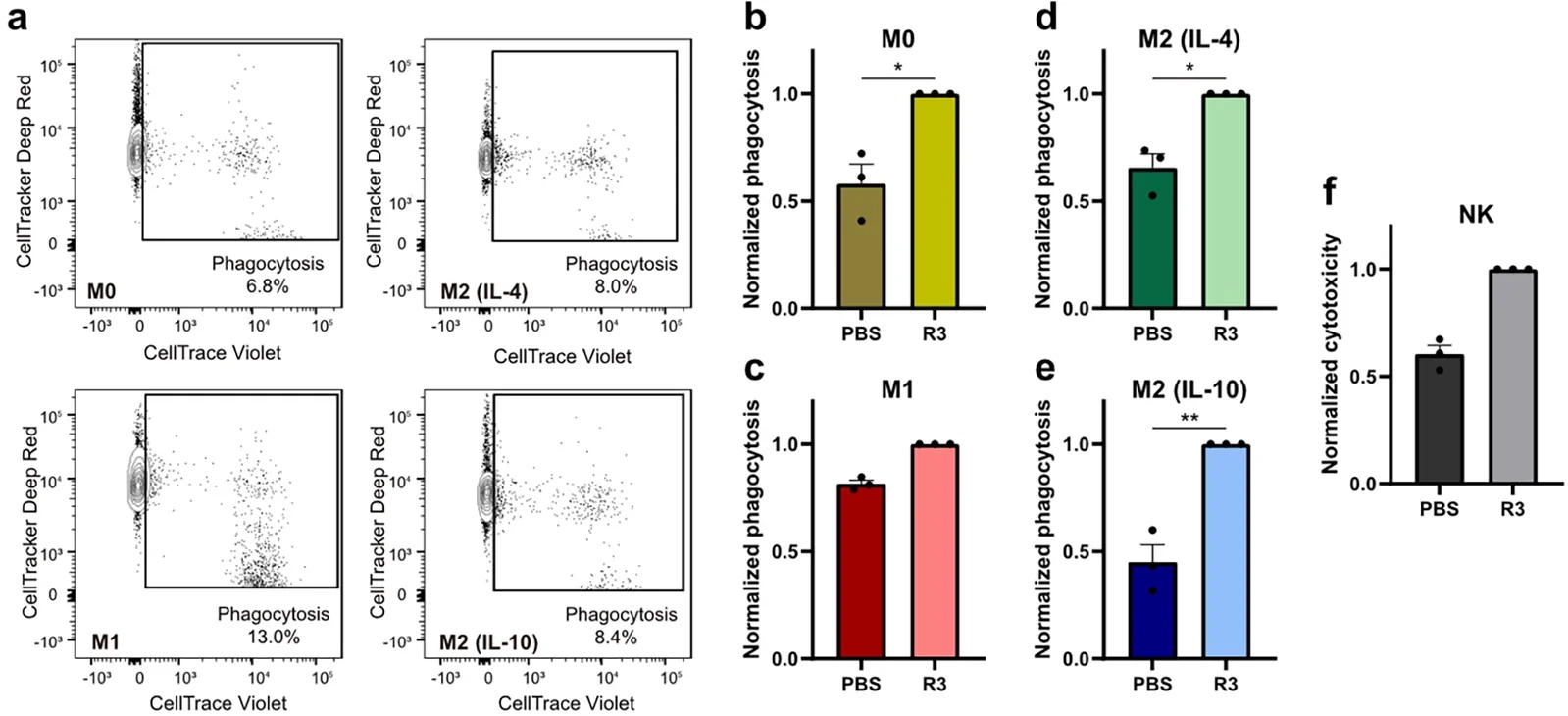
In vitro phagocytosis assay with flow cytometry. (a) Phagocytosis of M0-like, M1-like, or M2-like (IL-10-/IL-4-induced) macrophages to MCF-7. Normalized phagocytosis of (b) M0-like, (c) M1-like, (d) M2-like (IL-10-induced), and (e) M2-like (IL-4-induced) macrophages. Phagocytoses were normalized to the highest value of each experiment (*n* = 3 donors). (f) Cytotoxicity of NK cells to MCF-7 in the presence of 2 mM R3 or PBS control. Cytotoxicity activities were normalized to the highest value of each experiment (*n* = 3 donors). Statistical significance was determined using a paired, two-tailed *t* test on the original raw data across independent biological replicates (*n* = 3 donors; *p < 0.05, **p < 0.01) Data are mean ± SEM.

To assess cellular cytotoxicity, natural killer (NK) cells were cocultured with MCF-7 cells at an effector-to-target (E:T) ratio of 10:1. After 6 h of incubation, NK cell-mediated cytotoxicity increased by 54.4% in the presence of **R3** (Figure 4f). Furthermore, macrophage-mediated cytotoxicity was enhanced by at least 46.3% under identical conditions (Figure S7). These results confirm that this Siglec-targeting glycan efficiently enhances anticancer immune responses.

### Combination of R3 and Therapeutic Antibody for Cancer Targeting

In addition to acting as a standalone immune checkpoint inhibitor, **R3** was found to enhance antibody-mediated effector functions. As shown in Figure 5c, 1 mM **R3** alone induced an enhancement in M2-like macrophage phagocytosis comparable to 0.02 μg/mL trastuzumab in antibody-dependent cellular phagocytosis (ADCP) assays (100.4% enhancement for **R3** alone vs 145.3% for trastuzumab alone) compared to PBS control. Remarkably, when **R3** and trastuzumab were coadministered, phagocytosis increased by 258.9%. A similar enhancement was observed in M0-like macrophages, where phagocytosis increased by 70.0% (**R3** alone), 80.3% (trastuzumab alone), and 198.8% when combined (**R3** + trastuzumab). These results indicate that **R3** is an effective inhibitor of inhibitory glyco-immune checkpoints that significantly potentiate trastuzumab-mediated phagocytosis.

**Figure 5:**
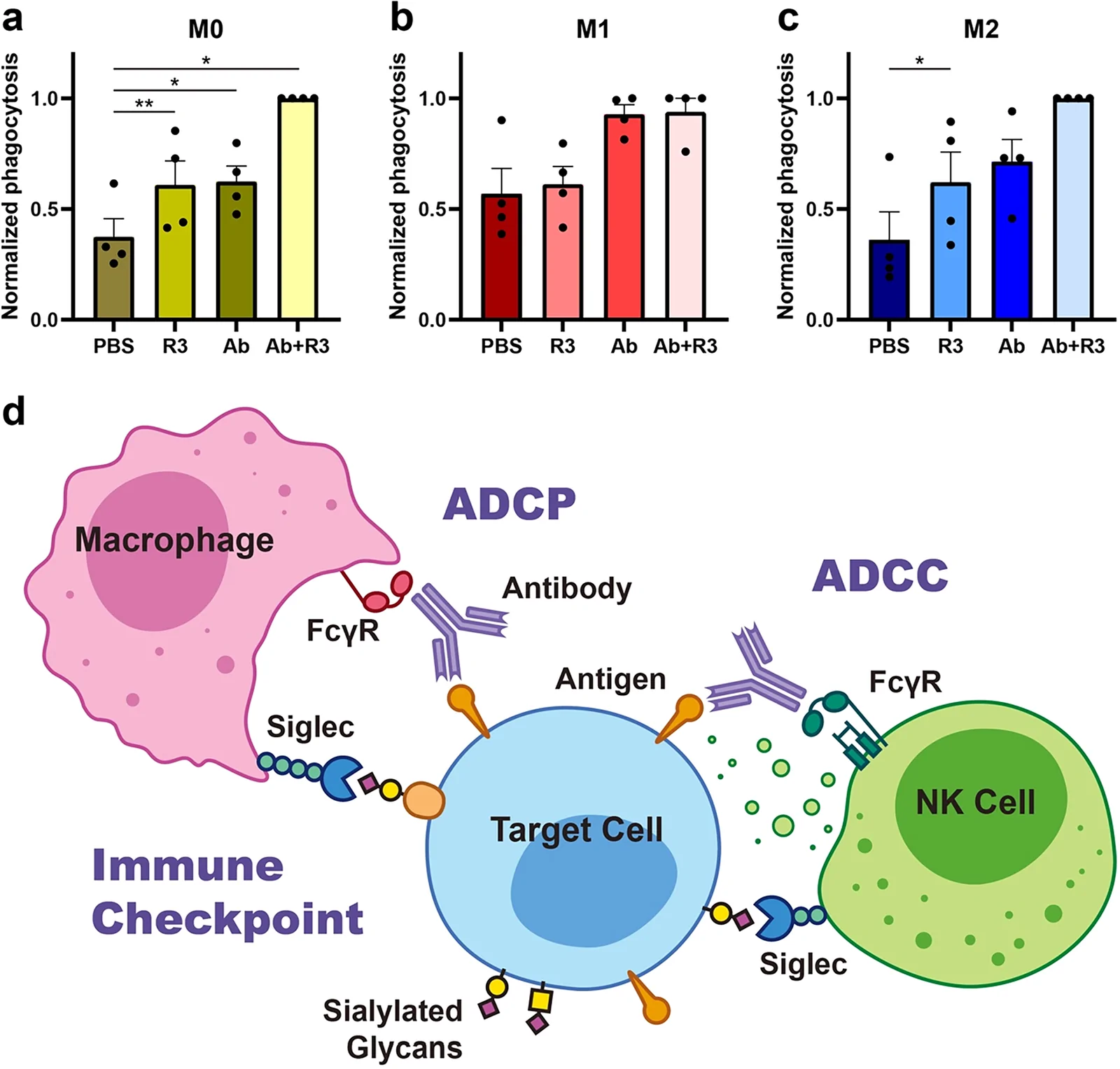
Combination of immune checkpoint blockade and ADCP to enhance anticancer activity. (a) M0-like, (b) M1-like and (c) M2-like (IL-10-induced) macrophages. R3 = 1 mM; Trastuzumab (Tras) = 0.02 μg/mL. Phagocytoses were normalized to the highest value of each experiment (*n* = 4 donors). Statistical significance was determined using a paired, two-tailed *t* test on the original raw data across independent biological replicates (*n* = 4 donors; *p < 0.05, **p < 0.01) Data are mean ± SEM (d) Immune checkpoint and Fc effector function in cancer immunotherapy.

## Discussion

Cancer immunotherapy has revolutionized oncology by harnessing the immune system to target and destroy tumors, as demonstrated by the clinical success of anti-PD-1, anti-PD-L1, and anti-CTLA-4 monoclonal antibodies as immune checkpoint inhibitors.^52,53^ These therapies function by suppressing inhibitory signaling pathways in T cells, thereby restoring their ability to recognize and eradicate cancer cells. In this study, we turned our attention to the clinically underexplored Siglec family, initially aiming to identify small-molecule glycans capable of acting as competitive blockers to disrupt the inhibitory checkpoint pathway mediated by the Siglec-10-CD24 interaction. Using glycan microarray screening, we identified a high-affinity, Neu5Gc-containing binder, **R3**, which successfully interrupted the binding between Siglec-10 and HER2-low MCF-7 breast cancer cells.

To systematically evaluate this lead scaffold, **R3** and a focused series of related structures were chemoenzymatically and chemically synthesized. Consistent with our initial observations, unnatural Neu5Gc-glycans exhibited substantially stronger binding toward Siglec-10 compared to natural biantennary *N*-glycans terminating in Neu5Ac (Figures S1 and S2).^32^ When we assembled **R1**–**R5** onto a dedicated glycan microarray to investigate their broad-spectrum interactions across the Siglec family (Figure 3), we discovered that in addition to Siglec-10, the inhibitory checkpoint receptors Siglec-7, -9, and -11 also exhibited robust binding to **R3**. Because Siglec-7 and -9 serve as primary inhibitory checkpoints on natural killer (NK) cells,^15,46,47^ and Siglec-9, -10, and -11 function as critical immunosuppressive receptors on macrophages,^54,55^ our findings demonstrate that **R3** acts as a common inhibitor targeting multiple major inhibitory Siglecs simultaneously. Furthermore, **R3** displayed strong affinity toward the inhibitory checkpoint receptor Siglec-5 as well as the activating checkpoint receptor Siglec-14.^56^

The signaling of inhibitory Siglecs, similar to PD-1, requires clustering of Siglec molecules to trigger downstream intracellular signaling or inhibitory responses. Engagement with sialylated ligands promotes Siglec clustering and phosphorylation of intracellular ITIMs, facilitating recruitment of SHP-1/SHP-2 phosphatases.^57,58^ It has been proposed that multivalent Siglec ligands may induce Siglec clustering that leads to inhibitory signaling for immune cells.^57–60^ In our study, we found that using high affinity monomeric sialoglycan **R3**, not only could successfully compete with the sialoglycan ligands on the cancer cell surfaces, but also inhibit the immune checkpoint response to enhance phagocytosis and cytotoxicity. We hypothesize that R3 may adopt a novel mode of binding that could prevent activation of the downstream inhibitory immune pathway. Our findings may provide a new direction to design immune checkpoint inhibitors.

In summary, our functional assays demonstrate that the enhancement of macrophage-mediated phagocytosis and NK cell-mediated cytotoxicity induced by **R3** directly correlates with its *in vitro* binding affinity for these corresponding inhibitory Siglecs. Recent clinical and translational advances have underscored the critical interplay between immune checkpoint inhibition and Fc-mediated effector functions during antibody therapy. By engaging Fc receptors on innate immune cells, therapeutic antibodies mediate cellular effector mechanisms such as ADCC and ADCP, which not only directly eliminate tumor cells but also help deplete immunosuppressive populations—such as regulatory T cells and myeloid-derived suppressor cells—within the tumor microenvironment. Combining glyco-immune checkpoint inhibition with optimized Fc-mediated effector functions offers a synergistic dual approach: reactivating adaptive antitumor immunity while simultaneously unlocking innate immune effector mechanisms for superior therapeutic efficacy (Figure 5).^61–63^

The robust, broad-spectrum binding of **R3** toward Siglec-7, -9, -10, -11, and -15 suggests that this biantennary scaffold can serve as a prime structural foundation for developing a common small-molecule checkpoint blocker to potentiate antitumor immunity and enhance clinical antibody effector functions (ADCC/ADCP). Our data also suggests that **R3** likely engages a structurally conserved ligand-binding pocket shared among these CD33-related Siglecs to alleviate immune suppression. Structural studies, including X-ray crystallography of **R3** in complex with target Siglecs, are currently underway to elucidate the precise molecular mechanisms of inhibition. These structural insights will guide the rational modification and optimization of **R3** into an effective, common small-molecule inhibitor targeting multiple inhibitory glyco-immune checkpoints for advanced cancer immunotherapy.

## Materials and Methods

### General Chemical Synthesis

All chemicals were purchased as reagent grade and used without further purification. Detailed syntheses and NMR spectra are listed in the Supporting Information.

### Statistics

Statistical analyses were performed in GraphPad Prism 10.

### Cell Culture

Human breast cancer cell lines MCF-7 (HTB-22, the American Type Culture Collection, WT) and CD24-knockout MCF-7 (KC-1753, KYinno Biotechnology, CD24-KO) were cultured in RPMI 1640 medium (Gibco) supplemented with 10% fetal bovine serum (FBS), 1 × nonessential amino acids (Gibco) and 1 × antibiotic-antimycotic (Gibco).^64^ The cells were cultured in a 37 °C incubator with 5% CO_2_ and humidified atmosphere control. Culture medium was changed every 3 to 4 days.

### MCF-7 total N-Glycan Mass Spectrometry Analysis

MCF-7 cells detached from the culture dish surface using TrypLE Express (Gibco) and washed with 1 × PBS. The collected pellet was lysed with 1 × RIPA buffer (Millipore) on ice for 30 min and centrifuged to remove cell debris. Lysate protein concentrations were determined using the BCA Protein Assay Kit (Pierce). Protein samples were treated with PNGase F (New England BioLabs), and glycans were purified with PGC column before mass spectrometry analysis. Sample preparation and mass spectrometry analysis were performed by the Academia Sinica Glycoscience Core Facility.

### Glycan Microarray

Glycan microarrays were fabricated by printing 100 μM glycan solution in 1 × printing buffer (300 mM sodium phosphate buffer (pH 8.5) with 0.01% Triton X-100) onto NHS-activated glass slides (Nexterion Slide H, SCHOTT).^65^ Glycan microarrays slides were blocked with SuperBlock Blocking Buffer in PBS (Thermo Fisher Scientific) at RT for 1 h, and washed three times with 1 × PBS buffer containing 0.05% Tween-20 (PBST). Recombinant human Siglec proteins (Siglecs-1, 2, 3, 4, 5, 6, 7, 8, 9, 10, 11, 14 and 15, R&D Systems) was serially diluted with PBST containing 30 mg/mL BSA (PBST-BSA), and added to the array for 1 h. After 3 washes with PBST, 2.5 ng/mL Alexa Flour 647-conjugated antihuman IgG antibody (donkey antihuman IgG, Fcγ fragment specific, Alexa Flour 647, Jackson ImmunoResearch) or Alexa Flour 647-conjugated anti-His antibody (Jackson ImmunoResearch) in PBST-BSA was added to the array and incubated at R.T. for 1 h. Finally, the microarray slides were washed with PBST, Milli-Q, and then dried. The microarrays were excited with 635 nm laser and scanned with GenePix 4300A Microarray Scanner (Molecular Devices), and the fluorescence intensities were analyzed by the GenePix Pro 7.0 software (Molecular Devices). Data analysis was done using GraphPad Prism 10.^65,66^

### Flow Cytometry

MCF-7 cells were detached from the dish surface with TrypLE Express, and washed with ice-cold FACS buffer (1% FBS in 1 × DPBS) before staining with the following antibodies: (1) For CD24 visualization: CD24 Monoclonal Antibody (eBioSN3 (SN3 A5-2H10)), PE (Invitrogen); isotype control: Mouse IgG1 kappa Isotype Control (P3.6.2.8.1), PE (Invitrogen). (2) For Siglec-10 binding/inhibitory experiments, 5 × 10^5^ MCF-7 cells were incubated with 10 μg/mL Siglec-10-Fc, along with either glycans R1, R2, R3, R4 or R5, at different concentrations in FACS buffer for 1 h on ice. Cells were washed with FACS buffer and stained with Alexa Flour 647-conjugated antihuman IgG antibody. IgG1, Kappa from human myeloma plasma (Sigma-Aldrich) was used as isotype control. Flow cytometry was performed on FACSCanto flow cytometer (BD Bioscience) and analyzed using FlowJo software (BD).

### Macrophage Differentiation

Human peripheral blood mononuclear cells (PBMCs) from healthy donors were purchased from Charles River Laboratories. Human PBMCs were thawed in 37 °C water bath, diluted in RPMI medium (1 × RPMI 1640 with 10% FBS and 1 × Penicillin-Streptomycin, Gibco), and centrifuged before the removal of cryopreservation solution. Cell pellet was washed and resuspended in 1 × MACS buffer (1 × PBS with 2 mM EDTA and 0.5% BSA). Monocytes were purified using CD14 MicroBeads (Miltenyi Biotec) following the provided instructions. CD14+ monocytes were then cultured in RPMI medium supplemented 50 ng/mL M-CSF (PeproTech) for 3 days, then differentiated into M1-like macrophages (M1 macrophages) with M-CSF with 10 ng/mL Lipopolysaccharide (LPS, Sigma) and 50 ng/mL Interferon γ (IFN-γ, Fujifilm) from day 3; for M2-like macrophages (M2 macrophages), cells were treated with M-CSF with 50 ng/mL Interleukin-10 (IL-10, PeproTech) or 20 ng/mL Interleukin-4 (IL-4, ABclonal) from day 3 until harvest (day 8 to 10).

### NK Cell Isolation

NK was isolated from the human PBMC CD14– flowthrough using the NK Cell Isolation Kit (Miltenyi Biotec) after monocyte purification in the previous section. Purified NK cells were cultured in NK MACS Basal Medium (with 1 × NK MACS Supplement; Miltenyi Biotec) with 5% human AB Serum (Sigma-Aldrich) and 500 IU/mL IL-2 (Miltenyi Biotec) for 2 days before the cytotoxicity assay.

### *In Vitro* Phagocytosis Assay

Differentiated macrophages and target cells (MCF-7) cells were detached from the dish surface with TrypLE Express. Cells were washed with 1 × PBS before fluorescent staining. Macrophages were stained with CellTracker Deep Red (Invitrogen) and MCF-7 cells were stained with CellTrace Violet (Invitrogen), according to the manufacturer’s instructions. Fluorescently labeled macrophages and target cells were cocultured at an effector to target ratio (E:T) of 1:2 (75,000 to 150,000 cells/well) in RPMI medium in ultralow attachment 96-well U-well plates (Millipore) for 2 h at 37 °C with 5% CO_2_. Cells were then collected and observed with FACSCanto flow cytometer and analyzed using FlowJo software. Phagocytosis was defined as the percentage of CellTrace Violet+ signal among macrophages (CellTracker Deep Red+) using PBS as control. Phagocytosis was normalized to the highest value for analysis.

### Cytotoxicity Assay

NK/Macrophage-mediated cytotoxicity was quantified using the LDH-Glo Cytotoxicity Assay kit (Promega) following the manufacturer’s instructions. Effector cells (NK cells or macrophages) were cocultured with MCF-7 at a E:T ratio of 10:1 (50,000 to 5,000 cells/well) in a 96-well plate in low-serum medium (for NK: 0.5% human AB Serum in NK MACS Basal Medium (with 1 × NK MACS Supplement) and 500 IU/mL IL-2; for macrophages: 2% low IgG FBS (Cytiva) in RPMI) in the presence of 2 mM R3 or PBS control for 6 h at 37 °C with 5% CO_2_. Maximum LDH release control was determined by lysing vehicle-only cells in a 0.2% Triton X-100. Postincubation, cell-free supernatants were harvested, and diluted 100 times in LDH storage buffer (200 mM Tris-HCl (pH 7.3) with 10% glycerol and 1% BSA). Bioluminescence from released LDH was measured using CLARIOstar Plus plate reader (BMG Labtech). Experiments were performed in triplicate and repeated three times. Relative luminescent units (RLU) were normalized to the highest value for analysis.

### Antibody Preparation

Anti-Her2 antibody Trastuzumab was prepared for ADCP. Trastuzumab expression vectors were transfected into ExpiCHO-S (Thermo Fisher Scientific) using the ExpiFectamine CHO Transfection Kit (Thermo Fisher Scientific) according to the manufacturer’s instructions. ExpiCHO-S cells were cultured at 37 °C with 8% CO_2_ in a humidified incubator on an orbital shaker at 125 rpm. Culture supernatant was harvested 7 days post-transfection and clarified by centrifugation. Recombinant antibodies were purified using Protein A affinity chromatography (Cytiva).

## Supplementary Material



## Data Availability

All data generated in this study will be freely available to the public after publication and deposited in the NIH-supported open-access repositories. The core structures and the *N*-glycans generated in this work will be available upon request after publication. In addition, the *N*-glycans and the binding profiles will be incorporated into the database of the Genomics Core Facility at the Scripps Research Institute and available to the public after publication.

## Notes

Synthetic and experimental procedures, supporting figures and characterization of compounds, glycan microarray assays and NMR data (PDF)
