# Supplementary material for: A Common Small-Molecule Inhibitor Targeting Multiple Siglecs Associated with Immune Checkpoint Suppression of Cytotoxicity and Phagocytosis
Source: J Am Chem Soc. 2026 Sep 29;148(40):43743–53. doi: 10.1021/jacs.6c15475 (PMC13674246; doi:10.1021/jacs.6c15475)
Supplement: Supplementary file 1 [file ja6c15475_si_001.pdf]

Supporting Information

A Common Small-Molecule Inhibitor Targeting Multiple Siglecs Associated with Immune Checkpoint Suppression of Cytotoxicity and Phagocytosis

Cinya Chung[†a], Ruofan Li[§a], Li-Chun Cheng[†a], Chen-Yo Fan[†], Kuo-Shiang Liao[†], Chih-Chuan Kung[†], Chi-Huey Wong[†§*]

[†]Genomics Research Center, Academia Sinica, 128 Academia Rd., Sec. 2, Taipei 11529, Taiwan

[§]Department of Chemistry, The Scripps Research Institute, 10550 N. Torrey Pines Rd., La Jolla, CA 92037, USA

[a]Equal contribution

[*]Corresponding Author: Chi-Huey Wong

Email: wong@scripps.edu

Table of Contents

General Information **2**

General Procedures for glycan synthesis **3**

Figure S1. Profiling of human Siglec-10 with *N*-glycan array **4**

Figure S2. Synthesis of galactoside R1 and its sialylated derivatives R2–R5 .... **5**

Figure S3. Flow cytometry sample gating for MCF-7 .... **6**

Figure S4. N-glycan profiling of MCF-7 cells **7**

Table S1. Neu5Gc percentages in complex-type N-glycans **8**

Figure S5. MALDI-ISD spectra of MCF-7 Neu5Ac- and Neu5Gc-containing glycans **9**

Figure S6. Gating strategy of *in vitro* phagocytosis with flow cytometry **10**

Figure S7. Macrophage cytotoxicity activity enhanced by R3 .... **11**

Table S2. Summary of preparation, ^{1}H NMR and LRMS data of 31–56 .... **12**

Synthesis of Glycans A–C, 44, 45 and 48 .... **21**

Scheme S1. Synthesis of Trisaccharide Intermediate **A** 21

Scheme S2. Synthesis of Tris-galactoside Acceptor **B** 22

Scheme S3. Synthesis of Pentasaccharides **44** and **45** 24

Scheme S4. Chemical Synthesis of Sulfated Trisaccharide **48** 28

Scheme S5. Synthesis of G2-Asn Glycan from SGP by Enzymatic Digestion 32

Reference **33**

^{1}H, ^{13}C and 2D-NMR Spectra **34**

General Information

All the organic reactions were performed under inert atmosphere (nitrogen or argon) using anhydrous solvents unless otherwise noted. All chemicals were purchased as reagent grade and used without further purification. Anhydrous dichloromethane (CH_2Cl_2), acetonitrile (CH_3CN), *N*,*N*-dimethyl formamide (DMF), toluene, methanol (MeOH), tetrahydrofuran (THF), *tert*-butanol (^{t}BuOH), *n*-butanol (^{n}BuOH) were purchased from commercial sources and were used without further distillation. Pulverized molecular sieves MS-3Å, MS-4Å and MS-5Å for glycosylation reactions were activated by flaming drying under high vacuum. All the reactions were monitored by analytical thin-layer chromatography (TLC) using silica gel 60 F254 plates and visualized under UV (254 nm) and/or by acidic ceric ammonium molybdate (CAM) or *p*-anisaldehyde. Flash chromatography was performed on silica gel (Fischer Chemical or Silicycle Inc.) of 40-63 µm particle size. Size exclusive chromatography was performed on P2 gel (Bio-rad, 45-90 µm) or Sephadex G-25 (Cytiva, superfine). ^{1}H NMR spectra were recorded on a Bruker AV NEO 500 MHz NMR and Bruker AVIII HD 600 MHz NMR spectrometers at 25 °C. All ^{1}H chemical shifts were calibrated using solvent residue peaks of $CDCl_3$ (δ = 7.26 ppm, s), MeOD (δ = 3.31 ppm, quint) and D_2O (δ = 4.79 ppm, s). ^{13}C NMR spectra were acquired on Bruker AVIII HD 600 MHz NMR spectrometer at 25 °C and all ^{13}C chemical shifts were calibrated using deuterated standards $CDCl_3$ (δ = 77.16 ppm, t) and MeOD (δ = 49.00 ppm, hept). Coupling constant values (*J* values) are reported in hertz (Hz). Splitting patterns are described using the following abbreviations: s, singlet; brs, broad singlet; d, doublet; t, triplet; q, quartet; quint, quintet; hept, heptet; dd, doublet of doublet; m, multiplet. High resolution ESI mass spectra were recorded on an Agilent 6230B time-of-flight LC/MS (LC/TOF) spectrometer.

All the enzymes used in this study, including *Bos taurus* galactosyltransferase (β4GalT1), *Vibrio* sp. bacterium (JT-FAJ-16) α2,3-sialyltransferase, human α2,6-sialyltransferase (ST6Gal1) and *Photobacterium damselae* α2,6-sialyltransferase double mutant (Pd2,6ST A200Y/S232Y) were expressed and purified as reported in the previous literatures.[1]

General procedures for glycan synthesis

The synthesis and characterization of *N*-glycan structures **1–30** were previously reported[1]. *N*-glycan structures **57–60** were from the collection of Consortium for Functional Glycomics at the Scripps Research Institute. Glycans **31–56** were synthesized either chemically or chemoenzymatically as described below.

I. **Enzymatic α2,3-Sialylation:** to a solution of terminal Gal *N*-glycan substrate (1.0 equiv.) and CMP-Neu5Ac (2.0 equiv. per equiv. of terminal Gal) in ddH_2O (final substrate concentration = 1 mM) were sequentially added Tris-HCl buffer (pH = 7.5, 1.0 M, 1/10 *v*/*v* (ddH_2O)), $MgCl_2$ (200 mM in ddH_2O, 1/10 *v*/*v* (ddH_2O)) and α2,3-sialyltransferase *Vibrio sp.* JT-FAJ-16 (mutant, 1% *w*/*w* of substrate per sialylation loci). The reaction mixture was incubated at 37 °C for 24 hrs before it was quenched by adding equal volume of EtOH and leaving on ice for 1 hr. The resulting suspension was centrifuged, and the supernatant was concentrated under vacuum. The residue was purified using P2 (Bio-Gel®, superfine, particle size <45 µm) or Sephadex™ G-25 (Cytiva, superfine, particle size ~20 µm) gel to afford sialylated glycan.

II. **Enzymatic α2,6-Sialylation:** to a solution of terminal Gal *N*-glycan substrate (1.0 equiv.) and CMP-Neu5Ac (2.0 equiv. per equiv. of terminal Gal) in ddH_2O (final substrate concentration = 1 mM) were sequentially added Tris-HCl buffer (pH = 7.5, 1.0 M, 1/10 *v*/*v* (ddH_2O)), $MgCl_2$ (200 mM in ddH_2O, 1/10 *v*/*v* (ddH_2O)) and proper α2,6-sialyltransferase (e.g., Pd2,6ST (A200Y/S232Y) or human ST6Gal1, 1% *w*/*w* of substrate per sialylation loci). The reaction mixture was incubated at 37 °C for 24 hrs before it was quenched by adding equal volume of EtOH and leaving on ice for 1 hr. The resulting suspension was centrifuged, and the supernatant was concentrated under vacuum. The residue was purified using P2 (Bio-Gel®, superfine, particle size <45 µm) or Sephadex™ G-25 (Cytiva, superfine, particle size ~20 µm) gel to afford sialylated glycan. In some cases (especially when using tri-antennary tri-galactoside glycan as an acceptor), the product so isolated was mixed with partially sialylated glycans due to incomplete sialylation reaction. The mixture was subjected to the same reaction conditions to pursue the completion of sialylation.

III. **Enzymatic β1,4-Galactosylation:** to a solution of terminal GlcNAc *N*-glycan substrate (1.0 equiv.) and UDP-Gal disodium salt (2.0 equiv. per equiv. of terminal GlcNAc) in ddH_2O (final substrate concentration = 4-5 mM) were sequentially added Tris-HCl buffer (pH = 7.5, 1.0 M, 1/20 *v*/*v*(ddH_2O)), $MnCl_2$ (200 mM in ddH_2O, 1/20 *v*/*v*(ddH_2O)) and bovine β4GalT1 (0.3 nmol protein per µmol galactosylation). The reaction mixture was incubated at 37 °C for 24 hrs before it was quenched by adding equal volume of EtOH and leaving on ice for 1 hr. The resulting suspension was centrifuged, and the supernatant was concentrated under vacuum. The residue was purified using P2 (Bio-Gel®, superfine, particle size <45 µm) or Sephadex™ G-25 (Cytiva, superfine, particle size ~20 µm) gel to afford galactosylated glycan. It could be further purified on a short DEAE (Bio-Gel®, Cl^- form) column if the product was slightly contaminated with UDP-Gal.

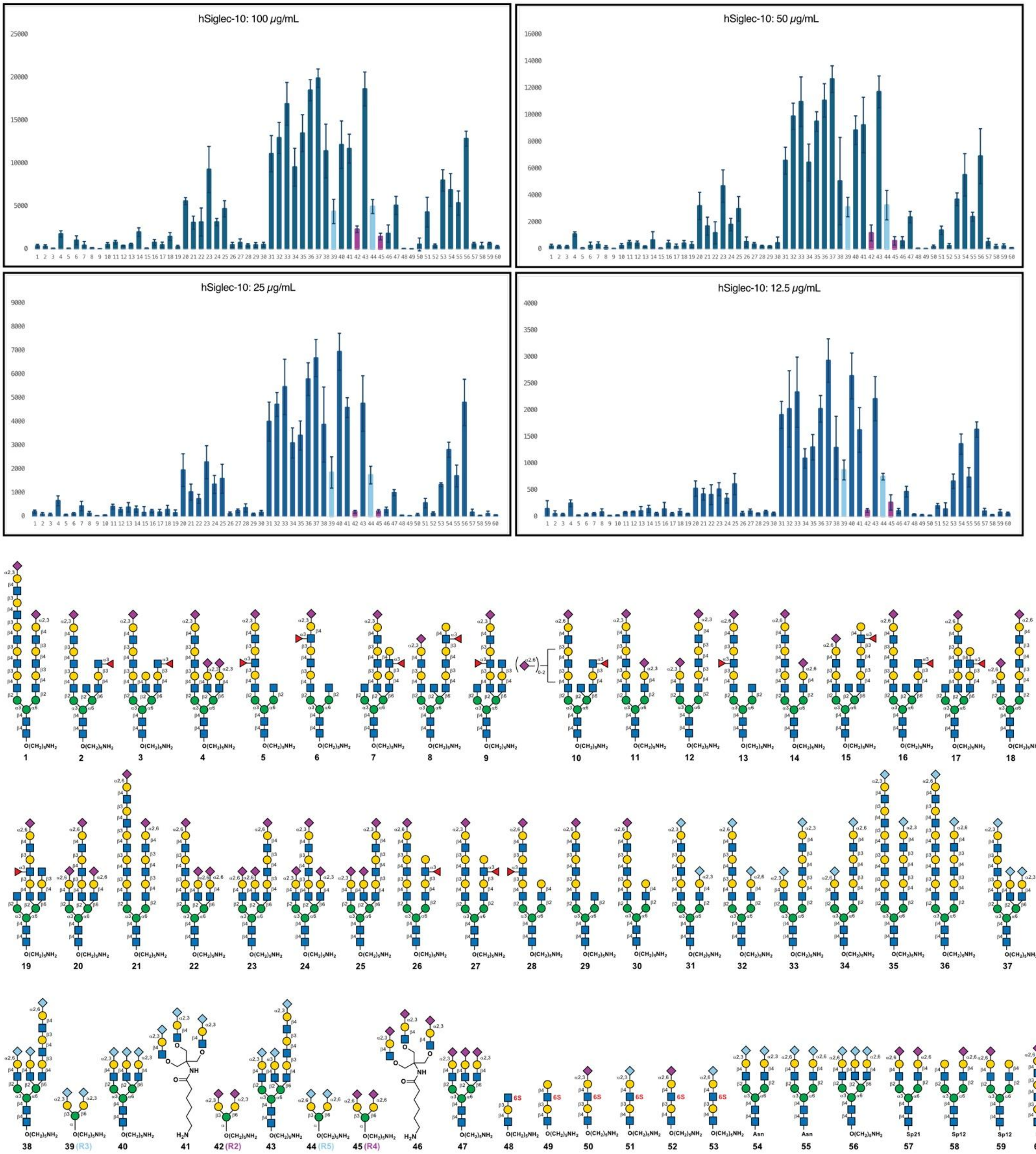

Figure S1. Profiling of human Siglec-10 with *N*-glycan array. Human Siglec-10-Fc (R&D Systems) was precomplexed with mouse anti-human IgG1 Fc antibody with Alexa Fluor[TM] 488. The N-glycan arrays (compounds 1–60) were incubated with the precomplexed proteins at different concentrations (100, 50, 25 or 12.5 μg/mL) for 3 h, and were then washed, dried and scanned at the wavelength of 488 nm.

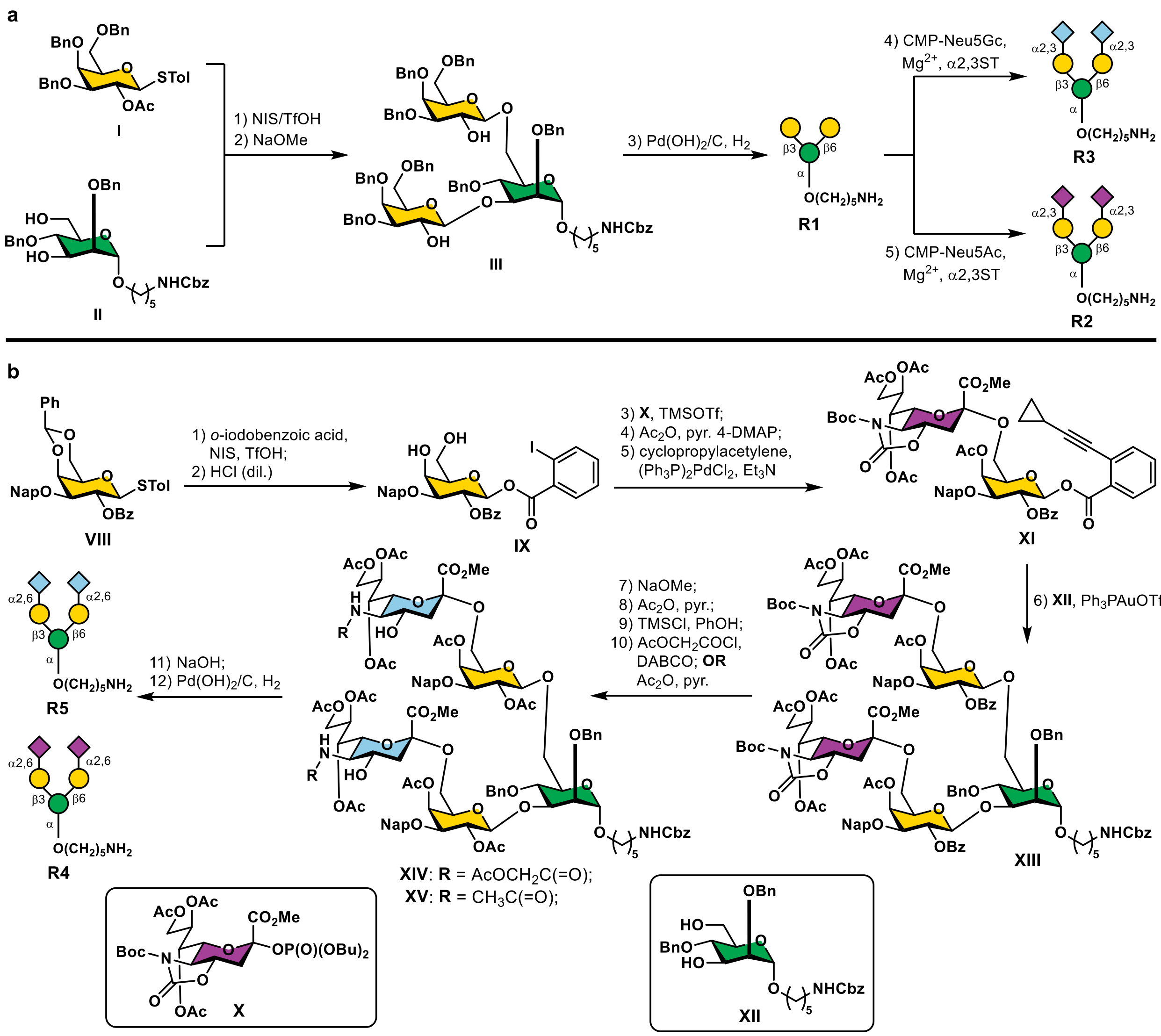

Figure S2. Synthesis of galactoside R1 and its sialylated derivatives R2–R5.
(a) Reagents and conditions: 1) **I** (2.5 equiv.), **II** (1.0 equiv.), NIS (2.5 equiv.), TfOH (0.5 equiv.), 4Å MS, CH_2Cl_2, −40 → 0 °C, 1 h; 2) NaOMe (0.5 equiv.), MeOH, r.t., 24 h, 82% overall; 3) $Pd(OH)_2$/C, H_2 (balloon), THF:H_2O:^{t}BuOH:AcOH (6:3:1:0.1, *v*/*v*/*v*/*v*), 24 h, 82%; 4) CMP-Neu5Gc (2.2 equiv.), $MgCl_2$ (20 mM), Tris·HCl (pH 7.5, 100 mM), α2,3-sialyltransferase (JT-FAJ-16), 89%; 5) CMP-Neu5Ac (2.2 equiv.), $MgCl_2$ (20 mM), Tris·HCl (pH 7.5, 100 mM), α2,3-sialyltransferase (JT-FAJ-16), 95%.
(b) Reagents and conditions: 1) *o*-iodobenzoic acid (2.0 equiv.), NIS (2.0 equiv.), TfOH (0.2 equiv.), 4Å MS, CH_2Cl_2, −40 → 0 °C, 1 h; 2) THF:*i*-PrOH:conc.HCl (20:50:1, *v*/*v*/*v*), 55 °C, 6 h, 73%; 3) **X** (1.1 equiv.), TMSOTf (1.1 equiv.), 4Å MS, CH_2Cl_2, −78 → −25 °C, 1 h; 4) Ac_2O (3.0 equiv.), pyridine (5.0 equiv.), 4-DMAP (0.5 equiv.), CH_2Cl_2, r.t., 24 h, 70% overall; 5) $(Ph_3P)_2PdCl_2$ (0.1 equiv.), CuI (0.2 equiv.), cyclopropylacetylene (5.0 equiv.), Et_3N:DMF (3:1, *v*/*v*), r.t., 24 h, 88%; 6) **XII** (1.0 equiv.), **XI-ABz** (2.5 equiv.), $Ph_3PAuOTf$ (1.0 equiv.), 4Å MS, CH_2Cl_2, 0 °C, 30 min, 52%; 7) NaOMe (10 equiv.), MeOH, r.t., 24 h; 8) Ac_2O:pyridine (1:3, *v*/*v*), r.t., 24 h; 9) TMSCl:PhOH (neat, 1:3, *mol*/*mol*), −78 → −20 °C, 1 h; 10) $AcOCH_2COCl$ (3.0 equiv.), DABCO (3.0 equiv.), CH_2Cl_2, 0 °C → r.t., 12 h; or Ac_2O:pyridine (1:3, *v*/*v*), r.t., 12 h, 48% overall for **XIV**, 52% overall for **XV**; 11) NaOMe:MeOH:H_2O (1:5:10, *v*/*v*/*v*), r.t., 48 h; 12) $Pd(OH)_2$/C, H_2 (balloon), THF:H_2O:^{t}BuOH:AcOH (6:3:1:0.1, *v*/*v*/*v*/*v*), 24 h, 75% overall for **44 (R5)**, 83% overall for **45 (R4)**.

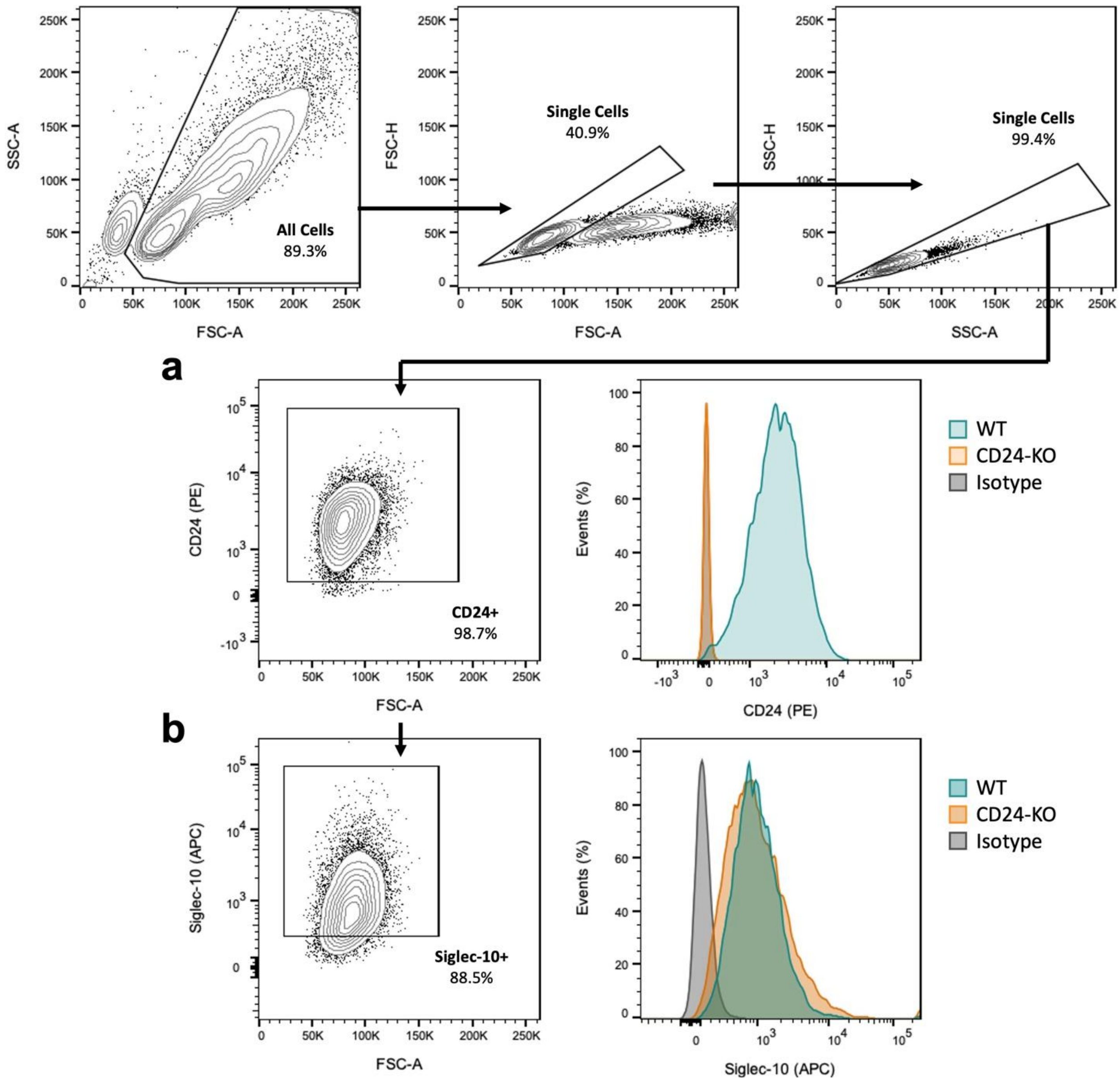

Figure S3. Flow cytometry sample gating for MCF-7. MCF-7 single cells were gated out from debris and doublets. (a) Wild type (WT) and CD24-knockout (CD24-KO) MCF-7 cells were stained with anti-CD24 antibody (anti-CD24 monoclonal antibody, clone SN3 (A5-2H10), PE). CD24 expression was defined from PE-modified isotype control (mouse IgG1, kappa). (b) WT and CD24-KO MCF-7 were incubated with human Siglec-10-Fc, using Alexa Flour® 647-conjugated anti-human IgG antibody as secondary antibody and human IgG1 as isotype control.

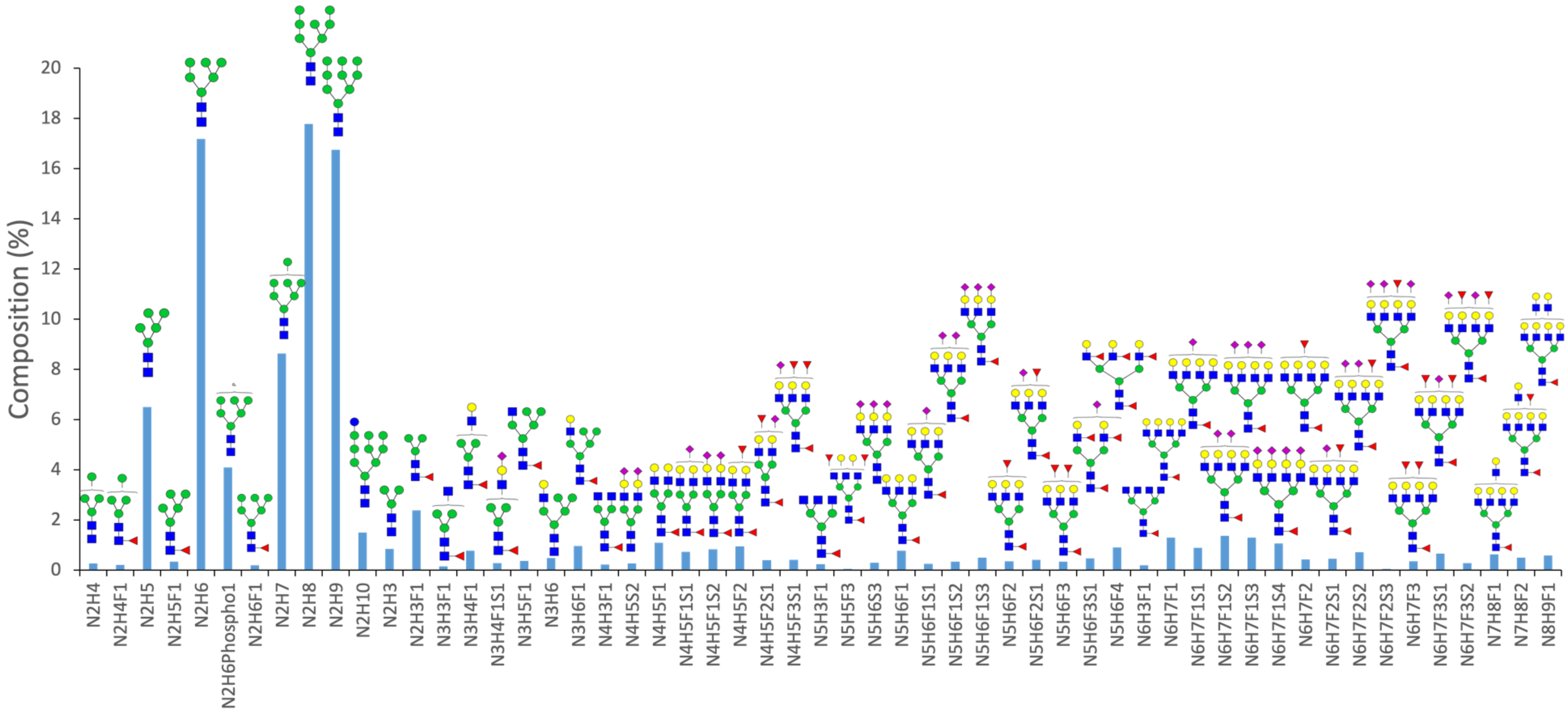

Figure S4. N-glycan profiling of MCF-7 cells.

(Abbreviations: N: HexNAc; H: Hexose; F: Fucose; S: Sialic acid)

Table S1. Neu5Gc percentages in complex-type N-glycans.

	BiF1S2	TriF1S3	TetraF1S4
0 Neu5Gc	97.6%	99.5%	99.5%
1 Neu5Gc	2.4%	0.5%	0.5%
2 Neu5Gc	0	0	0
3 Neu5Gc	0	0	0
4 Neu5Gc	0	0	0

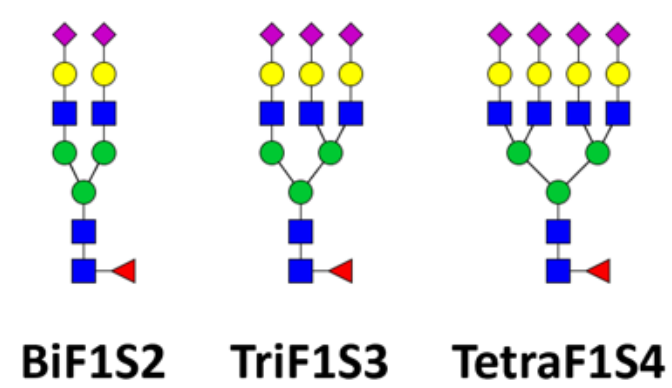

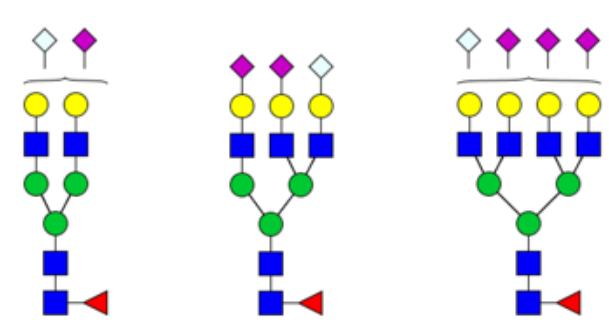

1 Neu5Gc-containing N-glycans

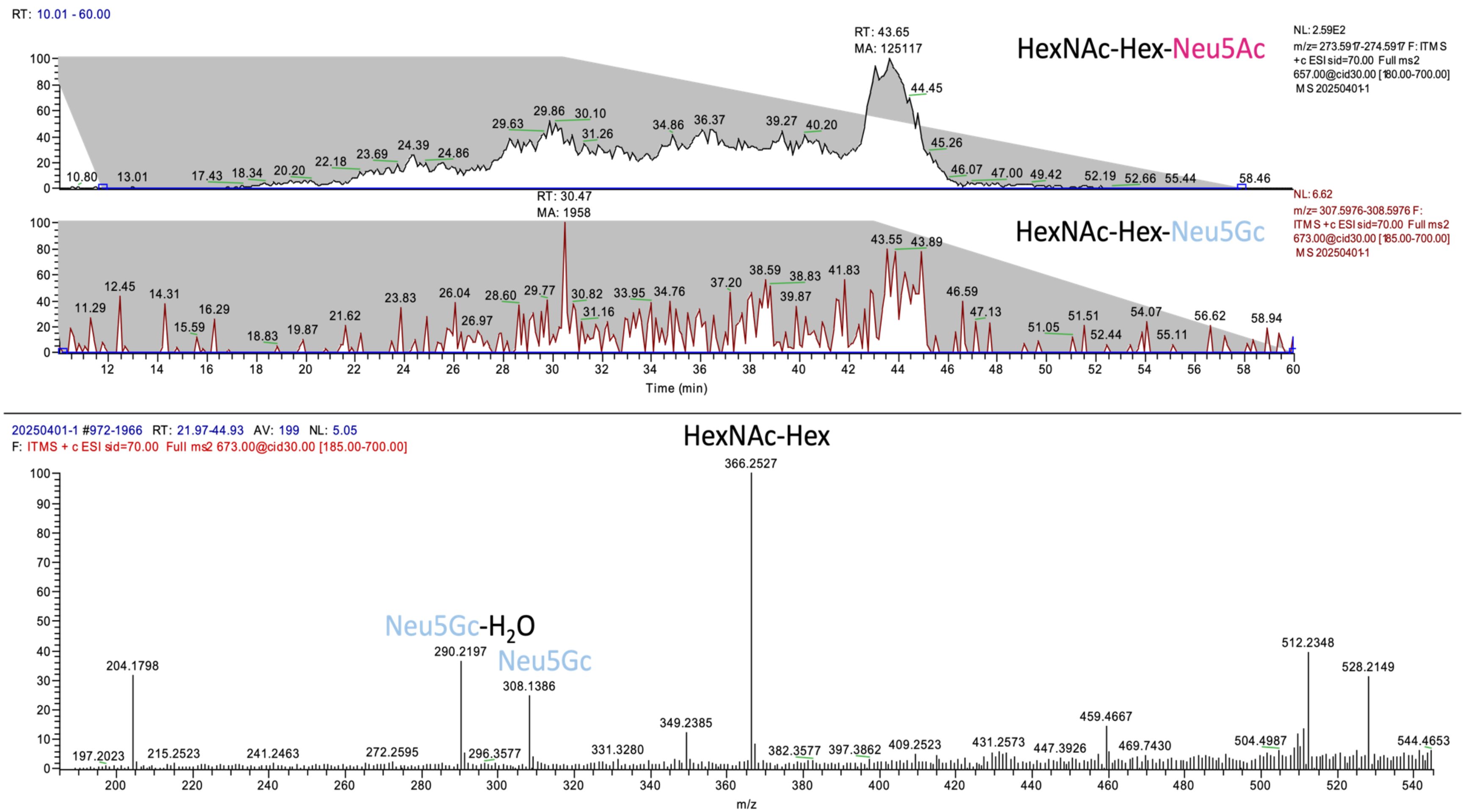

Figure S5. MALDI-ISD spectra of MCF-7 Neu5Ac- and Neu5Gc-containing glycans. WT: wild-type MCF-7.

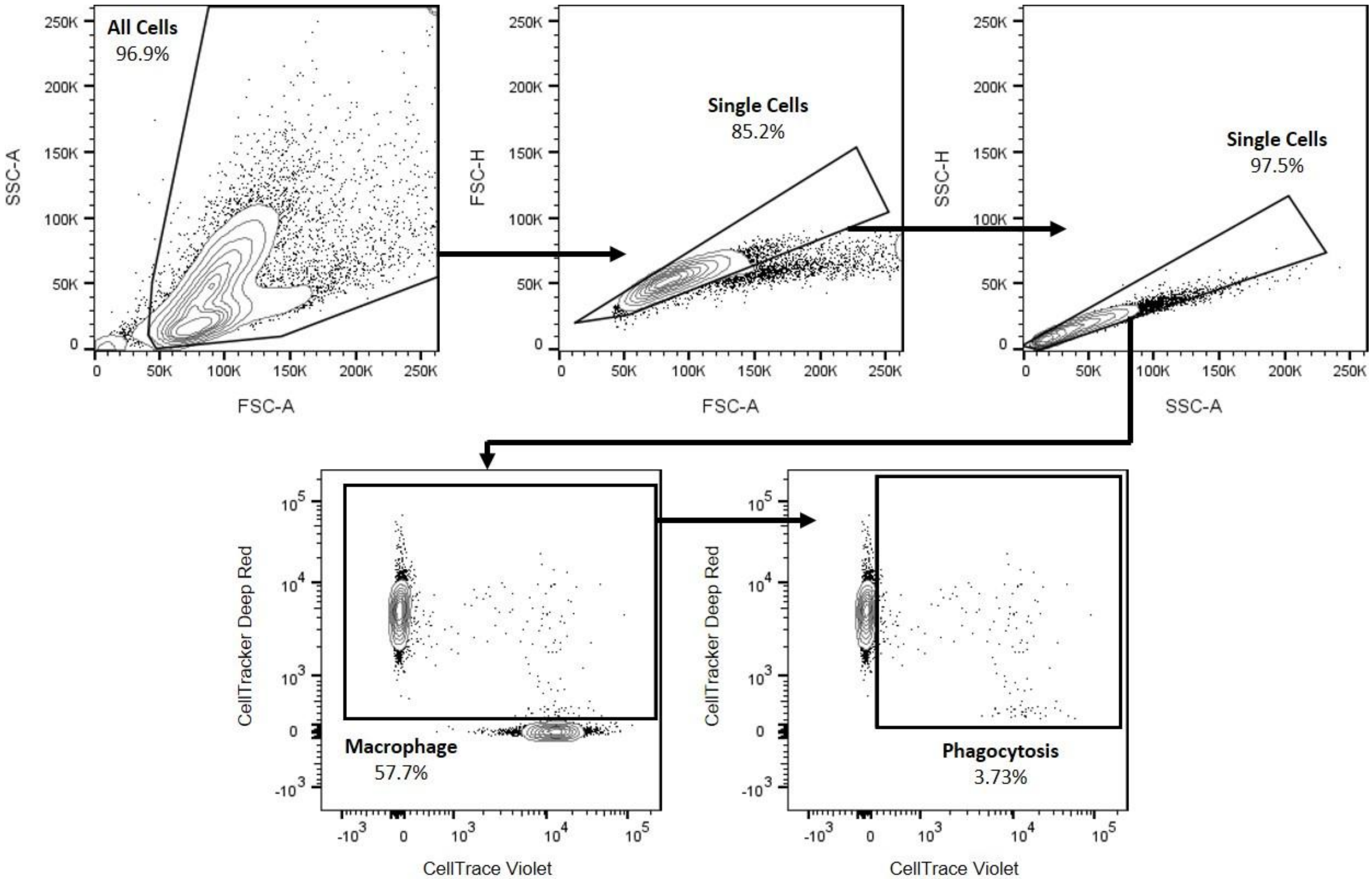

Figure S6. Gating strategy of *in vitro* phagocytosis with flow cytometry. Cell debris and doublets were gated out from single cells. Before co-culturing, macrophages were stained with CellTracker Deep Red, and MCF-7 cells were stained with CellTrace Violet. CellTrace Violet+ signals in macrophages were defined as phagocytosis using PBS as control.

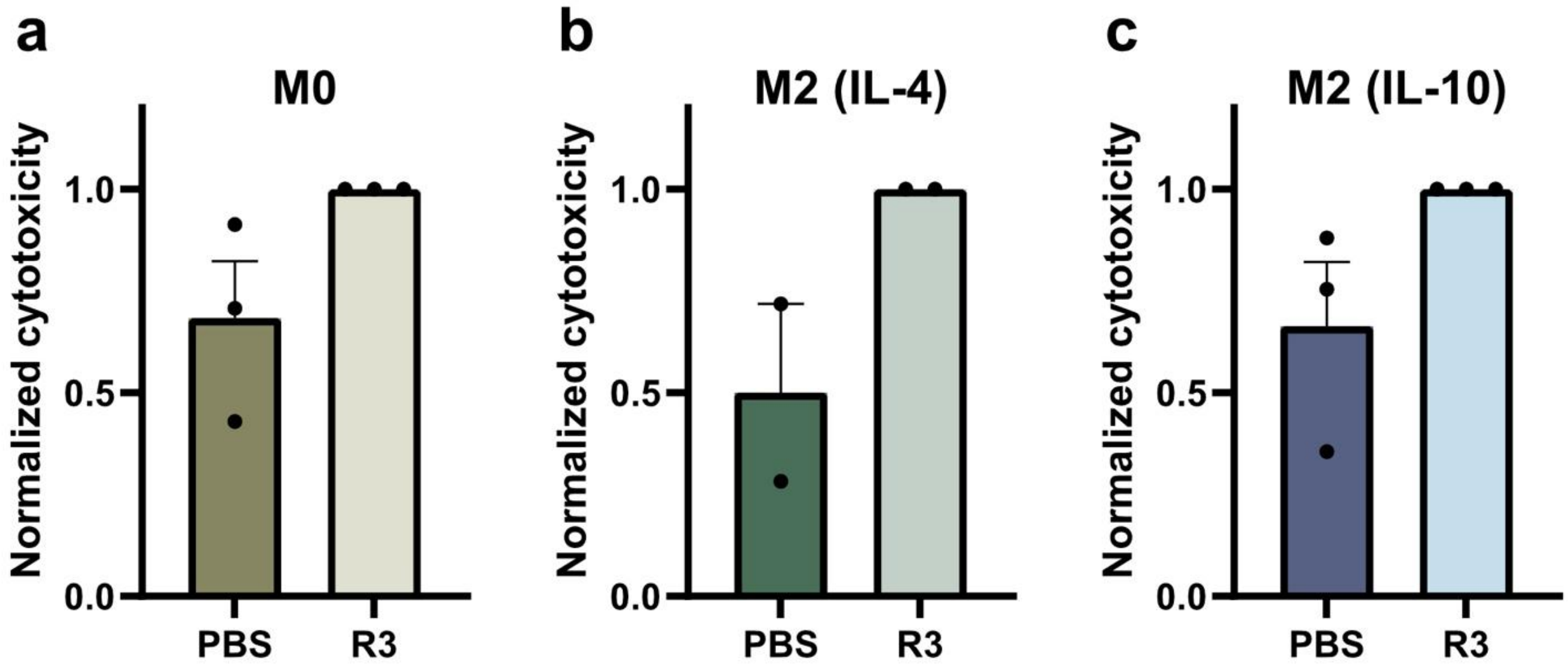

Figure S7. Macrophage cytotoxicity activity enhanced by R3. Enhancement of macrophage cytotoxicity activities in the presence of R3 were found in macrophages: (a) M0-like (n = 3 donors), (b) M2-like (IL-4-induced) (n = 2 donors), and (c) M2-like (IL-10-induced) (n = 3 donors). Data are mean ± SEM.

Table S2. Summary of preparation, ^{1}H NMR and LRMS data of 31–56

Cpd No.	Structure	Parent cpd	General Procedure (Reaction Scale)	Yield (quantity)	^{1}H NMR and LRMS Data
**31**	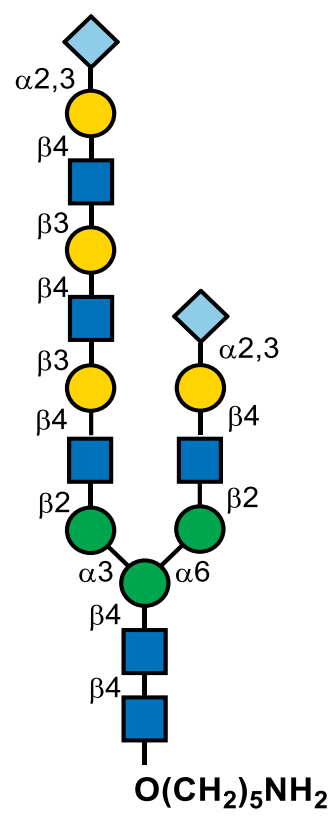 **31**	**Bis-galactoside (synthesis reported in ref.1)**	**I** (0.25 μmol)	80-90% (0.6 mg)	**^{1}H NMR (600 MHz, D_2O)** δ = 5.12 (s, 1H), 4.93 (s, 1H), 4.77 (s, 1H), 4.70 (d, *J* = 8.3 Hz, 2H), 4.63 – 4.52 (m, 5H), 4.52 – 4.42 (m, 3H), 4.25 (s, 1H), 4.19 (br s, 1H), 4.18 – 4.14 (m, 4H), 4.13 (apparent s, 8H), 4.03 – 3.43 (m, 84H), 2.99 (apparent t, *J* = 7.6 Hz, 2H), 2.81 – 2.74 (m, 2H), 2.09 (s, 3H), 2.07 – 2.04 (m, 6H), 2.04 (s, 9H), 1.82 (apparent t, *J* = 12.5 Hz, 2H), 1.67 (quint, *J* = 7.7 Hz, 2H), 1.60 (quint, *J* = 6.6 Hz, 2H), 1.44 – 1.36 (m, 2H) ppm.<br><br>**LRMS (ESI-MS)** *m/z* calculated for $C_{117}H_{193}N_9O_{84}^{2-}$ [M-2H]$^{2-}$: 1534.6, found: 1534.6
**32**	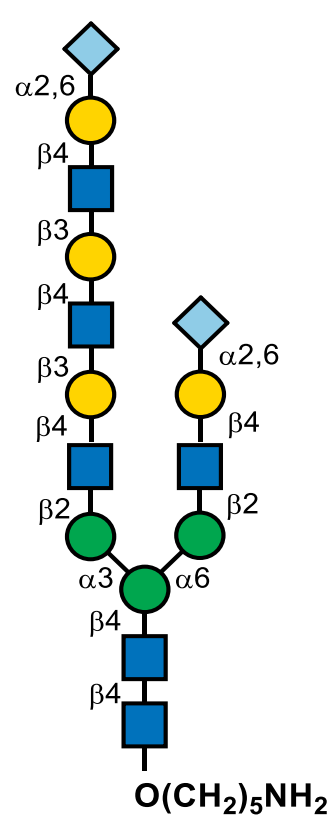 **32**	**Bis-galactoside (synthesis reported in ref.1)**	**II (Pd2,6ST (A200Y/S232Y))** (0.25 μmol)	80-90% (0.6 mg)	**^{1}H NMR (600 MHz, D_2O)** δ = 5.12 (s, 1H), 4.94 (s, 1H), 4.77 (s, 1H), 4.74 (d, *J* = 7.8 Hz, 1H), 4.70 (d, *J* = 8.3 Hz, 1H), 4.63 – 4.56 (m, 3H), 4.51 – 4.43 (m, 5H), 4.25 (br s, 1H), 4.19 (br d, *J* = 2.5 Hz, 1H), 4.18 – 4.14 (m, 3H), 4.12 (apparent s, 5H), 4.05 – 3.47 (m, 88H), 2.99 (apparent t, *J* = 7.6 Hz, 2H), 2.73 – 2.65 (m, 2H), 2.11 – 2.00 (m, 18H), 1.74 (apparent t, *J* = 12.2 Hz, 2H), 1.67 (quint, *J* = 7.7 Hz, 2H), 1.59 (hept, *J* = 6.6 Hz, 2H), 1.44 – 1.36 (m, 2H) ppm.<br><br>**LRMS (ESI-MS)** *m/z* calculated for $C_{117}H_{193}N_9O_{84}^{2-}$ [M-2H]$^{2-}$: 1534.6, found: 1534.7

**33**	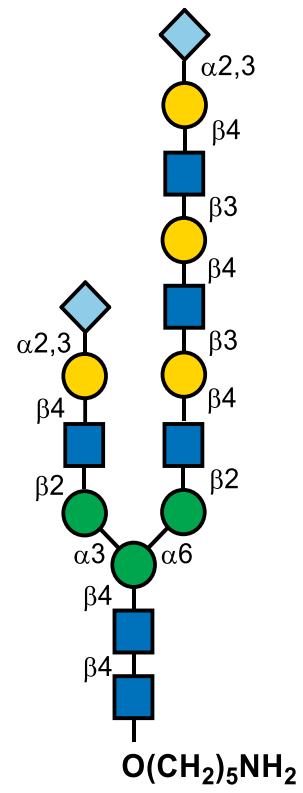 **33**	**Bis-galactoside (synthesis reported in ref.1)**	**I** (0.25 µmol)	80-90% (0.6 mg)	**^{1}H NMR (600 MHz, D_2O)** δ = 5.12 (s, 1H), 4.93 (s, 1H), 4.78 (s, 1H), 4.70 (d, *J* = 8.2 Hz, 2H), 4.63 – 4.53 (m, 5H), 4.52 – 4.44 (m, 3H), 4.25 (br s, 1H), 4.20 (br d, *J* = 2.6 Hz, 1H), 4.16 (br d, *J* = 3.1 Hz, 2H), 4.15 – 4.10 (m, 8H), 4.03 – 3.46 (m, 86H), 2.99 (apparent t, *J* = 7.5 Hz, 2H), 2.81 – 2.74 (m, 2H), 2.09 (s, 3H), 2.07 – 2.01 (m, 15H), 1.82 (apparent t, *J* = 12.1 Hz, 2H), 1.67 (apparent quint, *J* = 7.6 Hz, 2H), 1.60 (apparent quint, *J* = 6.6 Hz, 2H), 1.44 – 1.36 (m, 2H) ppm.<br><br>**LRMS (ESI-MS)** *m/z* calculated for $C_{117}H_{193}N_9O_{84}^{2-}$ $[M-2H]^{2-}$: 1534.6, found: 1534.6
**34**	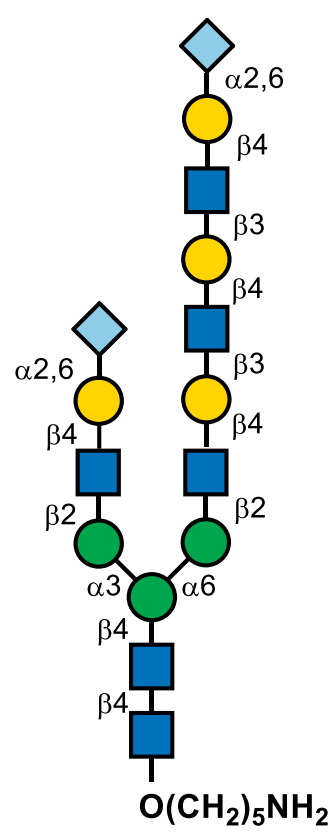 **34**	**Bis-galactoside (synthesis reported in ref.1)**	**II (Pd2,6ST (A200Y/S232Y))** (0.25 µmol)	80-90% (0.6 mg)	**^{1}H NMR (600 MHz, D_2O)** δ = 5.13 (s, 1H), 4.93 (s, 1H), 4.78 (s, 1H), 4.74 (d, *J* = 7.5 Hz, 1H), 4.71 (d, *J* = 8.3 Hz, 1H), 4.64 – 4.56 (m, 3H), 4.52 – 4.44 (m, 5H), 4.26 (br s, 1H), 4.20 (br d, *J* = 2.8 Hz, 1H), 4.17 (d, *J* = 3.2 Hz, 2H), 4.14 – 4.09 (m, 5H), 4.05 – 3.46 (m, 89H), 2.97 (apparent t, *J* = 7.7 Hz, 2H), 2.72 – 2.65 (m, 2H), 2.10 – 2.01 (m, 18H), 1.74 (apparent t, *J* = 12.2 Hz, 2H), 1.66 (apparent quint, *J* = 7.6 Hz, 2H), 1.59 (apparent quint, *J* = 6.6 Hz, 2H), 1.44 – 1.35 (m, 2H) ppm.<br><br>**LRMS (ESI-MS)** *m/z* calculated for $C_{117}H_{193}N_9O_{84}^{2-}$ $[M-2H]^{2-}$: 1534.6, found: 1534.7
**35**	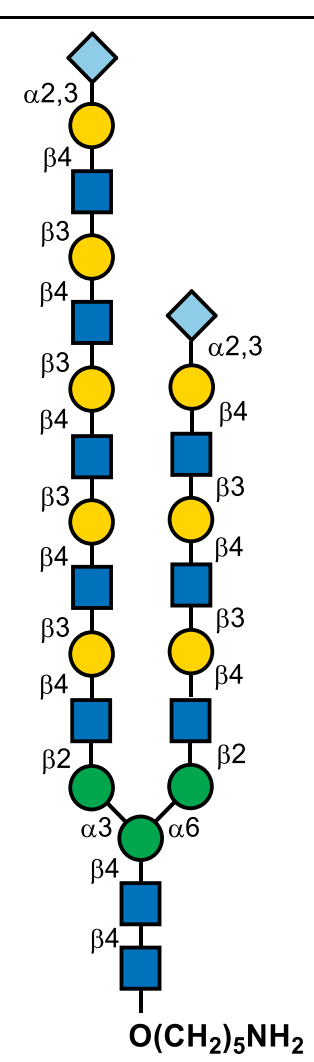 **35**	**Bis-galactoside (synthesis reported in ref.1)**	**I** (0.25 µmol)	80-90% (1.0 mg)	**^{1}H NMR (600 MHz, D_2O)** δ = 5.12 (s, 1H), 4.93 (s, 1H), 4.78 (s, 1H), 4.70 (d, *J* = 8.3 Hz, 5H), 4.62 – 4.53 (m, 5H), 4.51 – 4.42 (m, 8H), 4.25 (br s, 1H), 4.21 – 4.11 (m, 14H), 4.04 – 3.42 (m, 131H), 2.99 (apparent t, *J* = 7.6 Hz, 2H), 2.81 – 2.75 (m, 2H), 2.11 – 1.98 (m, 30H), 1.82 (apparent t, *J* = 12.1 Hz, 2H), 1.67 (apparent quint, *J* = 7.7 Hz, 2H), 1.59 (apparent quint, *J* = 6.6 Hz, 2H), 1.43 – 1.36 (m, 2H) ppm.<br><br>**LRMS (ESI-MS)** *m/z* calculated for $C_{173}H_{284}N_{13}O_{124}^{3-}$ $[M-3H]^{3-}$: 1509.5, found: 1509.4

**36**	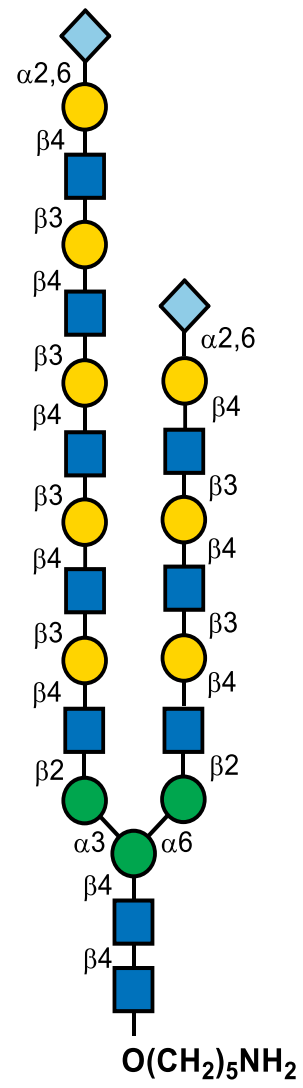 **36**	**Bis-galactoside (synthesis reported in ref.1)**	**II (Pd2,6ST (A200Y/S232Y))** (0.25 μmol)	80-90% (1.0 mg)	**^{1}H NMR (600 MHz, D$_2$O)** δ = 5.13 (s, 1H), 4.93 (s, 1H), 4.78 (s, 1H), 4.74 (d, *J* = 7.7 Hz, 4H), 4.70 (d, *J* = 8.5 Hz, 4H), 4.61 (d, *J* = 7.6 Hz, 1H), 4.57 (d, *J* = 8.0 Hz, 1H), 4.51 – 4.42 (m, 8H), 4.25 (br s, 1H), 4.19 (br s, 1H), 4.18 – 4.14 (m, 5H), 4.14 – 4.08 (m, 5H), 4.04 – 3.43 (m, 134H), 2.99 (apparent t, *J* = 7.6 Hz, 2H), 2.72 – 2.63 (m, 2H), 2.10 – 1.99 (m, 30H), 1.74 (apparent t, *J* = 12.2 Hz, 2H), 1.67 (apparent quint, *J* = 7.8 Hz, 2H), 1.63 – 1.55 (m, 2H), 1.44 – 1.35 (m, 2H) ppm.<br><br>**LRMS (ESI-MS)** *m/z* calculated for $C_{173}H_{284}N_{13}O_{124}^{3-}$ [M-3H]$^{3-}$: 1509.5, found:1509.5
**37**	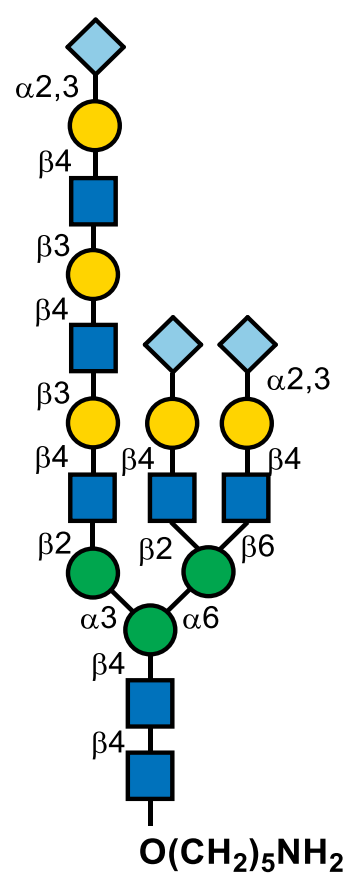 **37**	**Tris-galactoside (synthesis reported in ref.1)**	**I** (0.25 μmol)	80-90% (0.7 mg)	**^{1}H NMR (600 MHz, D$_2$O)** δ = 5.13 (s, 1H), 4.88 (s, 1H), 4.78 (s, 1H), 4.70 (d, *J* = 8.4 Hz, 2H), 4.64 – 4.52 (m, 7H), 4.52 – 4.43 (m, 3H), 4.29 – 4.07 (m, 15H), 4.04 – 3.36 (m, 106H), 2.99 (apparent t, *J* = 7.7 Hz, 2H), 2.82 – 2.73 (m, 2H), 2.11 – 1.99 (m, 21H), 1.82 (apparent t, *J* = 12.7 Hz, 2H), 1.67 (apparent quint, *J* = 7.8 Hz, 2H), 1.59 (apparent quint, *J* = 6.5 Hz, 2H), 1.47 – 1.34 (m, 2H) ppm.<br><br>**LRMS (ESI-MS)** *m/z* calculated for $C_{142}H_{232}N_{11}O_{103}^{3-}$ [M-3H]$^{3-}$: 1246.8, found: 1246.7
**38**	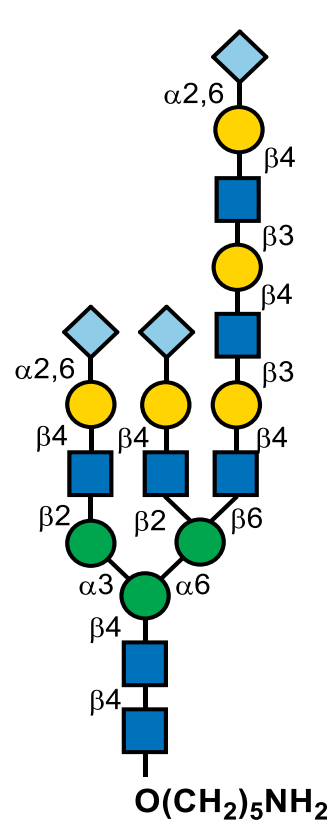 **38**	**Tris-galactoside (synthesis reported in ref.1)**	**II (Pd2,6ST (A200Y/S232Y))** (0.25 μmol)	60-70% (0.6 mg)	**^{1}H NMR (600 MHz, D$_2$O)** δ = 5.11 (s, 1H), 4.88 (s, 1H), 4.79 (s, 1H), 4.75 (d, *J* = 6.8 Hz, 2H), 4.70 (d, *J* = 6.9 Hz, 1H), 4.67 (d, *J* = 8.2 Hz, 1H), 4.62 – 4.54 (m, 2H), 4.52 (d, *J* = 8.0 Hz, 1H), 4.48 – 4.39 (m, 5H), 4.23 (s, 1H), 4.22 – 4.16 (m, 2H), 4.15 – 4.12 (m, 2H), 4.12 – 4.04 (m, 8H), 4.01 – 3.34 (m, 106H), 2.97 (apparent t, *J* = 7.6 Hz, 2H), 2.66 (dd, *J* = 12.1, 4.6 Hz, 3H), 2.09 – 1.96 (m, 21H), 1.71 (apparent t, *J* = 12.1 Hz, 3H), 1.64 (apparent quint, *J* = 7.5 Hz, 2H), 1.56 (apparent quint, *J* = 7.3 Hz, 2H), 1.42 – 1.32 (m, 2H) ppm.<br><br>**LRMS (ESI-MS)** *m/z* calculated for $C_{142}H_{232}N_{11}O_{103}^{3-}$ [M-3H]$^{3-}$: 1246.8, found: 1246.7

**39**	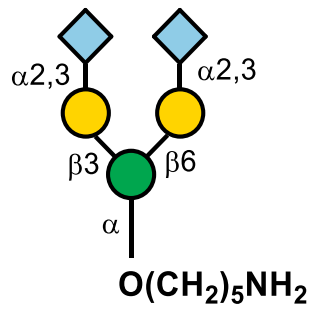 **39 (R3)**	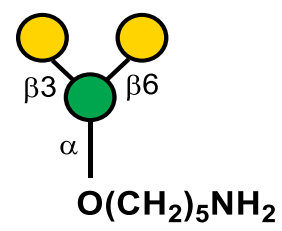 **A (R1)**	**I** (5.0 µmol)	89% (5.5 mg)	**^{1}H NMR (600 MHz, D$_2$O)** δ = 4.92 (d, *J* = 1.8 Hz, 1H), 4.61 (d, *J* = 7.8 Hz, 1H), 4.54 (d, *J* = 7.9 Hz, 1H), 4.17 (dd, *J* = 3.1, 1.9 Hz, 1H), 4.16 – 4.10 (m, 6H), 4.06 – 4.03 (m, 1H), 3.99 – 3.54 (m, 30H), 3.02 (apparent t, *J* = 7.6 Hz, 2H), 2.79 (apparent dt, *J* = 12.4, 4.4 Hz, 2H), 1.83 (apparent t, *J* = 12.1 Hz, 2H), 1.75 – 1.61 (m, 4H), 1.47 (apparent quint, *J* = 8.4 Hz, 2H) ppm. **LRMS (ESI-MS)** *m/z* calculated for $C_{45}H_{75}N_3O_{34}^{2-}$ [M-2H]$^{2-}$: 600.7, found: 600.8
**40**	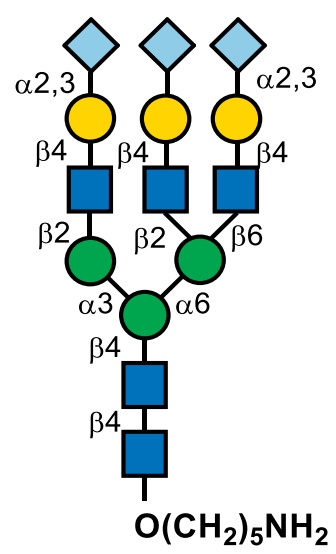 **40**	**Tris-galactoside (synthesis reported in ref.2, but NMR data were erroneously reported in ref.2)**	**I** (0.5 µmol)	80-90% (1.2 mg)	**^{1}H NMR (600 MHz, D$_2$O)** δ = 5.10 (s, 1H), 4.85 (s, 1H), 4.74 (s, 1H), 4.61 – 4.49 (m, 7H), 4.46 (d, *J* = 7.7 Hz, 1H), 4.25 – 4.14 (m, 3H), 4.14 – 4.05 (m, 10H), 4.01 – 3.33 (m, 62H), 2.96 (apparent t, *J* = 7.6 Hz, 2H), 2.75 (dd, *J* = 12.2, 4.2 Hz, 3H), 2.08 – 1.97 (m, 15H), 1.79 (apparent t, *J* = 12.1 Hz, 3H), 1.64 (apparent quint, *J* = 7.8 Hz, 2H), 1.59 – 1.52 (m, 2H), 1.40 – 1.32 (m, 2H) ppm. **LRMS (ESI-MS)** *m/z* calculated for $C_{114}H_{186}N_9O_{83}^{3-}$ [M-3H]$^{3-}$: 1003.4, found: 1003.4
**41**	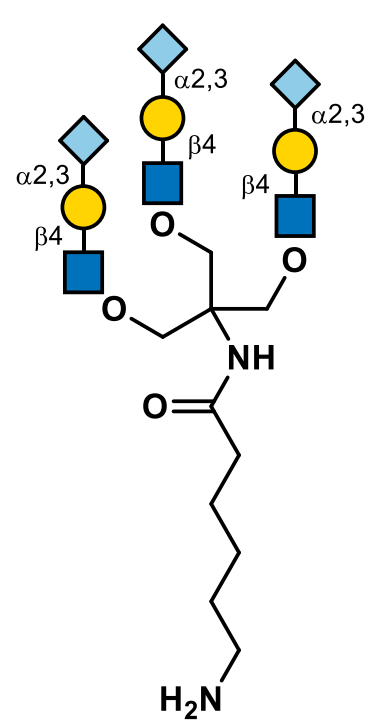 **41**	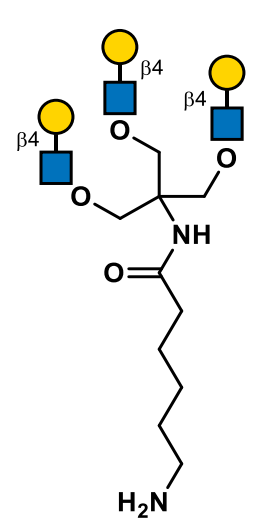 **B**	**I** (0.5 µmol)	95-100% (1.1 mg)	**^{1}H NMR (600 MHz, D$_2$O)** δ = 4.53 (d, *J* = 7.8 Hz, 3H), 4.38 (d, *J* = 6.9 Hz, 3H), 4.13 – 4.04 (m, 12H), 3.99 – 3.80 (m, 18H), 3.80 – 3.66 (m, 24H), 3.65 – 3.59 (m, 6H), 3.58 – 3.51 (m, 9H), 2.90 – 2.83 (m, 2H), 2.75 (dd, *J* = 12.4, 4.6 Hz, 3H), 2.28 – 2.13 (m, 2H), 2.03 (s, 9H), 1.79 (apparent t, *J* = 12.1 Hz, 3H), 1.63 – 1.51 (m, 4H), 1.35 (apparent quint, *J* = 7.7 Hz, 2H) ppm. **LRMS (ESI-MS)** *m/z* calculated for $C_{85}H_{140}N_8O_{61}^{2-}$ [M-2H]$^{2-}$: 1124.4, found: 1124.4
**42**	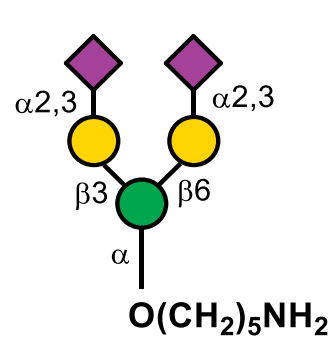 **42 (R2)**	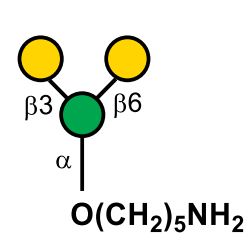 **A**	**I** (5.0 µmol)	95% (5.6 mg)	**^{1}H NMR (600 MHz, D$_2$O)** δ = 4.89 (s, 1H), 4.57 (d, *J* = 7.8 Hz, 1H), 4.51 (d, *J* = 7.9 Hz, 1H), 4.20 (d, *J* = 11.1 Hz, 1H), 4.13 (br t, *J* = 2.7 Hz, 1H), 4.11 – 4.07 (m, 2H), 4.03 – 3.98 (m, 1H), 3.93 (apparent t, *J* = 3.2 Hz, 2H), 3.89 – 3.80 (m, 9H), 3.78 – 3.51 (m, 18H), 2.98 (apparent t, *J* = 7.6 Hz, 2H), 2.74 (apparent dt, *J* = 12.5,

					4.8 Hz, 2H), 2.01 (s, 6H), 1.78 (apparent t, *J* = 12.1 Hz, 2H), 1.72 – 1.58 (m, 4H), 1.49 – 1.39 (m, 2H) ppm.<br><br>**LRMS (ESI-MS)** *m/z* calculated for $C_{45}H_{75}N_3O_{32}^{2-}$ $[M-2H]^{2-}$: 584.7, found: 584.7
**43**	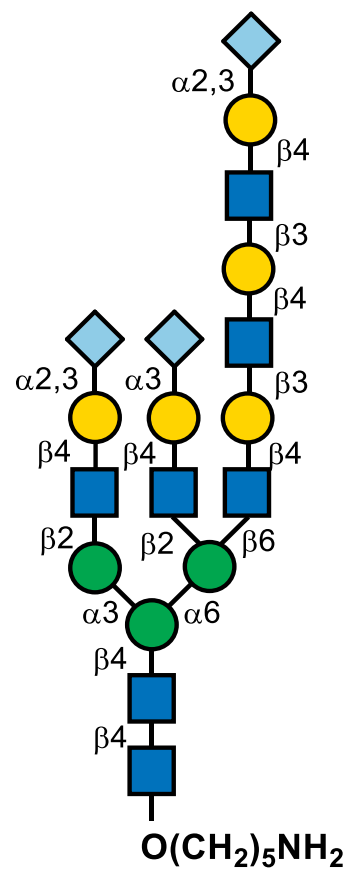 **43**	**Tris-galactoside (synthesis reported in ref.1)**	**I** (0.25 µmol)	80-90% (0.8 mg)	**^{1}H NMR (600 MHz, D_2O)** δ = 5.10 (s, 1H), 4.84 (s, 1H), 4.74 (s, 1H), 4.67 (d, *J* = 8.4 Hz, 2H), 4.61 – 4.48 (m, 7H), 4.49 – 4.40 (m, 3H), 4.24 – 4.03 (m, 13H), 4.01 – 3.34 (m, 106H), 2.95 (apparent t, *J* = 7.7 Hz, 2H), 2.75 (dd, *J* = 12.3, 4.6 Hz, 3H), 2.08 – 1.96 (m, 21H), 1.79 (apparent t, *J* = 12.1 Hz, 3H), 1.69 – 1.60 (m, 2H), 1.59 – 1.49 (m, 2H), 1.43 – 1.31 (m, 2H) ppm.<br><br>**LRMS (ESI-MS)** *m/z* calculated for $C_{142}H_{232}N_{11}O_{103}^{3-}$ $[M-3H]^{3-}$: 1246.8, found: 1246.8
**44**	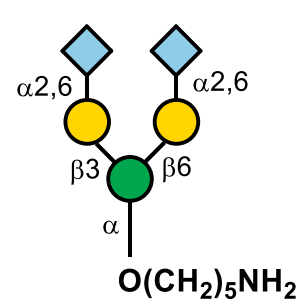 **44 (R5)**	**N.A.**	**N.A. (See Scheme S3 for synthetic details)**	**N.A.**	**^{1}H NMR (600 MHz, D_2O)** δ = 4.89 (s, 1H), 4.47 (d, *J* = 7.8 Hz, 1H), 4.44 (d, *J* = 7.9 Hz, 1H), 4.19 (d, *J* = 11.2 Hz, 1H), 4.15 – 4.07 (m, 5H), 4.03 – 3.72 (m, 21H), 3.68 – 3.49 (m, 11H), 3.00 (apparent t, *J* = 7.6 Hz, 2H), 2.72 (apparent dt, *J* = 12.4, 5.1 Hz, 2H), 1.78 – 1.59 (m, 6H), 1.52 – 1.39 (m, 2H) ppm.<br><br>**LRMS (ESI-MS)** *m/z* calculated for $C_{45}H_{75}N_3O_{34}^{2-}$ $[M-2H]^{2-}$: 600.7, found: 600.7
**45**	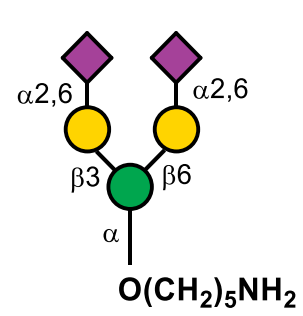 **45 (R4)**	**N.A.**	**N.A. (See Scheme S3 for synthetic details)**	**N.A.**	**^{1}H NMR (600 MHz, D_2O)** δ = 4.92 (s, 1H), 4.51 (d, *J* = 7.8 Hz, 1H), 4.47 (d, *J* = 7.9 Hz, 1H), 4.22 (dd, *J* = 11.5, 2.1 Hz, 1H), 4.14 (dd, *J* = 3.2, 1.8 Hz, 1H), 4.04 – 3.75 (m, 17H), 3.75 – 3.52 (m, 15H), 3.03 (apparent t, *J* = 7.6 Hz, 2H), 2.74 (apparent dt, *J* = 12.3, 4.8 Hz, 2H), 2.04 (s, 6H), 1.79 – 1.63 (m, 6H), 1.56 – 1.42 (m, 2H) ppm.<br><br>**LRMS (ESI-MS)** *m/z* calculated for $C_{45}H_{75}N_3O_{32}^{2-}$ $[M-2H]^{2-}$: 584.7, found: 584.7

**46**	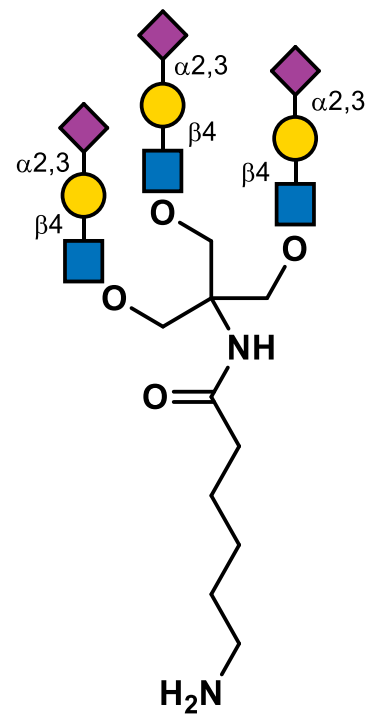 **46**	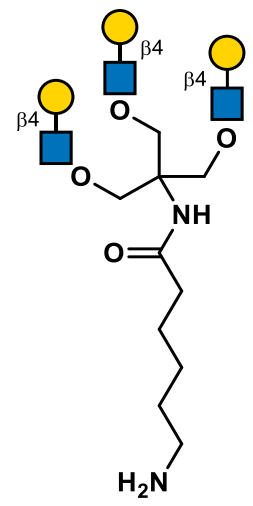 **B**	**I** (0.5 μmol)	90-100% (1.1 mg)	**^{1}H NMR (600 MHz, D$_2$O)** δ = 4.57 (d, J = 7.8 Hz, 3H), 4.42 (d, J = 6.3 Hz, 3H), 4.13 (dd, J = 9.6, 3.7 Hz, 6H), 4.05 – 3.95 (m, 6H), 3.93 – 3.82 (m, 12H), 3.80 – 3.52 (m, 39H), 3.02 (apparent t, J = 7.7 Hz, 2H), 2.77 (dd, J = 12.5, 4.7 Hz, 3H), 2.36 – 2.17 (m, 2H), 2.06 (s, 9H), 2.04 (s, 9H), 1.81 (apparent t, J = 12.1 Hz, 3H), 1.74 – 1.66 (m, 2H), 1.65 – 1.55 (m, 2H), 1.47 – 1.36 (m, 2H) ppm.<br><br>**LRMS (ESI-MS)** *m/z* calculated for $C_{85}H_{140}N_8O_{58}^{2-}$ [M-2H]$^{2-}$: 1100.4, found: 1100.5
**47**	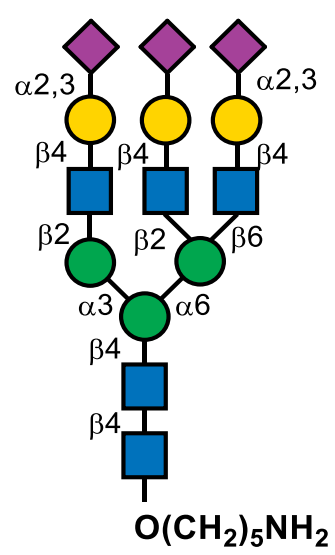 **47**	**Tris-galactoside (synthesis reported in ref.2, but NMR data were erroneously reported in ref.2)**	**I** (0.25 μmol)	90% (0.6 mg)	**^{1}H NMR (600 MHz, D$_2$O)** δ = 5.10 (s, 1H), 4.85 (s, 1H), 4.74 (s, 1H), 4.61 – 4.49 (m, 7H), 4.46 (d, J = 7.8 Hz, 1H), 4.26 – 4.14 (m, 3H), 4.14 – 4.03 (m, 4H), 4.02 – 3.32 (m, 82H), 2.96 (apparent t, J = 7.7 Hz, 2H), 2.73 (dd, J = 12.4, 4.6 Hz, 3H), 2.08 – 1.96 (m, 24H), 1.78 (apparent t, J = 12.0 Hz, 3H), 1.64 (apparent quint, J = 7.8 Hz, 2H), 1.57 (apparent quint, J = 7.2 Hz, 2H), 1.45 – 1.32 (m, 2H) ppm.<br><br>**LRMS (ESI-MS)** *m/z* calculated for $C_{114}H_{186}N_9O_{80}^{3-}$ [M-3H]$^{3-}$: 987.4, found: 987.4
**48**	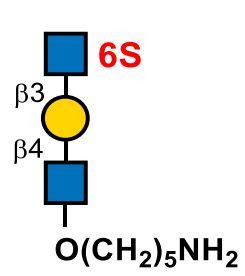 **48**	**N.A.**	**N.A. (See Scheme S4 for synthetic details)**	**N.A.**	**^{1}H NMR (600 MHz, D$_2$O)** δ = 4.70 (d, J = 8.5 Hz, 1H), 4.53 (d, J = 7.5 Hz, 1H), 4.47 (d, J = 7.9 Hz, 1H), 4.34 (dd, J = 11.2, 2.1 Hz, 1H), 4.23 (dd, J = 11.2, 5.8 Hz, 1H), 4.20 (d, J = 3.3 Hz, 1H), 4.00 (dd, J = 12.3, 2.2 Hz, 1H), 3.92 (apparent dt, J = 10.1, 6.0 Hz, 1H), 3.86 – 3.65 (m, 10H), 3.64 – 3.56 (m, 4H), 3.52 (apparent t, J = 9.4 Hz, 1H), 3.00 (apparent t, J = 7.5 Hz, 2H), 2.05 (s, 3H), 2.04 (s, 3H), 1.68 (apparent quint, J = 7.7 Hz, 2H), 1.61 (apparent quint, J = 6.5 Hz, 2H), 1.44 – 1.37 (m, 2H) ppm.<br><br>**LRMS (ESI-MS)** *m/z* calculated for $C_{27}H_{48}N_3O_{19}S^-$ [M-H]$^-$: 750.3, found: 750.3
**49**	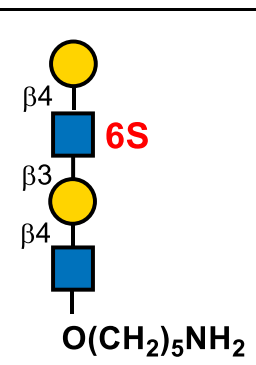 **49**	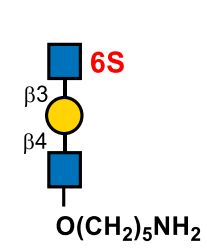 **48**	**III** (4.0 μmol)	85% (3.1 mg)	**^{1}H NMR (600 MHz, D$_2$O)** δ = 4.73 (d, J = 8.4 Hz, 1H), 4.53 (d, J = 7.8 Hz, 2H), 4.47 (d, J = 7.9 Hz, 1H), 4.44 – 4.39 (m, 1H), 4.32 (dd, J = 11.0, 4.0 Hz, 1H), 4.20 (d, J = 3.3 Hz, 1H), 4.00 (dd, J = 12.3, 2.2 Hz, 1H), 3.95 – 3.88 (m, 2H), 3.87 – 3.66 (m, 16H),

					3.65 – 3.56 (m, 3H), 3.54 (dd, J = 10.0, 7.8 Hz, 1H), 3.00 (apparent t, J = 7.6 Hz, 2H), 2.05 (s, 3H), 2.04 (s, 3H), 1.68 (apparent quint, J = 7.7 Hz, 2H), 1.61 (apparent quint, J = 6.5 Hz, 2H), 1.47 – 1.37 (m, 2H) ppm.<br><br>**LRMS (ESI-MS)** *m/z* calculated for $C_{33}H_{58}N_3O_{24}S^-$ [M-H]$^-$: 912.3, found: 912.3
**50**	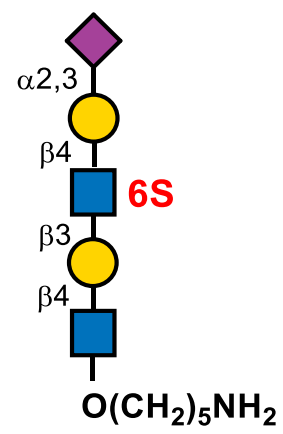<br>**50**	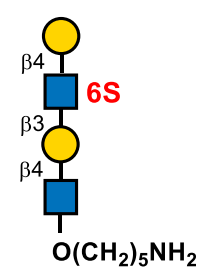<br>**49**	**I**<br>(1.0 μmol)	83%<br>(1.0 mg)	**^{1}H NMR (600 MHz, D$_2$O)** δ = 4.68 (d, J = 8.4 Hz, 1H), 4.57 (d, J = 7.9 Hz, 1H), 4.49 (d, J = 7.5 Hz, 1H), 4.44 (d, J = 7.8 Hz, 1H), 4.38 (d, J = 10.8 Hz, 1H), 4.29 (d, J = 10.7 Hz, 1H), 4.17 (d, J = 3.3 Hz, 1H), 4.10 (dd, J = 9.8, 3.1 Hz, 1H), 3.98 – 3.92 (m, 2H), 3.92 – 3.50 (m, 27H), 2.96 (apparent t, J = 7.4 Hz, 2H), 2.72 (dd, J = 12.4, 4.6 Hz, 1H), 2.01 (s, 9H), 1.78 (apparent t, J = 12.1 Hz, 1H), 1.64 (apparent quint, J = 7.7 Hz, 2H), 1.57 (apparent quint, J = 6.8 Hz, 2H), 1.43 – 1.33 (m, 2H) ppm.<br><br>**LRMS (ESI-MS)** *m/z* calculated for $C_{44}H_{75}N_4O_{32}S^-$ [M-H]$^-$: 1203.4, found: 1203.4
**51**	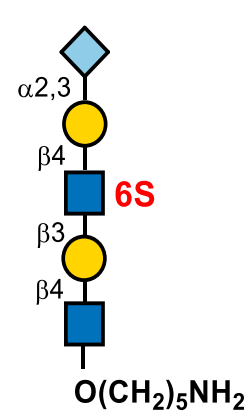<br>**51**	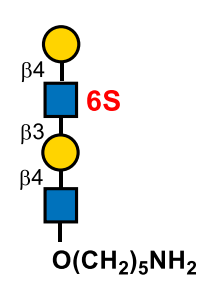<br>**49**	**I**<br>(1.0 μmol)	82%<br>(1.0 mg)	**^{1}H NMR (600 MHz, D$_2$O)** δ = 4.68 (d, J = 8.4 Hz, 1H), 4.57 (d, J = 7.8 Hz, 1H), 4.49 (d, J = 7.5 Hz, 1H), 4.44 (d, J = 7.8 Hz, 1H), 4.38 (d, J = 10.9 Hz, 1H), 4.29 (br d, J = 10.8 Hz, 1H), 4.17 (d, J = 3.2 Hz, 1H), 4.12 – 4.07 (m, 3H), 3.99 – 3.51 (m, 29H), 2.94 (apparent t, J = 7.6 Hz, 2H), 2.74 (dd, J = 12.4, 4.7 Hz, 1H), 2.00 (s, 6H), 1.79 (apparent t, J = 12.1 Hz, 1H), 1.63 (apparent quint, J = 7.8 Hz, 2H), 1.57 (apparent quint, J = 7.1 Hz, 2H), 1.43 – 1.34 (m, 2H) ppm.<br><br>**LRMS (ESI-MS)** *m/z* calculated for $C_{44}H_{75}N_4O_{33}S^-$ [M-H]$^-$: 1219.4, found: 1219.4
**52**	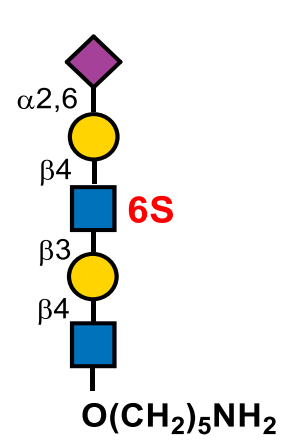<br>**52**	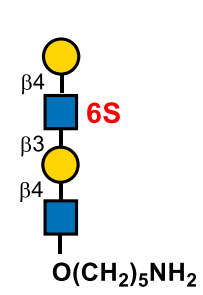<br>**49**	**II (hST6Gal1)**<br>(1.0 μmol)		**^{1}H NMR (600 MHz, D$_2$O)** δ = 4.74 (d, J = 8.1 Hz, 1H), 4.52 (d, J = 7.6 Hz, 1H), 4.50 – 4.45 (m, 2H), 4.43 (br d, J = 11.0 Hz, 1H), 4.28 (dd, J = 11.1, 5.9 Hz, 1H), 4.20 (d, J = 3.3 Hz, 1H), 4.03 – 3.51 (m, 30H), 2.98 (apparent t, J = 7.6 Hz, 2H), 2.67 (dd, J = 12.5, 4.8 Hz, 1H), 2.06 (s, 3H), 2.03 (s, 6H), 1.73 (apparent t, J = 12.2 Hz, 1H), 1.67 (apparent

					quint, J = 7.6 Hz, 2H), 1.60 (apparent quint, J = 6.5 Hz, 2H), 1.44 – 1.37 (m, 2H) ppm.<br><br>**LRMS (ESI-MS)** *m/z* calculated for $C_{44}H_{75}N_4O_{32}S^-$ [M-H]$^-$: 1203.4, found: 1203.4
**53**	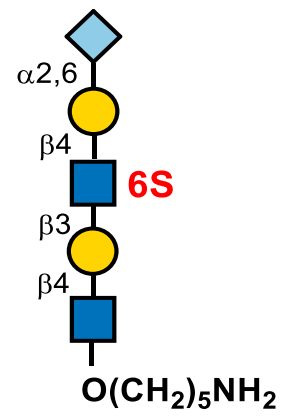<br>**53**	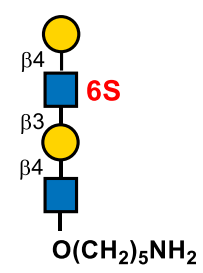<br>**49**	**II (hST6Gal1)**<br>(1.0 µmol)	98%<br>(1.2 mg)	**^{1}H NMR (600 MHz, D_2O)** δ = 4.71 (d, J = 8.1 Hz, 1H), 4.49 (d, J = 7.7 Hz, 1H), 4.45 (apparent t, J = 7.6 Hz, 2H), 4.40 (d, J = 10.9 Hz, 1H), 4.25 (dd, J = 11.1, 5.9 Hz, 1H), 4.18 (d, J = 3.3 Hz, 1H), 4.09 (s, 2H), 4.02 – 3.93 (m, 2H), 3.93 – 3.47 (m, 28H), 2.94 (apparent t, J = 7.6 Hz, 2H), 2.66 (dd, J = 12.4, 4.7 Hz, 1H), 2.03 (s, 3H), 2.01 (s, 3H), 1.72 (apparent t, J = 12.2 Hz, 1H), 1.64 (apparent quint, J = 7.7 Hz, 2H), 1.57 (apparent quint, J = 6.7 Hz, 2H), 1.43 – 1.31 (m, 2H) ppm.<br><br>**LRMS (ESI-MS)** *m/z* calculated for $C_{44}H_{75}N_4O_{33}S^-$ [M-H]$^-$: 1219.4, found: 1219.4
**54**	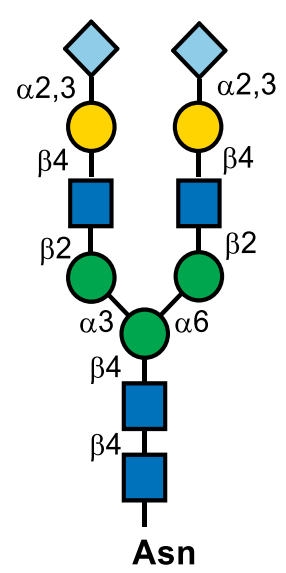<br>**54**	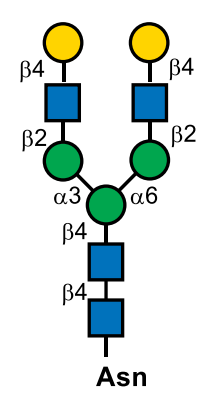<br>**C**<br>**(readily available from SGP)**	**I**<br>(0.5 µmol)	80-90%<br>(1.0 mg)	**^{1}H NMR (600 MHz, D_2O)** δ = 5.09 (s, 1H), 5.05 (d, J = 9.7 Hz, 1H), 4.90 (s, 1H), 4.74 (s, 1H), 4.59 (d, J = 8.2 Hz, 1H), 4.57 – 4.48 (m, 4H), 4.23 (d, J = 2.4 Hz, 1H), 4.17 (d, J = 1.8 Hz, 1H), 4.13 – 4.07 (m, 6H), 4.02 – 3.42 (m, 65H), 2.75 (dd, J = 12.4, 4.4 Hz, 2H), 2.65 (dd, J = 15.1, 4.6 Hz, 1H), 2.38 (dd, J = 15.2, 8.9 Hz, 1H), 2.06 (s, 3H), 2.02 (s, 3H), 2.02 (s, 3H), 1.98 (s, 3H), 1.79 (apparent t, J = 12.1 Hz, 2H) ppm.<br><br>**LRMS (ESI-MS)** *m/z* calculated for $C_{88}H_{142}N_8O_{66}^{2-}$ [M-2H]$^{2-}$: 1183.4, found: 1183.3
**55**	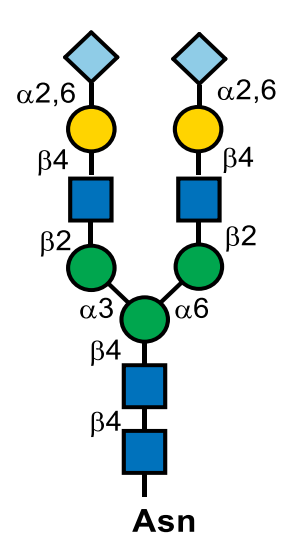<br>**55**	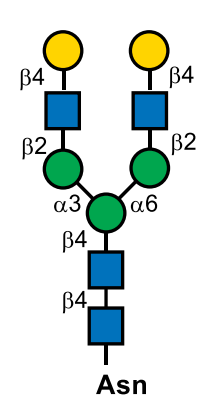<br>**C**<br>**(readily available from SGP)**	**II (hST6Gal1)**<br>(0.5 µmol)	80-90%<br>(1.0 mg)	**^{1}H NMR (600 MHz, D_2O)** δ = 5.11 (s, 1H), 5.05 (d, J = 9.8 Hz, 1H), 4.92 (s, 1H), 4.75 (s, 1H), 4.62 – 4.55 (m, 3H), 4.46 – 4.40 (m, 2H), 4.23 (br s, 1H), 4.17 (br s, 1H), 4.09 (apparent s, 5H), 4.04 – 3.68 (m, 41H), 3.67 – 3.44 (m, 25H), 2.73 (dd, J = 15.5, 4.3 Hz, 1H), 2.66 (br d, J = 12.2 Hz, 2H), 2.52 (dd, J = 15.5, 8.6 Hz, 1H), 2.08 – 2.02 (m, 9H), 1.99 (s, 3H), 1.72 (apparent t, J = 12.2 Hz, 2H) ppm.<br><br>**LRMS (ESI-MS)** *m/z* calculated for $C_{88}H_{142}N_8O_{66}^{2-}$ [M-2H]$^{2-}$: 1183.4, found: 1183.3

**56**	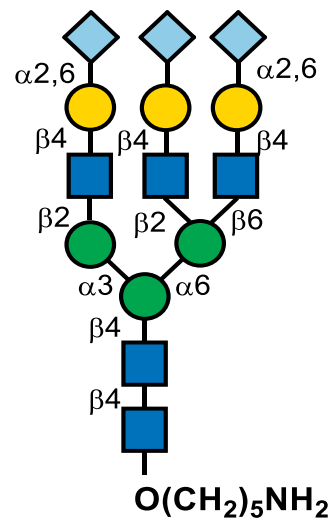<br>**56**	**Tris-galactoside (synthesis reported in ref.2, but NMR data were erroneously reported in ref.2)**	**II (hST6Gal1)**<br>(0.5 μmol)	60-70%<br>(1.0 mg)	**^{1}H NMR (600 MHz, D$_2$O)** δ = 4.99 (s, 1H), 4.76 (s, 1H), 4.64 (s, 1H), 4.50 – 4.37 (m, 4H), 4.37 – 4.26 (m, 4H), 4.13 (br s, 1H), 4.11 – 4.03 (m, 2H), 4.01 – 3.92 (m, 7H), 3.90 – 3.32 (m, 73H), 3.29 – 3.21 (m, 1H), 2.83 (apparent t, *J* = 7.7 Hz, 2H), 2.59 – 2.50 (m, 3H), 1.99 – 1.85 (m, 15H), 1.61 (apparent t, *J* = 11.3 Hz, 2H), 1.52 (apparent quint, *J* = 7.7 Hz, 2H), 1.45 (apparent quint, *J* = 7.3 Hz, 2H), 1.30 – 1.19 (m, 2H) ppm.<br><br>**LRMS (ESI-MS)** *m/z* calculated for $C_{114}H_{186}N_9O_{83}^{3-}$ $[M-3H]^{3-}$: 1003.4, found: 1003.4

Synthesis of Glycans A–C, 44, 45 and 48

Scheme S1. Synthesis of Trisaccharide Intermediate A[a]

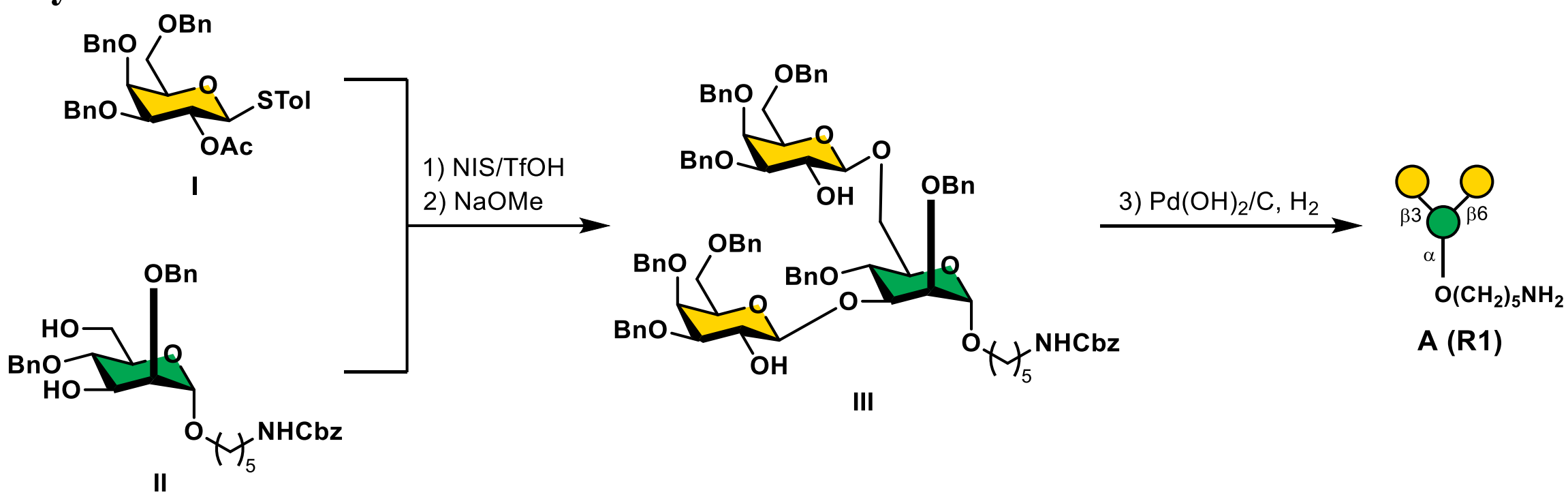

[a]Reagents and conditions: 1) **I** (2.5 equiv.), **II** (1.0 equiv.), NIS (2.5 equiv.), TfOH (0.5 equiv.), 4Å MS, CH_2Cl_2, −40 → 0 °C, 1 h; 2) NaOMe (0.5 equiv.), MeOH, r.t., 24 h, 82% overall; 3) $Pd(OH)_2/C$, H_2 (balloon), THF:H_2O:^{t}BuOH:AcOH (6:3:1:0.1, *v*/*v*/*v*/*v*), 24 h, 82%.

5-((benzyloxycarbonyl)amino)pentyl 3,4,6-tri-*O*-benzyl-β-D-galactopyranosyl-(1→3)-[3,4,6-tri-*O*-benzyl-β-D-galactopyranosyl-(1→6)]-2,4-di-*O*-benzyl-β-D-mannopyranoside (III): To a vigorously stirred suspension of **I**[2] (232 mg, 0.400 mmol, 1.0 equiv.), **II**[3] (598 mg, 1.00 mmol, 2.5 equiv.) and 4Å MS (1.0 g, flame dried) in 12 mL CH_2Cl_2 at −40 °C were sequentially added NIS (225 mg, 1.00 mmol, 2.5 equiv.) and TfOH (17.8 µL, 30.0 mg, 0.200 mmol, 0.5 equiv.). The darkened rection mixture was allowed to warm

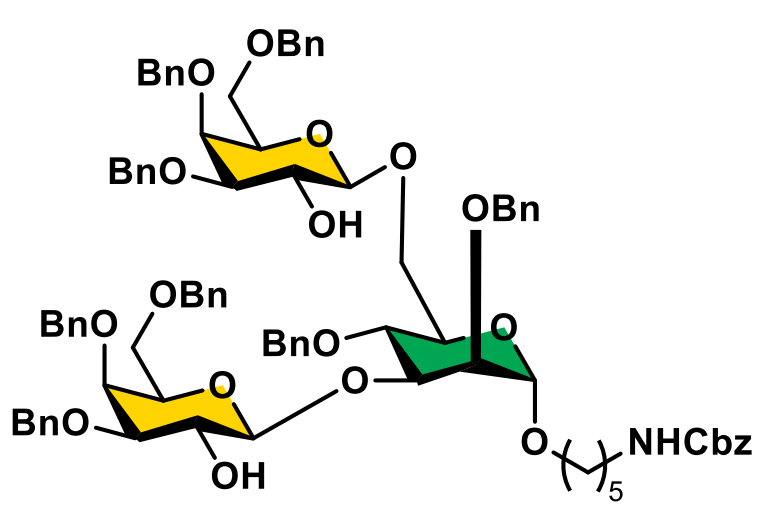

III

gradually to 0 °C over 1 h before it was quenched with a mixture of saturated aqueous $NaHCO_3$ and $Na_2S_2O_3$. The suspension was filtered through Celite® and the organic phase was separated. The aqueous phase was then washed with CH_2Cl_2 (10 mL × 3) and the organic phases were combined, dried over Na_2SO_4 and concentrated under vacuum. The resulting residue was redissolved in MeOH (10 mL) and to this slightly turbid solution was added NaOMe (37.0 µL, *ca.* 5.4 M in MeOH, 0.200 mmol, 0.5 equiv.) at room temperature. The resulting clear solution was stirred at the same temperature for another 24 h before it was neutralized with Amberlite™ IR-120 (0.3 g). The suspension was filtered, and the filtrate was concentrated under vacuum. The resulting residue was purified by flash chromatography (silica gel, EtOAc:hexanes = 1:4 → 1:2, *v*/*v*) to afford trisaccharide **III** (475 mg, 82% over the two steps) as an amorphous foam. **III**: ^{1}H NMR (600 MHz, $CDCl_3$) δ = 7.42 – 7.20 (m, 42H), 7.18 – 7.09 (m, 3H), 5.07 (s, 2H), 4.97 (d, *J* = 10.6 Hz, 1H), 4.95 – 4.87 (m, 3H), 4.80 – 4.70 (m, 6H), 4.67 (d, *J* = 11.9 Hz, 1H), 4.62 – 4.55 (m, 2H), 4.50 (d, *J* = 10.6 Hz, 1H), 4.47 – 4.37 (m, 4H), 4.35 – 4.28 (m, 2H), 4.22 – 4.15 (m, 2H), 4.06 – 3.98 (m, 2H), 3.93 (br d, *J* = 2.8 Hz, 1H), 3.90 (d, *J* = 2.9 Hz, 1H), 3.81 (t, *J* = 8.7 Hz, 1H), 3.79 – 3.72 (m, 3H), 3.67 – 3.53 (m, 6H), 3.49 – 3.40 (m, 3H), 3.31 (q, *J* = 7.0 Hz, 1H), 3.09 (quint, *J* = 6.6 Hz, 2H), 1.53 – 1.37 (m, 4H), 1.32 – 1.19 (m, 2H) ppm. HRMS (ESI-ToF) *m*/*z* calculated for $C_{87}H_{95}NO_{18}^{2-}$ $[M-2H]^{2-}$: 720.8280, found: 720.8290.

5-aminopentyl β-D-galactopyranosyl-(1→3)-[β-D-galactopyranosyl-(1→6)]-β-D-mannopyranoside (A): To a vigorously stirred suspension of trisaccharide **III** (30.0 mg, 20.8 µmol, 1.0 equiv.) and $Pd(OH)_2/C$ (20% *w*/*w*, 30 mg) in a mixed solvent of THF/H_2O/^{t}BuOH/AcOH (2.0 mL, 6:3:1:0.1, *v*/*v*/*v*/*v*) at room temperature was purged in hydrogen with a hydrogen balloon. The reaction was stirred for another 24 h before it was filtered

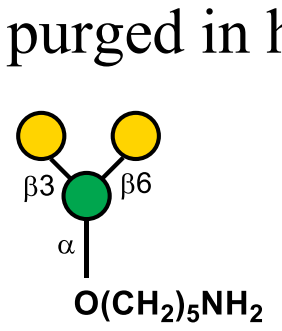

A (R1)

through Celite® and neutralized with aqueous ammonia (100 µL). The resulting clear solution was concentrated under vacuum, and the residue was purified on a G25 column to afford trisaccharide **A** (10.0 mg, 82%) as a fluffy white solid after lyophilization. **A**: ^{1}H NMR (600 MHz, D_2O) δ = 4.88 (d, *J* = 1.7 Hz, 1H), 4.48 (d, *J* = 7.8 Hz, 1H), 4.42 (d, *J* = 7.9 Hz, 1H), 4.18 (dd, *J* = 11.5, 2.0 Hz, 1H), 4.11 (apparent t, *J* = 2.5 Hz, 1H), 3.99 (dd, *J* = 9.5, 3.2 Hz, 1H), 3.94 – 3.83 (m, 4H), 3.83 – 3.61 (m, 10H), 3.61 – 3.49 (m, 3H), 2.97 (apparent t, *J* = 7.6 Hz, 2H), 1.76 – 1.55 (m, 4H), 1.49 – 1.36 (m, 2H) ppm; ^{13}C NMR (151 MHz, D_2O) δ = 103.3, 100.8, 99.5, 78.1, 75.3, 75.1, 72.6, 72.5, 71.6, 70.7, 70.6, 68.59, 68.57, 68.4, 67.63, 67.59, 64.8, 61.1, 61.0, 39.3, 27.9, 26.5, 22.3 ppm. HRMS (ESI-ToF) *m*/*z* calculated for $C_{23}H_{42}NO_{16}^-$ $[M-H]^-$: 588.2509, found: 588.2513.

Scheme S2. Synthesis of Tris-galactoside Acceptor B

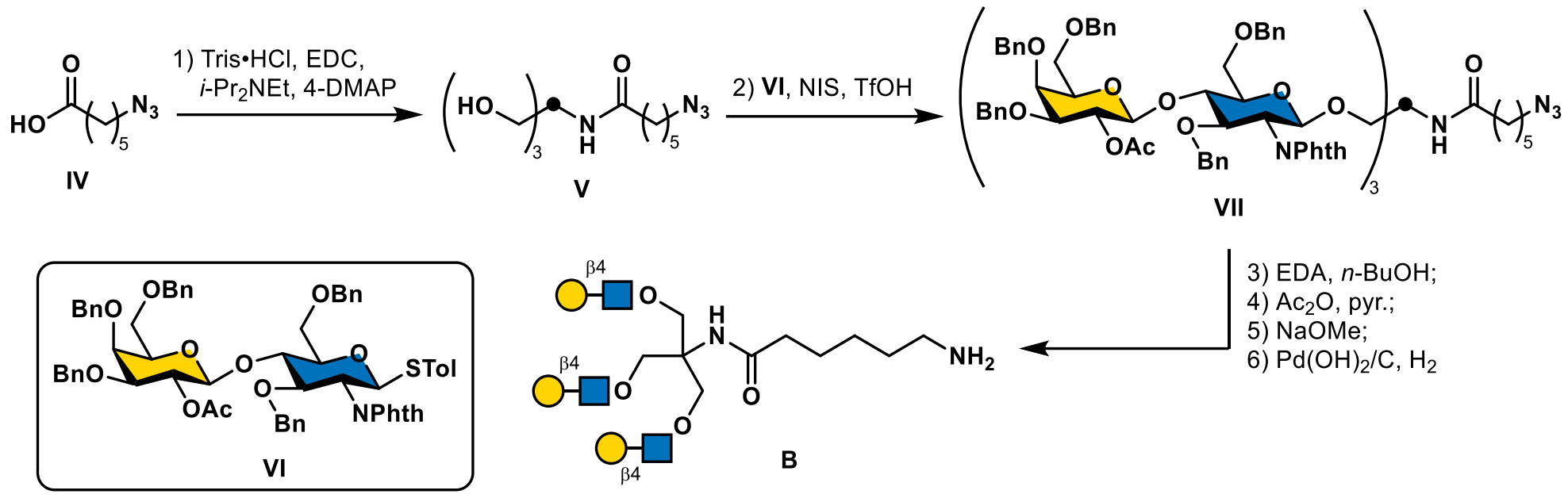

[a]Reagents and conditions: 1) **IV** (1.0 equiv.), Tris·HCl (1.0 equiv.), EDC (2.0 equiv.), *i*-Pr_2NEt (2.0 equiv.), 4-DMAP (0.2 equiv.), CH_2Cl_2, r.t., 24 h, 88%; 2) **VI** (4.0 equiv.), **V** (1.0 equiv.), NIS (4.0 equiv.), TfOH (0.5 equiv.), 4Å MS, −40 → 0 °C, 1 h, 39%; 3) EDA:*n*-BuOH (1:4, *v*/*v*), 90 °C, 18 h; 4) Ac_2O:pyridine (1:2, *v*/*v*), r.t., 24 h; 5) NaOMe, MeOH, r.t., 24 h; 6) $Pd(OH)_2/C$ (20%, *w*/*w*), THF:H_2O:*t*-BuOH:AcOH (60:30:10:1, *v*/*v*/*v*/*v*), H_2 (balloon), 60% overall.

6-Azido-*N*-(1,3-dihydroxy-2-(hydroxymethyl)propan-2-yl)hexanamide (V): to a stirred solution of 6-azidohexanoic acid (**IV**, 157 mg, 1.0 mmol, 1.0 equiv.) and Tris·HCl (158 mg, 1.0 mmol, 1.0 equiv.) in CH_2Cl_2 (10 mL) at room temperature were sequentially added EDC (383 mg, 2.0 mmol, 2.0 equiv.), *i*-Pr_2NEt (259 mg, 0.348 mL, 2.0 mmol, 2.0 equiv.) and 4-DMAP (24.4 mg, 0.2 mmol, 0.2 equiv.). The reaction mixture was allowed to stir for another 24 h before it was concentrated and purified by flash chromatography (silica gel, EtOAc:hexanes = 1:1 → EtOAc:MeOH = 10:1, *v*/*v*) to afford amide **V** (229 mg, 88%) as an off-white solid. **V**:

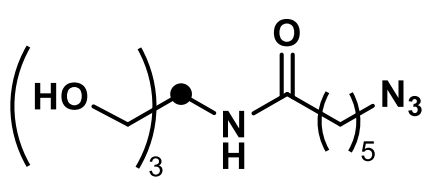

V

^1H NMR (400 MHz, $CDCl_3$) δ = 6.52 (br s, 1H), 3.59 (s, 6H), 3.28 (t, *J* = 6.8 Hz, 2H), 2.26 (t, *J* = 7.5 Hz, 2H), 1.74 – 1.51 (m, 4H), 1.51 – 1.33 (m, 2H) ppm; ^{13}C NMR (101 MHz, $CDCl_3$) δ = 61.5, 51.3, 36.7, 28.5, 26.0, 25.3 ppm. HRMS (ESI-ToF) *m*/*z* calculated for $C_{10}H_{19}N_4O_4^-$ [M-H]$^-$: 259.1412, found: 259.1417.

6-Azido-*N*-tris(3,4,6-tri-*O*-benzyl-2-*O*-acetyl-β-D-galactopyranosyl-(1→4)-3,6-di-*O*-benzyl-2-deoxy-2-phthalimido-β-D-glucopyranosyl methyl)methyl hexanamide (VII): to a stirred suspension of amide **V** (65.0 mg, 0.250 mmol, 1.0 equiv.), disaccharide donor **VI** (1.07 g , 1.00 mmol, 4.0 equiv.) and 4Å molecular sieves (1.0 g, flame dried) in CH_2Cl_2 (10 mL) at −40 °C were sequentially added NIS (225 mg, 1.00 mmol, 4.0 equiv.) and TfOH (18.8 mg, 11.1 µL, 0.125 mmol, 0.5 equiv.). The darkened reaction mixture was allowed to warm to 0 °C over 1 h before it was quenched by a mixture of saturated aqueous $NaHCO_3$ and $Na_2S_2O_3$. The resulting suspension was filtered through Celite® and the organic phase was separated. The aqueous phase was washed with CH_2Cl_2 (10 mL × 3) and the organic phases were combined, dried over Na_2SO_4 and concentrated under vacuum. The residue was purified by flash chromatography (silica gel, EtOAc:hexanes = 1:3 → 1:1, *v*/*v*) to afford hexasaccharide **VII** (305 mg, 39%) as an amorphous foam. **VII**: ^1H NMR (600 MHz, $CDCl_3$) δ = 7.80 – 7.55 (m, 12H), 7.42 – 7.12 (m, 60H), 6.91 – 6.73 (m, 15H), 5.96 (br s, 1H), 5.34 (dd, *J* = 10.0, 7.9 Hz, 3H), 4.92 (d, *J* = 11.6 Hz, 3H), 4.74 (d, *J* = 12.0 Hz, 3H), 4.70 – 4.62 (m, 6H), 4.49 (d, *J* = 11.6 Hz, 3H), 4.46 (d, *J* = 12.3 Hz, 3H), 4.39 – 4.27 (m, 12H), 4.24 (d, *J* = 11.7 Hz, 3H), 4.09 (d, *J* = 12.1 Hz, 3H), 3.94 – 3.81 (m, 12H), 3.72 – 3.63 (m, 6H), 3.53 (d, *J* = 10.6 Hz, 3H), 3.42 (apparent t, *J* = 8.0 Hz, 3H), 3.37 – 3.25 (m, 9H), 3.21 – 3.07 (m, 2H), 3.02 (d, *J* = 11.3 Hz, 3H), 2.95 (br d, *J* = 10.0 Hz, 3H), 2.07 (s, 9H), 1.78 (t, *J* = 7.1 Hz, 2H), 1.44 – 1.37 (m, 2H), 1.29 – 1.21 (m, 2H), 1.09 – 1.00 (m, 2H) ppm; ^{13}C NMR (151 MHz, $CDCl_3$) δ = 172.8, 169.6, 167.8, 138.95, 138.90, 138.09, 138.07, 134.2, 134.0, 131.9, 131.7, 128.57, 128.55, 128.3, 127.99, 127.98, 127.87, 127.85, 127.8, 127.7, 127.5, 127.4, 126.9, 123.4, 123.3, 100.6, 98.8, 80.4, 77.3, 76.9, 74.7, 74.6, 74.5, 73.5, 73.4, 73.2, 72.8, 72.1, 71.8, 68.1, 67.43, 67.36, 59.3, 55.5, 51.3, 36.3, 28.8, 25.9, 24.9, 21.3 ppm.

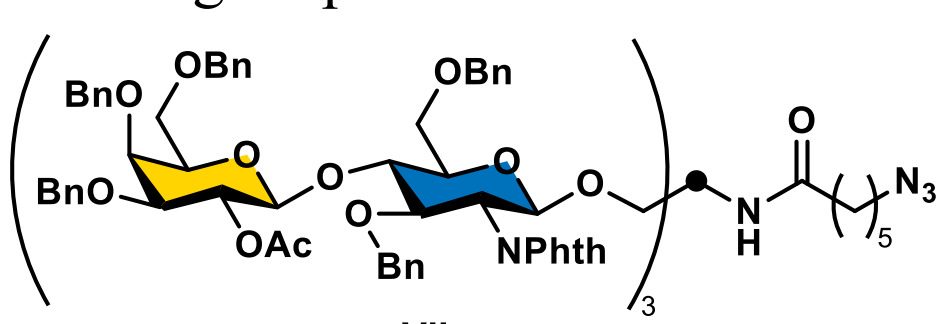

VII

6-Amino-*N*-tris(β-D-galactopyranosyl-(1→4)-2-deoxy-2-acetamido-β-D-glucopyranosyl methyl)methyl hexanamide (B): a solution of hexasaccharide **VII** (93.0 mg, 30.0 µmol, 1.0 equiv.) in ^{n}BuOH/EDA (3.5 mL, 4:1 *v*/*v*) was heated at 90 °C for 18 h. The reaction mixture was cooled to room temperature, concentrated under vacuum, and re-dissolved in pyridine/Ac_2O (3 mL, 2:1 *v*/*v*). The clear solution was stirred for another 24 h before it was concentrated under vacuum and azeotroped with toluene (10 mL × 3). The resulting residue was

then dissolved in MeOH (3.0 mL) and NaOMe (0.111 mL, *ca.* 5.4 M in MeOH, 0.600 mmol, 20 equiv.) was added at room temperature. The de-acetylation process continued for 12 h and the reaction mixture was diluted

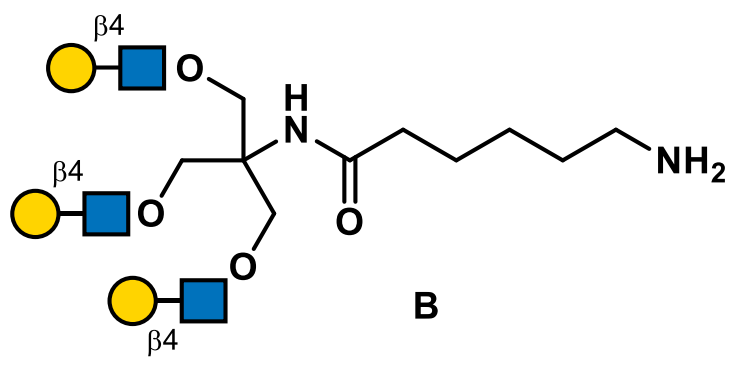

B

with MeOH (5 mL) and neutralized with Amberlite™ IR-120 (0.6 g, washed with MeOH). The suspension was filtered, concentrated and dried under vacuum. The reaction residue was purified by a flash column (silica gel, toluene:acetone = 7/3 → 1/1, *v*/*v*) to provide the corresponding multi-benzylated hexasaccharide as a glassy film. The latter was dissolved in a mixed solvent of THF, H_2O, ^{t}BuOH and AcOH (3.03 mL, 6:3:1:0.1, *v*/*v*/*v*/*v*) and to this stirred solution under argon was added $Pd(OH)_2/C$ (20% *w*/*w*, 50 mg). Hydrogen was purged in using a hydrogen balloon and the reaction was vigorously stirred at room temperature for another 24 h before it was filtered through Celite® and neutralized with aqueous ammonia (100 µL). The resulting clear solution was concentrated under vacuum, and the residue was purified on a G25 column to afford hexasaccharide **B** (24.0 mg, 60% overall) as a fluffy white solid after lyophilization. **B**: ^{1}H NMR (600 MHz, D_2O) δ = 4.45 (d, *J* = 7.8 Hz, 3H), 4.39 (d, *J* = 7.7 Hz, 3H), 4.06 (d, *J* = 10.0 Hz, 3H), 3.96 (dd, *J* = 12.4, 2.3 Hz, 3H), 3.90 (d, *J* = 3.5 Hz, 3H), 3.83 (dd, *J* = 12.4, 4.8 Hz, 3H), 3.78 – 3.62 (m, 24H), 3.58 – 3.53 (m, 3H), 3.52 (dd, *J* = 9.9, 7.8 Hz, 3H), 2.98 (apparent t, *J* = 7.6 Hz, 2H), 2.28 – 2.16 (m, 2H), 1.66 (apparent quint, *J* = 7.6 Hz, 2H), 1.58 (apparent hept, *J* = 6.9 Hz, 2H), 1.37 (apparent quint, *J* = 7.5 Hz, 2H) ppm; ^{13}C NMR (151 MHz, D_2O) δ = 176.5, 174.1, 102.8, 101.5, 78.3, 75.3, 74.7, 72.4, 72.0, 70.9, 68.5, 67.2, 61.0, 59.8, 59.1, 55.1, 39.2, 36.0, 26.4, 25.0, 24.6, 22.3 ppm. LRMS (ESI-MS) *m*/*z* calculated for $C_{52}H_{90}N_5O_{34}^-$ $[M-H]^-$: 1328.5, found: 1328.6

Scheme S3. Synthesis of Pentasaccharides 44 and 45

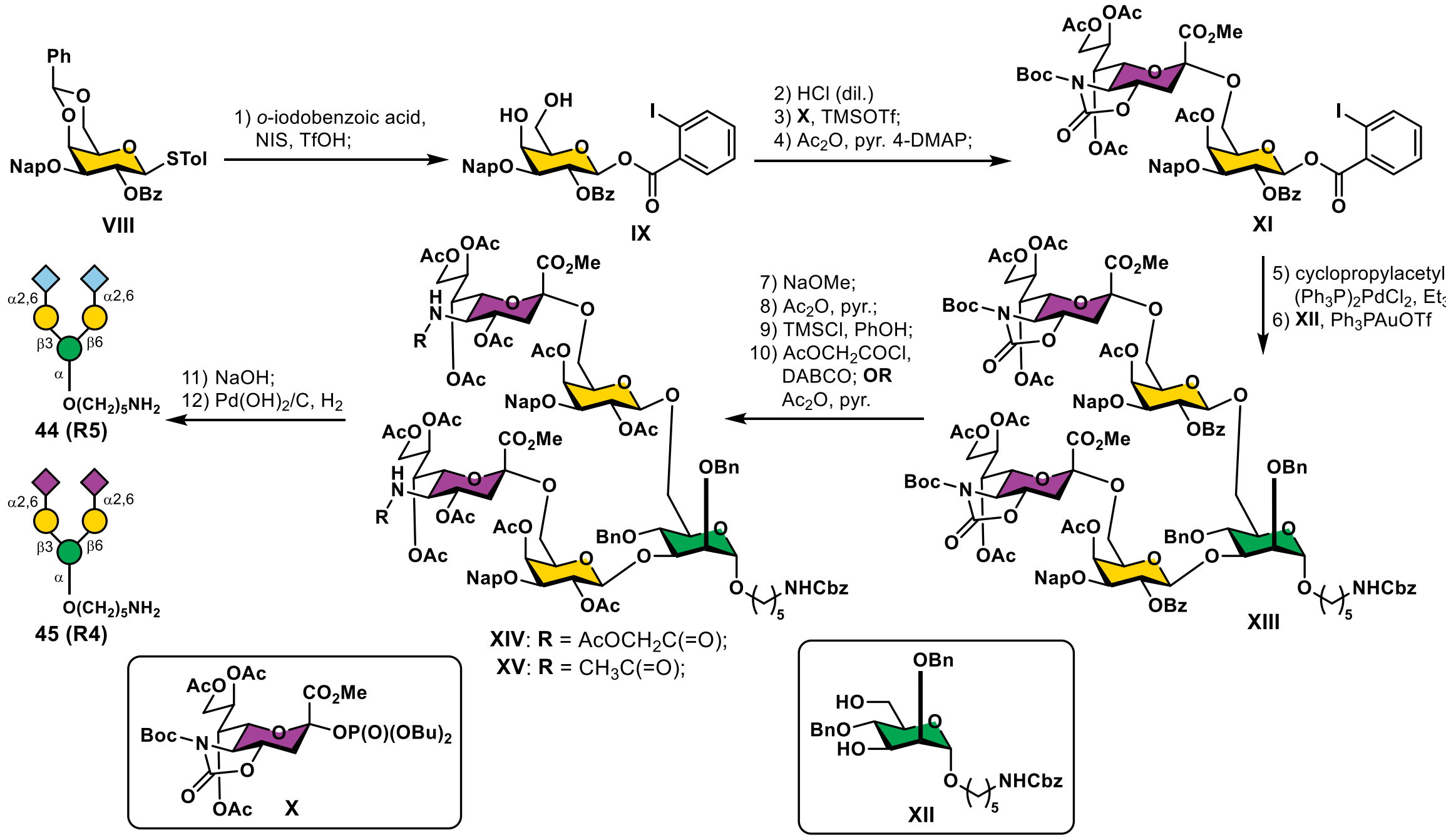

[a]Reagents and conditions: 1) *o*-iodobenzoic acid (2.0 equiv.), NIS (2.0 equiv.), TfOH (0.2 equiv.), 4Å MS, CH_2Cl_2, −40 → 0 °C, 1 h; 2) THF:*i*-PrOH:conc.HCl (20:50:1, *v*/*v*/*v*), 55 °C, 6 h, 73%; 3) **X** (1.1 equiv.), TMSOTf (1.1 equiv.), 4Å MS, CH_2Cl_2, −78 → −25 °C, 1 h; 4) Ac_2O (3.0 equiv.), pyridine (5.0 equiv.), 4-DMAP (0.5 equiv.), CH_2Cl_2, r.t., 24 h, 70% overall; 5) $(Ph_3P)_2PdCl_2$ (0.1 equiv.), CuI (0.2 equiv.), cyclopropylacetylene (5.0 equiv.), Et_3N:DMF (3:1, *v*/*v*), r.t., 24 h, 88%; 6) **XII** (1.0 equiv.), **XI-ABz** (2.5 equiv.), $Ph_3PAuOTf$ (1.0 equiv.), 4Å MS, CH_2Cl_2, 0 °C, 30 min, 52%; 7) NaOMe (10 equiv.), MeOH, r.t., 24 h; 8) Ac_2O:pyridine (1:3, *v*/*v*), r.t., 24 h; 9) TMSCl:PhOH (neat, 1:3, *mol*/*mol*), −78 → −20 °C, 1 h; 10) $AcOCH_2COCl$ (3.0 equiv.), DABCO (3.0 equiv.), CH_2Cl_2, 0 °C → r.t., 12 h; or Ac_2O:pyridine (1:3, *v*/*v*), r.t., 12 h, 48% overall for **XIV**, 52% overall for **XV**; 11) NaOMe:MeOH:H_2O (1:5:10, *v*/*v*/*v*), r.t., 48 h; 12) $Pd(OH)_2$/C, H_2 (balloon), THF:H_2O:^{t}BuOH:AcOH (6:3:1:0.1, *v*/*v*/*v*/*v*), 24 h, 75% overall for **44**, 83% overall for **45**.

4,6-Benzylidene-3-*O*-(2-naphthylmethyl)-2-*O*-benzoyl-β-D-galactopyranosyl *o*-iodobenzoate (IX): To a suspension of benzylidene **VIII** (1.0 g, 1.61 mmol, 1.0 equiv.), *o*-iodobenzoic acid (805 mg, 3.23 mmol, 2.0 equiv.) and 4Å MS (2.0 g, flame dried) in CH_2Cl_2 (25 mL) at −40 °C were sequentially added NIS (727 mg, 3.23 mmol, 2.0 equiv.) and TfOH (28.7 μL, 48.5 mg, 0.323 mmol, 0.2 equiv.). The darkened reaction mixture was allowed to warm to 0 °C over 1 h before it was quenched with a mixture of saturated aqueous $NaHCO_3$ and $Na_2S_2O_3$. The resulting suspension was filtered through Celite® and the organic layer was separated. The aqueous phase was washed with CH_2Cl_2 (10 mL × 3) and the organic layers were combined, dried over Na_2SO_4 and concentrated under vacuum. The crude was briefly purified by flash chromatography (silica gel, hexanes:EtOAc = 3/1, *v*/*v*) to afford the corresponding *o*-iodobenzoate (β:α-benzoate *ca*. 4:1, 900 mg, 75%) as a amorphous foam. ^{1}H NMR (600 MHz, $CDCl_3$,for major anomeric isomer) δ = 8.01 – 7.91 (m, 4H), 7.80 – 7.74 (m, 1H), 7.73 – 7.69 (m, 1H), 7.66 – 7.52 (m, 5H), 7.50 – 7.30 (m, 9H), 7.10 (td, *J* = 7.7, 1.7 Hz, 1H), 6.06 – 5.91 (m, 2H), 5.56 (s, 1H), 4.89 (d, *J* = 12.8 Hz, 1H), 4.82 (d, *J* = 12.8 Hz, 1H), 4.43 – 4.31 (m, 2H), 4.07 (dd, *J* = 12.6, 1.4 Hz, 1H), 3.92 (dd, *J* = 8.9, 3.4 Hz, 1H), 3.65 (s, 1H) ppm. HRMS (ESI-ToF) *m*/*z* calculated for $C_{31}H_{26}IO_8^-$ [M-H]$^-$: 653.0678, found: 653.0680

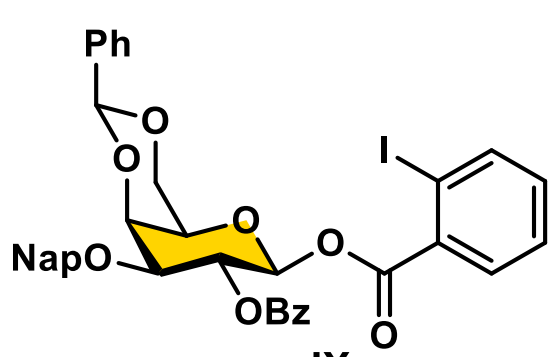

Methyl 5-*tert*-butylcarbamato-7,8,9-tri-*O*-acetyl-5,*N*-4,*O*-carbonyl-3,5-dideoxy-D-glycero-α-D-galacto-non-2-ulopyranosylonate-(2→6)-*o*-iodobenzoyl-4-*O*-acetyl-3-*O*-(2-naphthylmethyl)-2-*O*-benzoyl-β-D-galactopyranoside (XI): To a solution of *o*-iodobenzoate (900 mg, 1.21 mmol, 1.0 equiv.) in THF (6.0 mL) was slowly added HCl acidified *i*-PrOH (15 mL, *i*-PrOH:conc.HCl = 50:1, *v*/*v*) at room temperature. The homogeneous reaction solution was allowed to warm to 55 °C and stirred at the same temperature for another 6 h before it was cooled back to room temperature and neutralized with saturated aqueous $NaHCO_3$ (5 mL). The resulting suspension was concentrated, re-dissolved in EtOAc (20 mL) and washed with brine (10 mL × 2). The organic layer was separated, dried over Na_2SO_4 and concentrated under vacuum. The resulting crude was purified by a flash chromatography (silica gel, hexanes:EtOAc = 2:1 → 1:1, *v*/*v*) to afford diol **IX** (580 mg, 73%) as an amorphous foam. ^{1}H NMR (600 MHz, $CDCl_3$) δ = 7.95 – 7.87 (m, 3H), 7.73 (dd, *J* = 7.8, 1.7 Hz,

1H), 7.64 – 7.59 (m, 2H), 7.57 – 7.53 (m, 2H), 7.47 – 7.41 (m, 2H), 7.39 – 7.30 (m, 4H), 7.28 – 7.25 (m, 1H), 7.08 (td, *J* = 7.7, 1.7 Hz, 1H), 5.96 (d, *J* = 8.4 Hz, 1H), 5.83 (dd, *J* = 9.7, 8.4 Hz, 1H), 4.88 (d, *J* = 12.5 Hz, 1H), 4.69 (d, *J* = 12.4 Hz, 1H), 4.29 (dd, *J* = 3.2, 1.2 Hz, 1H), 4.03 (dd, *J* = 11.7, 6.0 Hz, 1H), 3.94 (dd, *J* = 11.7, 5.0 Hz, 1H), 3.83 (dd, *J* = 9.7, 3.2 Hz, 1H), 3.79 (t, *J* = 5.6 Hz, 1H) ppm. To a suspension of diol **IX** (425 mg, 0.649 mmol, 1.0 equiv.), sialyl phosphate donor (518 mg, 0.714 mmol, 1.1 equiv.) and 4Å MS (1.0 g, flame dried) in CH_2Cl_2 (10 mL) at −78 °C was added dropwise TMSOTf (0.129 mL, 159 mg, 0.714 mmol, 1.1 equiv.). The reaction mixture was allowed to slowly be warmed to −25 °C over 1 h before it was quenched with saturated aqueous $NaHCO_3$ (10 mL). The resulting suspension was filtered through Celite® and the organic layer was separated. The aqueous layer was washed with CH_2Cl_2 (10 mL × 3) and the organic layers were combined, dried over Na_2SO_4 and concentrated under vacuum. The crude was dissolved in CH_2Cl_2 (15 mL) and to this resulting solution were sequentially added pyridine (0.260 mL, 0.257 mg, 3.25 mmol, 5.0 equiv.), Ac_2O (0.184 mL, 199 mg, 1.95 mmol, 3.0 equiv.) and 4-DMAP (40.0 mg, 0.325 mmol, 0.5 equiv.) at room temperature. The reaction mixture was allowed to stir at room temperature for 24 h before it was quenched with saturated aqueous $NaHCO_3$ (10 mL). The organic layer was separated, and the aqueous layer was washed with CH_2Cl_2 (10 mL ×3). The organic layers were combined, dried over Na_2SO_4 and concentrated under vacuum. The resulting reaction crude was briefly purified by a flash chromatography (silica gel, hexanes:EtOAc = 3/2, *v*/*v*) to afford disaccharyl *o*-iodobenzoate **XI** (β:α-benzoate *ca*. 4:1, 550 mg, 70%) as an off-white foam. ^{1}H NMR (600 MHz, $CDCl_3$, for major anomeric isomer) δ = 7.95 – 7.88 (m, 4H), 7.73 – 7.70 (m, 1H), 7.63 – 7.60 (m, 1H), 7.57 – 7.51 (m, 2H), 7.49 (d, *J* = 8.4 Hz, 1H), 7.45 – 7.31 (m, 5H), 7.21 (dd, *J* = 8.4, 1.7 Hz, 1H), 7.10 (td, *J* = 8.0, 2.0 Hz, 1H), 6.01 (d, *J* = 8.4 Hz, 1H), 5.74 (d, *J* = 3.2 Hz, 1H), 5.69 (dd, *J* = 10.0, 8.3 Hz, 1H), 5.45 (ddd, *J* = 8.7, 6.3, 2.8 Hz, 1H), 5.36 (dd, *J* = 8.2, 1.7 Hz, 1H), 4.88 (d, *J* = 12.6 Hz, 1H), 4.63 (d, *J* = 12.6 Hz, 1H), 4.55 (dd, *J* = 9.4, 1.7 Hz, 1H), 4.34 (dd, *J* = 12.3, 2.8 Hz, 1H), 4.14 (dd, *J* = 12.3, 6.3 Hz, 1H), 4.05 (apparent t, *J* = 6.4 Hz, 1H), 4.02 – 3.94 (m, 2H), 3.91 (dd, *J* = 10.0, 3.4 Hz, 1H), 3.81 (s, 3H), 3.72 (dd, *J* = 11.1, 9.4 Hz, 1H), 3.50 (dd, *J* = 10.0, 7.0 Hz, 1H), 2.88 (dd, *J* = 12.1, 3.6 Hz, 1H), 2.24 (s, 3H), 2.13 (s, 3H), 2.11 – 2.10 (m, 1H), 2.10 (s, 3H), 2.04 (s, 3H), 1.53 (s, 9H) ppm; ^{13}C NMR (151 MHz, $CDCl_3$, for major anomeric isomer) δ = 170.8, 170.3, 170.0, 168.4, 165.5, 163.8, 152.6, 150.9, 141.8, 134.7, 133.6, 133.4, 133.12, 133.07, 132.2, 132.1, 130.0, 129.5, 128.51, 128.48, 128.3, 128.2, 127.9, 127.7, 127.0, 126.2, 126.1, 126.0, 99.4, 95.1, 93.2, 85.3, 76.6, 75.5, 74.5, 73.1, 71.6, 70.4, 70.0, 68.7, 65.9, 63.3, 63.1, 58.7, 53.3, 36.8, 27.9, 21.3, 21.1, 21.0, 20.9 ppm. LRMS (ESI-MS) *m*/*z* calculated for $C_{55}H_{58}INO_{22}^+$ $M^{\cdot+}$: 1211.2, found: 1211.2

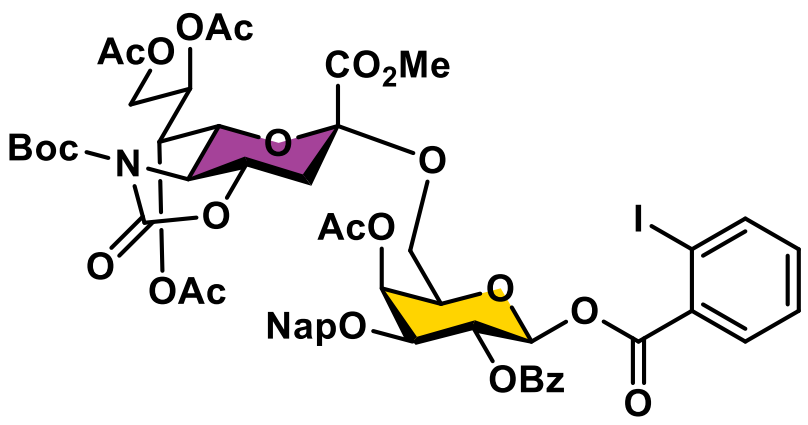

XI

5-Benzylcarbamatopentyl bis[methyl 5-*tert*-butylcarbamato-7,8,9-tri-*O*-acetyl-5,*N*-4,*O*-carbonyl-3,5-dideoxy-D-glycero-α-D-galacto-non-2-ulopyranosylonate-(2→6)-4-*O*-acetyl-3-*O*-(2-naphthylmethyl)-2-*O*-benzoyl-β-D-galactopyranosyl-(1→3) and (1→6)]-2,4-di-*O*-benzyl-α-D-mannopyranoside (XIII): To a solution of disaccharide *o*-iodobenzoate (550 mg, 0.454 mmol, 1.0 equiv.) in a mixture of Et_3N and DMF (16 mL, Et_3N:DMF = 3:1, *v*/*v*) at room temperature were sequentially added $(Ph_3P)_2PdCl_2$ (31.8 mg, 45.4 μmol, 0.1 equiv.) and CuI (17.7 mg, 90.8 μmol, 0.2 equiv.). The greenish solution was purged with argon for 15 min before cyclopropylacetylene (0.192 mL, 150 mg, 2.27 mmol, 5.0 equiv.) was added. The resulting dark red solution was allowed to stir for another 24 h at room temperature before it was dried under vacuum. The residue was purified by flash chromatography (silica gel, hexanes:EtOAc = 2/1 → 1/1, *v*/*v*) to afford *o*-cyclopropylethynyl benzoate **XI-ABz** (460 mg, 88%) as a pale-yellow foam. ^{1}H NMR (400 MHz, $CDCl_3$) δ = 7.93 (dd, *J* = 8.0, 1.3 Hz, 1H), 7.88 (dd, *J* = 8.3, 1.4 Hz, 2H), 7.71 (dd, *J* = 7.7, 1.6 Hz, 1H), 7.63 – 7.61 (m, 1H), 7.56 – 7.46 (m, 3H), 7.44 – 7.28 (m, 6H), 7.25 – 7.18 (m, 2H), 6.02 (d, *J* = 8.4 Hz, 1H), 5.73 (d, *J* = 3.4 Hz, 1H), 5.70 (dd, *J* = 10.0, 8.4 Hz, 1H), 5.45 (ddd, *J* = 8.8, 6.2, 2.8 Hz, 1H), 5.36 (dd, *J* = 8.2, 1.7 Hz, 1H), 4.88 (d, *J* = 12.6 Hz, 1H), 4.63 (d, *J* = 12.5 Hz, 1H), 4.55 (dd, *J* = 9.4, 1.7 Hz, 1H), 4.34 (dd, *J* = 12.3, 2.8 Hz, 1H), 4.14 (dd, *J* = 12.3, 6.2 Hz, 1H), 4.07 – 3.89 (m, 4H), 3.81 (s, 3H), 3.72 (dd, *J* = 11.3, 9.5 Hz, 1H), 3.50 (dd, *J* = 9.5, 6.4 Hz, 1H),

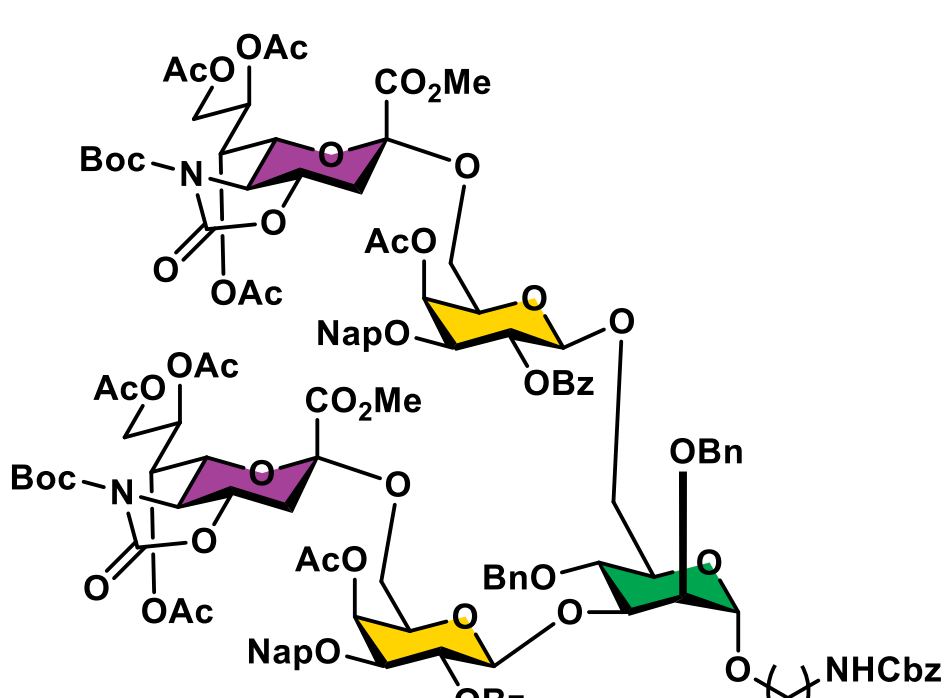

XIII

2.90 – 2.85 (m, 1H), 2.25 (s, 3H), 2.16 – 2.13 (m, 1H), 2.12 (s, 3H), 2.09 (s, 3H), 2.03 (s, 3H), 1.53 (s, 9H), 1.50 – 1.42 (m, 1H), 0.93 – 0.82 (m, 4H) ppm. To a suspension of diol **XII**[3] (87.0 mg, 0.150 mmol, 1.0 equiv.), *o*-cyclopropylethynylbenzoate **XI** (431 mg, 0.375 mmol, 2.5 equiv.) and 4ÅMS (600 mg, flame dried) in CH_2Cl_2 (8 mL) at 0 °C was slowly added a solution of $Ph_3PAuOTf$[4] (3.00 mL, *ca.* 0.05 M in CH_2Cl_2, 0.15 mmol, 1.0 equiv.). The reaction mixture was kept at 0 °C for another 30 min before it was quenched with saturated aqueous $NaHCO_3$ (10 mL). The resulting suspension was filtered through Celite® and the organic layer was separated. The aqueous layer was washed with CH_2Cl_2 (10 mL × 3) and the organic layers were combined, dried over Na_2SO_4 and concentrated under vacuum. The crude was briefly purified by flash chromatography (silica gel, hexanes:EtOAc = 2/1 → 1/1, *v*/*v*) to afford pentasaccharide **XIII** (195 mg, 52%) as an amorphous foam. ^{1}H NMR (500 MHz, $CDCl_3$, peaks were generally broadened due to severe rotameric effect of this compound) δ = 7.97 – 7.78 (m, 4H), 7.77 – 7.64 (m, 4H), 7.63 – 7.04 (m, 29H), 6.95 (br s, 2H), 5.73 – 5.57 (m, 2H), 5.51 – 5.25 (m, 5H), 5.11 (s, 3H), 4.96 – 4.69 (m, 4H), 4.67 – 4.42 (m, 5H), 4.42 – 4.27 (m, 3H), 4.27 – 4.03 (m, 5H), 4.02 – 3.34 (m, 20H), 3.22 – 3.01 (m, 3H), 2.87 (br d, *J* = 12.0 Hz, 1H), 2.76 (br d, *J* = 11.3 Hz, 1H), 2.70 – 2.52 (m, 2H), 2.32 – 1.82 (m, 26H), 1.55 (s, 18H), 1.28 (br s, 6H), 1.04 (br s, 4H) ppm.

5-benzylcarbamatopentyl bis[methyl 5-(2-acetoxy)acetamido-4,7,8,9-tetra-*O*-acetyl-3,5-dideoxy-D-glycero-α-D-galacto-non-2-ulopyranosylonate-(2→6)-4-*O*-acetyl-3-*O*-(2-naphthylmethyl)-2-*O*-benzoyl-β-D-galactopyranosyl-(1→3) and (1→6)]-2,4-di-*O*-benzyl-α-D-mannopyranoside (XIV) and 5-benzylcarbamatopentyl bis[methyl 5-acetamido-4,7,8,9-tetra-*O*-acetyl-3,5-dideoxy-D-glycero-α-D-galacto-non-2-ulopyranosylonate-(2→6)-4-*O*-acetyl-3-*O*-(2-naphthylmethyl)-2-*O*-benzoyl-β-D-galactopyranosyl-(1→3) and (1→6)]-2,4-di-*O*-benzyl-α-D-mannopyranoside (XV): To a solution of pentasaccharide **XIII** (80.0 mg, 31.9 µmol, 1.0 equiv.) in MeOH (5 mL) was added NaOMe (59.0 µL, *ca.* 5.4 M in MeOH, 0.319 mmol, 10 equiv.) at room temperature. The reaction mixture was allowed to stir for another 24 h before it was neutralized by Amberlite™ IRC120 (300 mg). The resulting suspension was filtered, dried under vacuum and the crude reaction residue was re-dissolved in a mixture of pyridine/Ac_2O (4 mL, 3:1, *v*/*v*) and the homogenous solution was allowed to stir at room temperature for another 24 h. All the volatiles were removed under vacuum, and the resulting crude was treated with a mixture of TMSCl and PhOH (3 mL, 1:3, *mol*/*mol*) at −78 °C. The reaction mixture was allowed to slowly warm to −20 °C over 1 h before it was poured into a cooled $NaHCO_3$ solution. The resulting suspension was extracted with CH_2Cl_2 (10 mL ×3) and the organic phases were combined, dried over Na_2SO_4 and concentrated under vacuum. The residue was briefly purified on a short column (silica gel, CH_2Cl_2:MeOH = 1:0 →10:1, *v*/*v*) to get rid of excessive PhOH. To a solution of free amine in CH_2Cl_2 (3 mL) were sequentially added acetoxyacetyl chloride (10.3 µL, 13.1 mg, 95.7 µmol, 3.0 equiv.) and DABCO (10.7 mg, 95.7 µmol, 3.0 equiv.) at 0° C. The reaction mixture was allowed to warm to room temperature and then kept at this temperature for another 12 h before it was quenched by saturated aqueous $NaHCO_3$ (10 mL). The organic layer was separated, and the aqueous layer was extracted with CH_2Cl_2 (5 mL × 3). The organic phases were combined, dried over Na_2SO_4 and concentrated under vacuum. The resulting residue was briefly purified on a short column (silica gel, straight EtOAc) to afford bis(2-acetoxyacetamide) **XIV** (37.0 mg, 48%) as a glassy film. Alternatively, the free amine obtained in the last step was treated with a mixture of pyridine and Ac_2O (3.0 mL, 3:1, *v*/*v*) at room temperature for 12 h. All the volatiles were removed under vacuum, and the residue was briefly purified on a short column (silica gel, straight EtOAc) to afford bis(acetamide) **XV** (38.1 mg, 52%) as a glassy film.

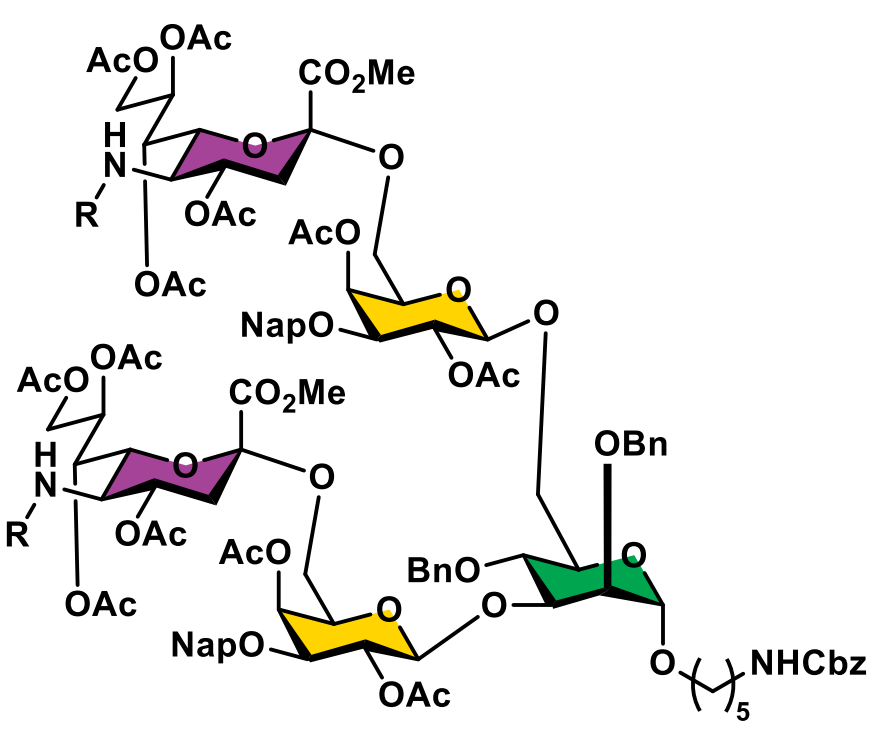

XIV: **R** = $AcOCH_2C(=O)$;
XV: **R** = $CH_3C(=O)$;

5-Aminopentyl bis[5-(2-hydroxy)acetamido-3,5-dideoxy-D-glycero-α-D-galacto-non-2-ulopyranosylonate-(2→6)-β-D-galactopyranosyl-(1→3) and (1→6)]-α-D-mannopyranoside (44) and 5-aminopentyl bis[5-acetamido-3,5-dideoxy-D-glycero-α-D-galacto-non-2-ulopyranosylonate-(2→6)-β-D-galactopyranosyl-(1→3) and (1→6)]-α-D-mannopyranoside (45): to a solution of bis(Neu5Gc) pentasaccharide **XIV** (24.0 mg, 10.0 µmol, 1.0 equiv.) in a mixed solvent of MeOH and H_2O (1.5 mL, MeOH:H_2O = 1:2, *v*/*v*) at room

temperature was added NaOMe (0.100 mL, *ca.* 5.4 M in MeOH, 0.540 mmol, 54 equiv.). The clear reaction mixture was stirred for another 48 h before it was neutralized with Amberlite™ IRC120 (600 mg). The resulting suspension was filtered, and the filtrate was concentrated under vacuum. The residue was directly dissolved in a mixed solvent of THF, H_2O, ^{t}BuOH and AcOH (2.02 mL, THF:H_2O:^{t}BuOH:AcOH = 6:3:1:0.1, *v*/*v*/*v*/*v*). To this solution at room temperature under argon was added $Pd(OH)_2$/C (25 mg, 20% *w*/*w*) and the suspension was purged with hydrogen by a hydrogen balloon. The reaction mixture was vigorously stirred for another 24 h before it was filtered through Celite®. The filtrate was concentrated to complete dryness under vacuum, and the residue was basified with aqueous NaOH (50 µL, *ca.* 1.0 M, 50 µmol, 5.0 equiv.) before it was purified on a G25 Sephadex column to afford the final bis(Neu5Gc) pentasaccharide **44** (10.0 mg, 75%) as a fluffy solid after lyophilization. ^{1}H NMR (600 MHz, D_2O) δ = 4.89 (s, 1H), 4.47 (d, *J* = 7.8 Hz, 1H), 4.44 (d, *J* = 7.9 Hz, 1H), 4.19 (d, *J* = 11.2 Hz, 1H), 4.15 – 4.07 (m, 5H), 4.03 – 3.72 (m, 21H), 3.68 – 3.49 (m, 11H), 3.00 (t, *J* = 7.6 Hz, 2H), 2.72 (apparent dt, *J* = 12.4, 5.1 Hz, 2H), 1.78 – 1.59 (m, 6H), 1.52 – 1.39 (m, 2H) ppm; ^{13}C NMR (151 MHz, D_2O) δ = 175.7, 103.4, 101.4, 100.5, 100.3, 99.5, 79.1, 73.7, 73.3, 72.4, 72.3, 72.2, 71.7, 71.3, 70.7, 70.6, 68.61, 68.58, 68.5, 68.2, 68.1, 67.95, 67.94, 67.91, 67.6, 64.9, 63.7, 63.2, 62.55, 62.51, 60.93, 60.91, 51.50, 51.46, 40.20, 40.16, 39.4, 27.9, 26.4, 22.3 ppm. LRMS (ESI-MS) *m*/*z* calculated for $C_{45}H_{75}N_3O_{34}^{2-}$ [M-2H]$^{2-}$: 600.7, found: 600.7. Bis(Neu5Ac) pentasaccharide **XV** (23.0 mg, 10.0 µmol, 1.0 equiv.) was converted to the corresponding free glycan **45** (10.8 mg, 83%) using the same procedure. ^{1}H NMR (500 MHz, D_2O) δ = 4.92 (s, 1H), 4.51 (d, *J* = 7.8 Hz, 1H), 4.47 (d, *J* = 7.9 Hz, 1H), 4.22 (dd, *J* = 11.5, 2.1 Hz, 1H), 4.14 (dd, *J* = 3.2, 1.8 Hz, 1H), 4.04 – 3.75 (m, 17H), 3.75 – 3.52 (m, 15H), 3.03 (t, *J* = 7.6 Hz, 2H), 2.74 (apparent dt, *J* = 12.3, 4.8 Hz, 2H), 2.04 (s, 6H), 1.79 – 1.63 (m, 6H), 1.56 – 1.42 (m, 2H) ppm; ^{13}C NMR (151 MHz, D_2O) δ = 174.95, 174.90, 173.4, 103.4, 101.4, 100.4, 100.3, 99.5, 79.1, 73.6, 73.3, 72.6, 72.5, 72.37, 72.36, 71.7, 71.3, 70.7, 70.6, 68.59, 68.56, 68.5, 68.22, 68.19, 68.18, 68.15, 67.9, 67.6, 64.9, 63.6, 63.2, 62.6, 62.5, 51.81, 51.77, 40.2, 40.1, 39.4, 27.9, 26.4, 22.3, 22.01, 21.98 ppm. LRMS (ESI-MS) *m*/*z* calculated for $C_{45}H_{75}N_3O_{32}^{2-}$ [M-2H]$^{2-}$: 584.7, found: 584.7.

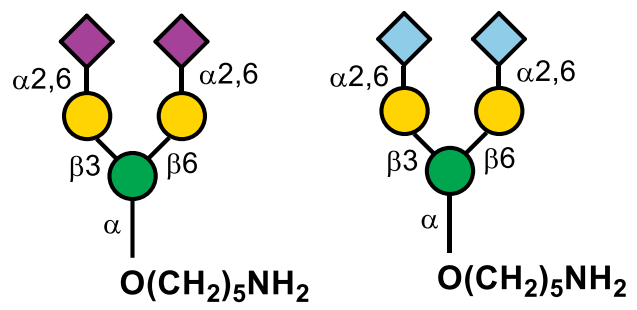

45 (R4) **44 (R5)**

Scheme S4. Chemical Synthesis of Sulfated Trisaccharide 48

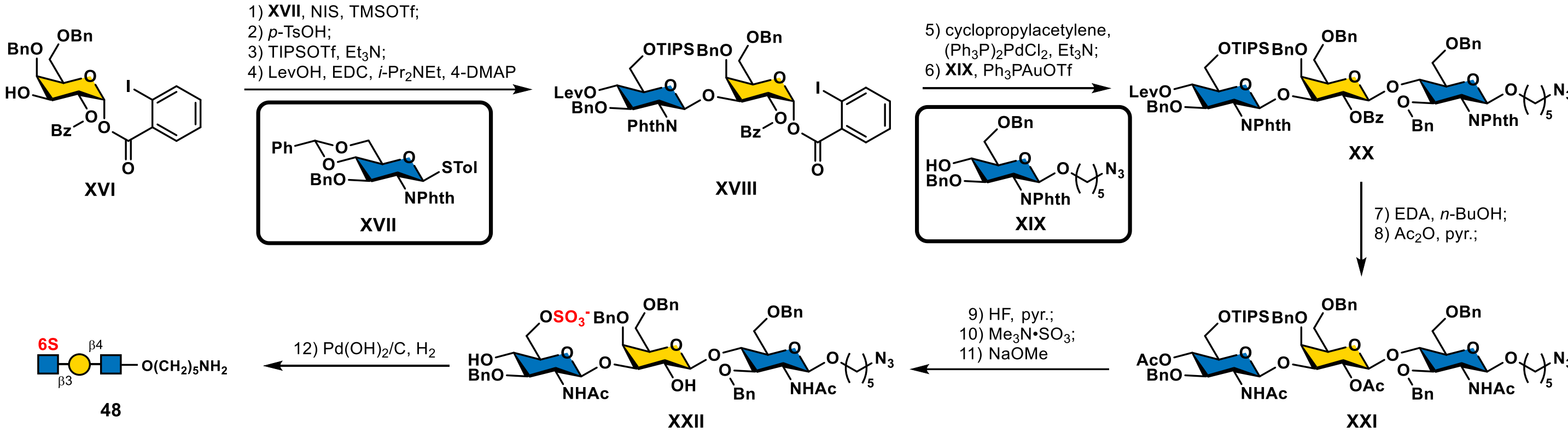

^{a}Reagents and conditions: 1) **XVI** (1.0 equiv.), **XVII** (1.1 equiv.), NIS (1.1 equiv.), TMSOTf (0.15 equiv.), 4ÅMS, CH_2Cl_2, −40 → −20°C, 30 min; 2) *p*-TsOH (0.5 equiv.), MeCN, r.t., 24 h; 3) TIPSOTf (2.0 equiv.), Et_3N (3.0 equiv.), CH_2Cl_2, 0 °C → r.t., 12 h; 4) LevOH (2.0 equiv.), EDC (2.0 equiv.), *i*-Pr_2NEt (2.0 equiv.), 4-DMAP (0.2 equiv.), CH_2Cl_2, r.t., 12 h, 55% overall; 5) $(Ph_3P)_2PdCl_2$ (0.1 equiv.), CuI (0.2 equiv.), cyclopropylacetylene (5.0 equiv.), Et_3N:DMF (3:1, *v*/*v*), r.t., 24 h, 85%; 6) **XVIII-ABz** (1.0 equiv.), **XIX** (1.2 equiv.), $Ph_3PAuOTf$ (0.2 equiv.), 4Å MS, CH_2Cl_2, 0 °C, 30 min, 73%; 7) EDA:*n*-BuOH (1:4, *v*/*v*), 90 °C, 18 h; 8) Ac_2O:pyridine (1:2, *v*/*v*), r.t., 24 h, 79% overall; 9) HF:pyridine (3:7 *w*/*w*), THF, 0 °C, 4 h; 10) $Me_3N·SO_3$ (5.0 equiv.), pyridine, 80 °C, 3 h; 11) NaOMe (5.0 equiv.), MeOH, r.t., 24 h, 60% overall; 12) $Pd(OH)_2$/C (20%, *w*/*w*), THF:H_2O:*t*-BuOH (6:3:1, *v*/*v*/*v*), H_2 (balloon), 24 h, 85%.

3-*O*-benzyl-4-*O*-(4-oxopentanoyl)-6-*O*-triisopropylsilyl-2-deoxy-2-phthalimido-β-D-glucopyranosyl-(1→3)-4,6-di-*O*-benzyl-2-*O*-benzoyl-α-D-galactopyranosyl 2-iodobenzoate (XVIII): to a suspension of **XVI**[5] (prepared from the corresponding *p*-tolyl thioglycoside, 1.00 g, 1.44 mmol, 1.0 equiv.), **XVII**[2] (1.20 g, 1.58 mmol, 1.1 equiv.), NIS (356 mg, 1.58 mmol, 1.1 equiv.) and 4Å MS (1.5 g, flame dried) in CH_2Cl_2 (20 mL) at −40 °C was slowly added TMSOTf (39.1 µL, 48.0 mg, 0.216 mmol, 0.15 equiv.). The darkened suspension was allowed to warm to −20 °C over 30 min before it was quenched with a mixture of saturated aqueous $NaHCO_3$ and $Na_2S_2O_3$ (20 mL). The resulting suspension was filtered through Celite® and the organic layer was separated. The aqueous phase was washed with CH_2Cl_2 (15 mL × 2) and the organic phases were combined, dried over Na_2SO_4 and concentrated under vacuum. The crude was briefly purified by flash chromatography (silica gel, hexanes:EtOAc = 2/1, *v*/*v*) to afford the corresponding *o*-iodobenzoate as an amorphous foam, which was re-dissolved in MeCN (25 mL). To this solution at room temperature was added *p*-TsOH (137 mg, 0.720 mmol, .0.5 equiv.) and the reaction mixture was allowed to stir at the same temperature for another 24 h before it was quenched with saturated aqueous $NaHCO_3$ (10 mL). The reaction mixture was concentrated under vacuum, and the aqueous phase was extracted with EtOAc (20 mL ×3). The organic phases were combined, dried over Na_2SO_4, and concentrated under vacuum. The residue was re-dissolved in CH_2Cl_2 (25 mL) and to this solution at 0 °C were sequentially added Et_3N (0.600 mL, 437 mg, 4.32 mmol, 3.0 equiv.) and TIPSOTf (0.770 mL, 882 mg, 2.88 mmol, 2.0 equiv.). The reaction mixture was allowed to slowly warm to room temperature and was stirred for another 12 h before it was quenched with saturated aqueous $NaHCO_3$ (10 mL). The organic phase was separated, dried over Na_2SO_4 and concentrated under vacuum. The resulting residue was dissolved in CH_2Cl_2 (20 mL) and to this solution were sequentially added levulinic acid (334 mg, 2.88 mmol, 2.0 equiv.), EDC (552 mg, 2.88 mmol, 2.0 equiv.), *i*-Pr_2NEt (0.500 mL, 372 mg, 2.88 mmol, 2.0 equiv.) and 4-DMAP (88.0 mg, 0.720 mmol, 0.5 equiv.) at room temperature. The reaction mixture was allowed to stir at the same temperature for another 12 h before it was quenched with $NaHCO_3$ (20 mL). The organic phase was separated, dried over Na_2SO_4 and concentrated under vacuum. The residue was purified by flash chromatography (silica gel, hexanes:EtOAc = 4:1 → 2:1, *v*/*v*) to afford disaccharide **XVIII** (1.05 g, 55%) as a pale-yellow foam. **XVIII**: ^{1}H NMR (600 MHz, $CDCl_3$) δ = 7.96 (d, *J* = 7.9 Hz, 1H), 7.71 – 7.62 (m, 3H), 7.60 – 7.49 (m, 3H), 7.42 – 7.33 (m, 7H), 7.33 – 7.25 (m, 7H), 7.17 (td, *J* = 7.7, 1.7 Hz, 1H), 6.93 (apparent d, *J* = 6.9 Hz, 2H), 6.87 (apparent t, *J* = 7.4 Hz, 2H), 6.85 – 6.80 (m, 1H), 6.60 (br d, *J* = 7.4 Hz, 1H), 6.56 (d, *J* = 3.9 Hz, 1H), 5.55 (dd, *J* = 10.5, 3.9 Hz, 1H), 5.35 (d, *J* = 8.3 Hz, 1H), 5.07 (apparent t, *J* = 9.5 Hz, 1H), 5.04 (d, *J* = 11.3 Hz, 1H), 4.60 (d, *J* = 11.3 Hz, 1H), 4.56 (d, *J* = 12.2 Hz, 1H), 4.53 – 4.46 (m, 2H), 4.41 (d, *J* = 12.1 Hz, 1H), 4.39 (dd, *J* = 10.5, 3.0 Hz, 1H), 4.31 – 4.24 (m, 3H), 4.21 (apparent t, *J* = 2.2 Hz, 1H), 3.84 (dd, *J* = 11.2, 2.2 Hz, 1H), 3.79 (dd, *J* = 11.2, 6.6 Hz, 1H), 3.71 (ddd, *J* = 9.2, 6.7, 2.2 Hz, 1H), 3.60 (dd,

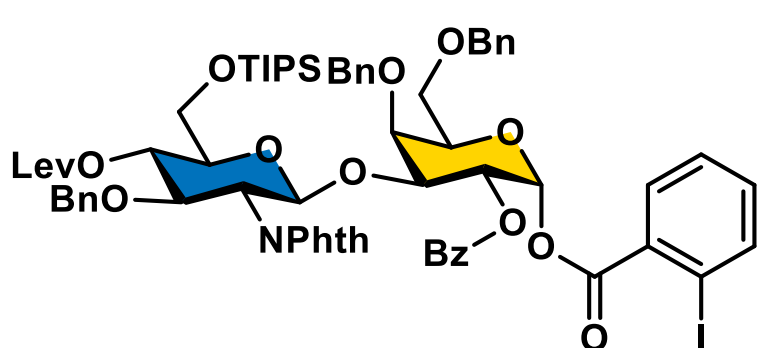

XVIII

J = 9.9, 6.4 Hz, 1H), 3.49 (dd, J = 9.9, 5.4 Hz, 1H), 2.78 – 2.59 (m, 2H), 2.54 (ddd, J = 17.2, 7.5, 5.6 Hz, 1H), 2.44 (dt, J = 17.3, 6.4 Hz, 1H), 2.15 (s, 3H), 1.15 – 1.06 (m, 3H), 1.03 (d, J = 6.9 Hz, 18H) ppm; ^{13}C NMR (151 MHz, $CDCl_3$) δ = 206.0, 171.6, 168.1, 167.0, 164.9, 141.4, 138.5, 138.0, 137.9, 135.2, 133.6, 133.4, 133.1, 132.9, 131.2, 130.7, 130.1, 128.9, 128.8, 128.44, 128.38, 128.2, 128.1, 128.0, 127.9, 127.8, 127.7, 127.5, 127.3, 123.4, 122.8, 99.6, 93.8, 91.8, 76.95, 76.91, 76.5, 75.6, 75.3, 74.1, 73.6, 73.2, 72.1, 69.3, 69.1, 62.9, 56.0, 37.8, 29.8, 28.0, 18.11, 18.09, 11.9 ppm. LRMS (ESI-MS) *m/z* calculated for $C_{69}H_{76}INNaO_{16}Si^+$ $[M+Na]^+$: 1352.4, found: 1352.5

5-Azidopentyl 3-*O*-benzyl-4-*O*-(4-oxopentanoyl)-6-*O*-triisopropylsilyl-2-deoxy-2-phthalimido-β-D-glucopyranosyl-(1→3)-4,6-di-*O*-benzyl-2-*O*-benzoyl-β-D-galactopyranosyl-(1→4)-3,6-di-*O*-benzyl-2-deoxy-2-phthalimido-β-D-glucopyranoside (XX): to a solution of *o*-iodobenzoate **XVIII** (1.0 g, 0.752 mmol, 1.0 equiv.) in a mixture of Et_3N and DMF (20 mL, Et_3N:DMF = 3:1, *v*/*v*) at room temperature were sequentially added $(Ph_3P)_2PdCl_2$ (52.8 mg, 75.2 μmol, 0.1 equiv.) and CuI (28.6 mg,0.150 mmol, 0.2 equiv.). The greenish solution was purged with argon for 15 min before cyclopropylacetylene (0.295 mL, 250mg, 3.76 mmol, 5.0 equiv.) was added. The resulting dark red solution was allowed to stir for another 24 h at room temperature before it was dried under vacuum. The residue was purified by flash chromatography (silica gel, hexanes:EtOAc = 3/1 → 2/1, *v*/*v*) to afford *o*-cyclopropylethynyl benzoate **XVIII-ABz** (810 mg, 85%) as a pale-yellow foam. **XVIII-ABz**: ^{1}H NMR (600 MHz, $CDCl_3$) δ = 7.73 (d, J = 7.8 Hz, 1H), 7.67 (br d, J = 7.4 Hz, 1H), 7.60 – 7.56 (m, 2H), 7.52 (t, J = 7.3 Hz, 2H), 7.47 – 7.41 (m, 2H), 7.40 – 7.34 (m, 4H), 7.33 – 7.24 (m, 10H), 6.91 (d, J = 7.0 Hz, 2H), 6.86 (t, J = 7.3 Hz, 2H), 6.82 (t, J = 7.1 Hz, 1H), 6.59 (br d, J = 7.4 Hz, 1H), 6.51 (d, J = 3.9 Hz, 1H), 5.53 (dd, J = 10.4, 3.9 Hz, 1H), 5.35 (d, J = 8.3 Hz, 1H), 5.08 (apparent t, J = 9.5 Hz, 1H), 5.04 (d, J = 11.2 Hz, 1H), 4.58 (d, J = 11.3 Hz, 1H), 4.54 (d, J = 12.1 Hz, 1H), 4.50 – 4.44 (m, 2H), 4.40 (d, J = 12.0 Hz, 1H), 4.33 (dd, J = 10.4, 2.9 Hz, 1H), 4.29 – 4.20 (m, 4H), 3.84 (dd, J = 11.2, 2.3 Hz, 1H), 3.80 (dd, J = 11.2, 6.3 Hz, 1H), 3.71 (ddd, J = 9.0, 6.2, 2.4 Hz, 1H), 3.57 (dd, J = 9.6, 6.0 Hz, 1H), 3.53 (dd, J = 9.6, 6.3 Hz, 1H), 2.76 – 2.62 (m, 2H), 2.53 (ddd, J = 17.3, 7.4, 5.8 Hz, 1H), 2.44 (dt, J = 17.3, 6.4 Hz, 1H), 2.14 (s, 3H), 1.27 (tt, J = 8.1, 5.2 Hz, 1H), 1.15 – 1.05 (m, 3H), 1.05 – 1.00 (m, 18H), 0.74 – 0.58 (m, 4H) ppm; ^{13}C NMR (151 MHz, $CDCl_3$) δ = 206.0, 171.6, 168.2, 167.0, 164.9, 164.0, 138.7, 138.0, 137.9, 134.5, 133.6, 133.4, 132.9, 132.0, 131.3, 131.2, 130.8, 130.3, 130.0, 129.1, 128.8, 128.5, 128.4, 128.22, 128.18, 128.1, 127.8, 127.7, 127.6, 127.4, 127.0, 125.2, 123.5, 122.8, 100.0, 99.7, 91.0, 77.0, 76.7, 75.6, 75.5, 74.3, 74.0, 73.7, 72.6, 72.2, 69.5, 68.8, 62.9, 56.0, 37.9, 29.9, 28.0, 18.14, 18.12, 12.0, 9.1, 9.0, 0.7 ppm. LRMS (ESI-MS) *m/z* calculated for $C_{74}H_{81}NNaO_{16}Si^+$ $[M+Na]^+$: 1290.5, found: 1290.5. To a suspension of **XVIII-ABz** (800 mg, 0.630 mmol, 1.0 equiv.), **XIX** (454 mg, 0.757 mmol, 1.2 equiv.) and 4Å MS (1.0 g, flame dried) in CH_2Cl_2 (20 mL) at 0 °C was slowly added a solution of $Ph_3PAuOTf$ (2.52 mL, *ca.* 0.05 M in CH_2Cl_2, 0.126 mmol, 0.2 equiv.). The reaction mixture was stirred at the same temperature for another 30 min before it was quenched by saturated aqueous $NaHCO_3$. The resulting suspension was filtered, and the organic phase of the filtrate was separated. The aqueous phase was extracted with CH_2Cl_2 (15 mL × 3). The organic phases were combined, dried over Na_2SO_4 and concentrated under vacuum. The residue was purified by flash chromatography (silica gel, hexanes:EtOAc = 4:1 → 2:1, *v*/*v*) to afford trisaccharide **XX** (776 mg, 73%) as a pale-yellow foam. **XX**: ^{1}H NMR (600 MHz, $CDCl_3$) δ = 7.83 – 7.53 (m, 9H), 7.44 – 7.20 (m, 18H), 6.95 – 6.73 (m, 11H), 5.33 (dd, J = 10.0, 8.1 Hz, 1H), 5.21 (d, J = 8.3 Hz, 1H), 5.08 (dd, J = 10.1, 8.9 Hz, 1H), 5.04 (d, J = 11.6 Hz, 1H), 4.92 – 4.85 (m, 1H), 4.82 (d, J = 12.1 Hz, 1H), 4.62 (d, J = 12.3 Hz, 1H), 4.57 – 4.44 (m, 4H), 4.39 (d, J = 12.0 Hz, 1H), 4.31 – 4.19 (m, 5H), 4.09 – 4.03 (m, 2H), 4.03 – 3.99 (m, 1H), 3.93 – 3.78 (m, 4H), 3.72 (ddd, J = 10.2, 6.4, 2.2 Hz, 1H), 3.65 (dt, J = 10.0, 6.1 Hz, 1H), 3.56 (apparent t, J = 6.2 Hz, 1H), 3.47 – 3.38 (m, 3H), 3.30 – 3.18 (m, 2H), 3.07 (ddd, J = 9.9, 3.7, 1.8 Hz, 1H), 2.93 – 2.79 (m, 2H), 2.77 – 2.62 (m, 2H), 2.55 (ddd, J = 17.3, 7.5, 5.8 Hz, 1H), 2.45 (dt, J = 17.3, 6.4 Hz, 1H), 2.15 (s, 3H), 1.40 – 1.24 (m, 4H), 1.18 – 1.09 (m, 3H), 1.09 – 0.98 (m, 20H) ppm; ^{13}C NMR (151 MHz, $CDCl_3$) δ = 206.0, 171.5, 168.1, 167.0, 164.5, 139.2, 139.0, 138.5, 138.4, 138.0, 133.8, 133.6, 133.3, 132.9, 131.5, 131.0, 129.9, 129.6, 128.6, 128.40, 128.35, 128.22, 128.18, 128.1, 128.0, 127.9, 127.7, 127.5, 127.4, 127.32, 127.28, 126.7, 123.43, 123.35, 123.2, 122.8, 100.6, 99.4, 98.2, 79.5, 77.10, 77.05, 76.8, 75.5, 75.1, 74.7, 74.6, 74.2, 74.0, 73.5, 73.4, 72.4, 72.1, 69.0,

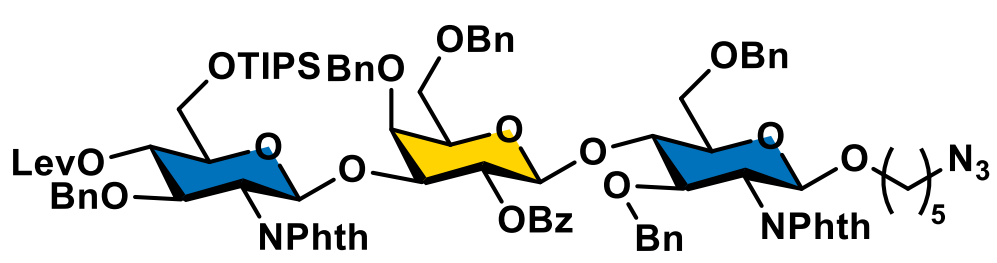

XX

68.9, 67.5, 62.9, 56.0, 55.7, 51.2, 37.8, 29.9, 28.7, 28.3, 28.0, 23.1, 18.10, 18.08, 11.9 ppm. LRMS (ESI-MS) *m*/*z* calculated for $C_{95}H_{107}N_5NaO_{21}Si^+$ $[M+Na]^+$: 1704.7, found: 1704.7

5-Azidopentyl 3-*O*-benzyl-4-*O*-acetyl-6-*O*-triisopropylsilyl-2-deoxy-2-acetamido-β-D-glucopyranosyl-(1→3)-4,6-di-*O*-benzyl-2-*O*-acetyl-β-D-galactopyranosyl-(1→4)-3,6-di-*O*-benzyl-2-deoxy-2-acetamido-β-D-glucopyranoside (XXI): a solution of trisaccharide **XX** (168 mg, 0.100 mmol, 1.0 equiv.) in ^{n}BuOH/EDA (5.0 mL, 4:1 *v*/*v*) was heated at 90 °C for 18 h. The reaction mixture was cooled to room temperature, concentrated under vacuum, and re-dissolved in pyridine/Ac_2O (6 mL, 2:1 *v*/*v*). The clear solution was stirred for another 24 h before it was concentrated under vacuum and azeotroped with toluene (20 mL × 3). The resulting residue was purified briefly by flash chromatography (silica gel, hexanes:EtOAc = 1:1 → 1:2, *v*/*v*) to afford tetra-acetate **XXI** (110 mg, 79% overall). **XXI**: ^{1}H NMR (600 MHz, $CDCl_3$) δ = 7.34 – 7.19 (m, 25H),

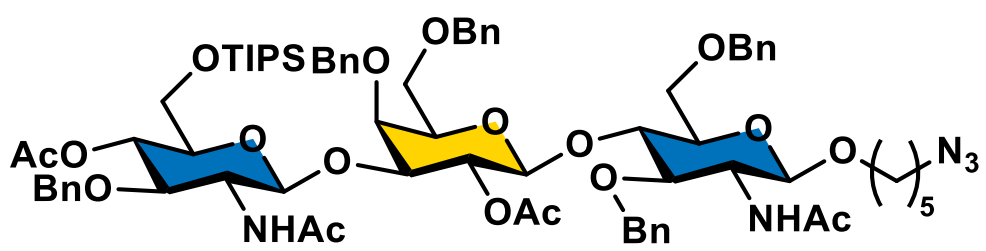

XXI

6.12 (br d, *J* = 8.9 Hz, 1H), 5.51 (d, *J* = 6.9 Hz, 1H), 5.22 (dd, *J* = 10.1, 8.0 Hz, 1H), 5.19 (d, *J* = 8.1 Hz, 1H), 4.94 (apparent t, *J* = 9.6 Hz, 1H), 4.82 (d, *J* = 12.1 Hz, 1H), 4.68 – 4.60 (m, 3H), 4.60 – 4.52 (m, 5H), 4.46 (d, *J* = 12.0 Hz, 1H), 4.42 (d, *J* = 1.9 Hz, 1H), 4.35 – 4.30 (m, 2H), 3.97 – 3.92 (m, 2H), 3.90 (d, *J* = 3.3 Hz, 1H), 3.85 – 3.66 (m, 8H), 3.55 (ddd, *J* = 10.1, 5.4, 3.3 Hz, 1H), 3.52 – 3.45 (m, 3H), 3.37 (apparent dt, *J* = 10.1, 6.8 Hz, 1H), 3.18 (t, *J* = 6.9 Hz, 2H), 2.73 (apparent dt, *J* = 10.5, 7.5 Hz, 1H), 2.08 (s, 3H), 1.95 (s, 3H), 1.94 (s, 3H), 1.82 (s, 3H), 1.58 – 1.50 (m, 4H), 1.39 – 1.32 (m, 2H), 1.09 – 1.03 (m, 3H), 1.01 (d, *J* = 6.1 Hz, 18H) ppm. LRMS (ESI-MS) *m*/*z* calculated for $C_{75}H_{101}N_5NaO_{18}Si^+$ $[M+Na]^+$: 1410.7, found: 1410.7

5-Azidopentyl 3-*O*-benzyl-6-*O*-sulfo-2-deoxy-2-acetamido-β-D-glucopyranosyl-(1→3)-4,6-di-*O*-benzyl-β-D-galactopyranosyl-(1→4)-3,6-di-*O*-benzyl-2-deoxy-2-acetamido-β-D-glucopyranoside (XXII): to a solution of trisaccharide **XXI** (105 mg, 75.6 μmol, 1.0 equiv.) in THF (4.0 mL) at 0 °C were sequentially added pyridine (0.150 mL, 147 mg, 1.86 mmol, 25 equiv.) and HF·py (0.100 mL, 110 mg). The reaction mixture was kept at this temperature for another 4 h before it was poured into cooled saturated aqueous $NaHCO_3$. The organic phase was separated, and the aqueous phase was extracted with EtOAc (10 mL × 3). The organic phases were combined, dried over Na_2SO_4 and concentrated under vacuum. The resulting residue was directly re-dissolved in pyridine (3.0 mL) and to this solution at room temperature was added $Me_3N·SO_3$ (52.6 mg,

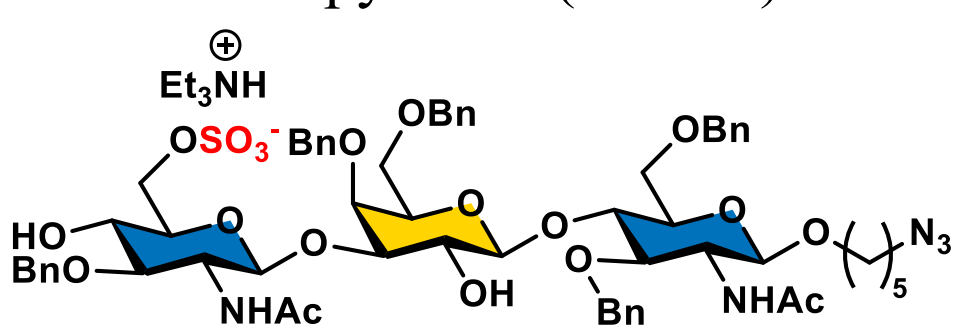

XXII

0.378 mmol, 5.0 equiv.). The reaction mixture was stirred at 80 °C for additional 3 h before it was cooled back to room temperature. The resulting residue was then re-dissolved in MeOH (3.0 mL) and to this solution at room temperature was added NaOMe (0.140 mL, *ca.* 5.4 M in MeOH, 0.756 mmol, 10.0 equiv.). The reaction mixture was allowed to stir for another 24 h before it was neutralized with Amberlite™ IRC120 (800 mg). The suspension was filtered, basified with Et_3N (0.100 mL), dried under vacuum and purified by flash chromatography (silica gel, toluene:MeOH = 10:1 → 5:1, *v*/*v*) to afford sulfated trisaccharide **XXII** (60 mg, 60% overall) as a glassy colorless film. **XXII**: ^{1}H NMR (600 MHz, MeOD) δ = 7.38 – 7.09 (m, 25H), 5.00 (d, *J* = 12.0 Hz, 1H), 4.98 (d, *J* = 11.4 Hz, 1H), 4.90 (d, *J* = 11.4 Hz, 1H), 4.76 (d, *J* = 8.4 Hz, 1H), 4.69 (d, *J* = 11.5 Hz, 1H), 4.65 (d, *J* = 11.9 Hz, 1H), 4.54 (d, *J* = 11.9 Hz, 1H), 4.53 – 4.49 (m, 2H), 4.45 (d, *J* = 8.3 Hz, 1H), 4.42 (dd, *J* = 11.0, 1.5 Hz, 1H), 4.37 (d, *J* = 7.7 Hz, 1H), 4.32 (d, *J* = 11.9 Hz, 1H), 4.24 (dd, *J* = 10.9, 5.1 Hz, 1H), 4.16 (d, *J* = 12.0 Hz, 1H), 4.01 – 3.95 (m, 3H), 3.90 – 3.81 (m, 3H), 3.77 (br t, *J* = 9.3 Hz, 1H), 3.71 (dd, *J* = 9.9, 7.7 Hz, 1H), 3.65 – 3.44 (m, 9H), 3.39 (dd, *J* = 10.0, 6.7 Hz, 1H), 3.26 (apparent t, *J* = 6.8 Hz, 2H), 1.93 – 1.89 (m, 6H), 1.63 – 1.53 (m, 4H), 1.47 – 1.39 (m, 2H) ppm; ^{13}C NMR (151 MHz, MeOD) δ = 173.6, 173.1, 140.8, 140.5, 140.3, 139.8, 139.6, 129.5, 129.4, 129.3, 129.2, 129.1, 129.00, 128.96, 128.93, 128.92, 128.8, 128.5, 128.4, 128.17, 128.15, 104.2, 104.0, 102.7, 84.0, 83.9, 81.9, 78.0, 77.2, 76.4, 76.1, 75.9, 75.6, 75.3, 75.0, 74.4, 74.3, 72.8, 72.0, 70.6, 70.3, 69.6, 68.3, 56.7, 56.2, 52.5, 30.1, 29.6, 24.4, 23.2, 23.0 ppm. LRMS (ESI-MS) *m*/*z* calculated for $C_{62}H_{76}N_5O_{19}S^-$ $[M-Et_3NH^+]^-$: 1226.5, found: 1226.6

5-Aminopentyl 6-*O*-sulfo-2-deoxy-2-acetamido-β-D-glucopyranosyl-(1→3)-β-D-galactopyranosyl-(1→4)-2-deoxy-2-acetamido-β-D-glucopyranoside (48): To a solution of benzylated trisaccharide **XXII** (40 mg, 30.0 μmol, 1.0 equiv.) in a mixed solvent of THF, H_2O and *t*-BuOH (2.0 mL, THF:H_2O:*t*-BuOH = 6:3:1, *v*/*v*/*v*) was added $Pd(OH)_2$/C (40 mg, 20% *w*/*w*) under argon. The resulting suspension was purged with hydrogen

using a hydrogen balloon and the reaction mixture was vigorously stirred at room temperature for another 24 h before it was filtered through Celite®. The filtrate was concentrated under vacuum and was purified on a P2

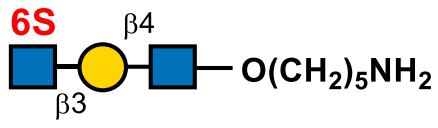

48

column to afford the debenzylated product **48** (19.0 mg, 85%) as a fluffy solid after lyophilization. **48**: ^{1}H NMR (600 MHz, D_2O) δ = 4.70 (d, *J* = 8.5 Hz, 1H), 4.53 (d, *J* = 7.5 Hz, 1H), 4.47 (d, *J* = 7.9 Hz, 1H), 4.34 (dd, *J* = 11.2, 2.1 Hz, 1H), 4.23 (dd, *J* = 11.2, 5.8 Hz, 1H), 4.20 (d, *J* = 3.3 Hz, 1H), 4.00 (dd, *J* = 12.3, 2.2 Hz, 1H), 3.92 (apparent dt, *J* = 10.1, 6.0 Hz, 1H), 3.86 – 3.65 (m, 10H), 3.64 – 3.56 (m, 4H), 3.52 (apparent t, *J* = 9.4 Hz, 1H), 3.00 (apparent t, *J* = 7.5 Hz, 2H), 2.05 (s, 3H), 2.04 (s, 3H), 1.68 (apparent quint, *J* = 7.7 Hz, 2H), 1.61 (apparent quint, *J* = 6.5 Hz, 2H), 1.44 – 1.37 (m, 2H) ppm; ^{13}C NMR (151 MHz, D_2O) δ = 174.9, 174.4, 102.9, 102.8, 101.1, 82.3, 78.6, 75.1, 74.8, 73.49, 73.47, 72.4, 70.1, 69.9, 69.6, 68.3, 67.2, 61.1, 60.1, 55.6, 55.0, 39.3, 28.0, 26.3, 22.14, 22.09 ppm. LRMS (ESI-MS) *m*/*z* calculated for $C_{27}H_{48}N_3O_{19}S^-$ $[M-H]^-$: 750.3, found: 750.3

Scheme S5. Synthesis of G2-Asn Glycan from SGP by Enzymatic Digestion[5]

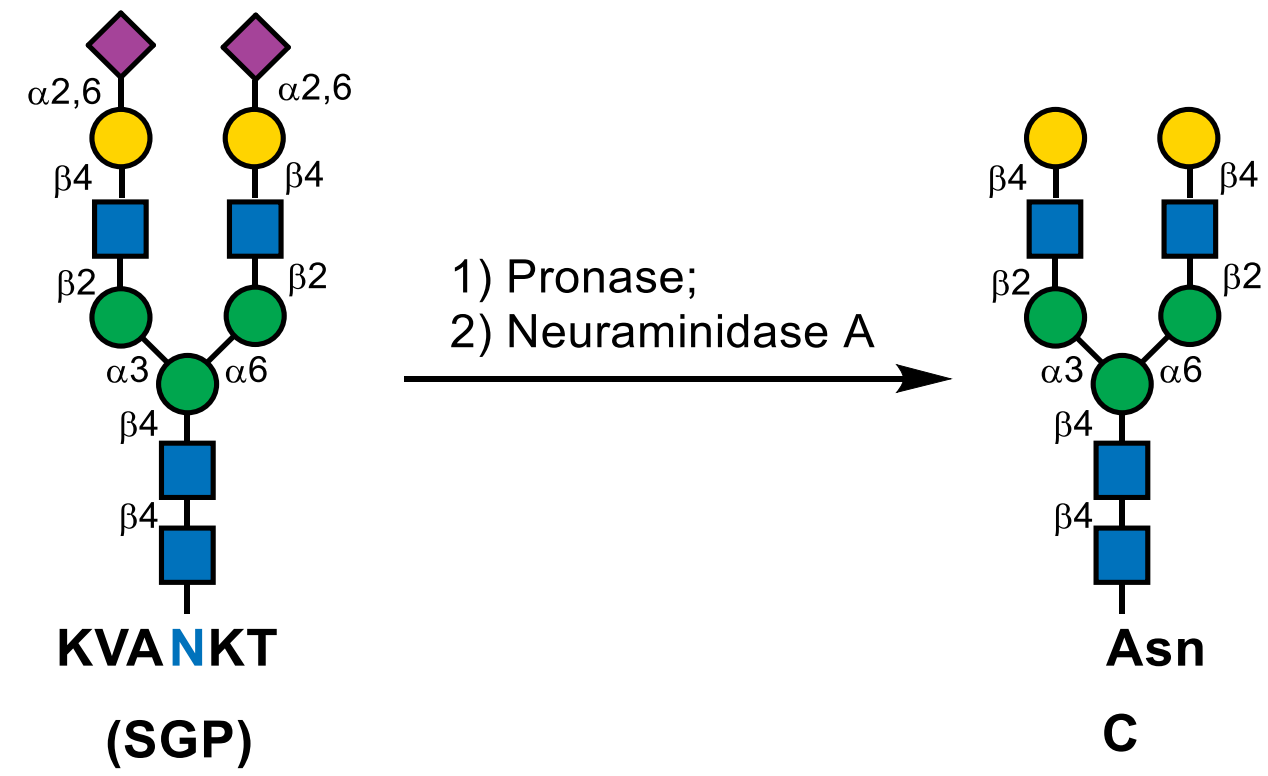

***N*^{4}-{β-D-galactopyranosyl-(1→4)-2-acetamido-2-deoxy-β-D-glucopyranosyl-(1→2)-α-D-mannopyranosyl-(1→3)-[β-D-galactopyranosyl-(1→4)-2-acetamido-2-deoxy-β-D-glucopyranosyl-(1→2)-α-D-mannopyranosyl-(1→6)]-β-D-mannopyranosyl-(1→4)-2-acetamido-2-deoxy-β-D-glucopyranosyl-(1→4)-2-acetamido-2-deoxy-β-D-glucopyranosyl}-L-asparagine (C)**: Sialylglycopeptide (SGP, 10.0 mg, 3.5 µmol, 1.0 equiv.) was digested sequentially with Pronase® (Sigma, from *Streptomyces griseus*) and α2-3,6,8,9 Neuraminidase A (New England Biolabs, from *Arthrobacter ureafaciens*) in Tris·HCl buffer (pH = 7.5) and the resulting solution was subjected onto a P2 column to afford the G2-glycan **C** (4.5 mg, 74% overall) as a fluffy solid after lyophilization. **C**: ^{1}H NMR (500 MHz, D_2O) δ = 5.04 (s, 1H), 4.99 (d, *J* = 9.7 Hz, 1H), 4.85 (d, *J* = 1.7 Hz, 1H), 4.69 (s, 1H), 4.54 (d, *J* = 7.9 Hz, 1H), 4.50 (d, *J* = 7.9 Hz, 2H), 4.43 – 4.36 (m, 2H), 4.17 (d, *J* = 2.8 Hz, 1H), 4.11 (dd, *J* = 3.4, 1.6 Hz, 1H), 4.03 (dd, *J* = 3.6, 1.6 Hz, 1H), 3.93 – 3.37 (m, 52H), 2.59 (dd, *J* = 15.3, 4.7 Hz, 1H), 2.33 (dd, *J* = 15.3, 8.8 Hz, 1H), 2.00 (s, 3H), 1.97 (s, 3H), 1.97 (s, 3H), 1.93 (s, 3H) ppm. LRMS (ESI-MS) *m*/*z* calculated for $C_{66}H_{110}N_6O_{46}^{2-}$ $[M-2H]^{2-}$: 876.3, found: 876.3

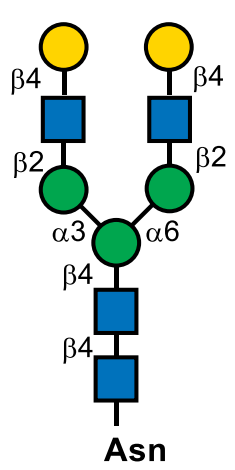

C

References

(1) Li, R.; Chen, P.; Zeng, Y. F.; Tseng, T. H.; Gannedi, V.; Krasnova, L.; Wong, C. H. Expedient Assembly of Multiantennary N-Glycans from Common N-Glycan Cores with Orthogonal Protection for the Profiling of Glycan-Binding Proteins. *J. Am. Chem. Soc.* **2025**, *147* (15), 12937–12948.
(2) Shivatare, S. S.; Chang, S. H.; Tsai, T. I.; Ren, C. T.; Chuang, H. Y.; Hsu, L.; Lin, C. W.; Li, S. T.; Wu, C. Y.; Wong, C. H. Efficient convergent synthesis of bi-, tri-, and tetra-antennary complex type N-glycans and their HIV-1 antigenicity. *J. Am. Chem. Soc.* **2013**, *135* (41), 15382–15391.
(3) Liao, H. Y.; Hsu, C. H.; Wang, S. C.; Liang, C. H.; Yen, H. Y.; Su, C. Y.; Chen, C. H.; Jan, J. T.; Ren, C. T.; Chen, C. H.; Cheng, T. J.; Wu, C. Y.; Wong, C. H. Differential receptor binding affinities of influenza hemagglutinins on glycan arrays. *J. Am. Chem. Soc.* **2010**, *132* (42), 14849–14856.
(4) Li, Y.; Yang, Y.; Yu, B. An efficient glycosylation protocol with glycosyl ortho-alkynylbenzoates as donors under the catalysis of $Ph_3PAuOTf$. *Tetrahedron Lett.* **2008**, *49* (22), 3604–3608.
(5) Donahue, T. C.; Ou, C.; Yang, Q.; Flinko, R.; Zhang, X.; Zong, G.; Lewis, G. K.; Wang, L. X. Synthetic Site-Specific Antibody-Ligand Conjugates Promote Asialoglycoprotein Receptor-Mediated Degradation of Extracellular Human PCSK9. *ACS Chem. Biol.* **2023**, *18* (7), 1611–1623.

^{1}H, ^{13}C and 2D-NMR Spectra

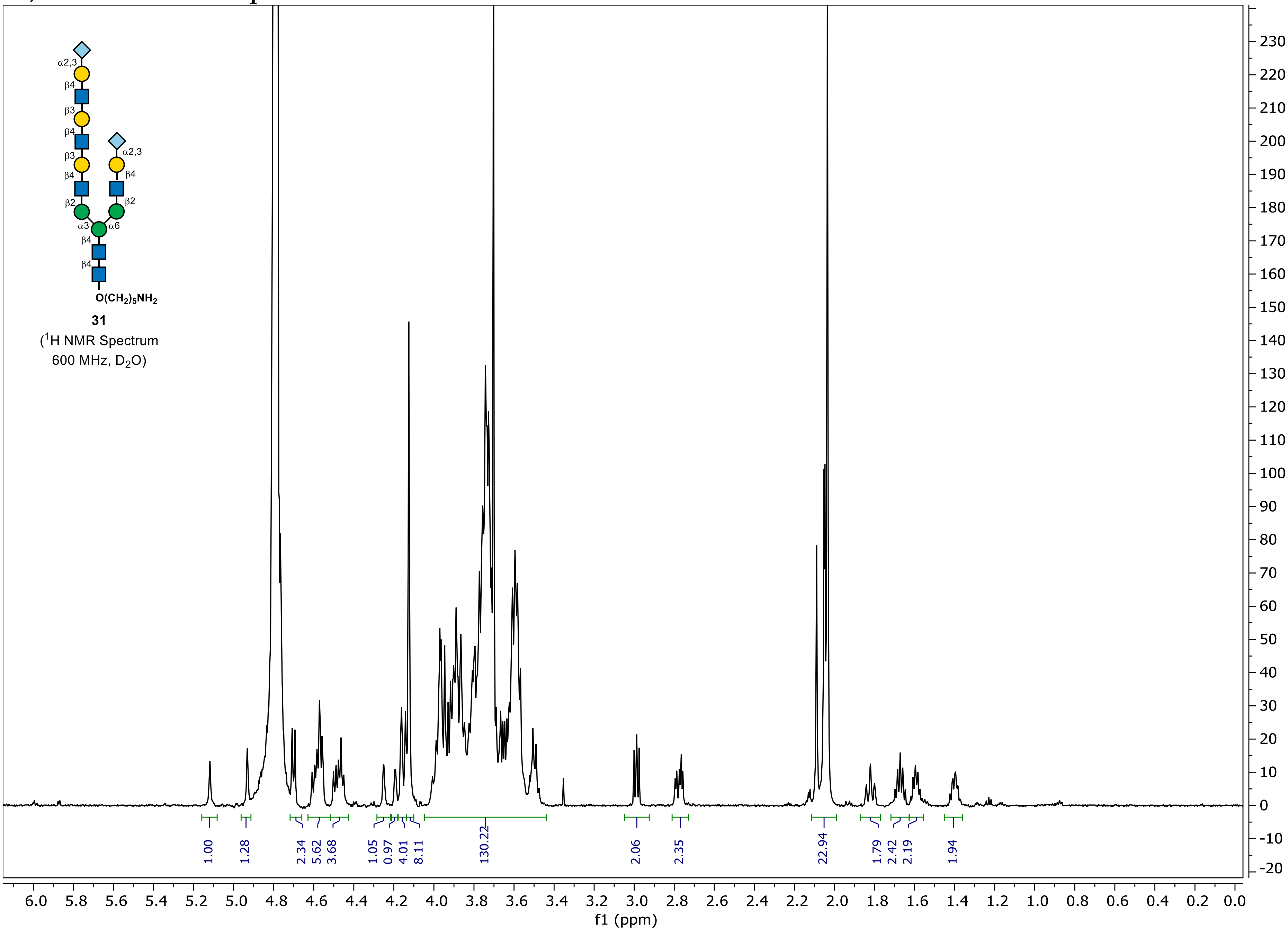

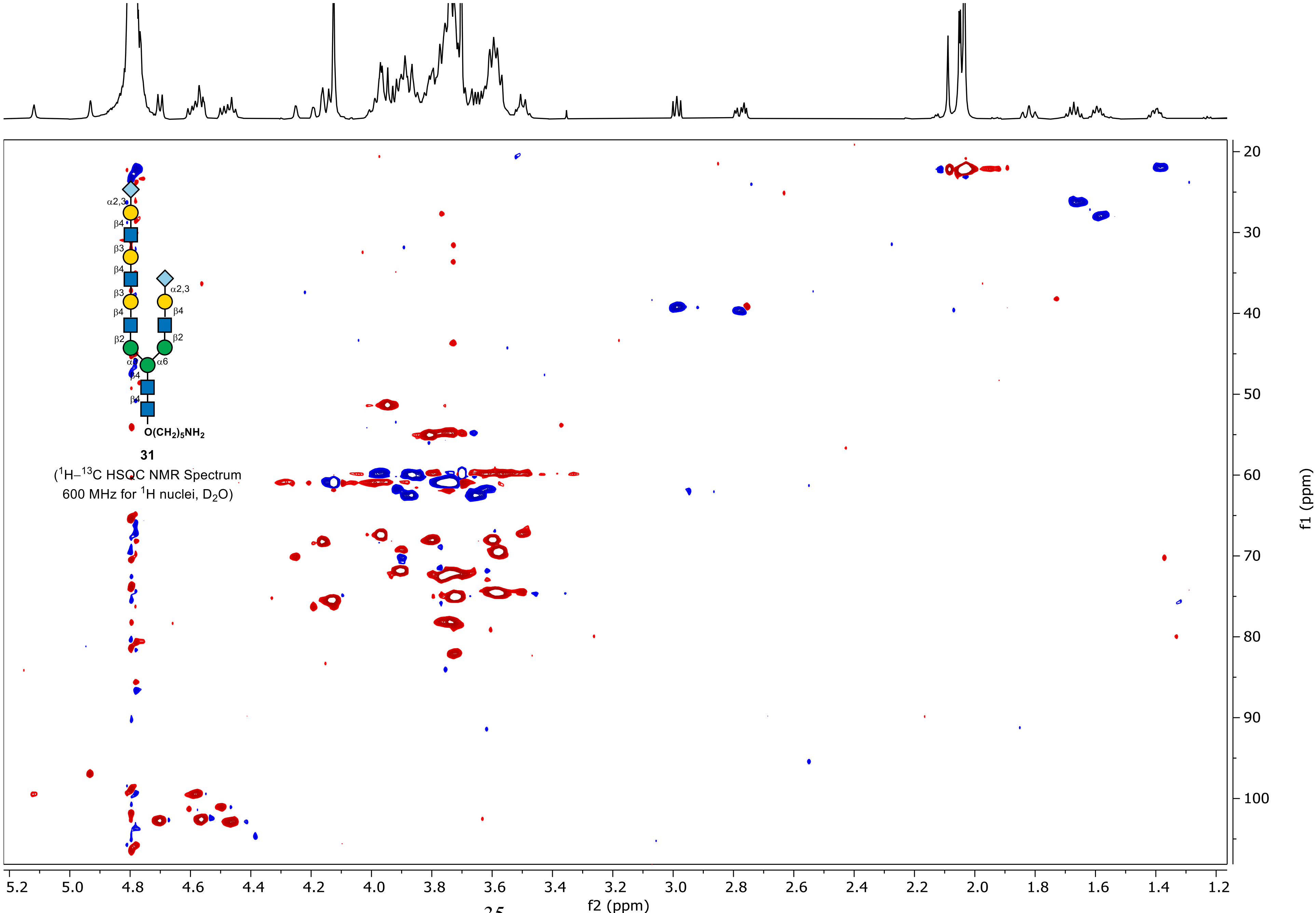
α2,3
β4
β3
β4
β3
β4
β2
α3
β4
β4
α2,3
β4
β2
α6
O(CH2)5NH2
31
(1H–13C HSQC NMR Spectrum
600 MHz for 1H nuclei, D2O)
f2 (ppm)
f1 (ppm)

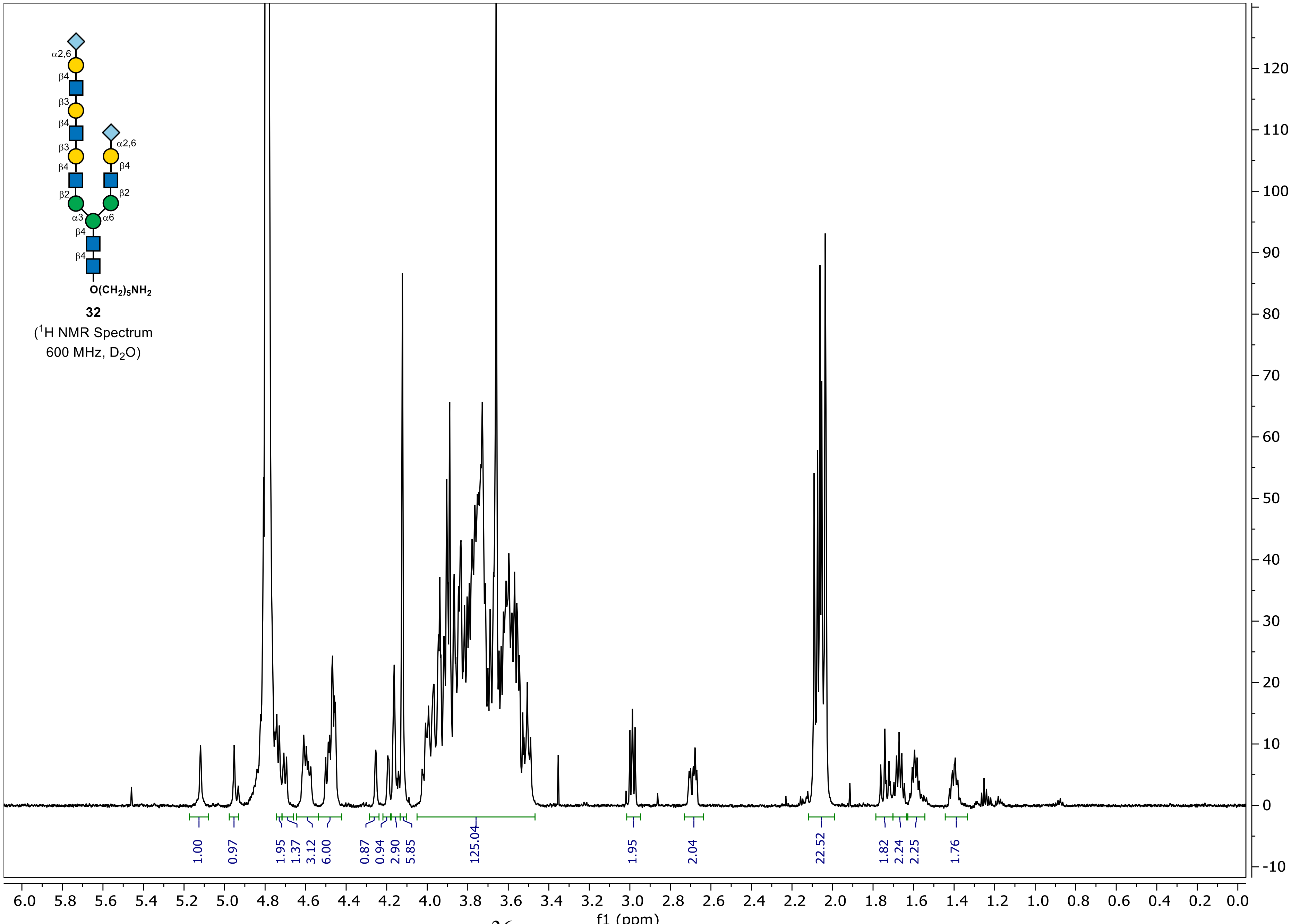
α2,6
β4
β3
β4
β3
β4
β2
α3
α6
β4
β4
α2,6
β4
β2
O(CH2)5NH2
32
(1H NMR Spectrum
600 MHz, D2O)
1.00
0.97
1.95
1.37
3.12
6.00
0.87
0.94
2.90
5.85
125.04
1.95
2.04
22.52
1.82
2.24
2.25
1.76
f1 (ppm)

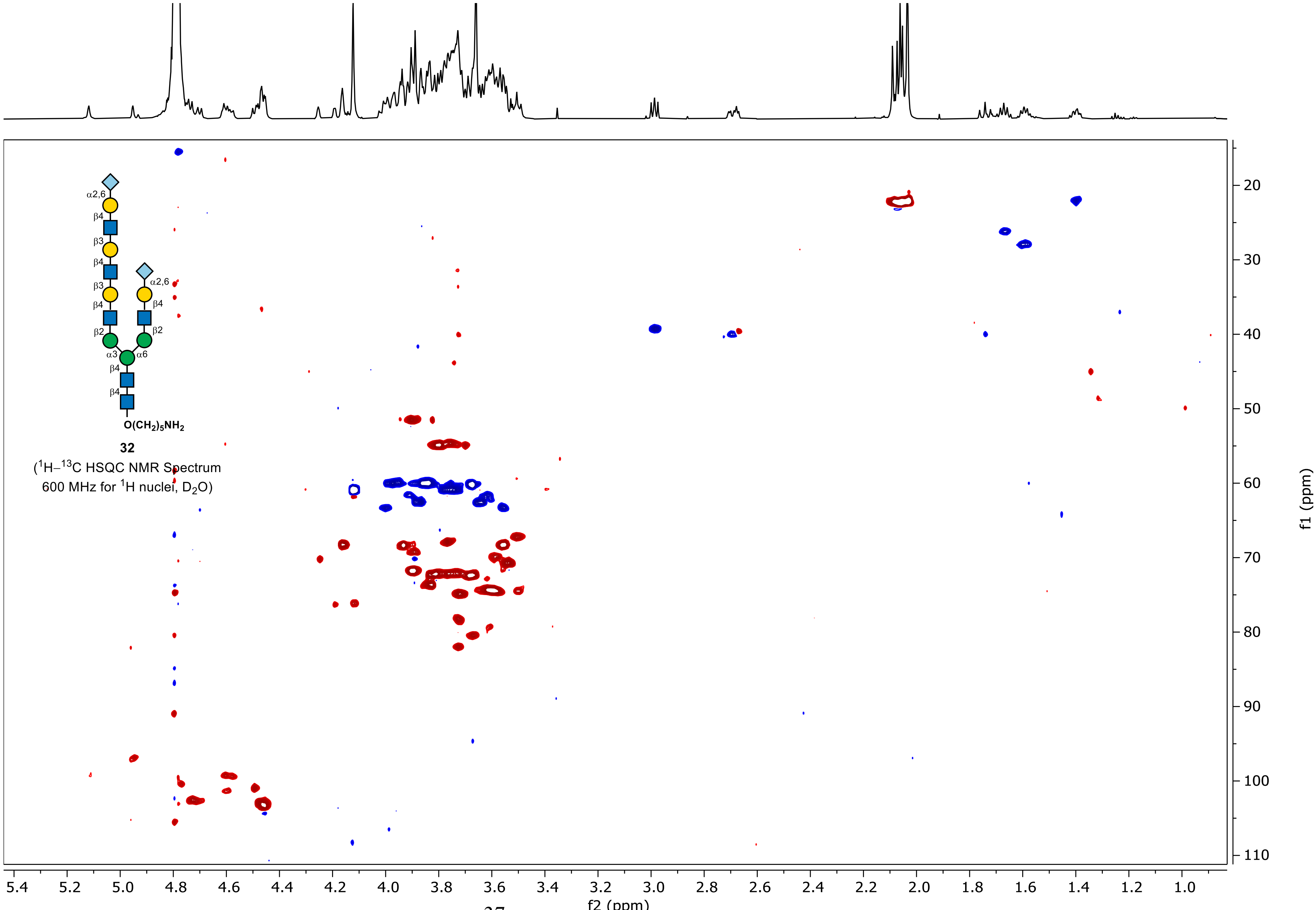
α2,6
β4
β3
β4
β3
β4
β2
α2,6
β4
β2
α3
α6
β4
β4
O(CH2)5NH2
32
(1H–13C HSQC NMR Spectrum
600 MHz for 1H nuclei, D2O)
f2 (ppm)
f1 (ppm)

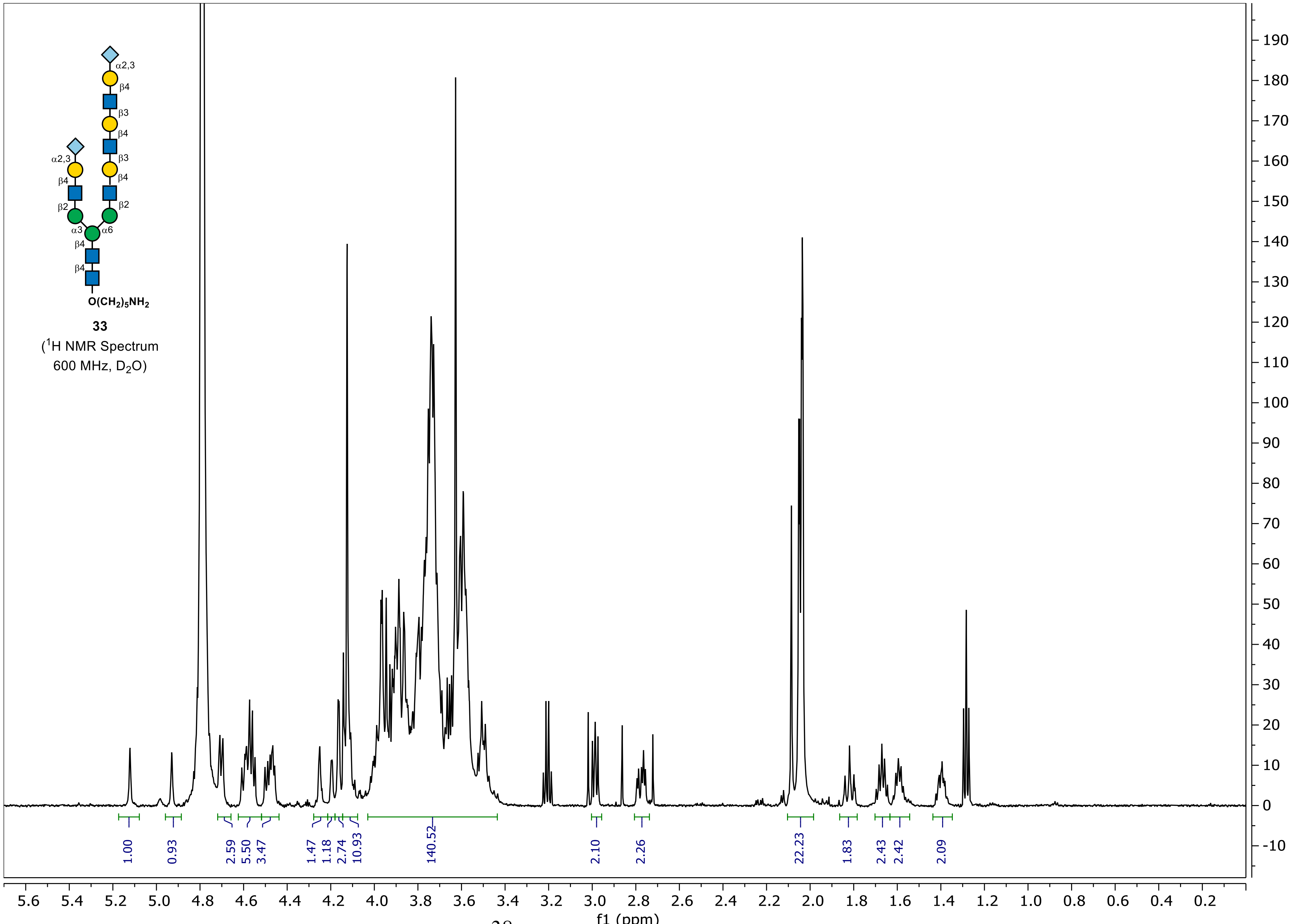
α2,3
β4
β3
β4
β3
β4
β2
α2,3
β4
β2
α3
α6
β4
β4
O(CH2)5NH2
33
(1H NMR Spectrum
600 MHz, D2O)
1.00
0.93
2.59
5.50
3.47
1.47
1.18
2.74
10.93
140.52
2.10
2.26
22.23
1.83
2.43
2.42
2.09
f1 (ppm)

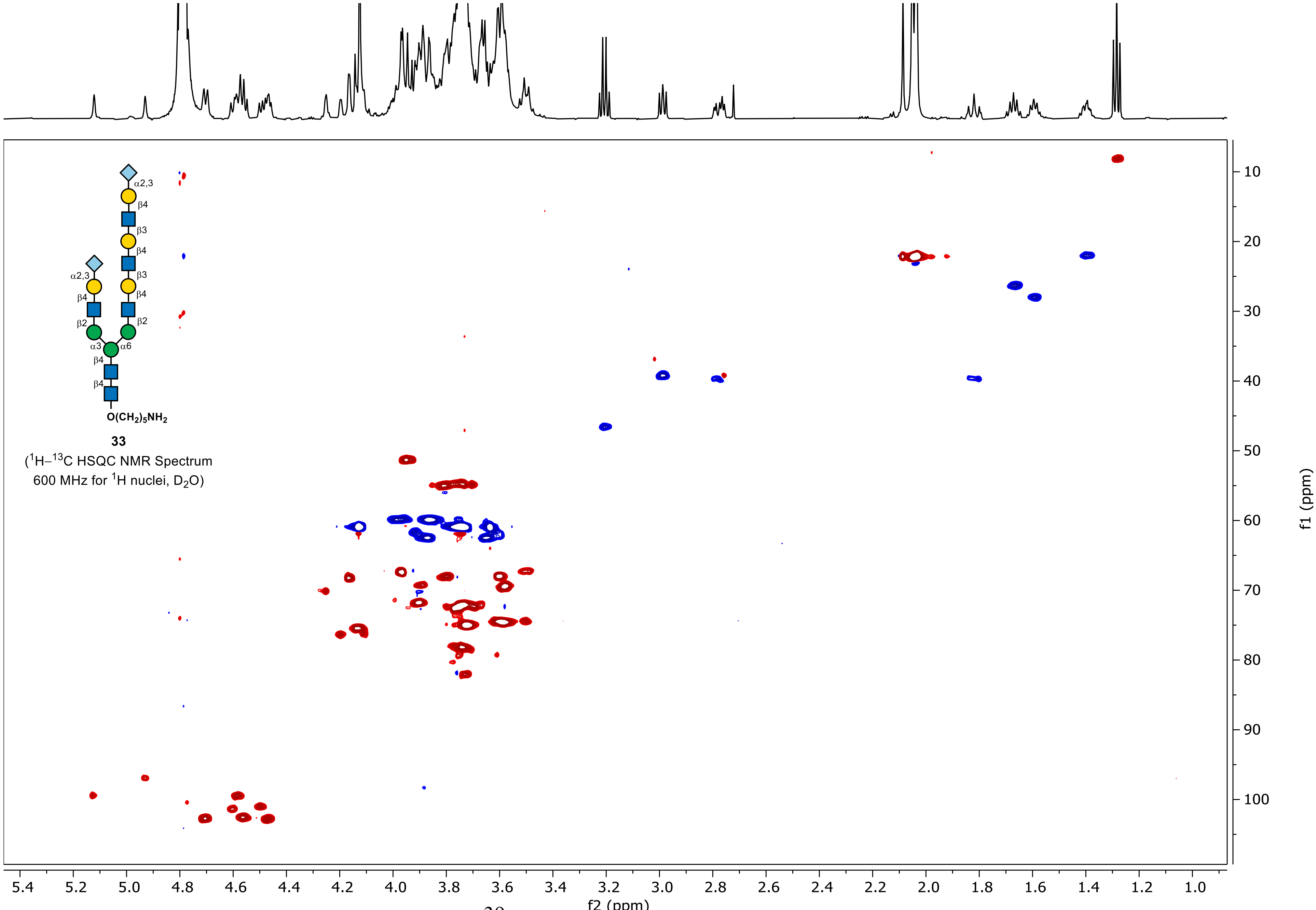

33

(^{1}H–^{13}C HSQC NMR Spectrum
600 MHz for ^{1}H nuclei, D_2O)

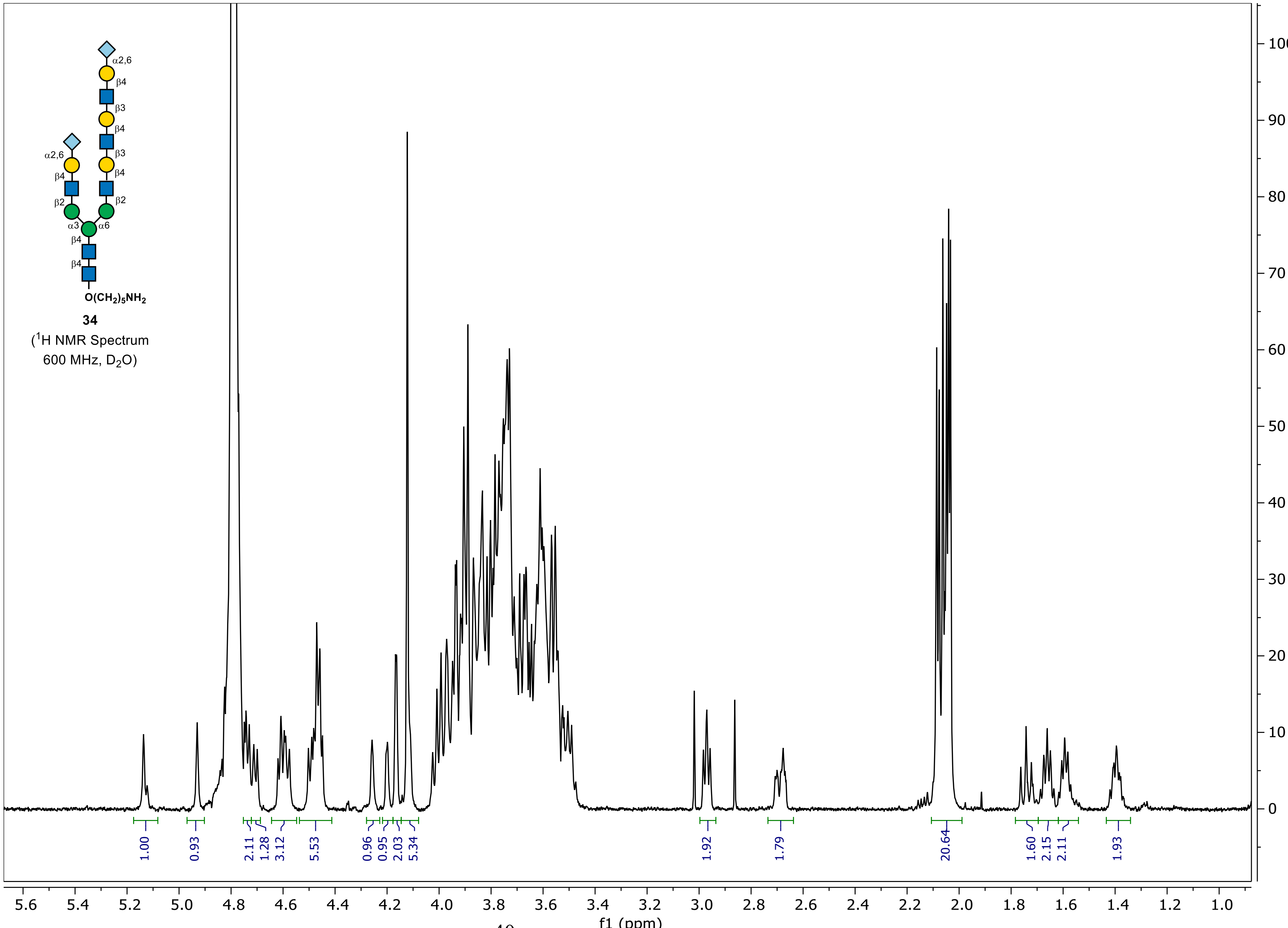
α2,6
β4
β3
β4
β3
β4
β2
α2,6
β4
β2
α3
α6
β4
β4
O(CH2)5NH2
34
(1H NMR Spectrum
600 MHz, D2O)
1.00
0.93
2.11
1.28
3.12
5.53
0.96
0.95
2.03
5.34
1.92
1.79
20.64
1.60
2.15
2.11
1.93
f1 (ppm)

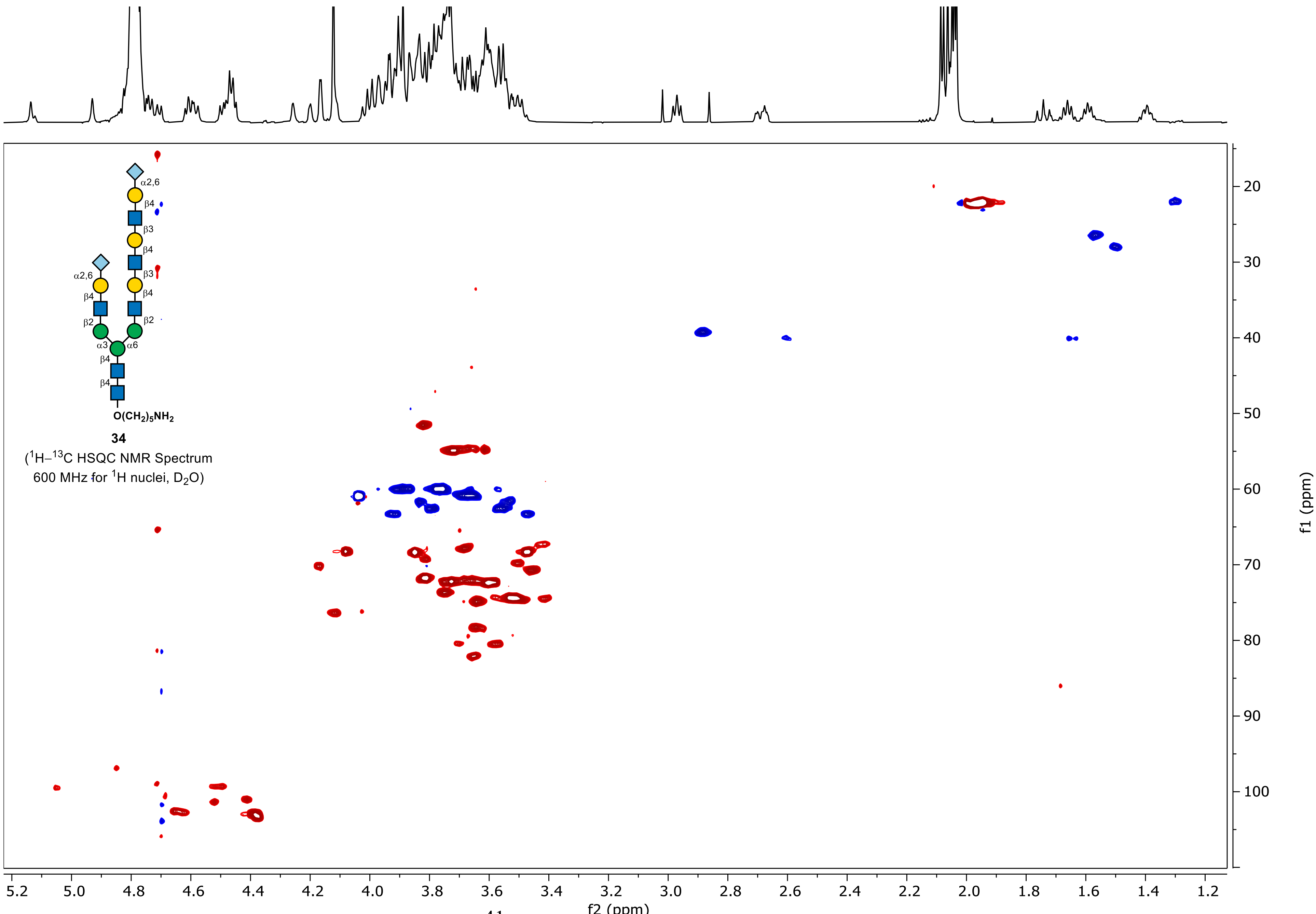

α2,6
β4
β3
β4
β3
β4
β2
α2,6
β4
β2
α3
α6
β4
β4
O(CH2)5NH2
34
(1H–13C HSQC NMR Spectrum
600 MHz for 1H nuclei, D2O)
f1 (ppm)
f2 (ppm)

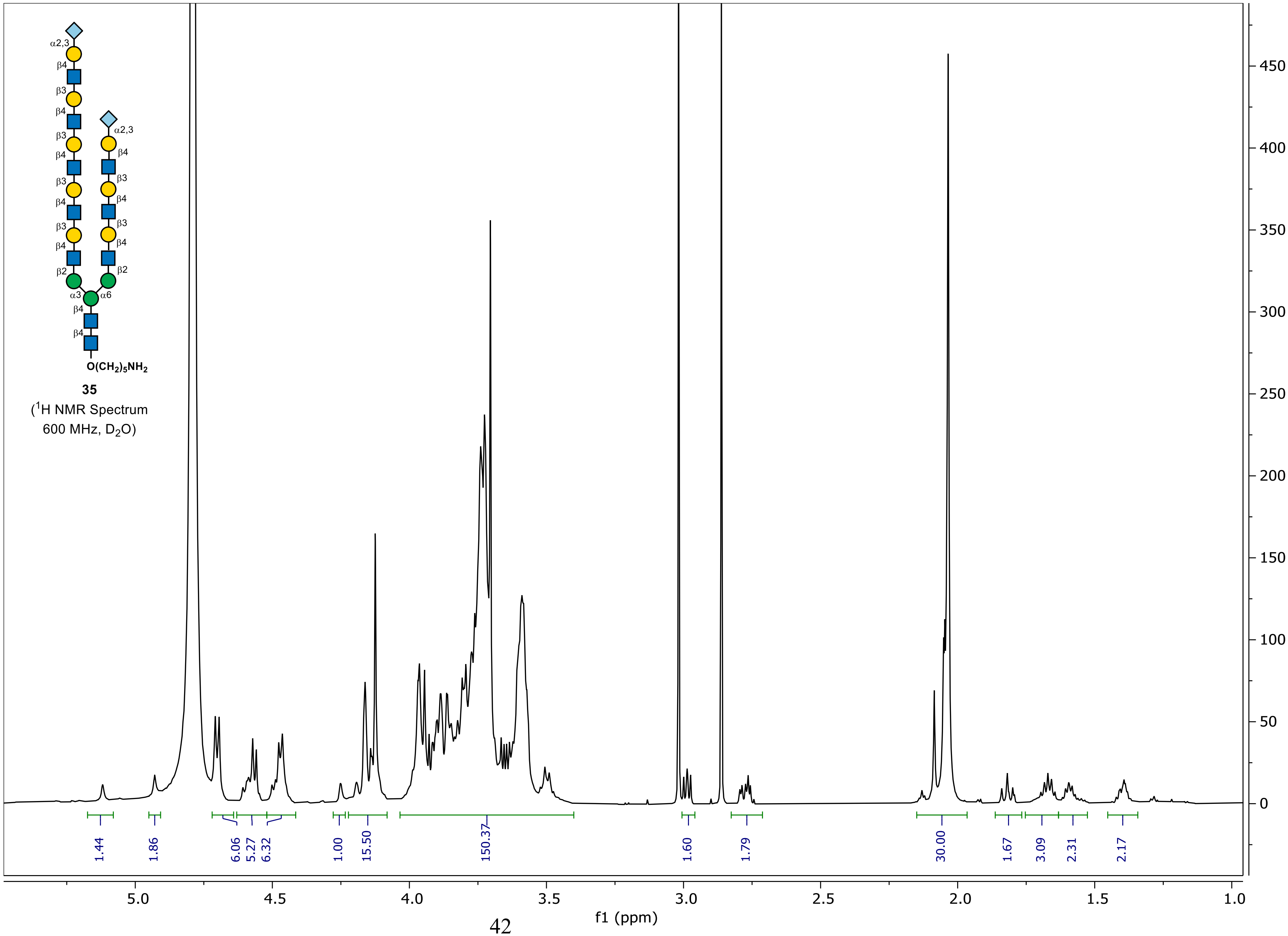
α2,3
β4
β3
O(CH2)5NH2
35
(1H NMR Spectrum
600 MHz, D2O)
1.44
1.86
6.06
5.27
6.32
1.00
15.50
150.37
1.60
1.79
30.00
1.67
3.09
2.31
2.17
f1 (ppm)

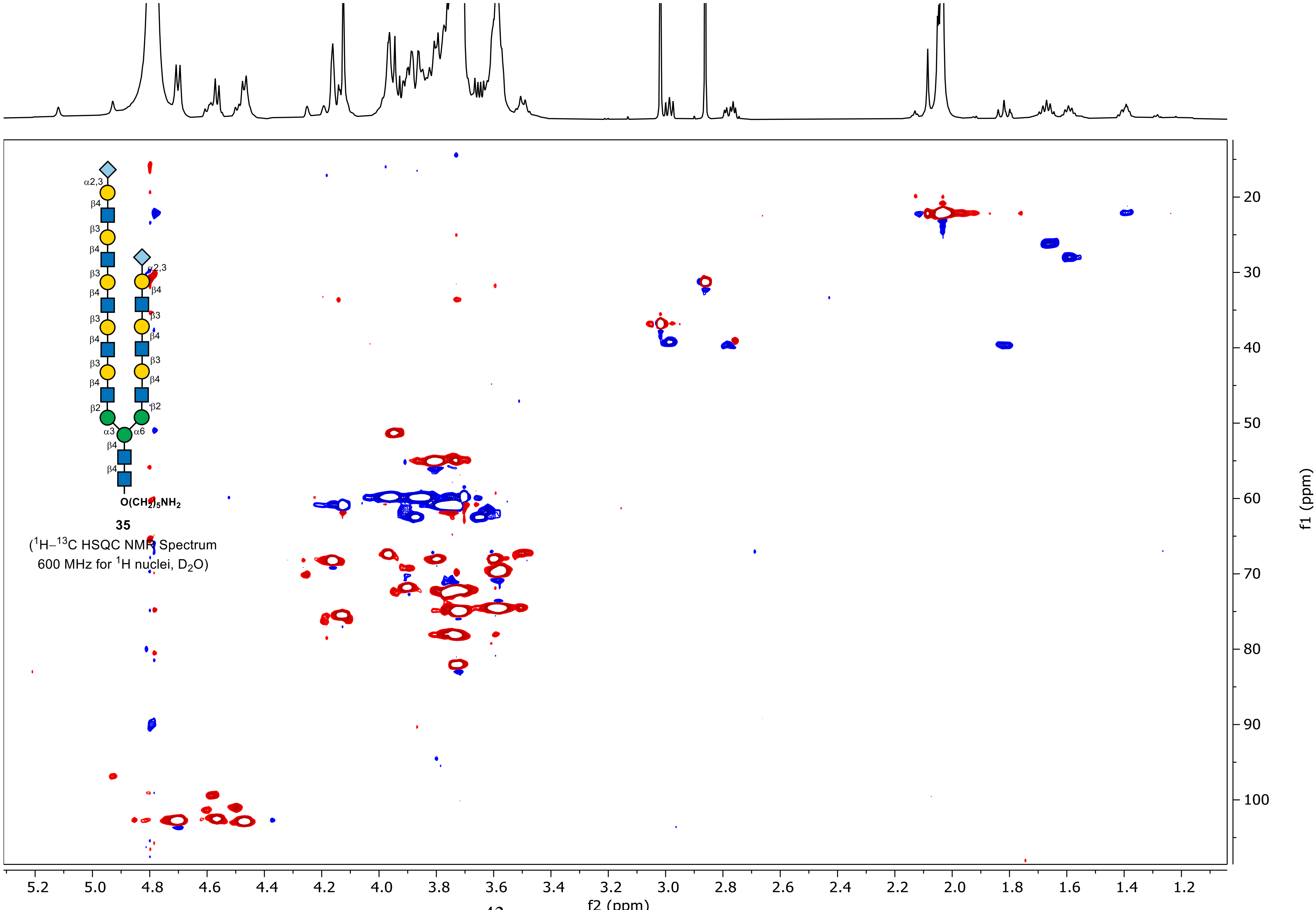
35
(¹H–¹³C HSQC NMR Spectrum
600 MHz for ¹H nuclei, D₂O)
O(CH₂)₅NH₂
f2 (ppm)
f1 (ppm)

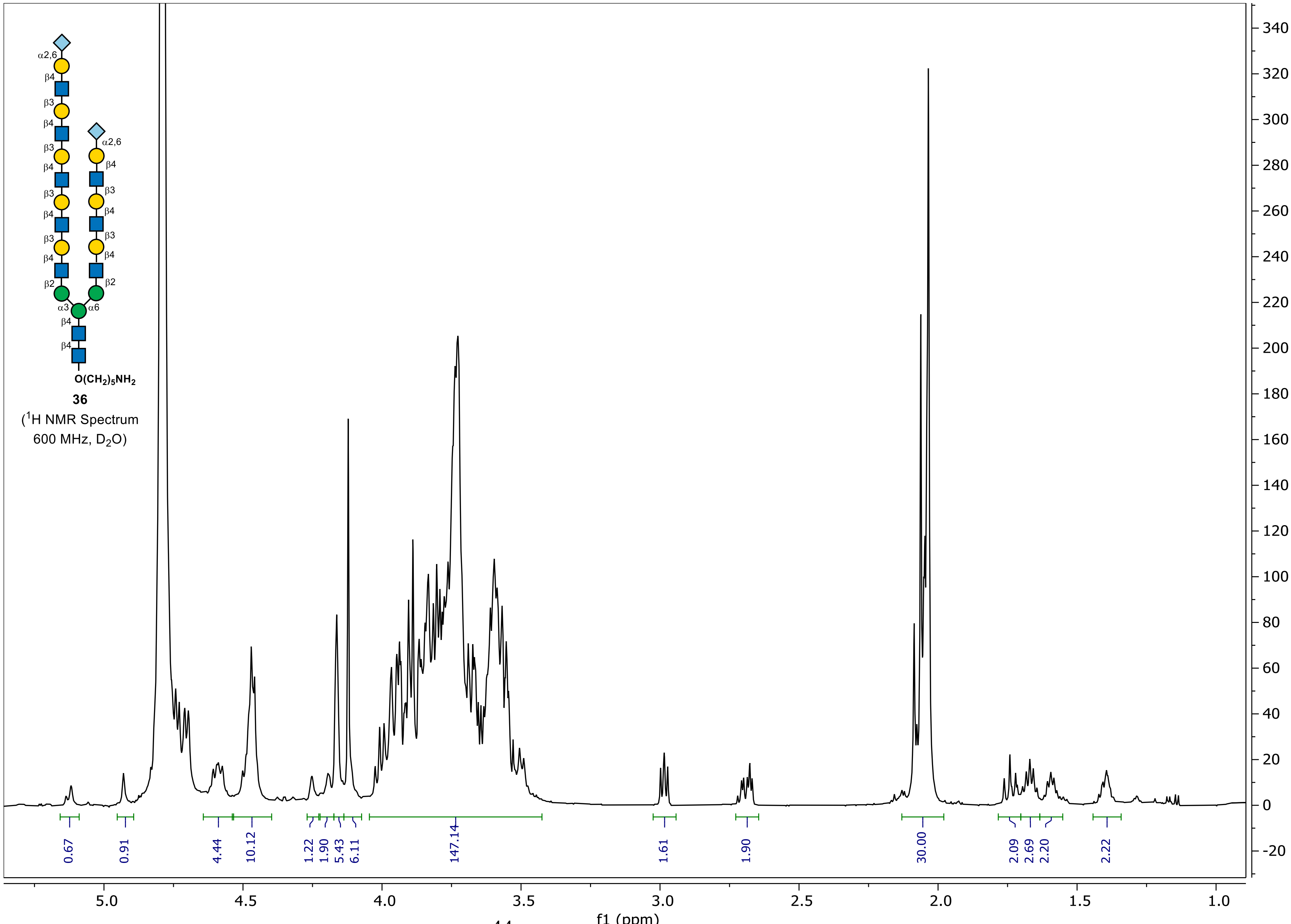
36
(¹H NMR Spectrum
600 MHz, D2O)
O(CH2)5NH2
f1 (ppm)

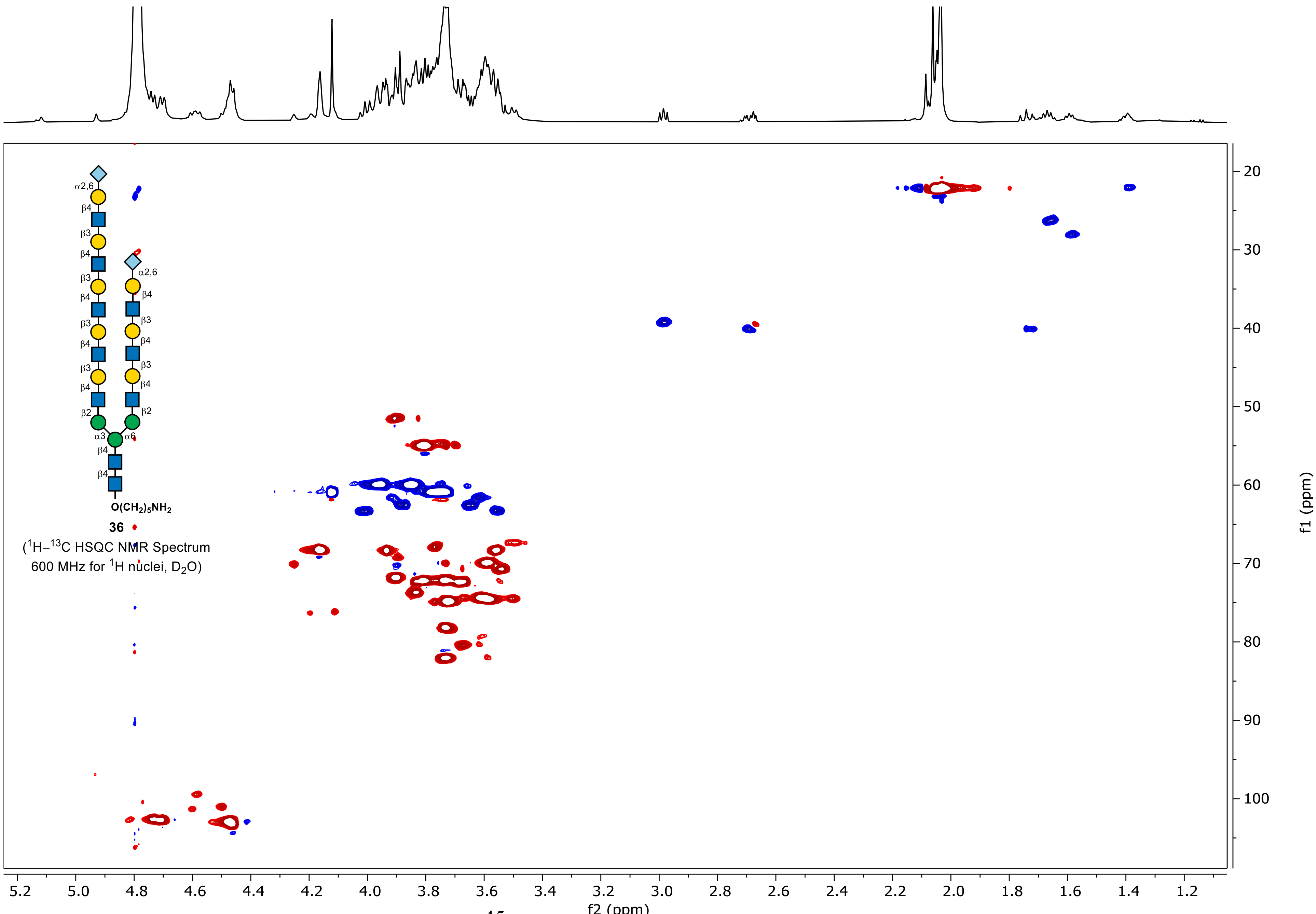
α2,6
β4
β3
β4
β3
β4
β3
β4
β3
β4
β2
α3
α6
β4
β4
O(CH2)5NH2
36
(1H–13C HSQC NMR Spectrum
600 MHz for 1H nuclei, D2O)
f2 (ppm)
f1 (ppm)

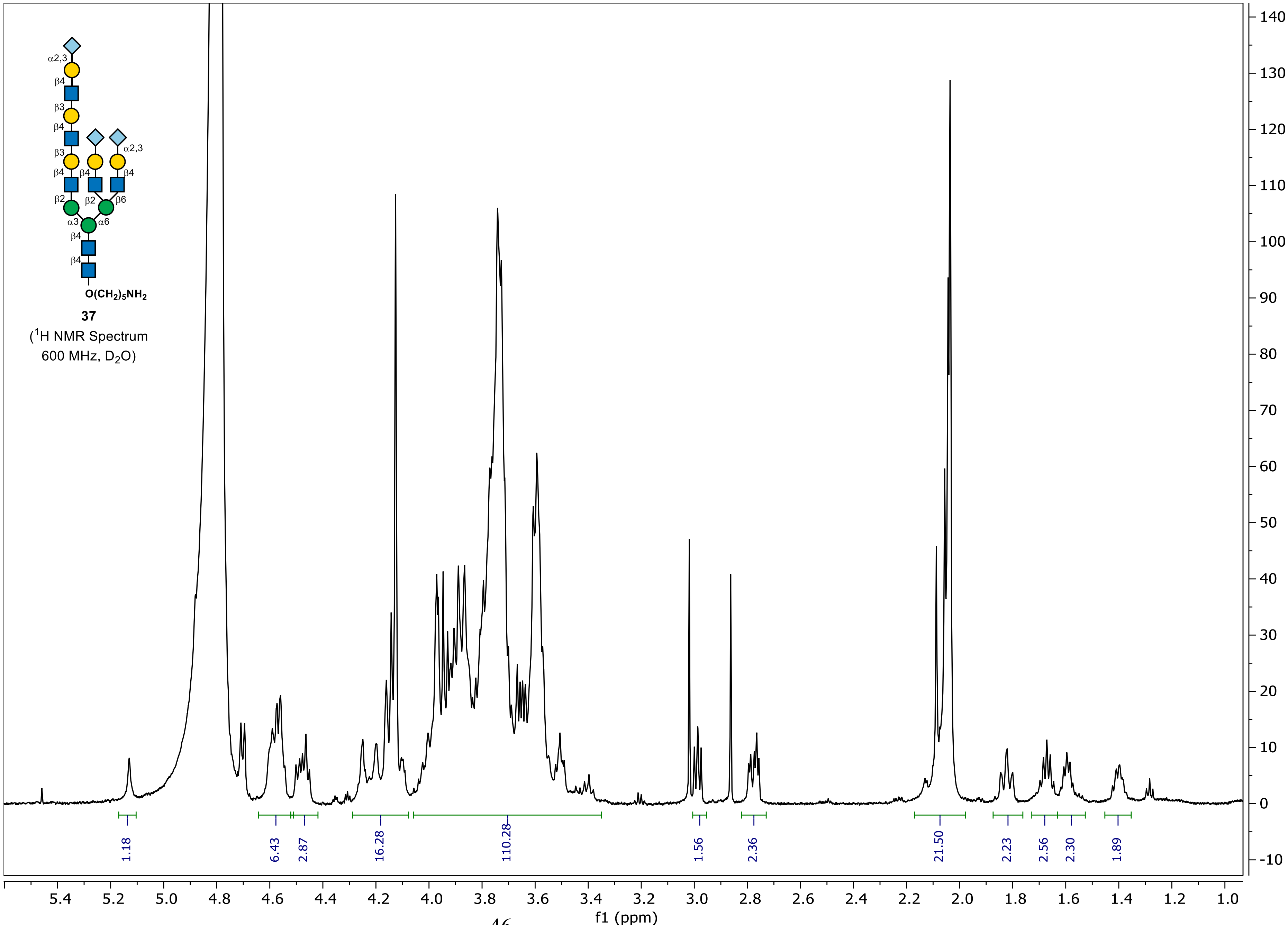

α2,3
β4
β3
β4
β3
β4
β2
α2,3
β4
β4
β2
β6
α3
α6
β4
β4
O(CH2)5NH2
37
(1H NMR Spectrum
600 MHz, D2O)
1.18
6.43
2.87
16.28
110.28
1.56
2.36
21.50
2.23
2.56
2.30
1.89
f1 (ppm)

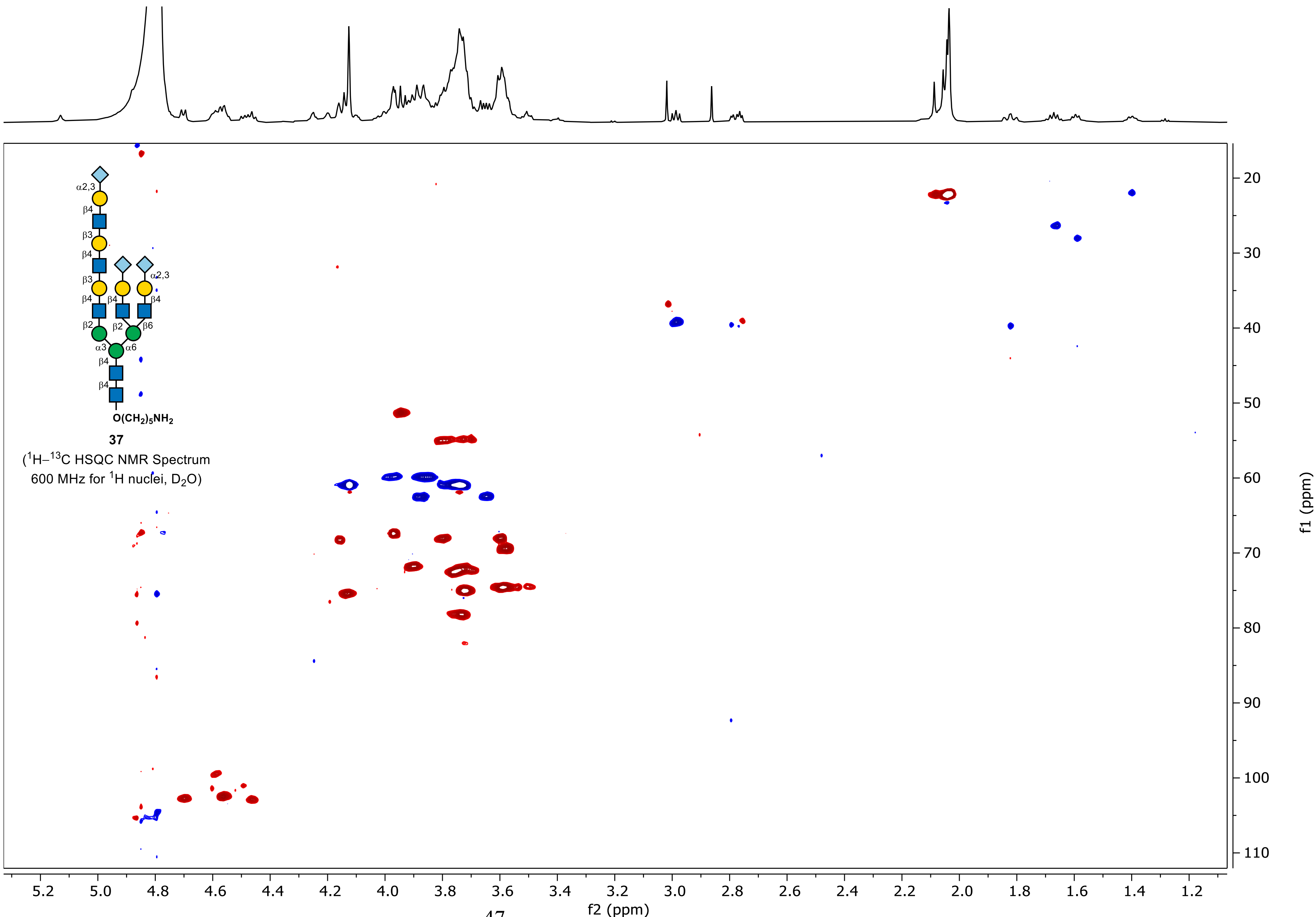
α2,3
β4
β3
β4
β3
β4
β2
β4
β4
α2,3
β2
β6
α3
α6
β4
β4
O(CH2)5NH2
37
(1H–13C HSQC NMR Spectrum
600 MHz for 1H nuclei, D2O)
f1 (ppm)
f2 (ppm)

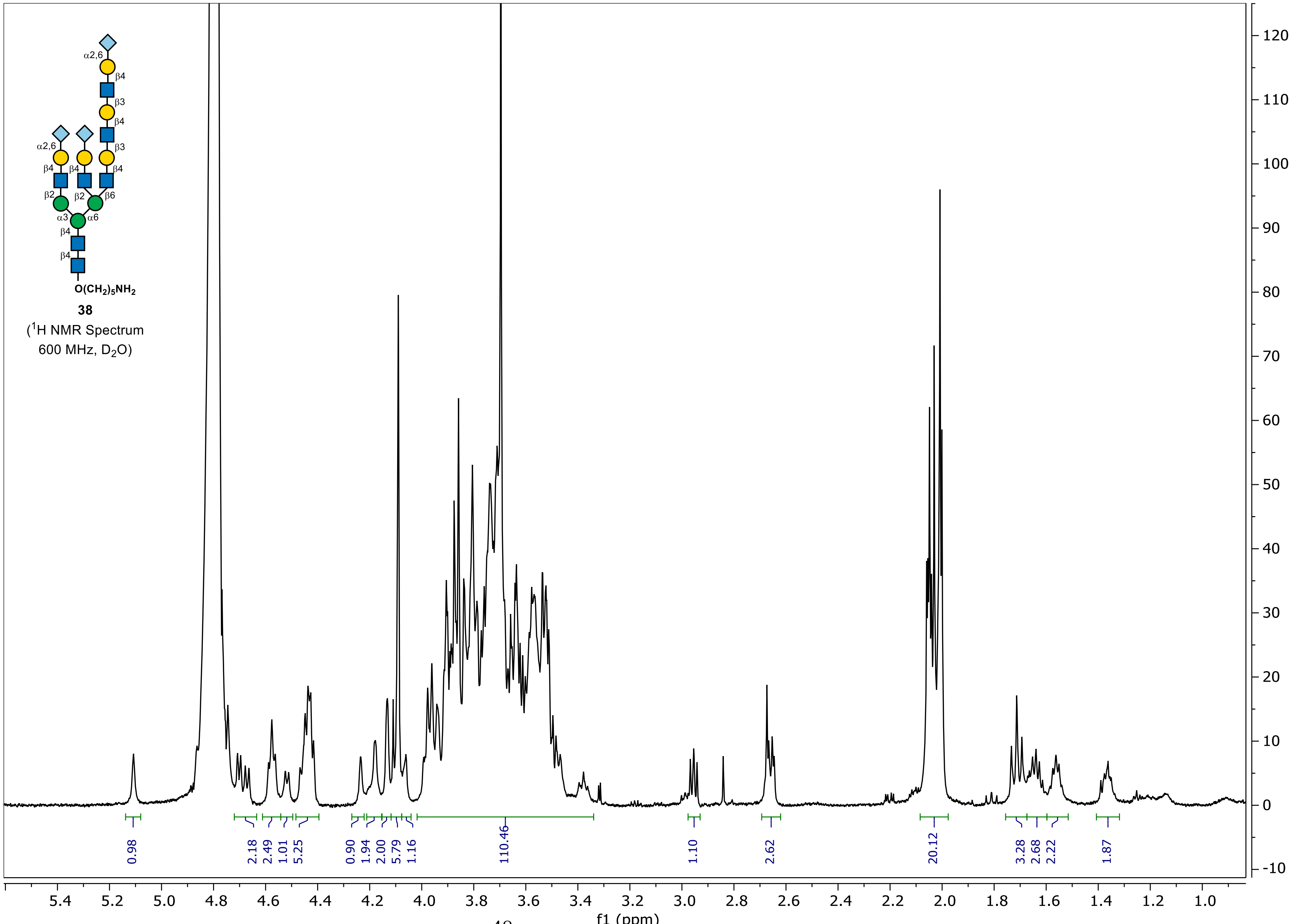
α2,6
β4
β3
β4
β3
β4
α2,6
β4
β4
β4
β2
β2
β6
α3
α6
β4
β4
O(CH2)5NH2
38
(1H NMR Spectrum
600 MHz, D2O)
0.98
2.18
2.49
1.01
5.25
0.90
1.94
2.00
5.79
1.16
110.46
1.10
2.62
20.12
3.28
2.68
2.22
1.87
f1 (ppm)

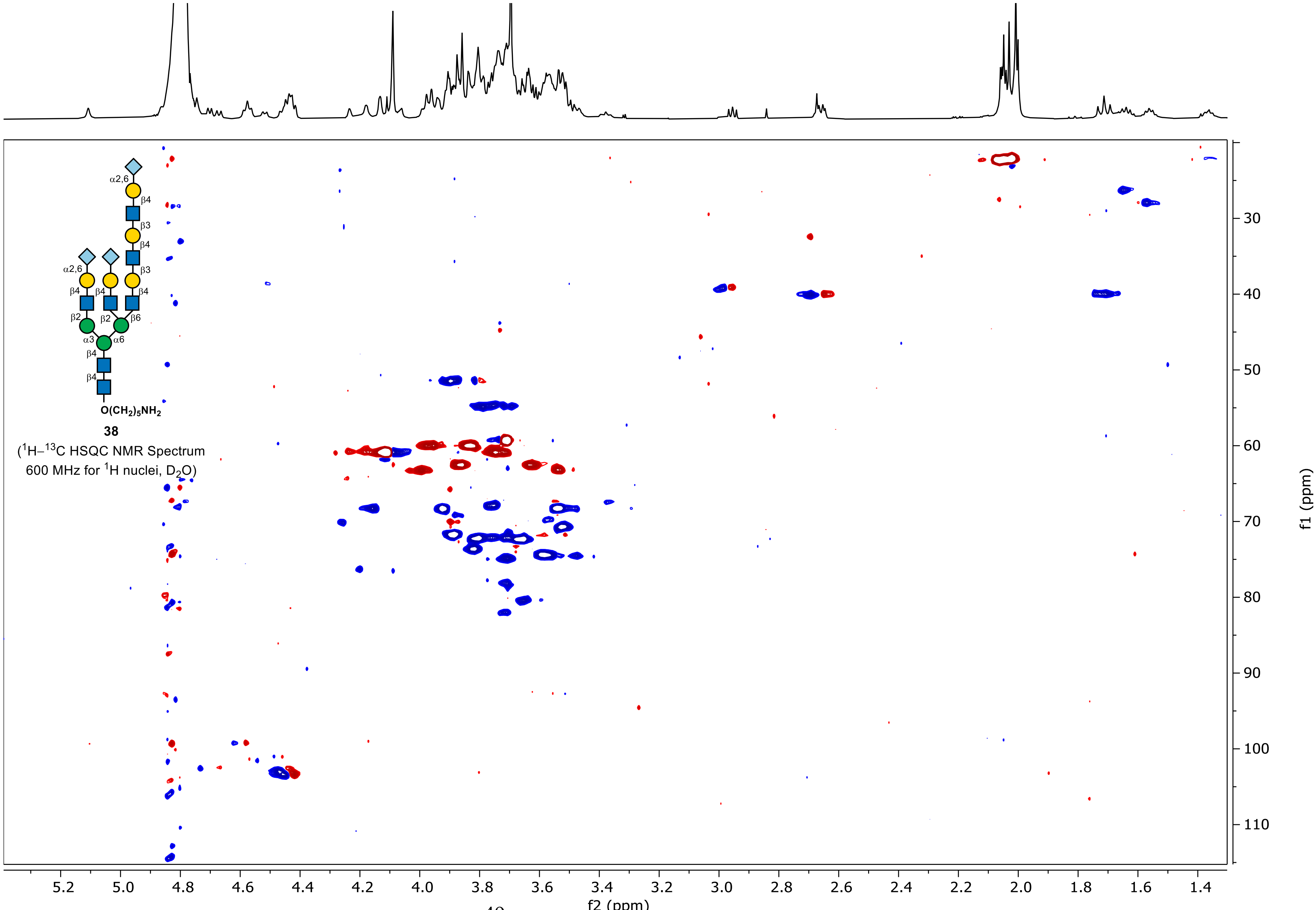
α2,6
β4
β3
β4
β3
α2,6
β4
β4
β4
β2
β2
β6
α3
α6
β4
β4
O(CH2)5NH2
38
(1H–13C HSQC NMR Spectrum
600 MHz for 1H nuclei, D2O)
f2 (ppm)
f1 (ppm)

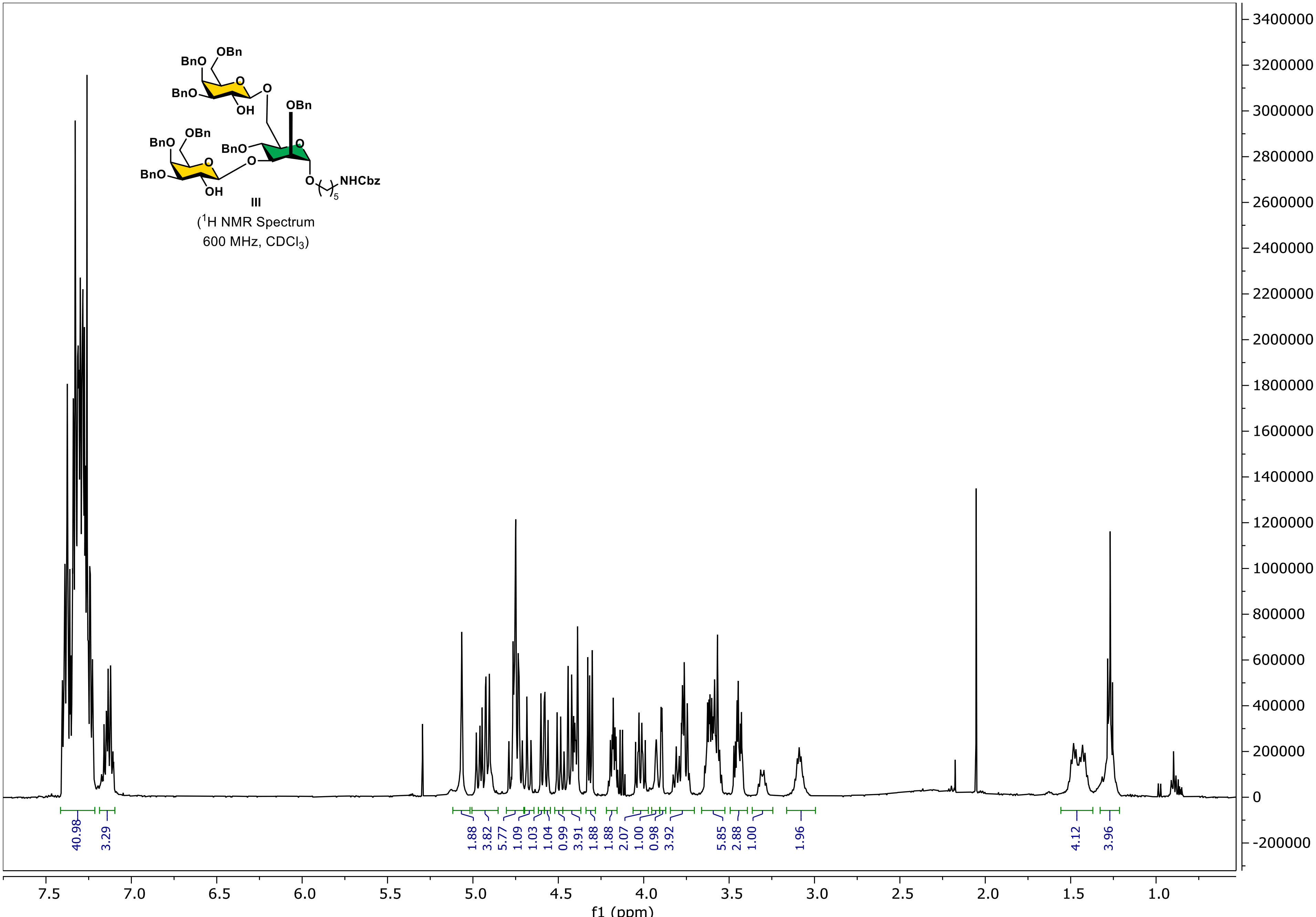
III
(1H NMR Spectrum
600 MHz, CDCl3)
f1 (ppm)

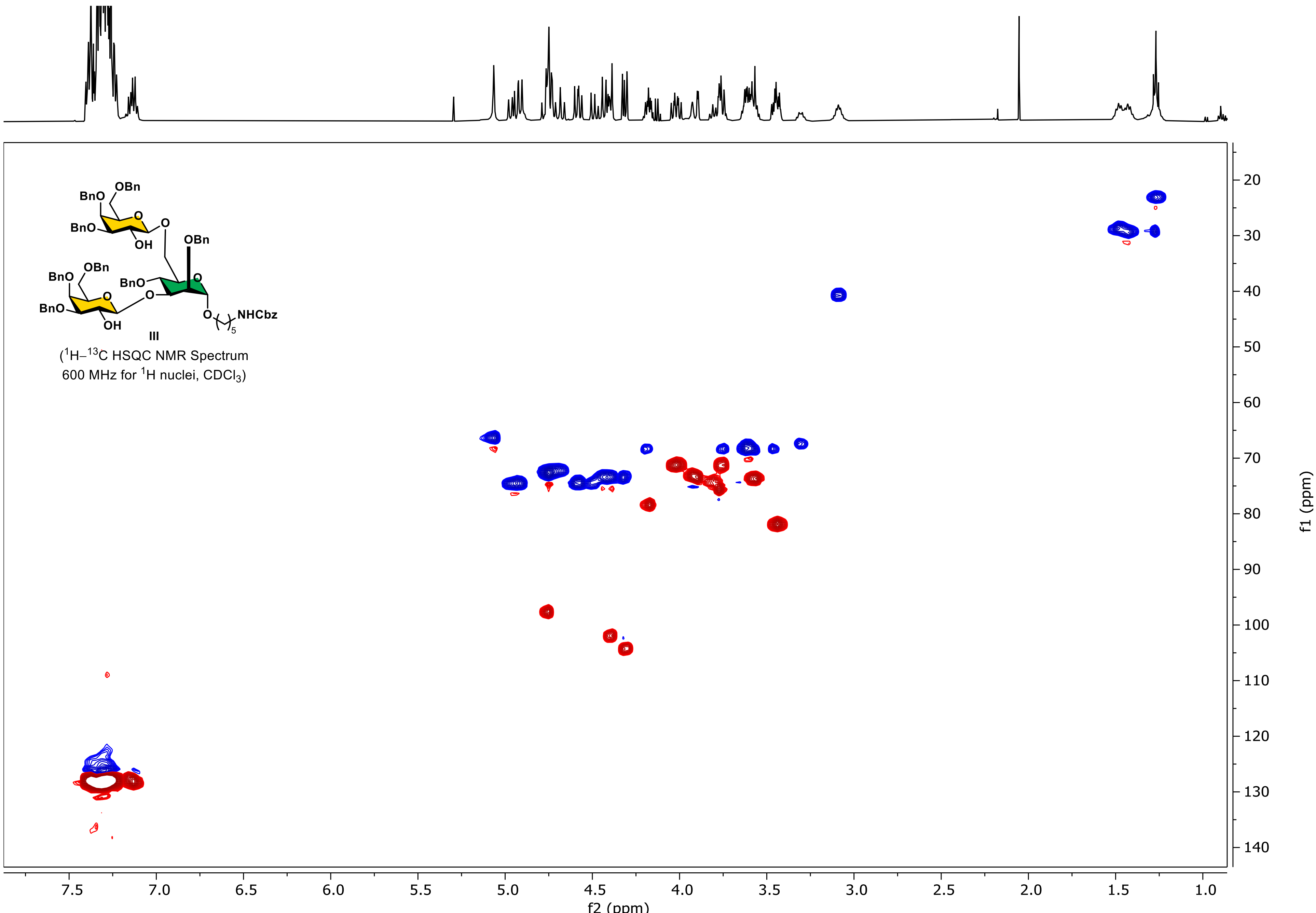

OBn
BnO
BnO
O
OH
O
OBn
OBn
BnO
BnO
O
O
BnO
O
BnO
O
OH
O
NHCbz
5
III
(¹H–¹³C HSQC NMR Spectrum
600 MHz for ¹H nuclei, CDCl₃)
f1 (ppm)
20
30
40
50
60
70
80
90
100
110
120
130
140
7.5
7.0
6.5
6.0
5.5
5.0
4.5
4.0
3.5
3.0
2.5
2.0
1.5
1.0
f2 (ppm)

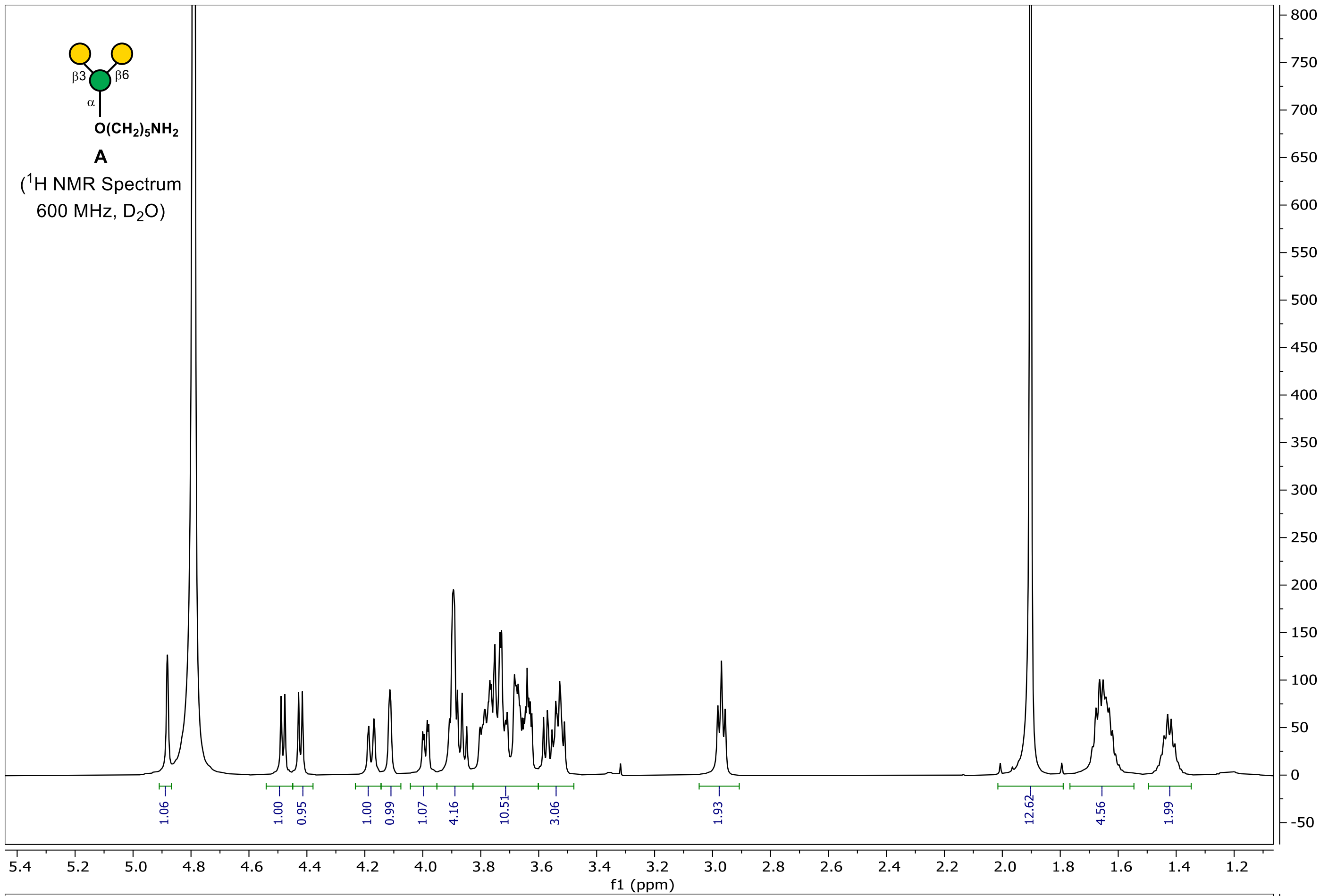
β3
β6
α
O(CH2)5NH2
A
(1H NMR Spectrum
600 MHz, D2O)
1.06
1.00
0.95
1.00
0.99
1.07
4.16
10.51
3.06
1.93
12.62
4.56
1.99
f1 (ppm)

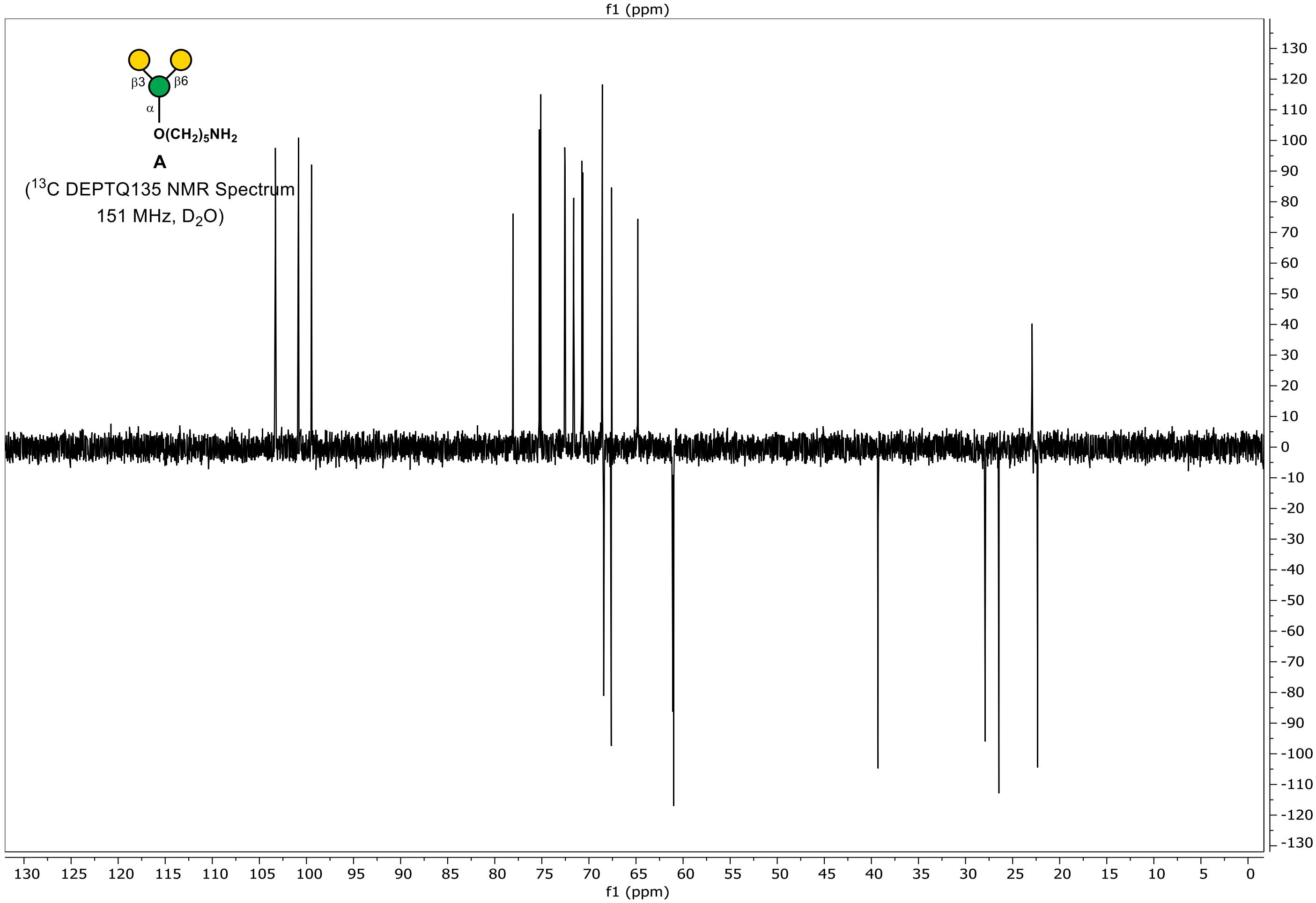
β3
β6
α
O(CH2)5NH2
A
(13C DEPTQ135 NMR Spectrum
151 MHz, D2O)
f1 (ppm)

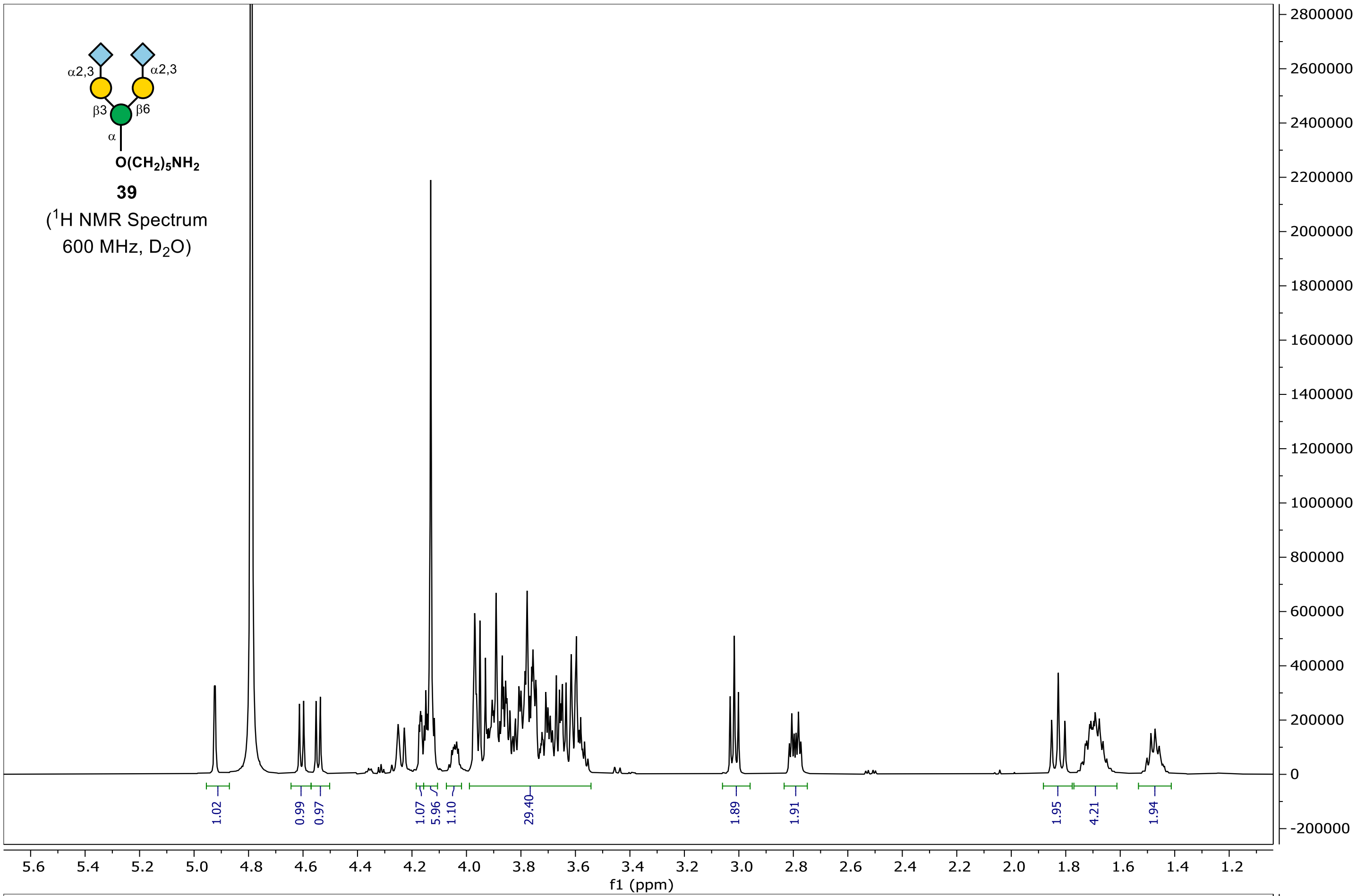
α2,3
α2,3
β3
β6
α
O(CH2)5NH2
39
(1H NMR Spectrum
600 MHz, D2O)
1.02
0.99
0.97
1.07
5.96
1.10
29.40
1.89
1.91
1.95
4.21
1.94
f1 (ppm)

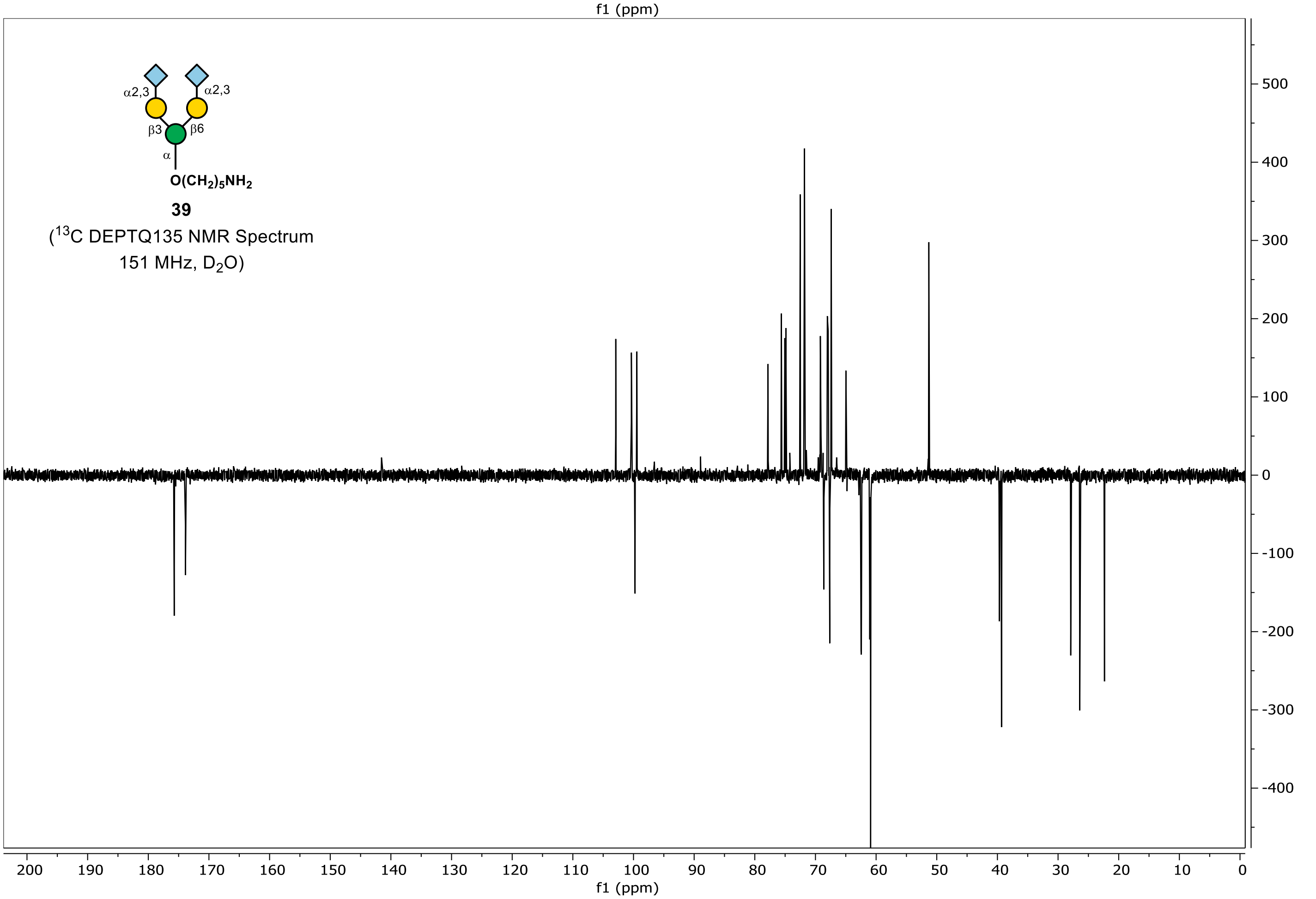
α2,3
α2,3
β3
β6
α
O(CH2)5NH2
39
(13C DEPTQ135 NMR Spectrum
151 MHz, D2O)
f1 (ppm)

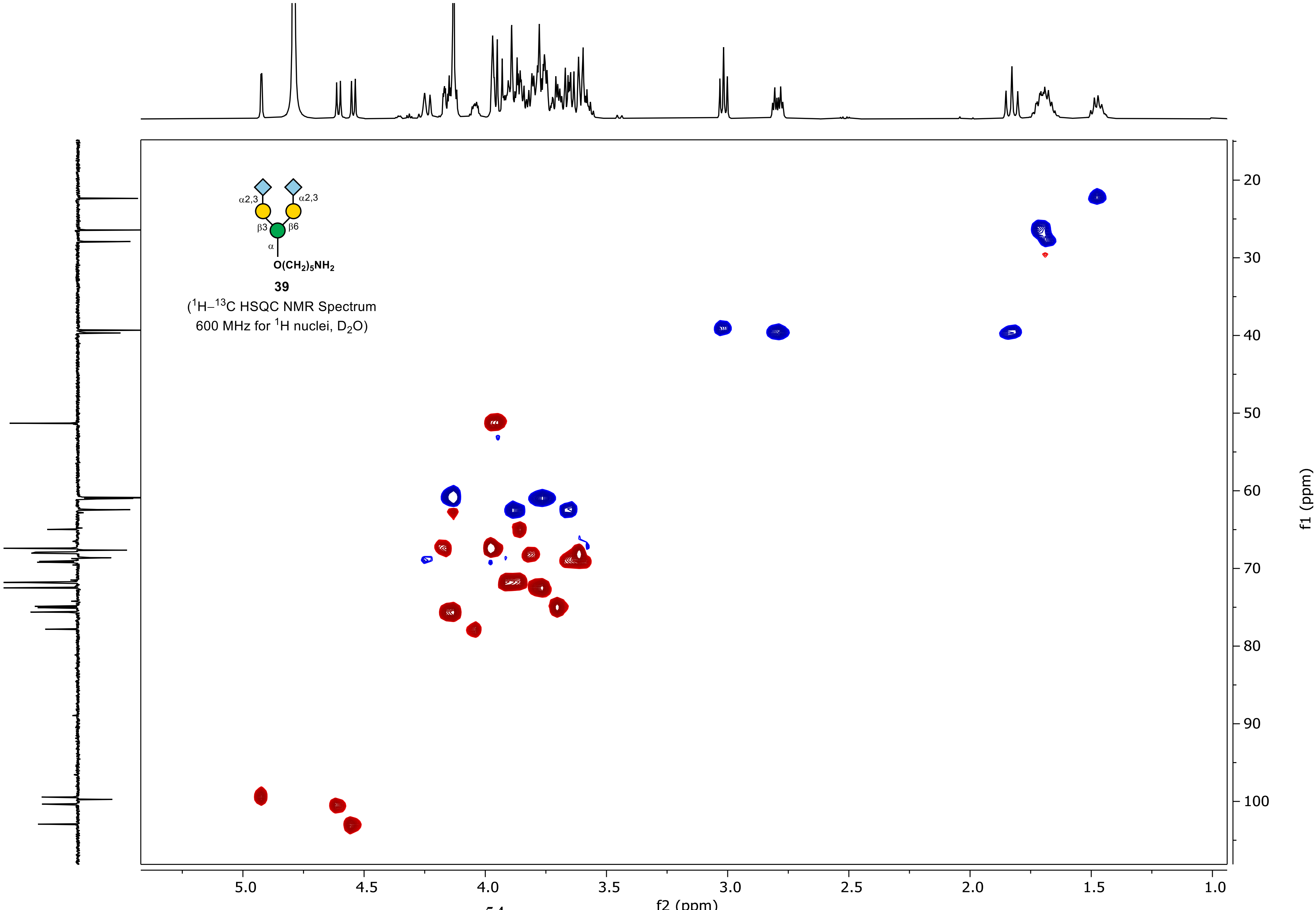

α2,3
α2,3
β3
β6
α
O(CH2)5NH2
39
(1H–13C HSQC NMR Spectrum
600 MHz for 1H nuclei, D2O)
f2 (ppm)
f1 (ppm)

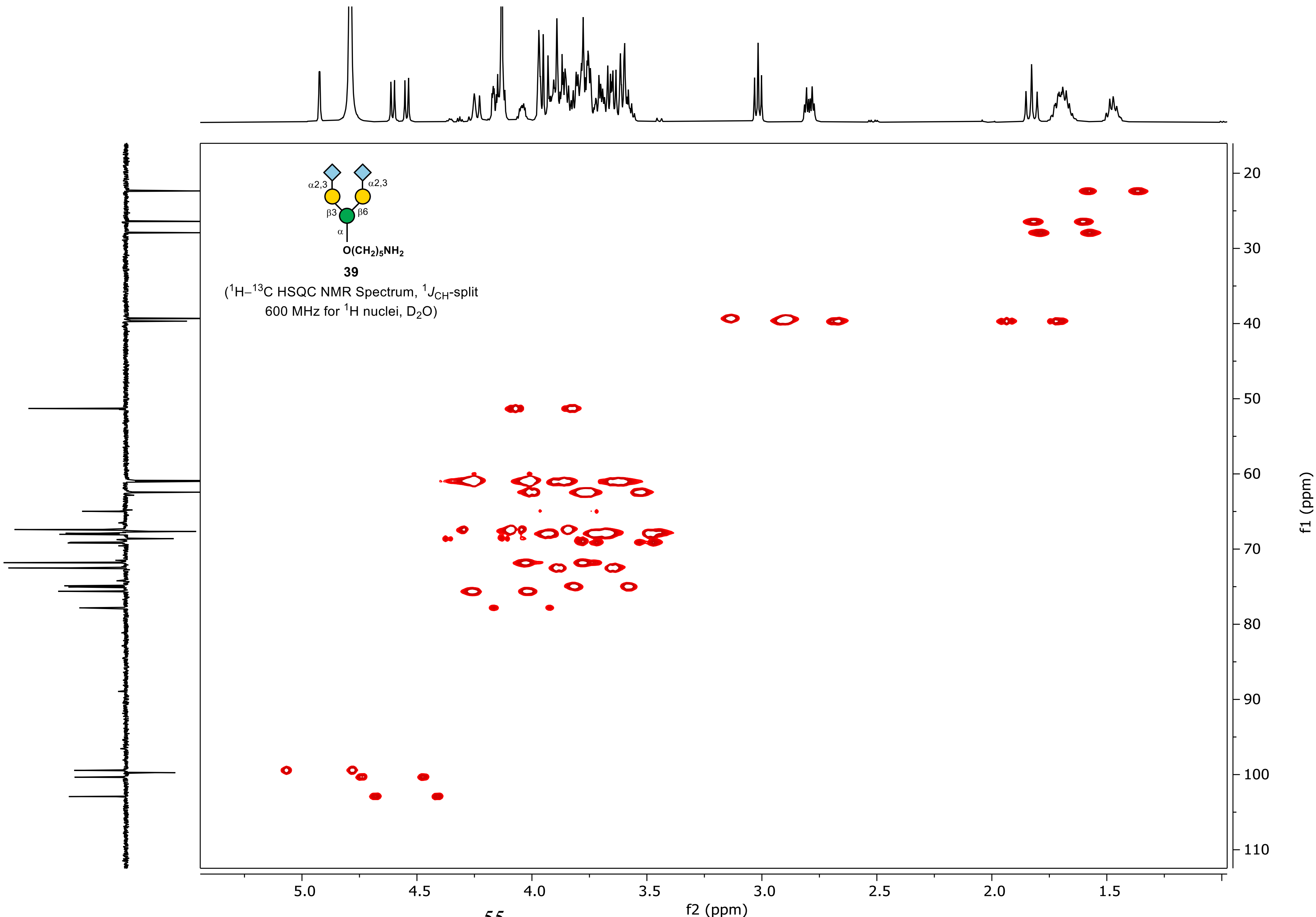

α2,3
α2,3
β3
β6
α
O(CH2)5NH2
39
(1H–13C HSQC NMR Spectrum, 1JCH-split
600 MHz for 1H nuclei, D2O)
f1 (ppm)
f2 (ppm)

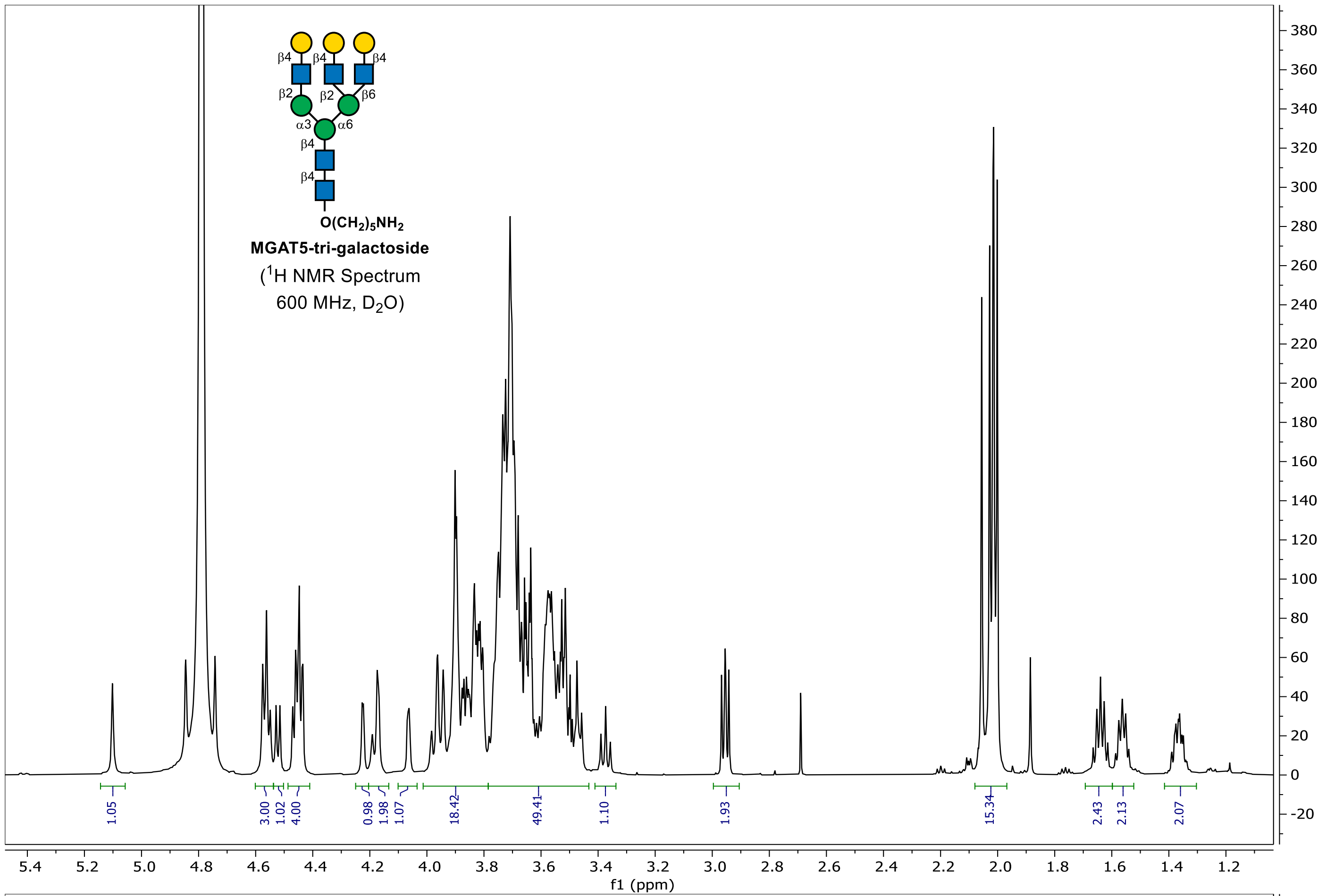
O(CH2)5NH2
MGAT5-tri-galactoside
(1H NMR Spectrum
600 MHz, D2O)
f1 (ppm)

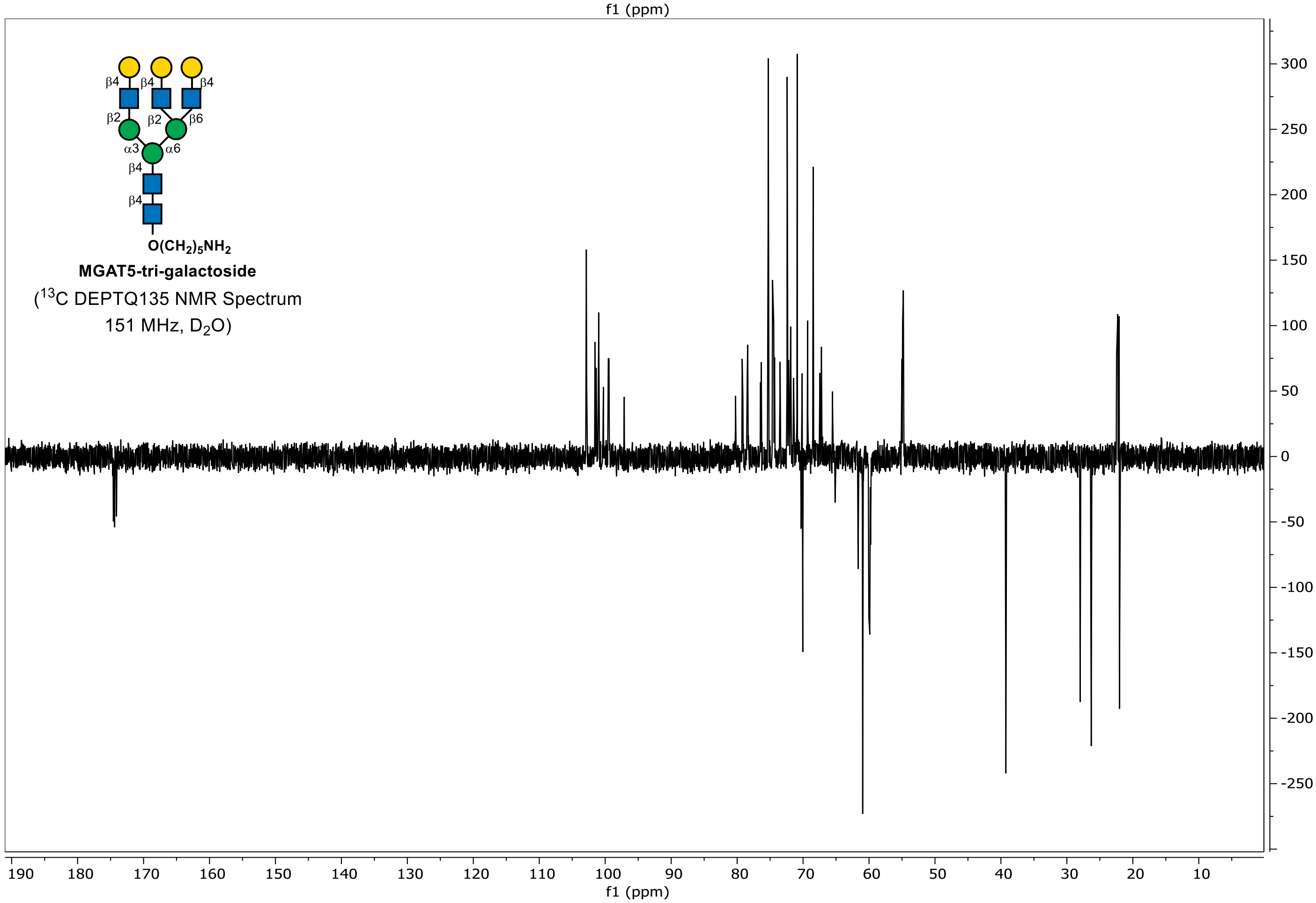
O(CH2)5NH2
MGAT5-tri-galactoside
(13C DEPTQ135 NMR Spectrum
151 MHz, D2O)
f1 (ppm)

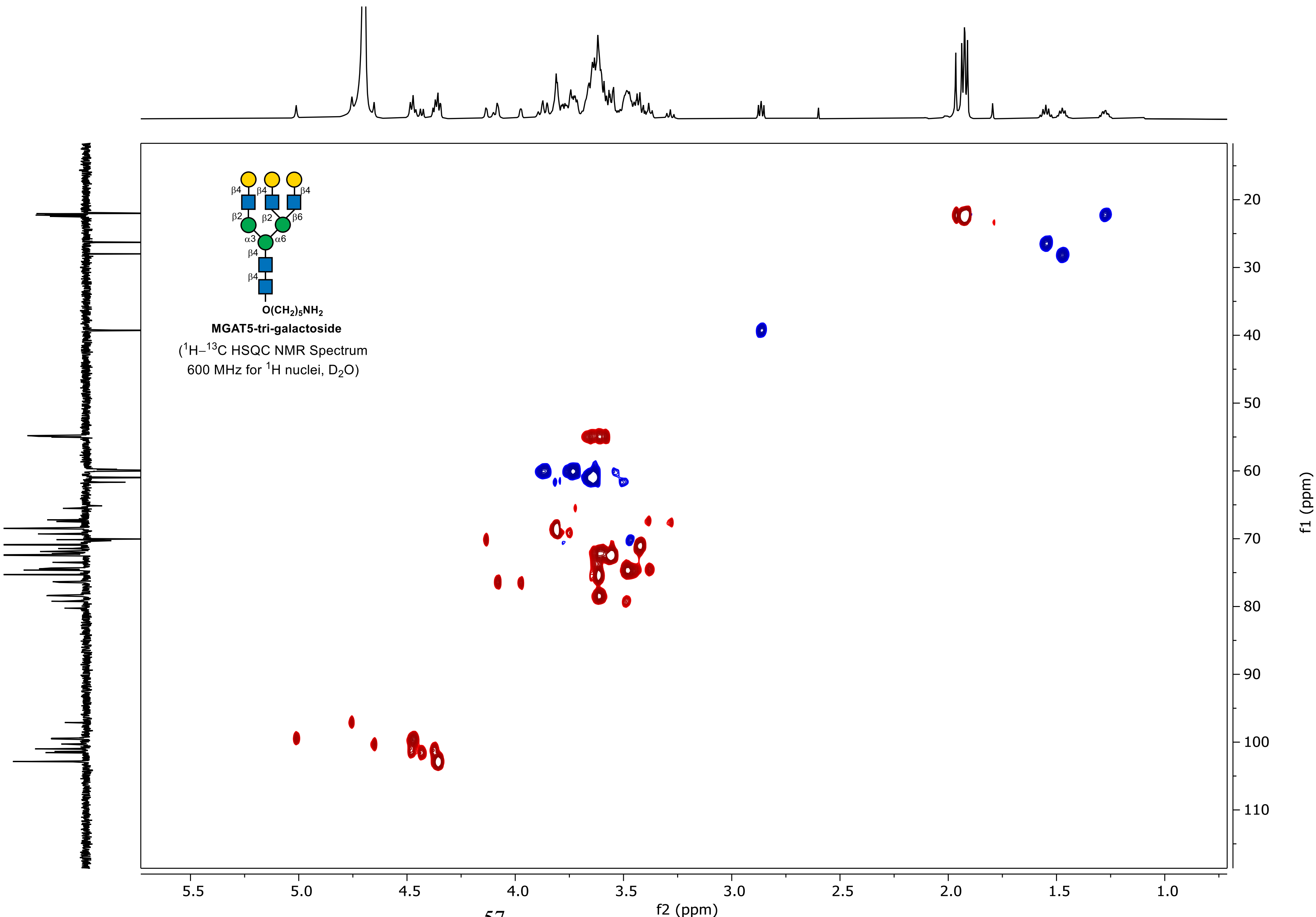
β4
β4
β4
β2
β2
β6
α3
α6
β4
β4
O(CH2)5NH2
MGAT5-tri-galactoside
(1H–13C HSQC NMR Spectrum
600 MHz for 1H nuclei, D2O)
f2 (ppm)
f1 (ppm)

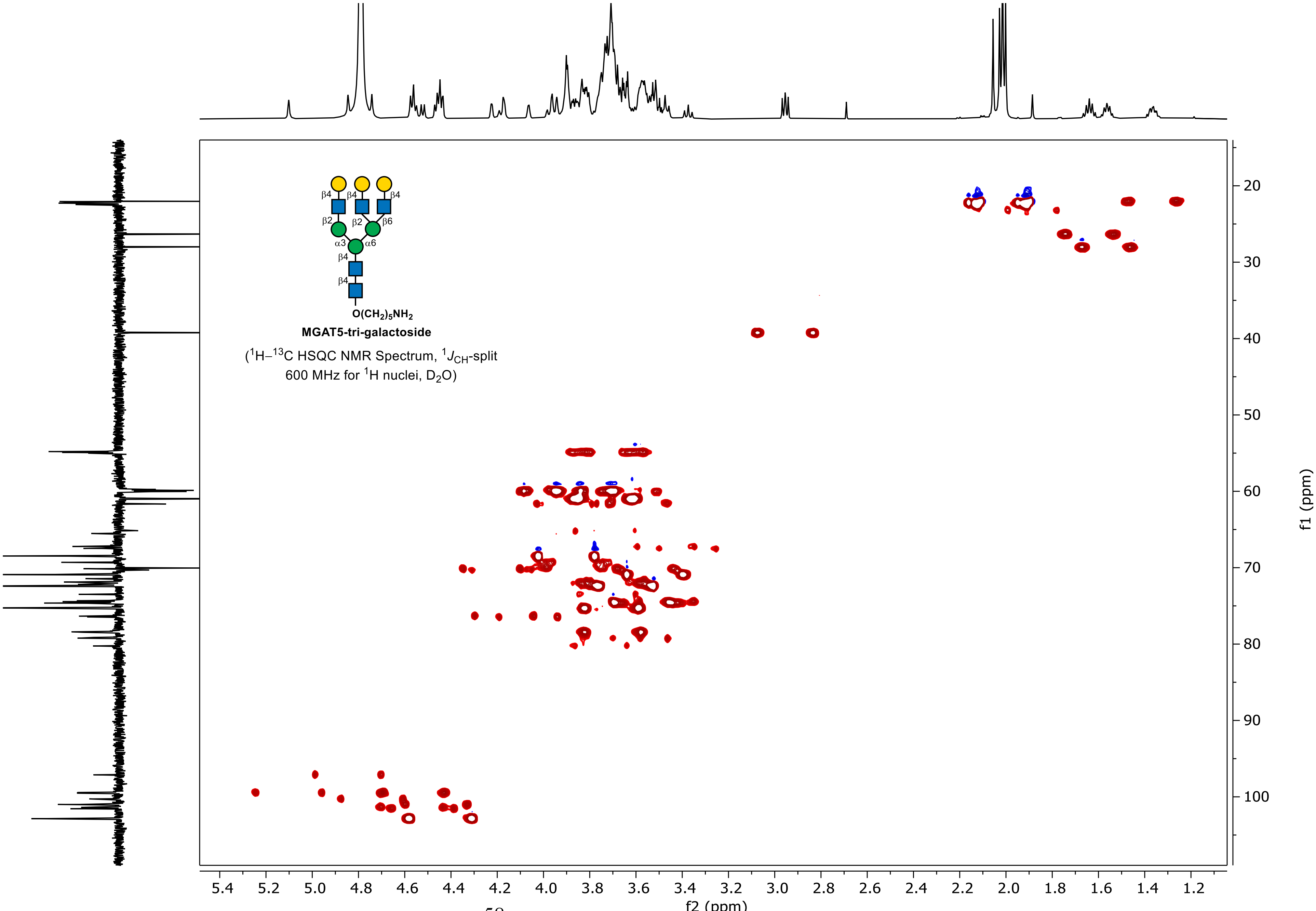
β4
β4
β4
β2
β2
β6
α3
α6
β4
β4
O(CH2)5NH2
MGAT5-tri-galactoside
(1H–13C HSQC NMR Spectrum, 1JCH-split
600 MHz for 1H nuclei, D2O)
f2 (ppm)
f1 (ppm)

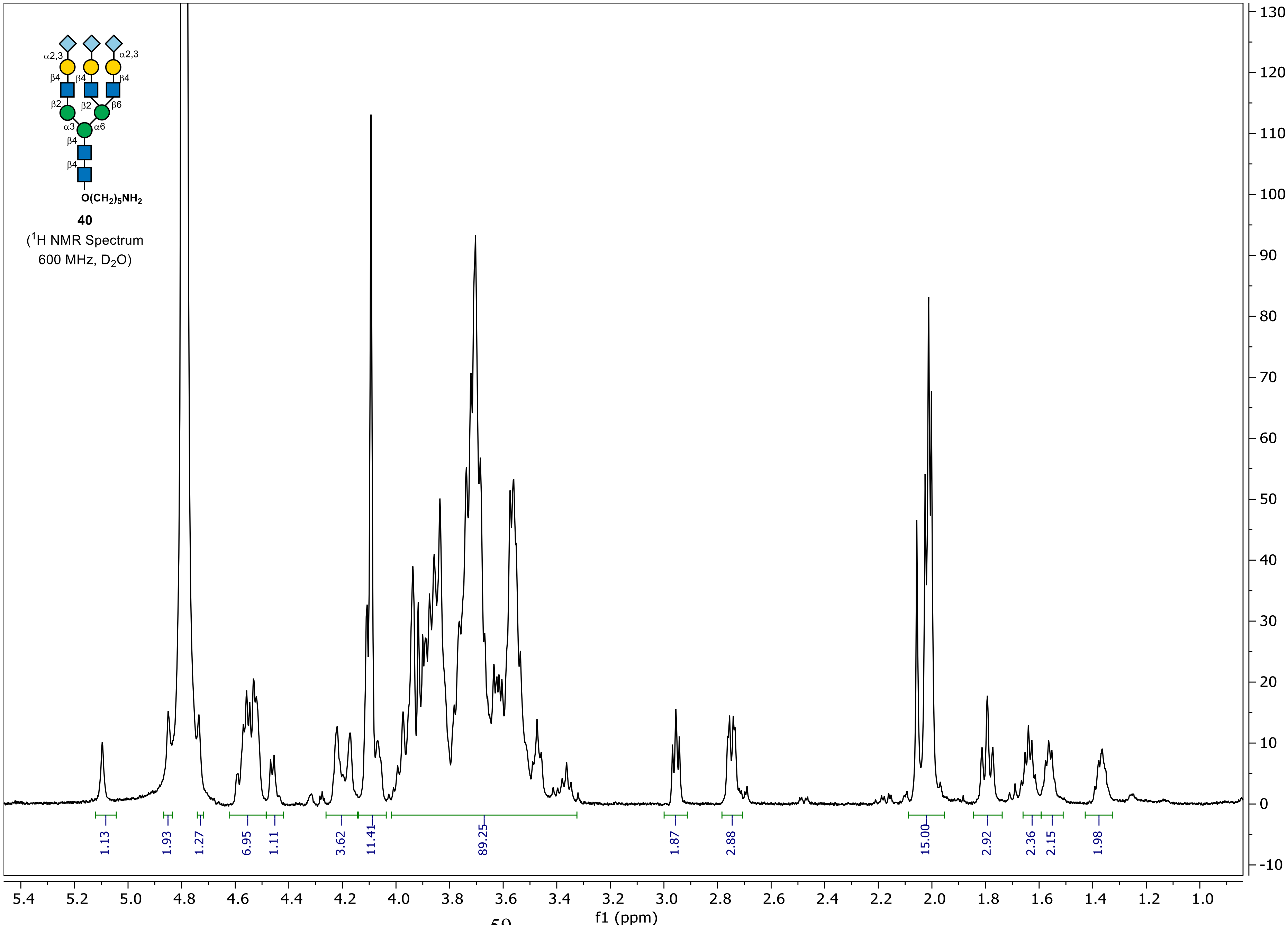

α2,3
α2,3
β4
β4
β4
β2
β2
β6
α3
α6
β4
β4
O(CH2)5NH2
40
(1H NMR Spectrum
600 MHz, D2O)
1.13
1.93
1.27
6.95
1.11
3.62
11.41
89.25
1.87
2.88
15.00
2.92
2.36
2.15
1.98
f1 (ppm)

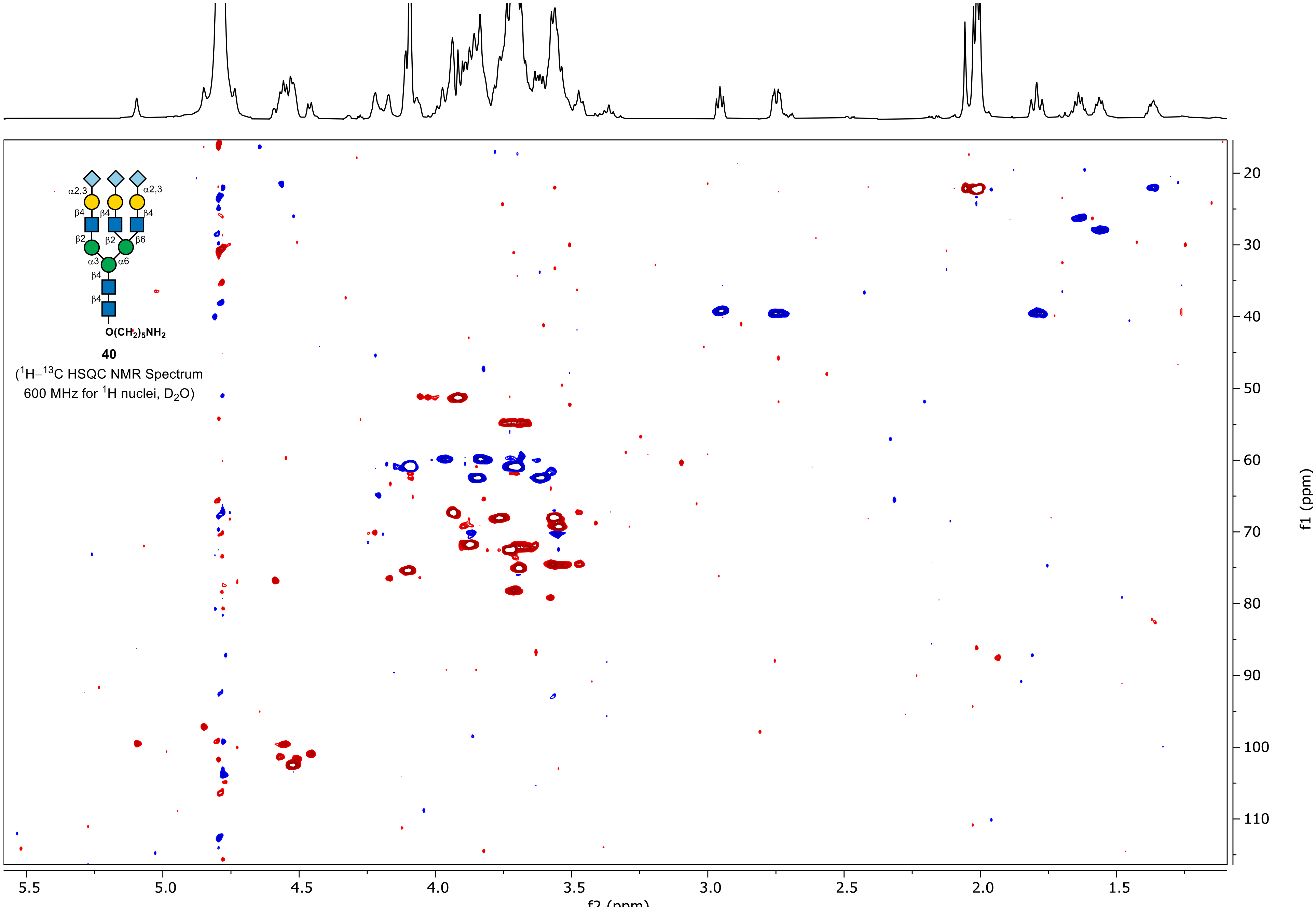
α2,3
β4
β2
α3
α6
β6
O(CH2)5NH2
40
(1H–13C HSQC NMR Spectrum
600 MHz for 1H nuclei, D2O)
f2 (ppm)
f1 (ppm)

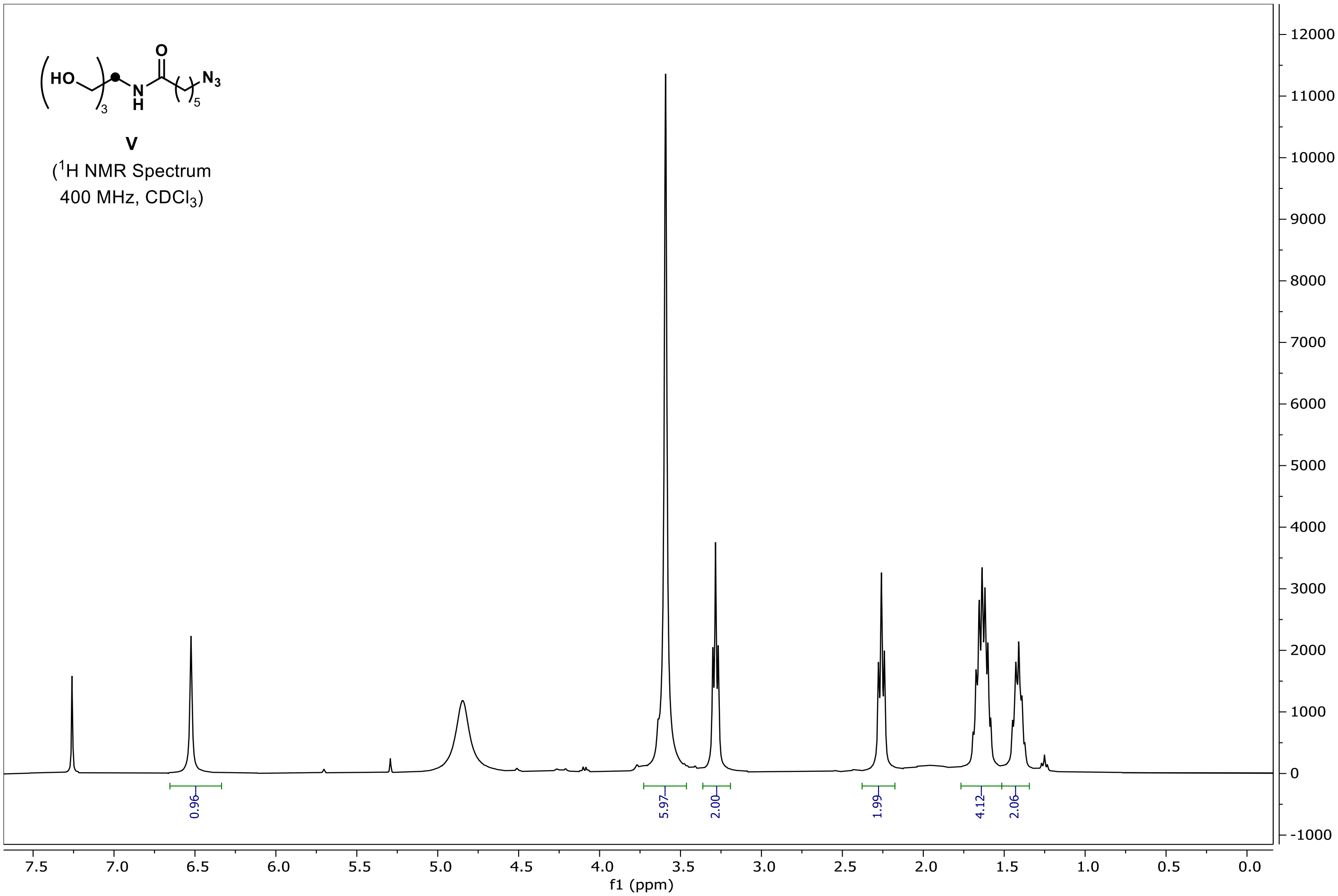
HO
N
H
O
N3
V
(1H NMR Spectrum
400 MHz, CDCl3)
f1 (ppm)

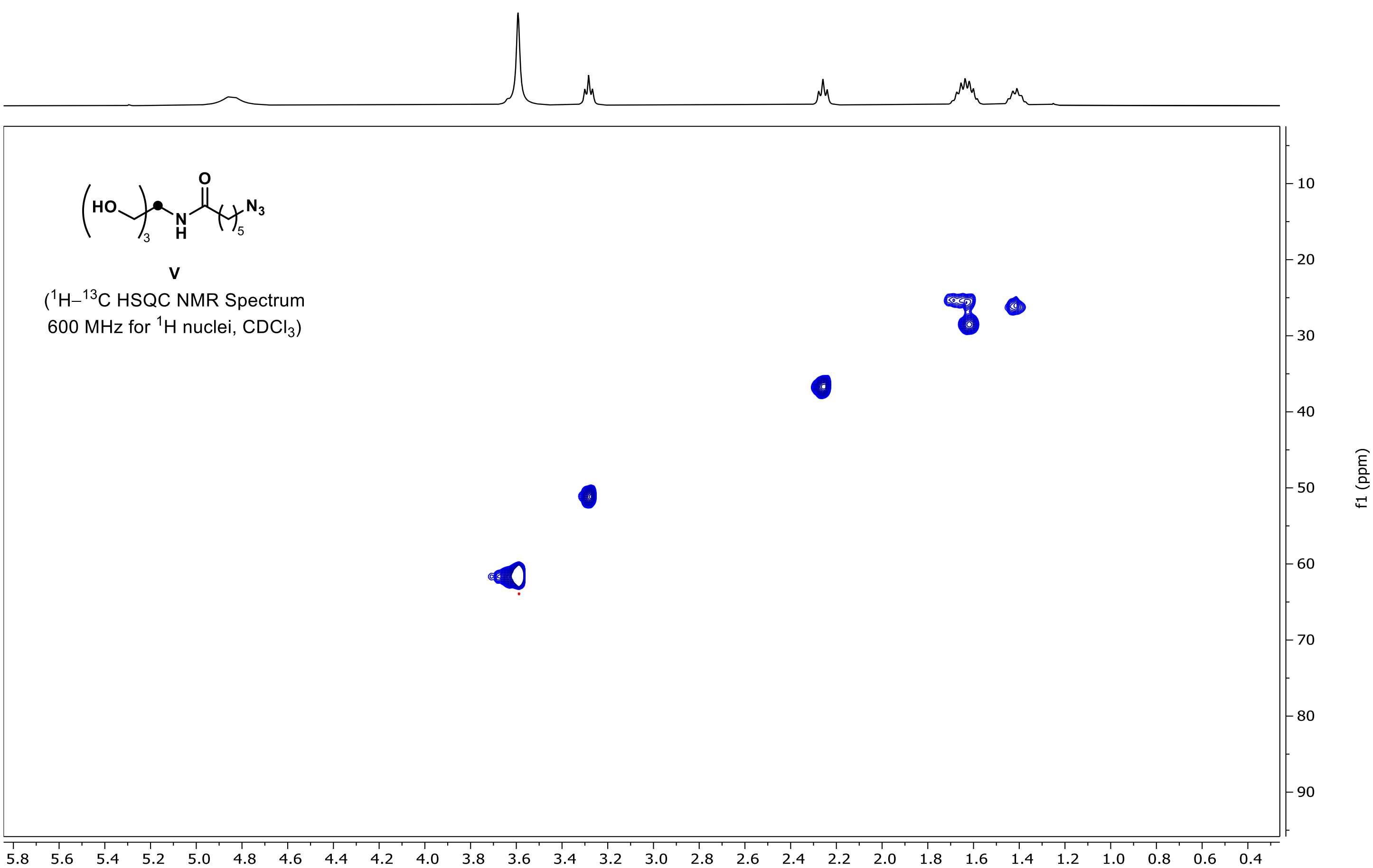
HO
N
H
O
N3
V
(1H–13C HSQC NMR Spectrum
600 MHz for 1H nuclei, CDCl3)
f2 (ppm)
f1 (ppm)

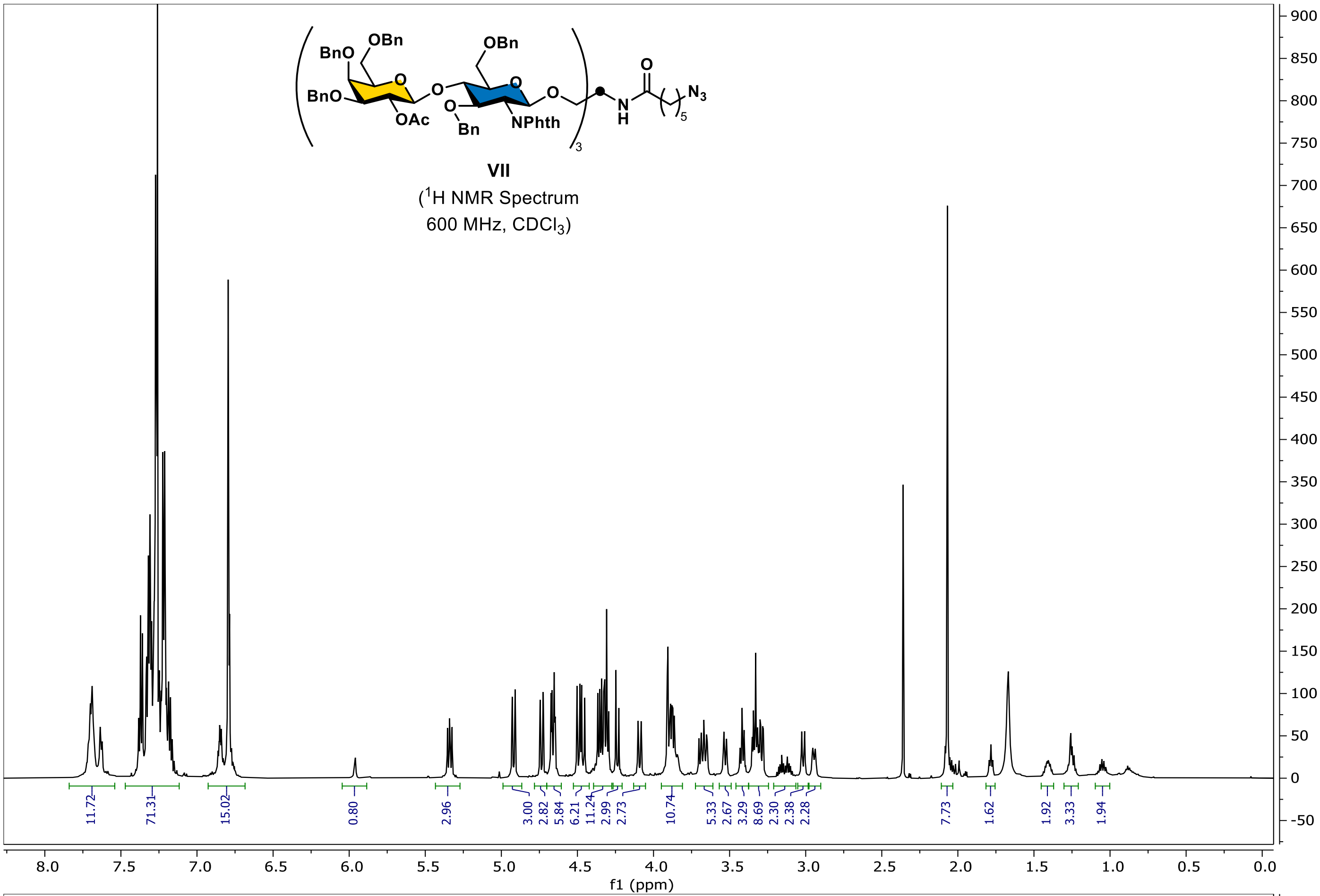
BnO
OBn
BnO
OAc
O
Bn
OBn
NPhth
N
H
O
N3
5
3
VII
(1H NMR Spectrum
600 MHz, CDCl3)
f1 (ppm)

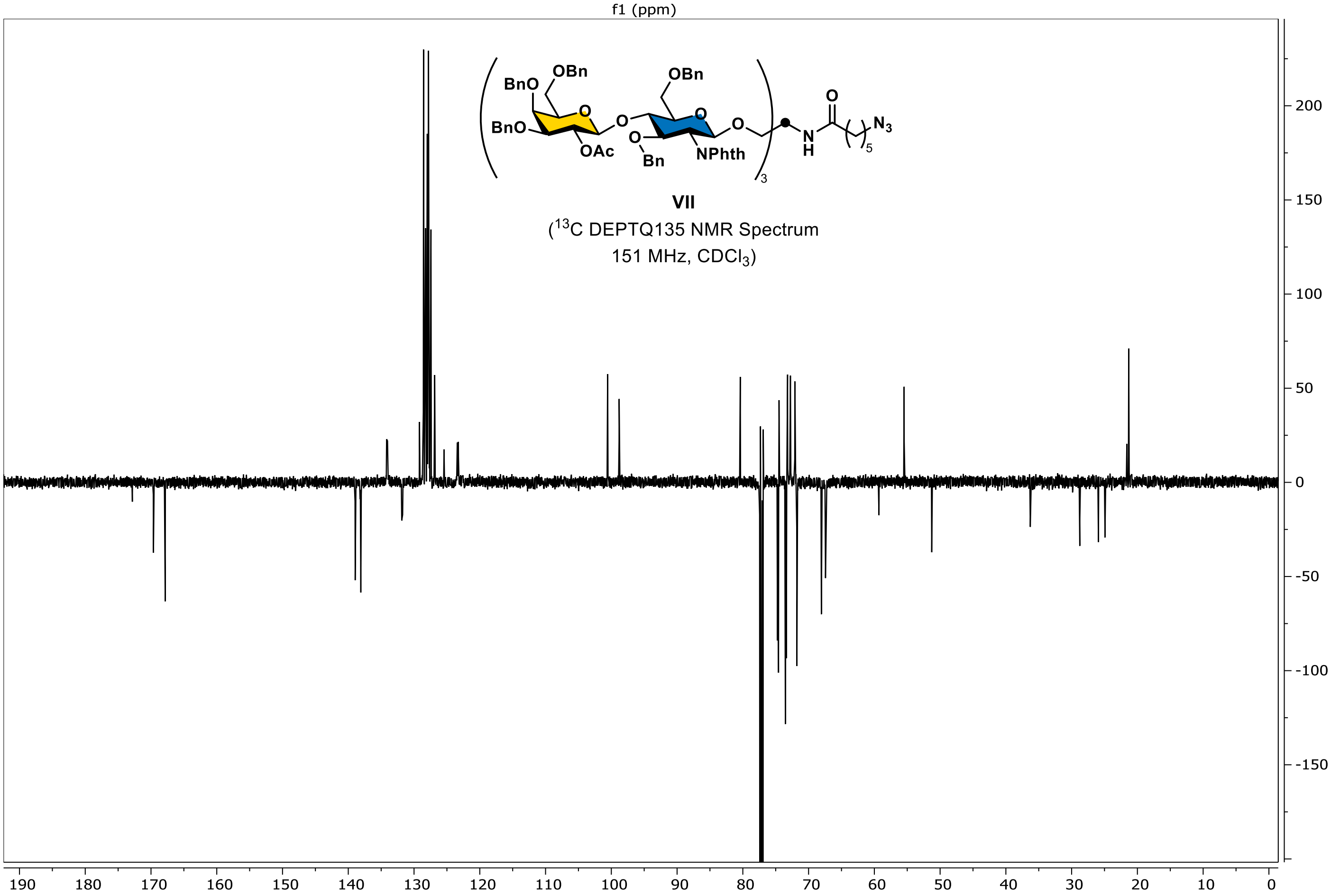
BnO
OBn
BnO
OAc
O
Bn
OBn
NPhth
N
H
O
N3
5
3
VII
(13C DEPTQ135 NMR Spectrum
151 MHz, CDCl3)
f1 (ppm)

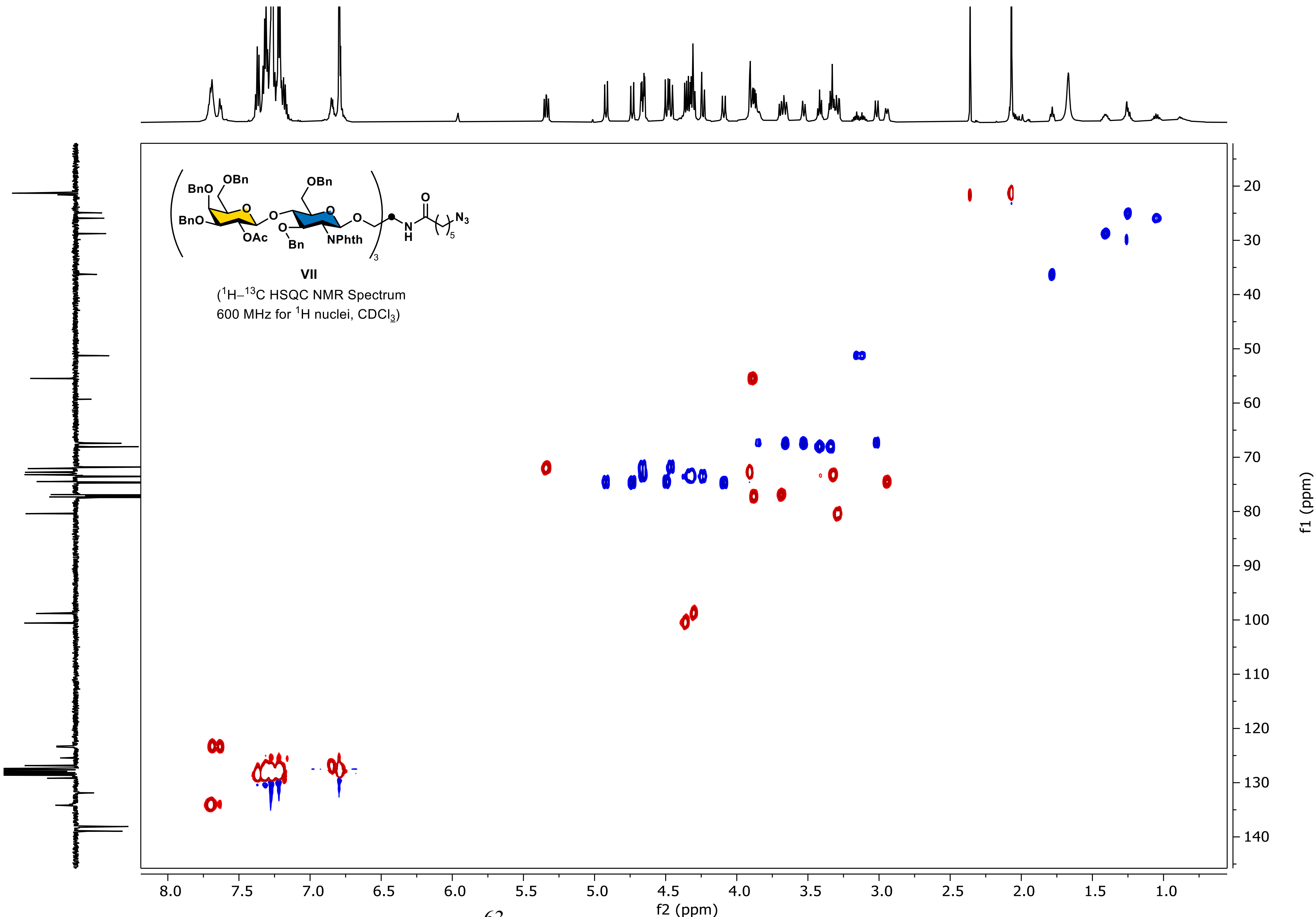

BnO
OBn
BnO
O
OAc
O
O
Bn
OBn
O
NPhth
O
N
H
O
N3
5
3
VII
(1H–13C HSQC NMR Spectrum
600 MHz for 1H nuclei, CDCl3)
f1 (ppm)
f2 (ppm)

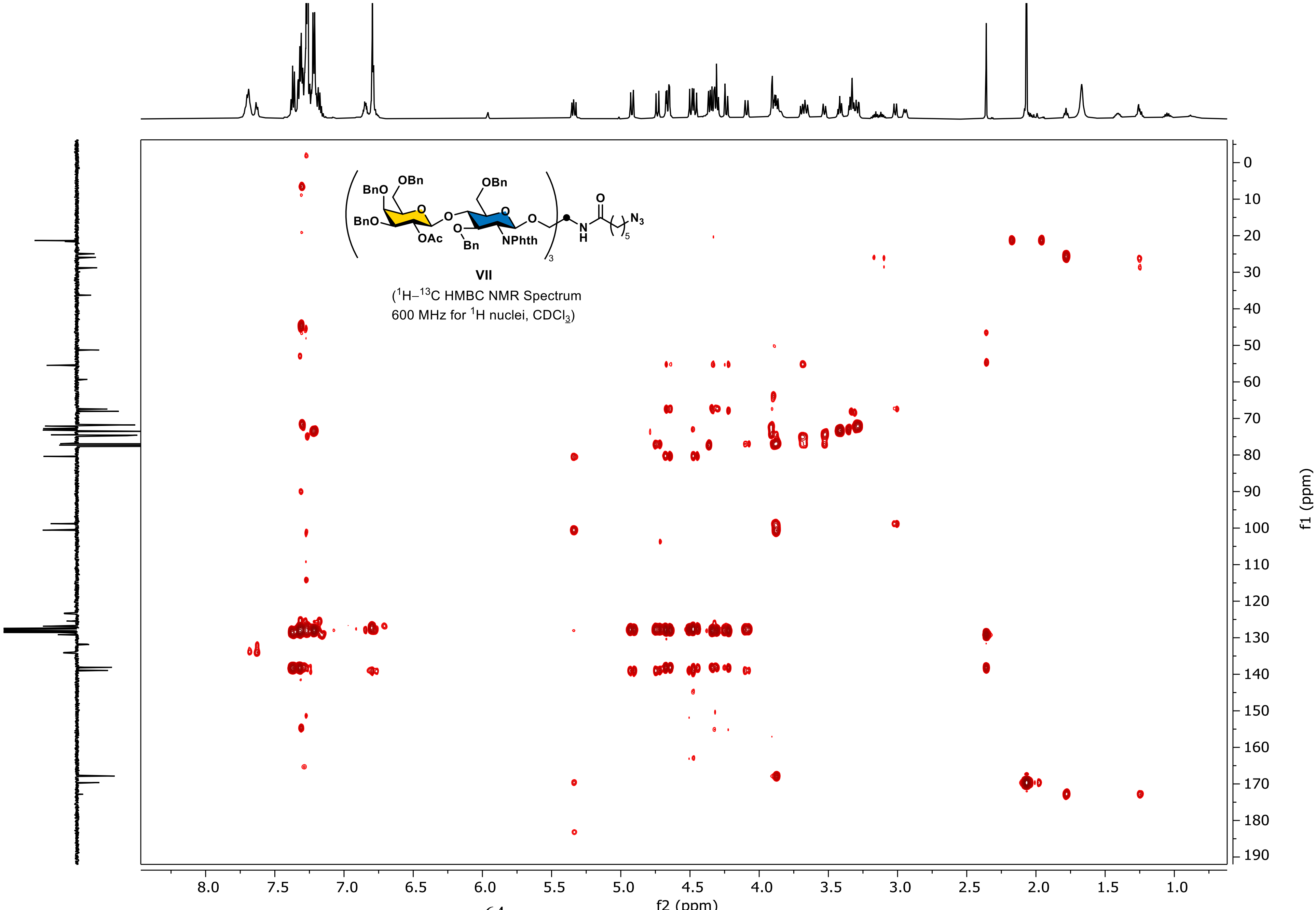

BnO
OBn
BnO
OAc
O
O
O
OBn
O
Bn
NPhth
O
3
N
H
O
5
N3
VII
(1H–13C HMBC NMR Spectrum
600 MHz for 1H nuclei, CDCl3)
f2 (ppm)
f1 (ppm)

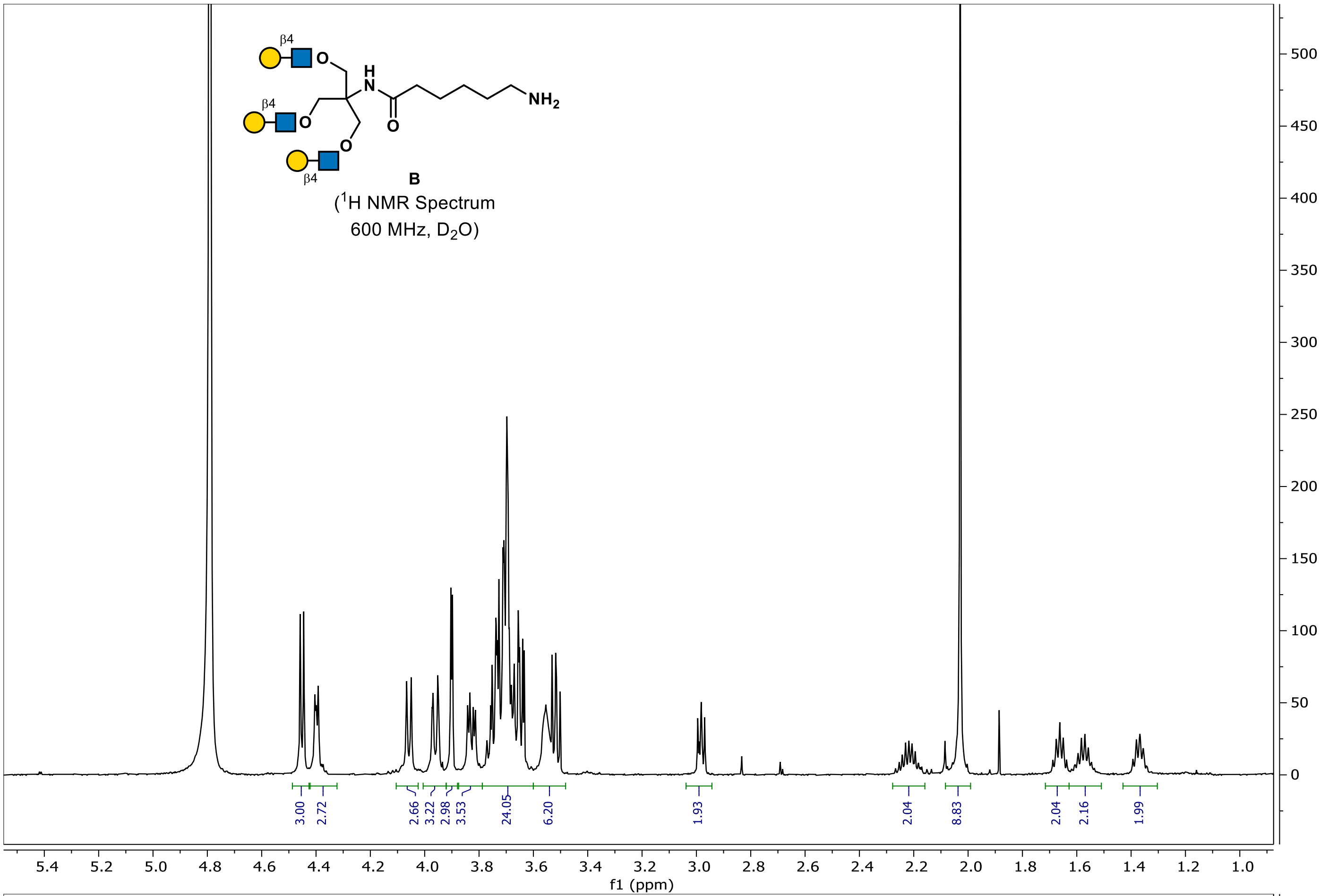
β4
β4
β4
H
N
NH2
O
O
O
O
B
(1H NMR Spectrum
600 MHz, D2O)
3.00
2.72
2.66
3.22
2.98
3.53
24.05
6.20
1.93
2.04
8.83
2.04
2.16
1.99
f1 (ppm)

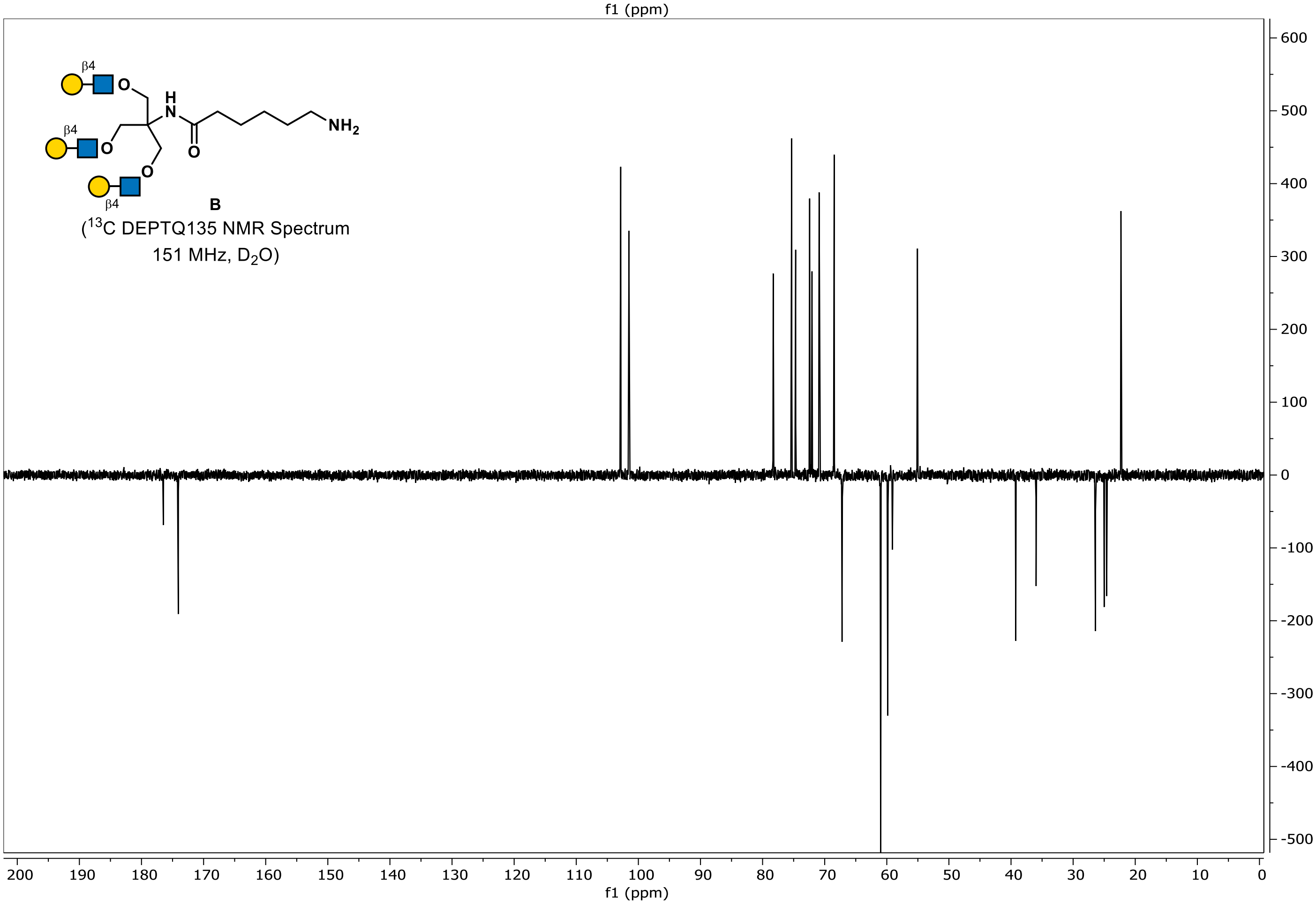
β4
β4
β4
H
N
NH2
O
O
O
O
B
(13C DEPTQ135 NMR Spectrum
151 MHz, D2O)
f1 (ppm)

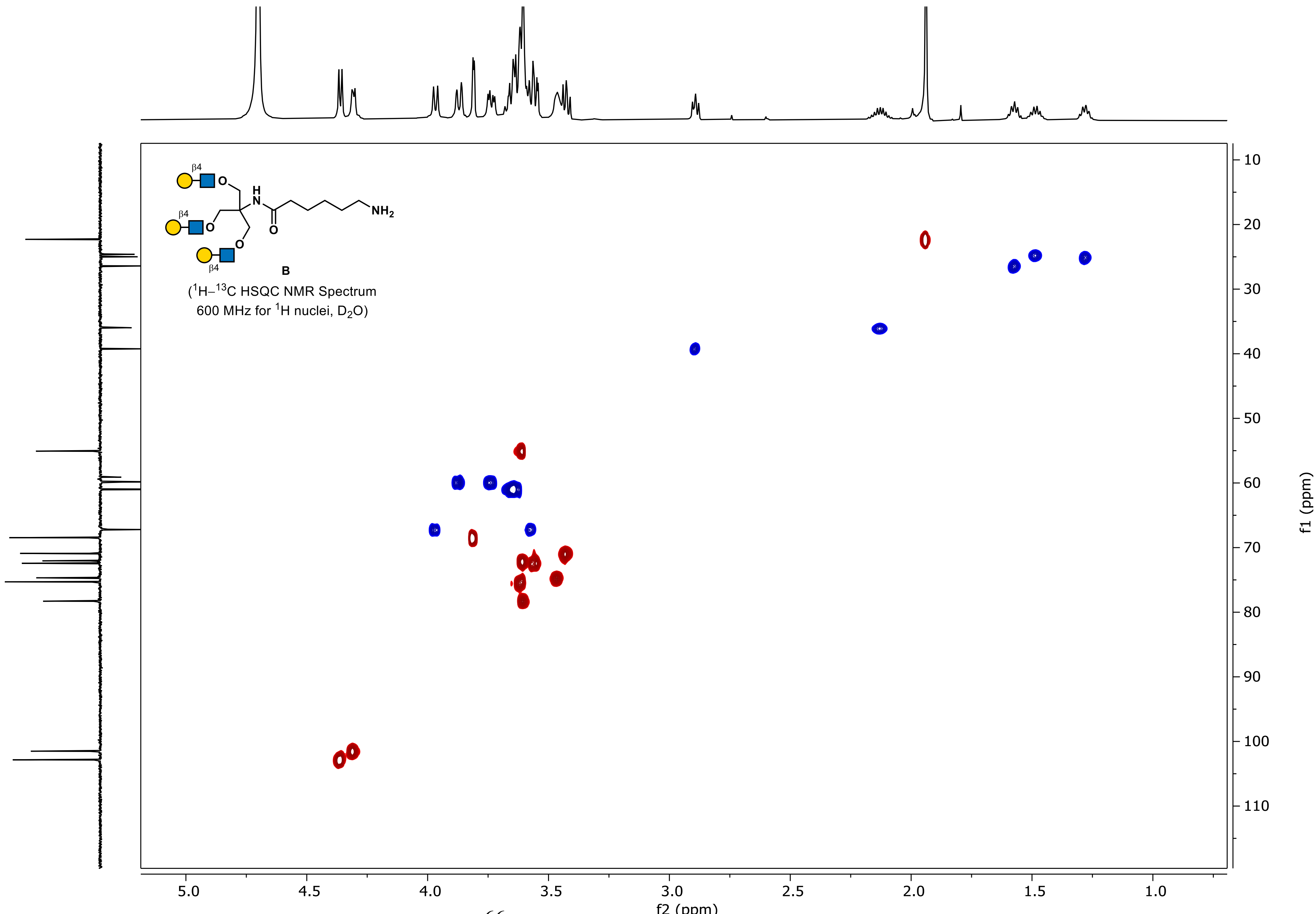
β4
O
H
N
NH2
O
B
(1H–13C HSQC NMR Spectrum
600 MHz for 1H nuclei, D2O)
f2 (ppm)
f1 (ppm)

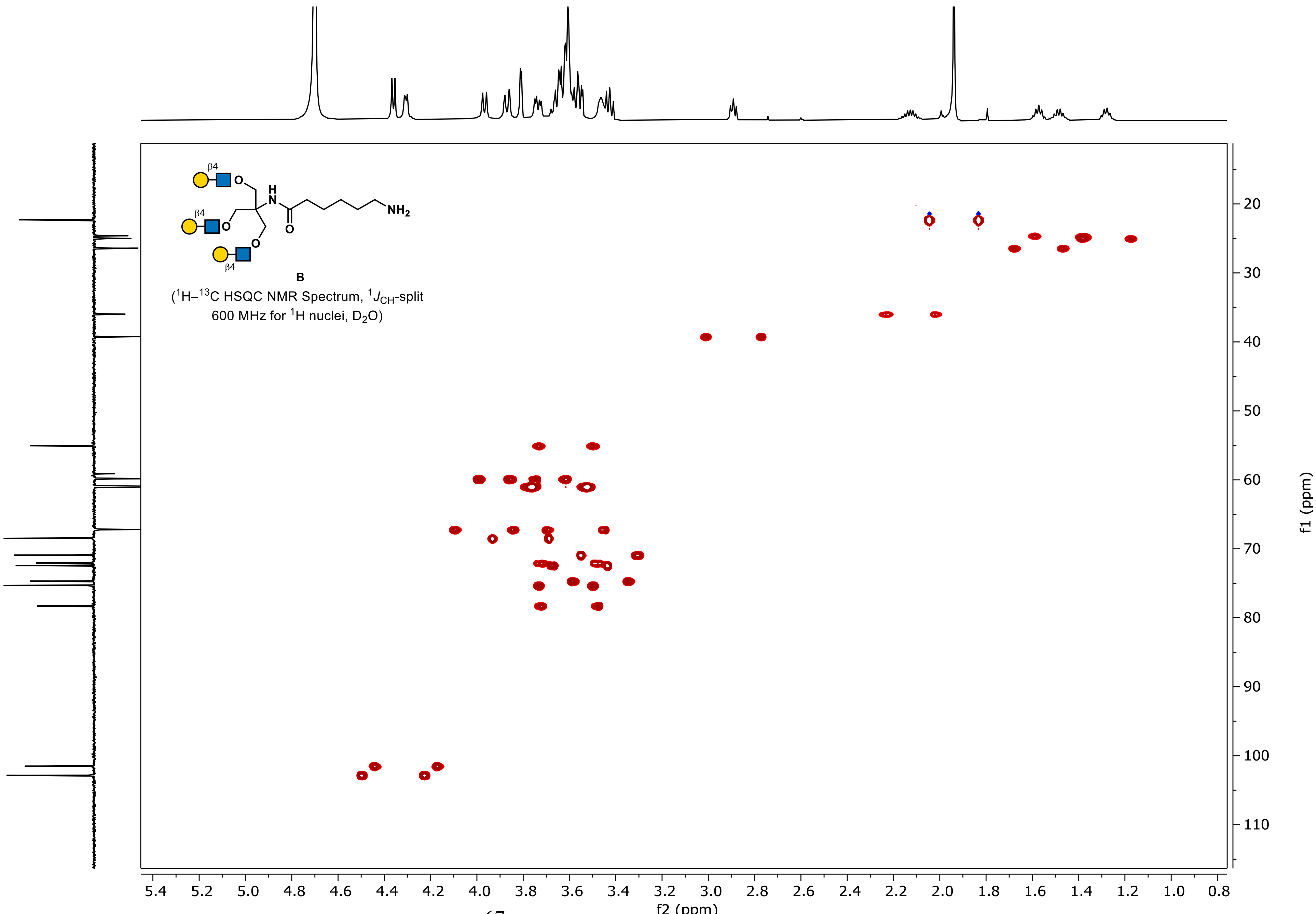
β4
β4
β4
H
N
NH2
O
O
O
O
B
(1H–13C HSQC NMR Spectrum, 1JCH-split
600 MHz for 1H nuclei, D2O)
f1 (ppm)
f2 (ppm)

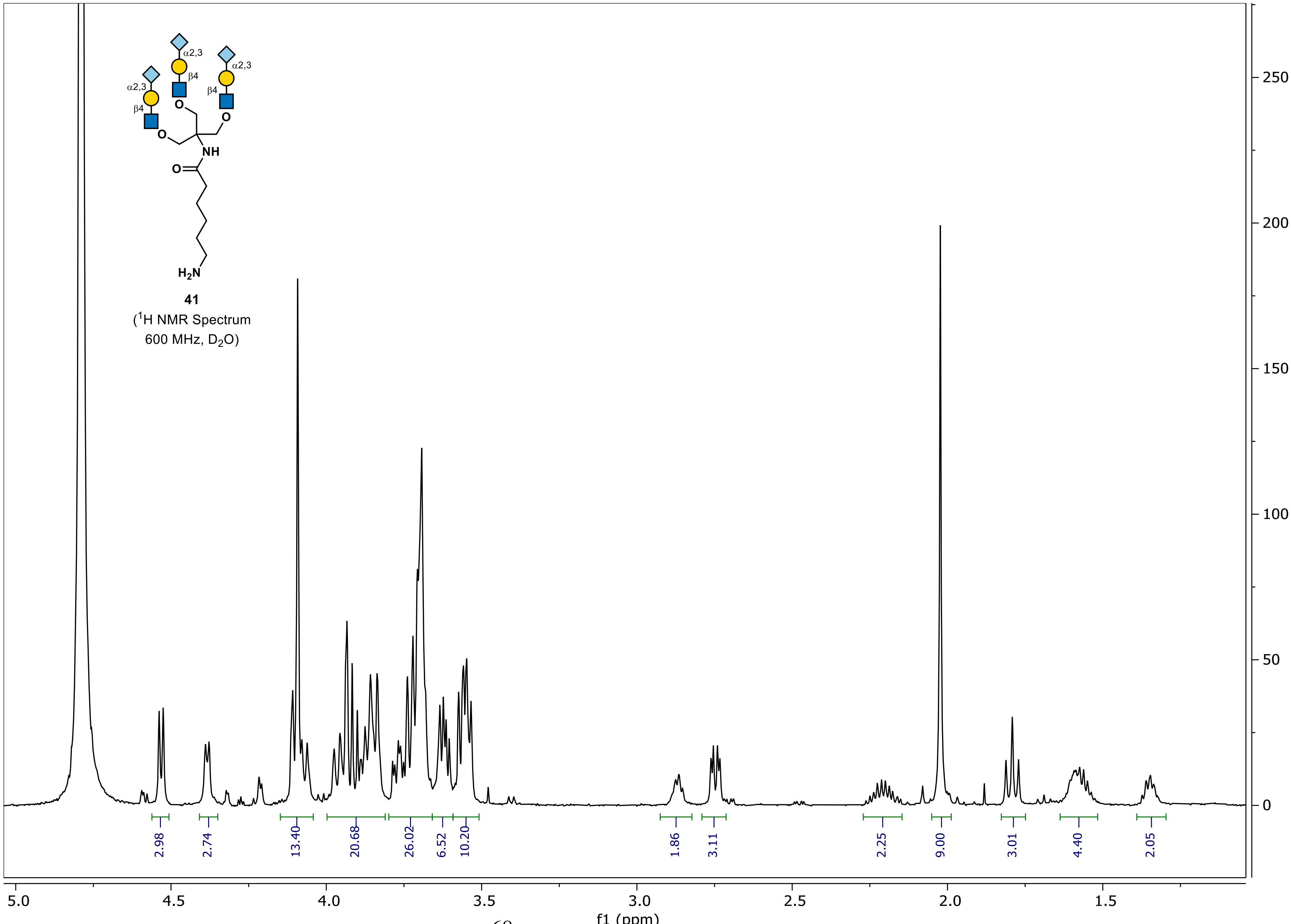
α2,3
β4
α2,3
β4
α2,3
β4
O
O
O
NH
O
H2N
41
(1H NMR Spectrum
600 MHz, D2O)
250
200
150
100
50
0
2.98
2.74
13.40
20.68
26.02
6.52
10.20
1.86
3.11
2.25
9.00
3.01
4.40
2.05
5.0
4.5
4.0
3.5
3.0
2.5
2.0
1.5
f1 (ppm)

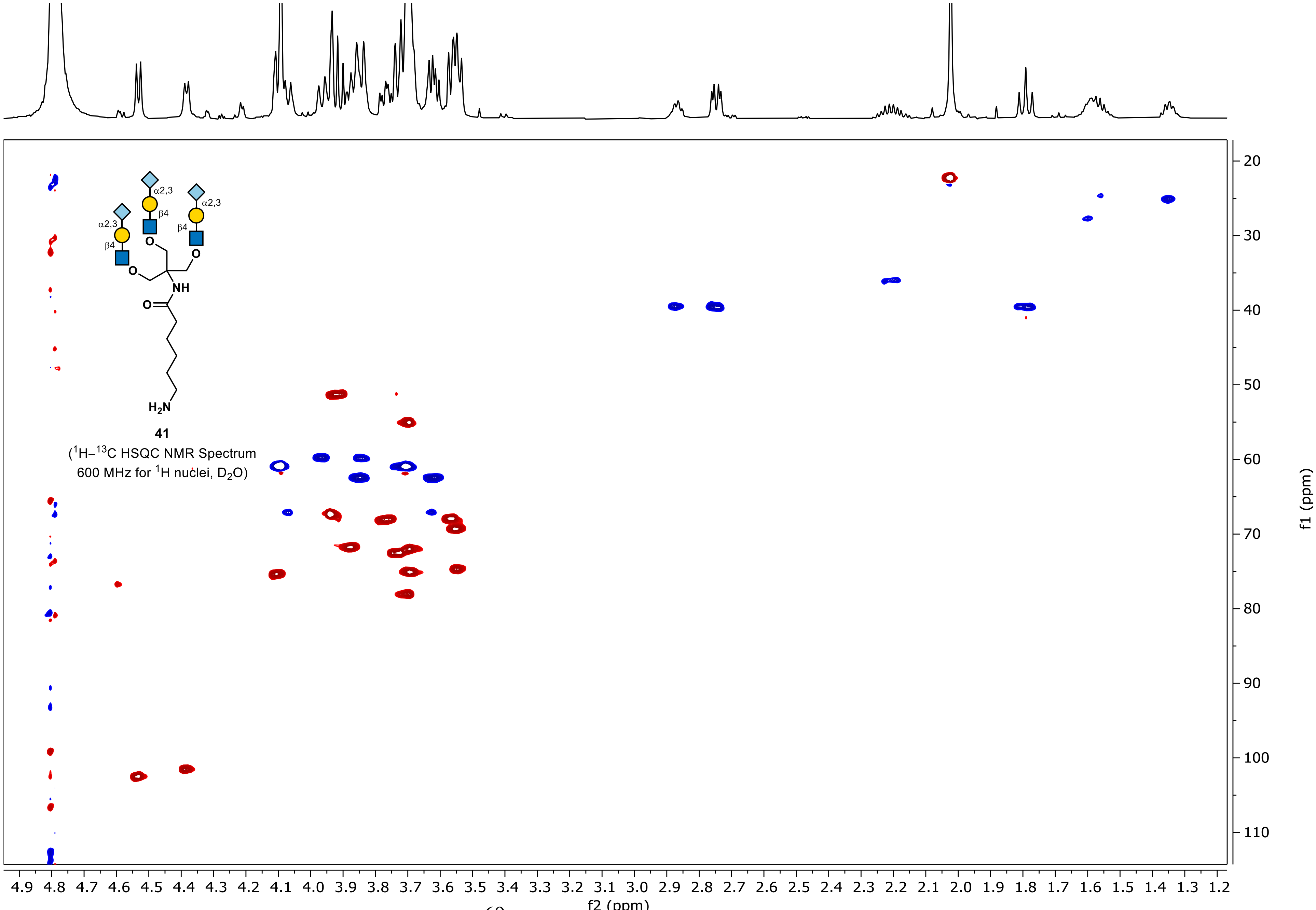

α2,3
β4
O
NH
O
H2N
41
(1H–13C HSQC NMR Spectrum
600 MHz for 1H nuclei, D2O)
f2 (ppm)
f1 (ppm)

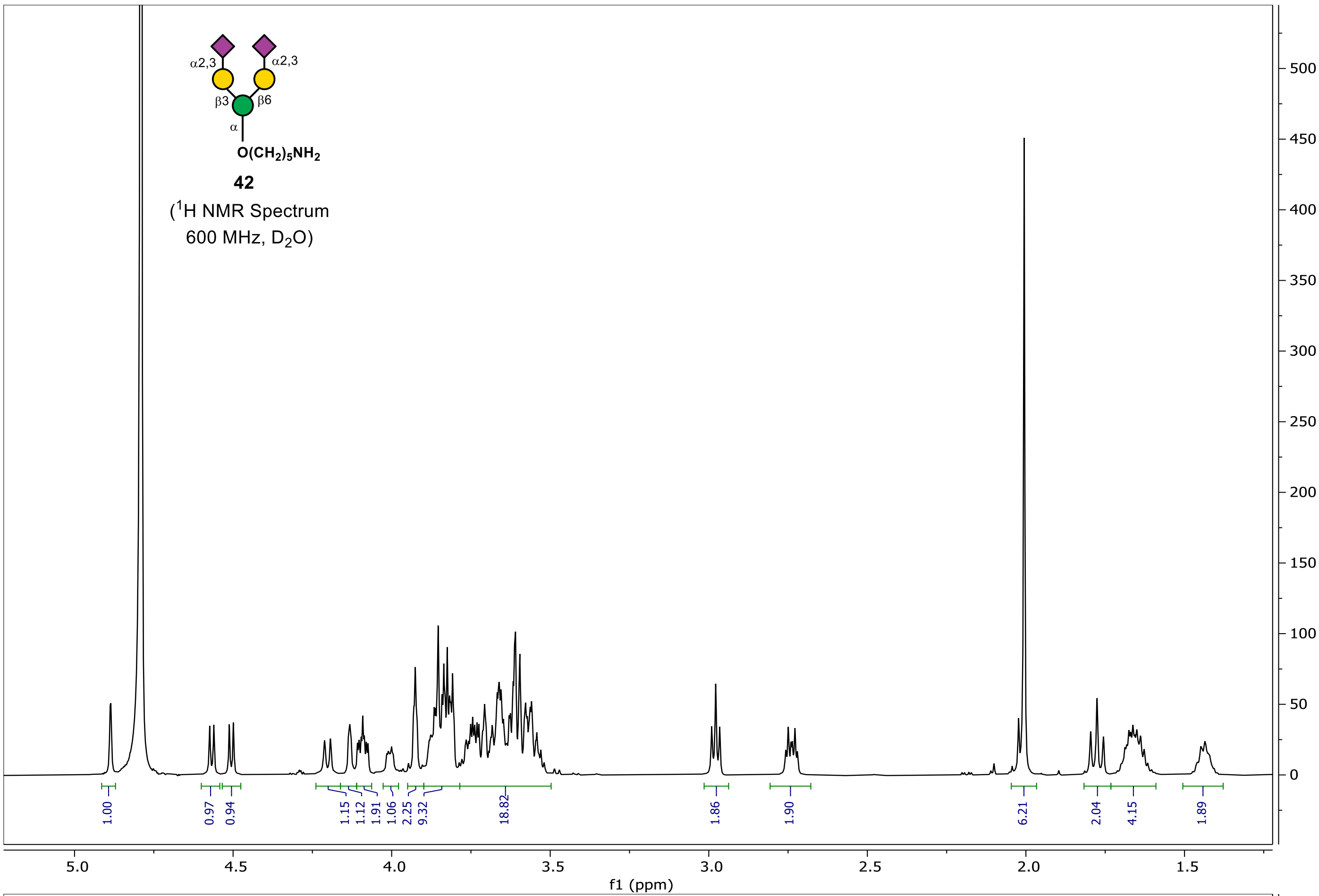
α2,3
α2,3
β3
β6
α
O(CH2)5NH2
42
(1H NMR Spectrum
600 MHz, D2O)
f1 (ppm)

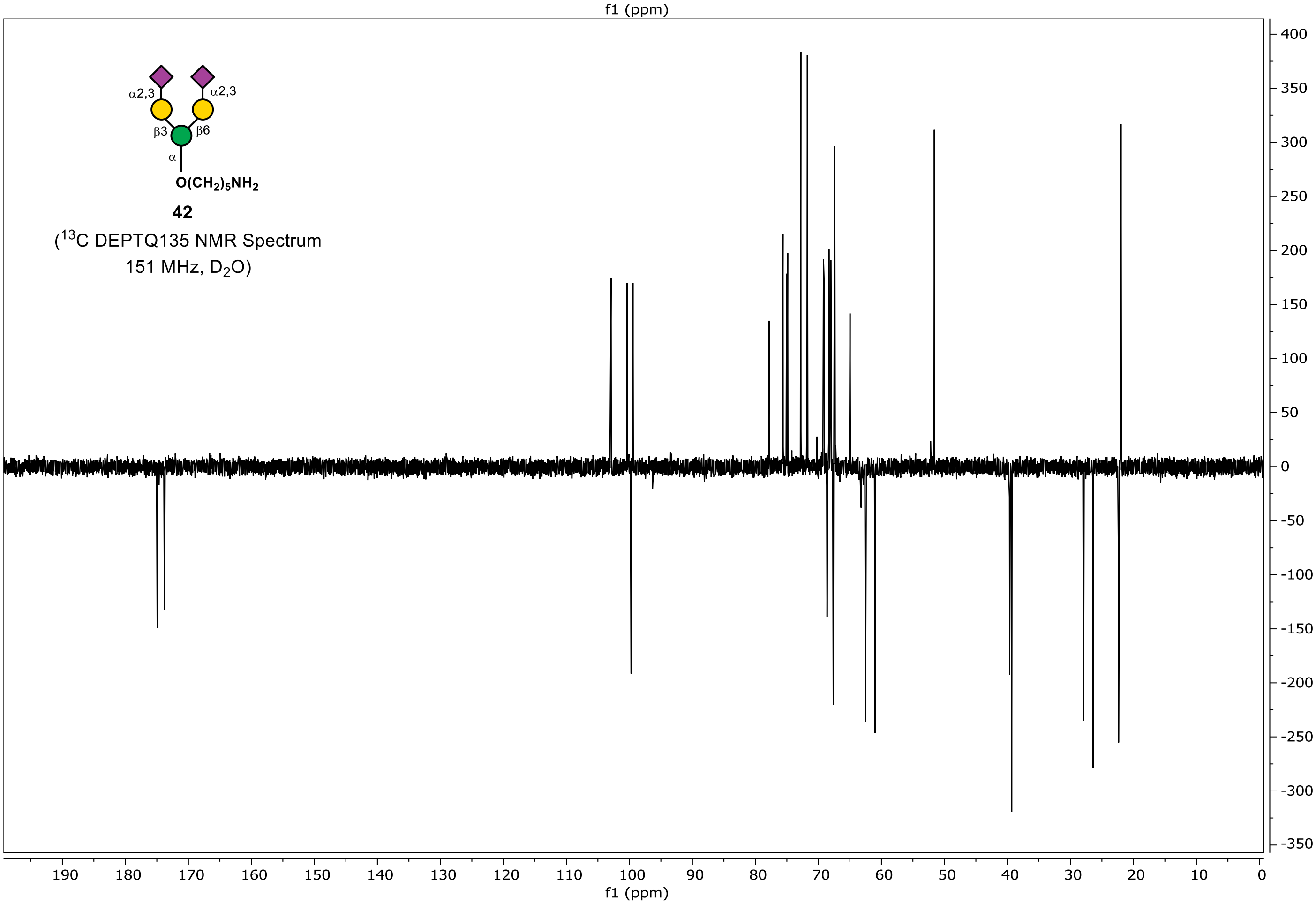
α2,3
α2,3
β3
β6
α
O(CH2)5NH2
42
(13C DEPTQ135 NMR Spectrum
151 MHz, D2O)
f1 (ppm)

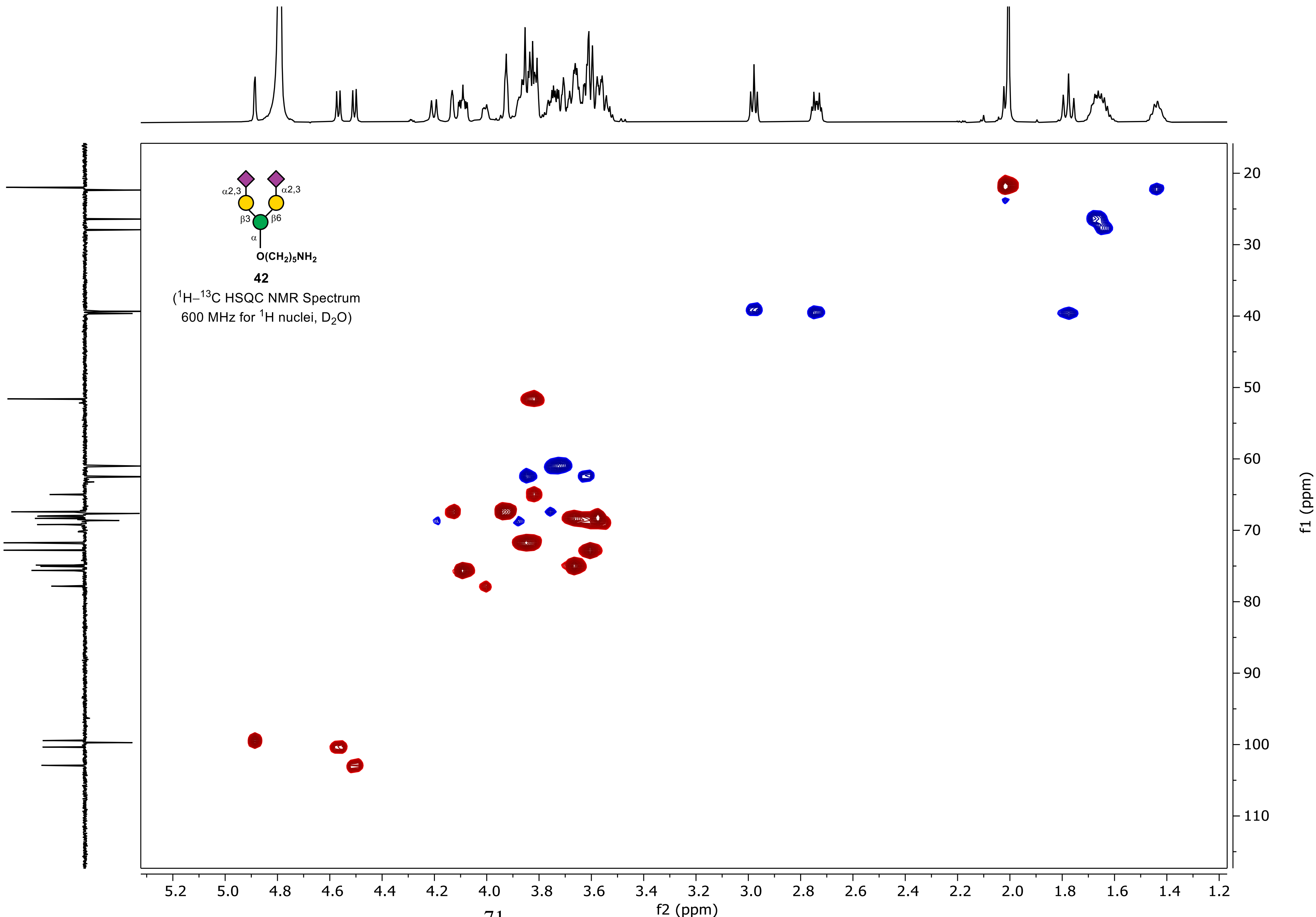
α2,3
α2,3
β3
β6
α
O(CH2)5NH2
42
(1H–13C HSQC NMR Spectrum
600 MHz for 1H nuclei, D2O)
f2 (ppm)
f1 (ppm)

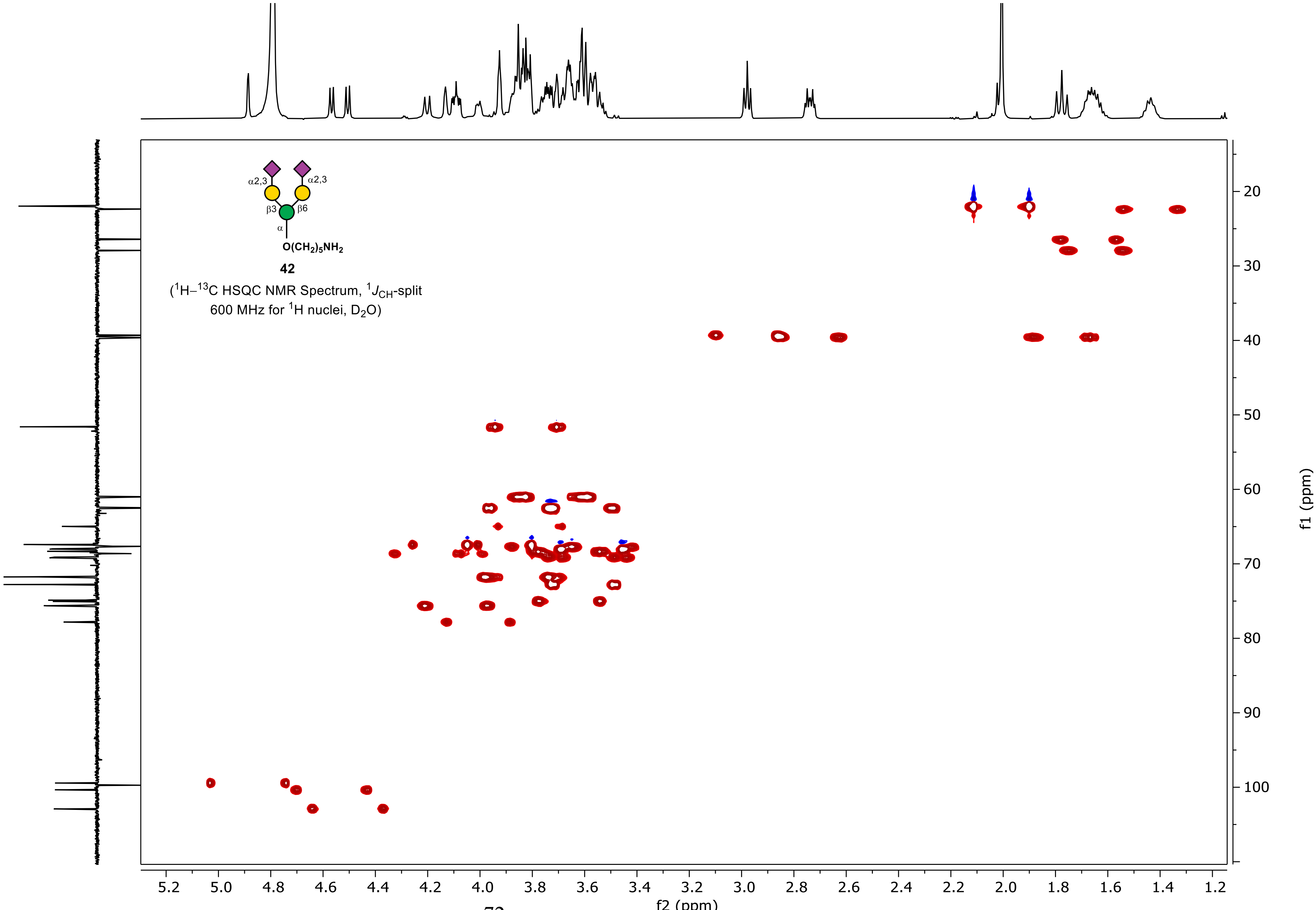
α2,3
α2,3
β3
β6
α
O(CH2)5NH2
42
(1H–13C HSQC NMR Spectrum, 1JCH-split
600 MHz for 1H nuclei, D2O)
f2 (ppm)
f1 (ppm)

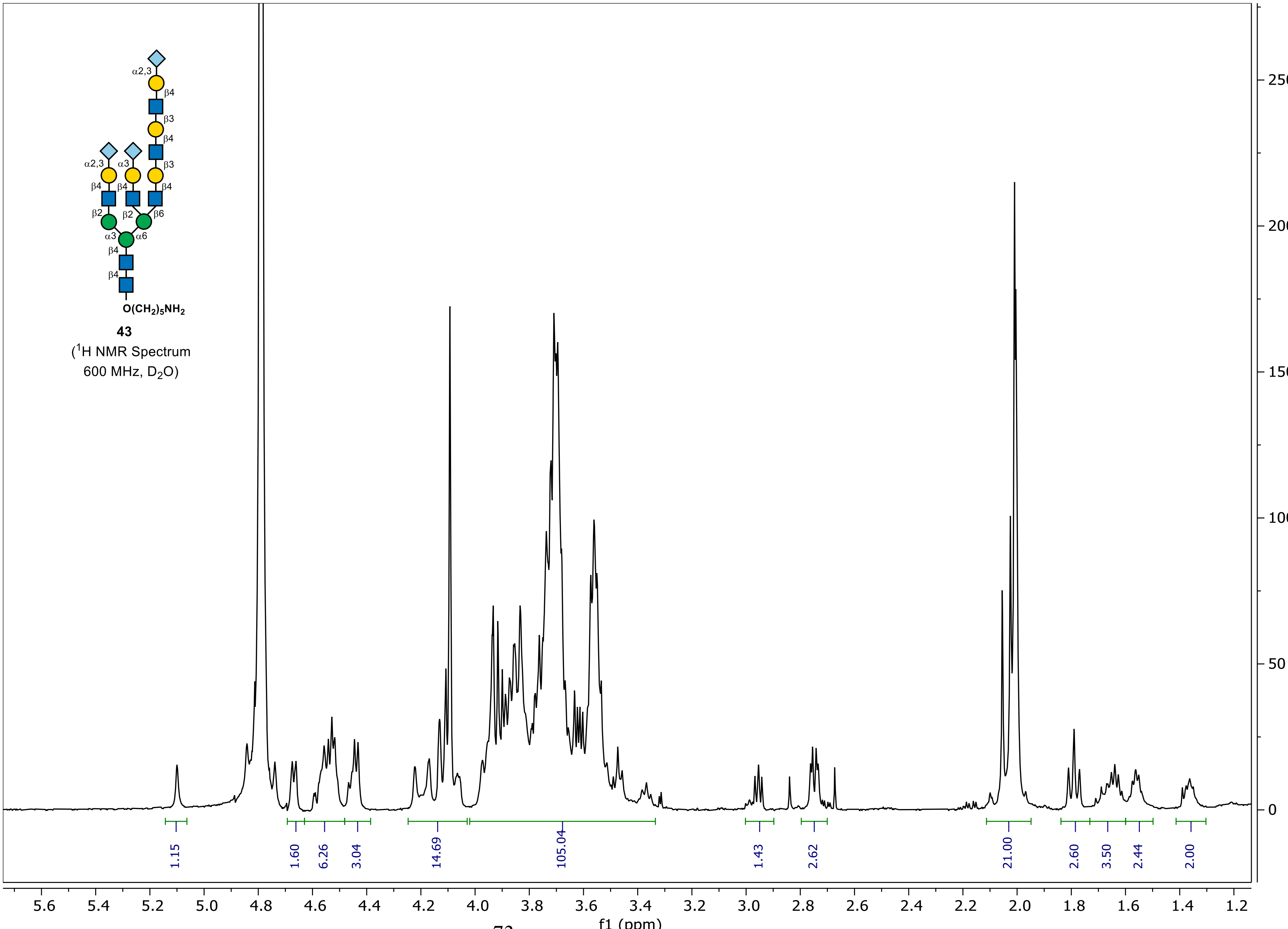

α2,3
β4
β3
β4
β3
α2,3
α3
β4
β4
β4
β2
β2
β6
α3
α6
β4
β4
O(CH2)5NH2
43
(1H NMR Spectrum
600 MHz, D2O)
1.15
1.60
6.26
3.04
14.69
105.04
1.43
2.62
21.00
2.60
3.50
2.44
2.00
5.6 5.4 5.2 5.0 4.8 4.6 4.4 4.2 4.0 3.8 3.6 3.4 3.2 3.0 2.8 2.6 2.4 2.2 2.0 1.8 1.6 1.4 1.2
f1 (ppm)
0 50 100 150 200 250

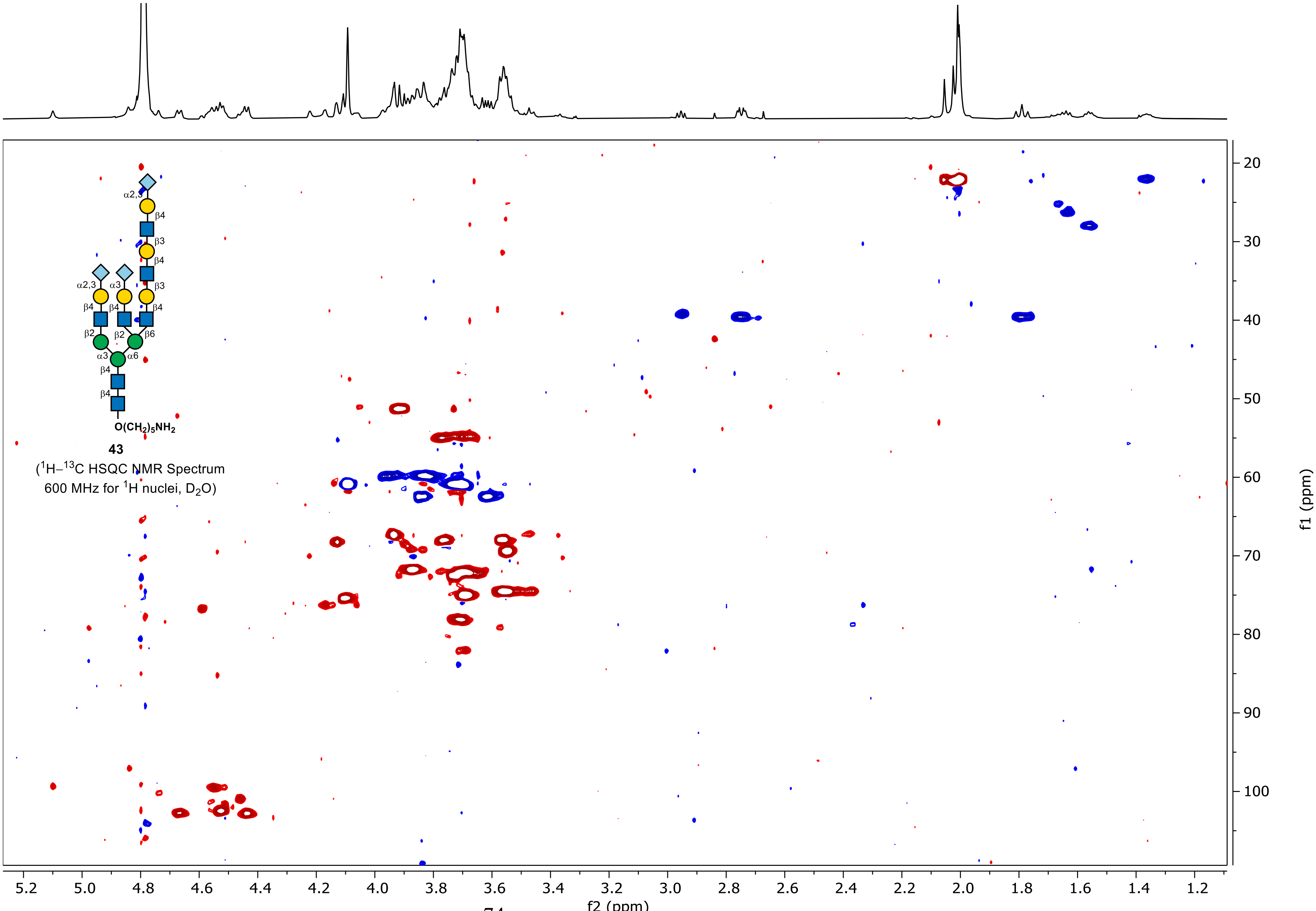

α2,3
β4
β3
β4
β3
β4
α2,3
α3
β4
β4
β2
β2
β6
α3
α6
β4
β4
O(CH2)5NH2
43
(1H–13C HSQC NMR Spectrum
600 MHz for 1H nuclei, D2O)
f2 (ppm)
f1 (ppm)

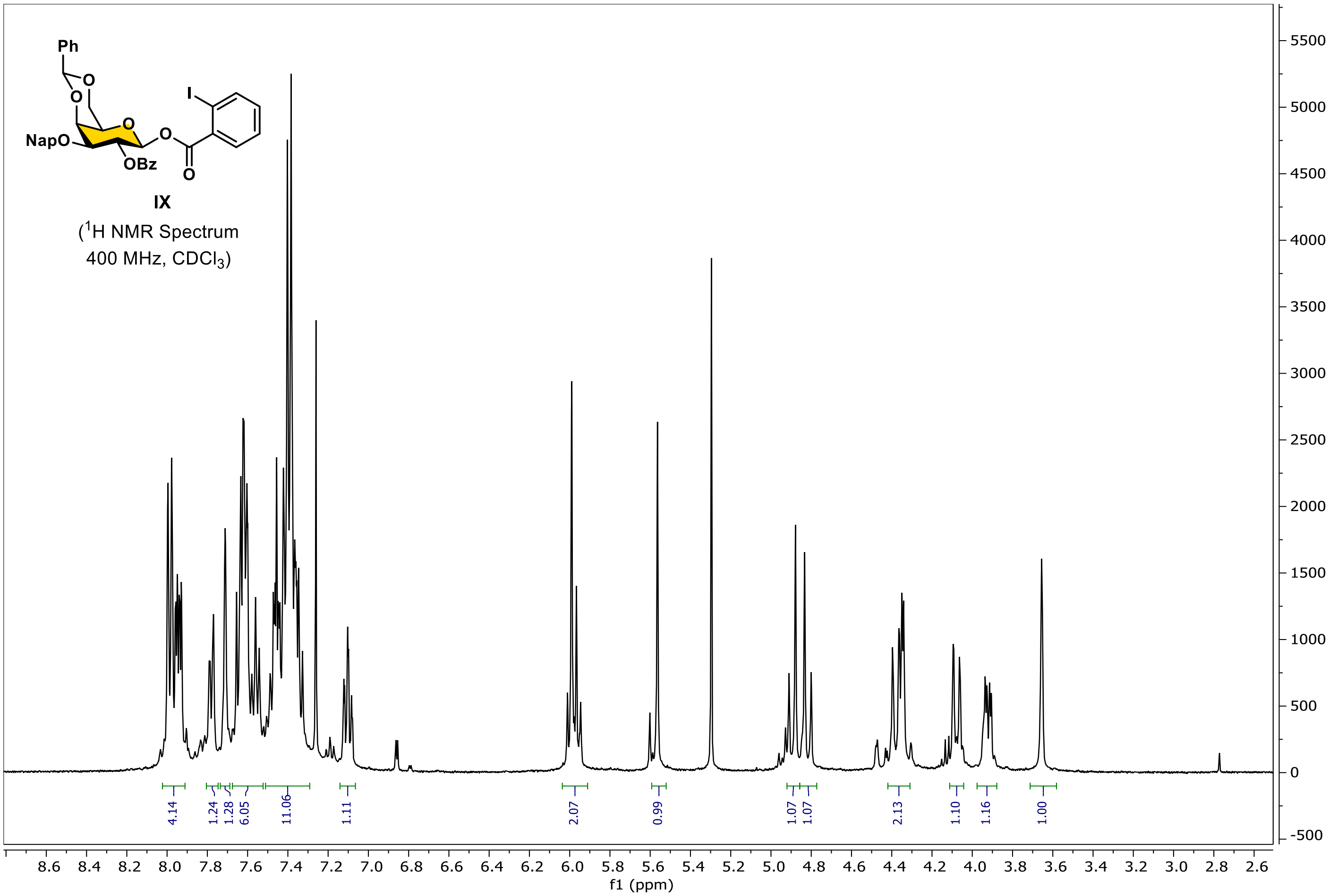
Ph
O
O
I
NapO
O
O
OBz
O
IX
(^{1}H NMR Spectrum
400 MHz, $CDCl_3$)
4.14
1.24
1.28
6.05
11.06
1.11
2.07
0.99
1.07
1.07
2.13
1.10
1.16
1.00
f1 (ppm)

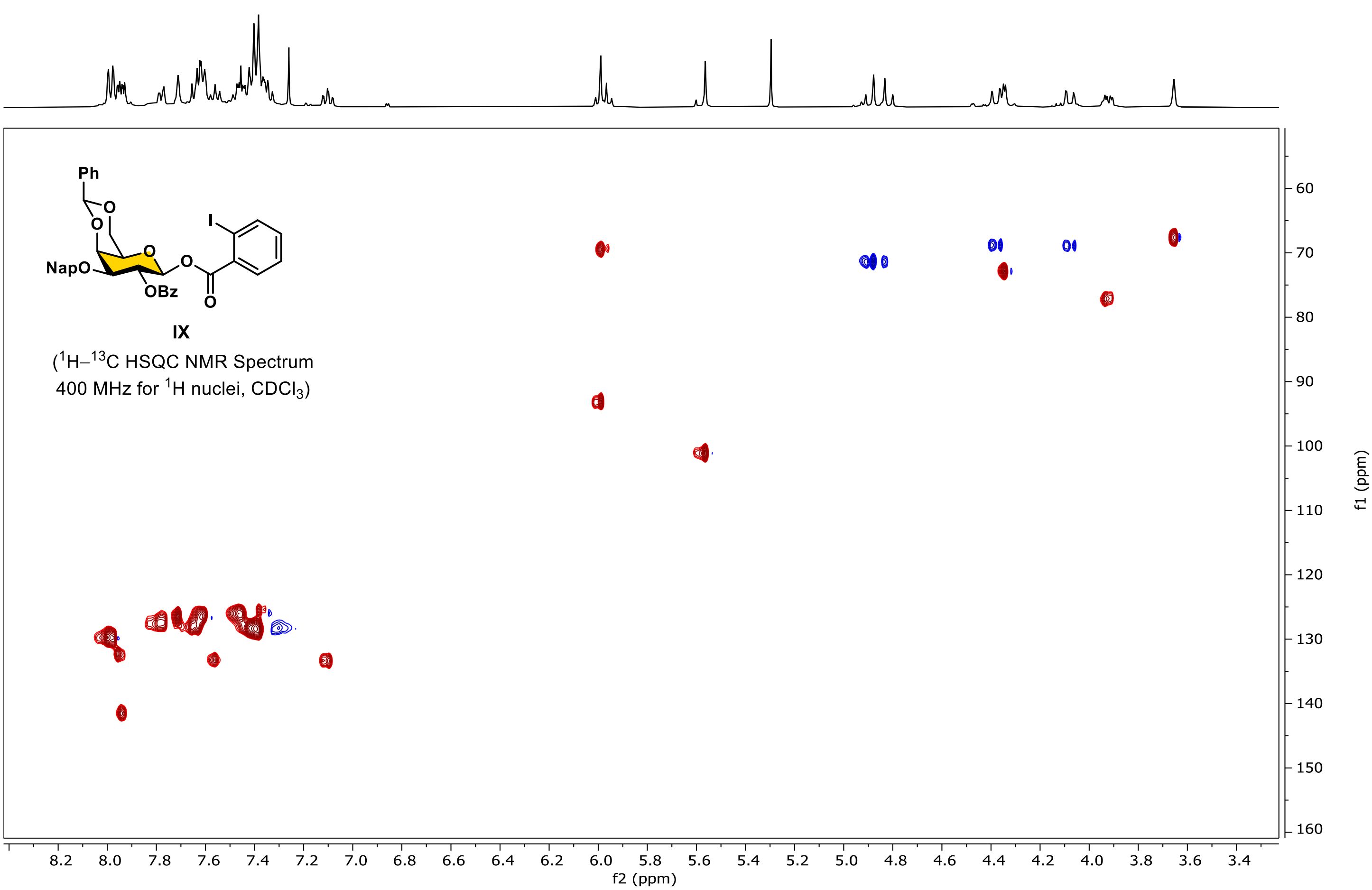
Ph
O
O
I
NapO
O
O
OBz
O
IX
(^{1}H–^{13}C HSQC NMR Spectrum
400 MHz for ^{1}H nuclei, $CDCl_3$)
f2 (ppm)
f1 (ppm)

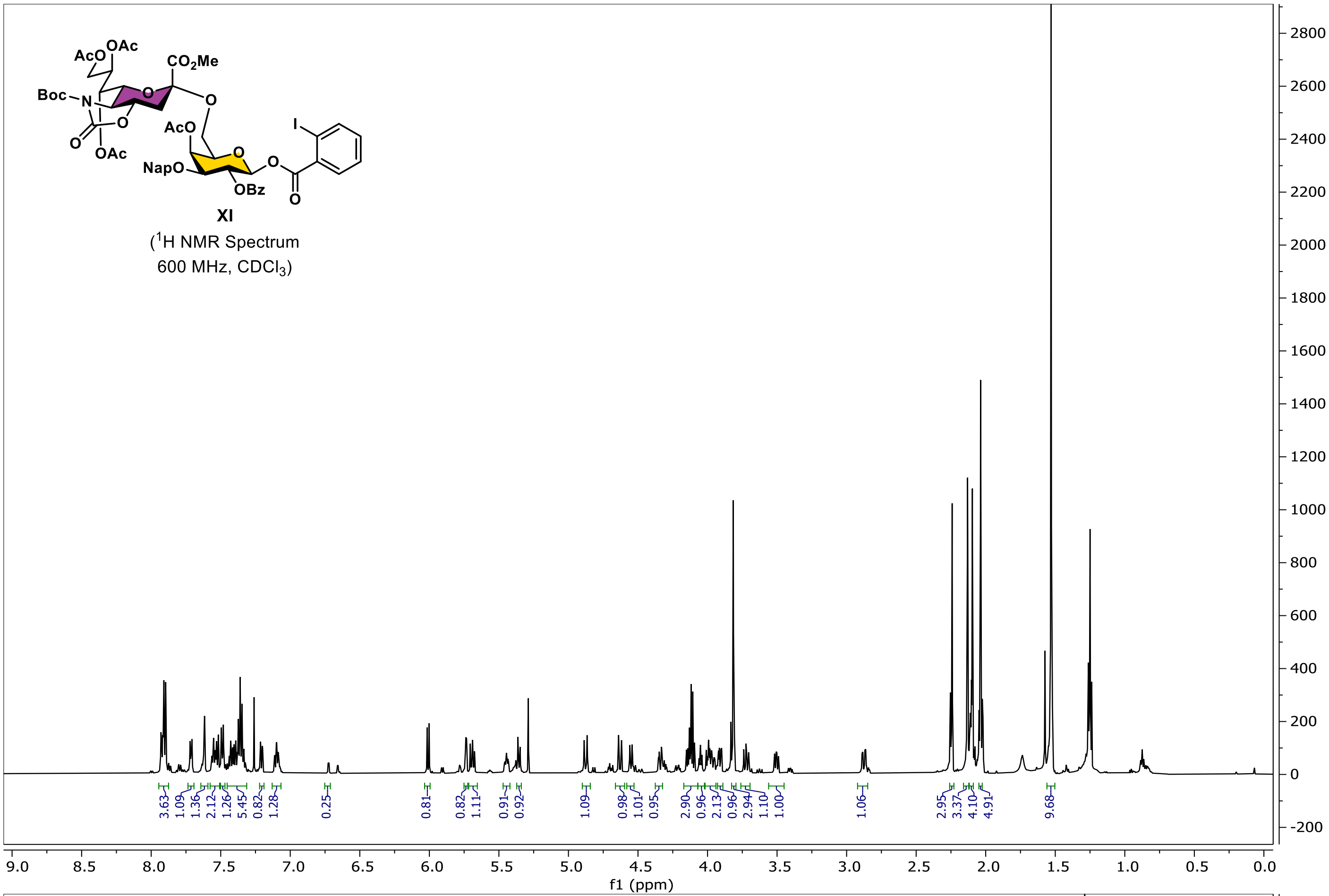
AcO
OAc
CO2Me
Boc
N
O
O
O
AcO
OAc
I
NapO
OBz
O
XI
(1H NMR Spectrum
600 MHz, CDCl3)
3.63
1.09
1.36
2.12
1.26
5.45
0.82
1.28
0.25
0.81
0.82
1.11
0.91
0.92
1.09
0.98
1.01
0.95
2.90
0.96
2.13
0.96
2.94
1.10
1.00
1.06
2.95
3.37
4.10
4.91
9.68
f1 (ppm)

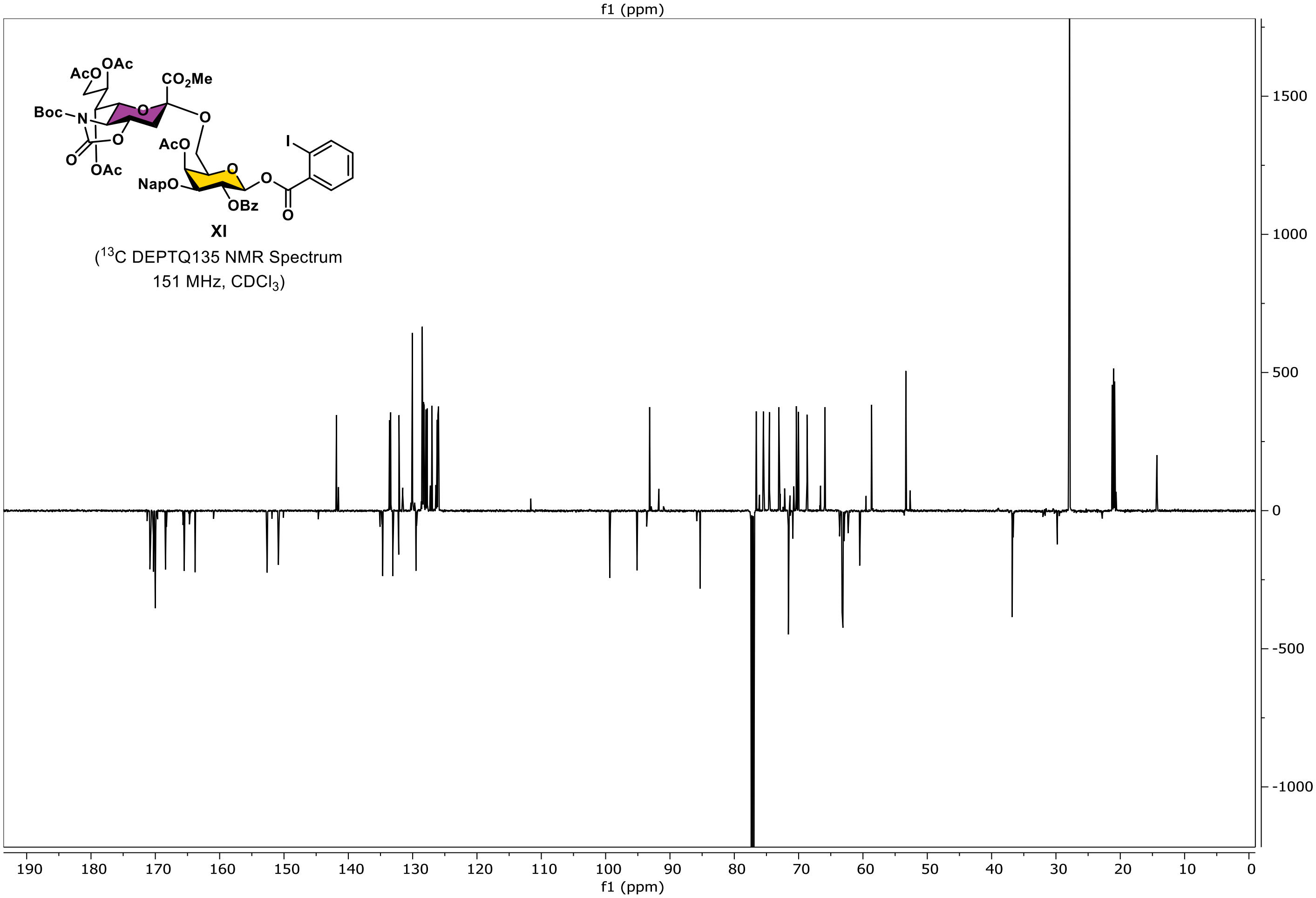
AcO
OAc
CO2Me
Boc
N
O
O
O
AcO
OAc
I
NapO
OBz
O
XI
(13C DEPTQ135 NMR Spectrum
151 MHz, CDCl3)
f1 (ppm)

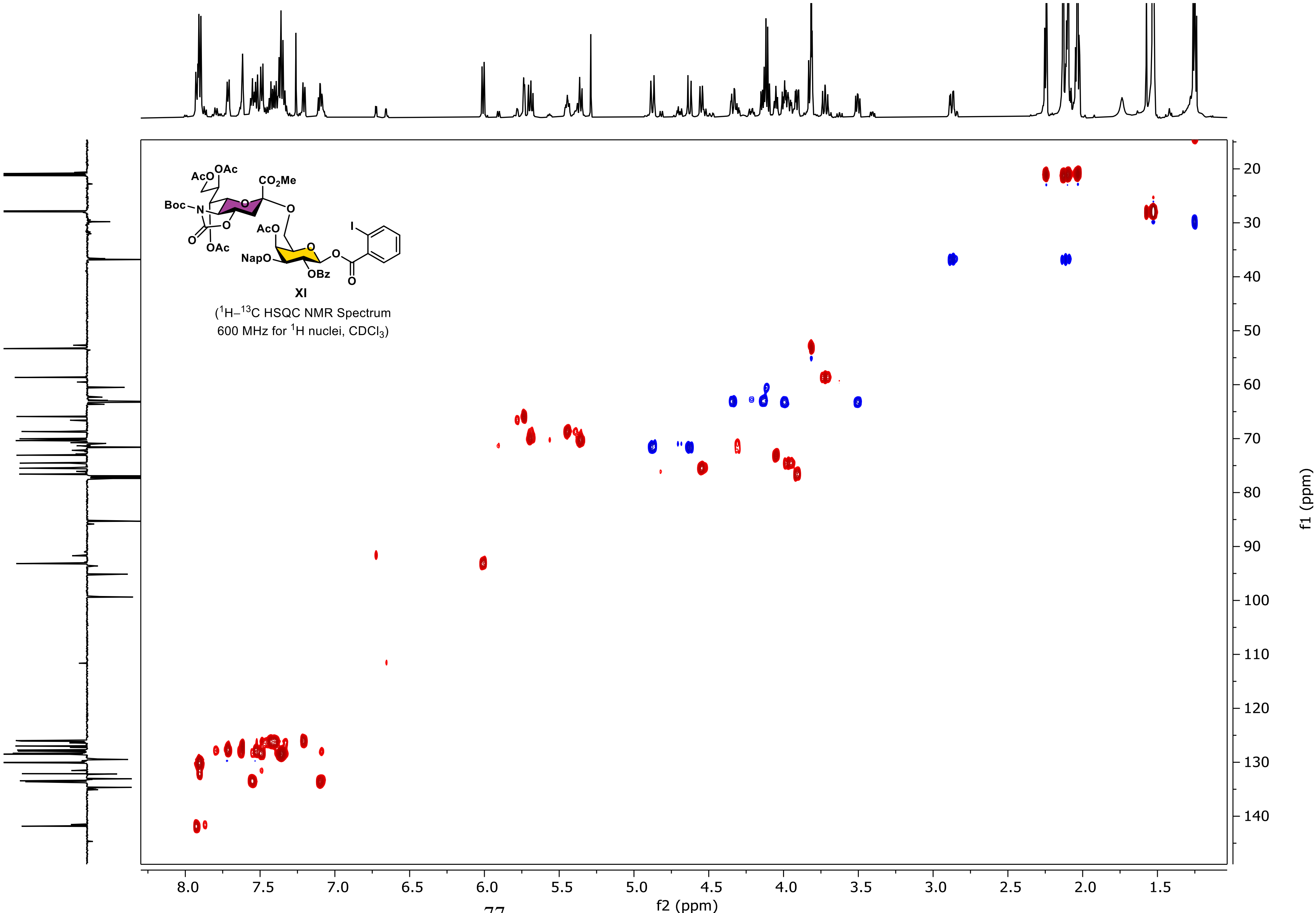
XI
(1H–13C HSQC NMR Spectrum
600 MHz for 1H nuclei, CDCl3)
f2 (ppm)
f1 (ppm)

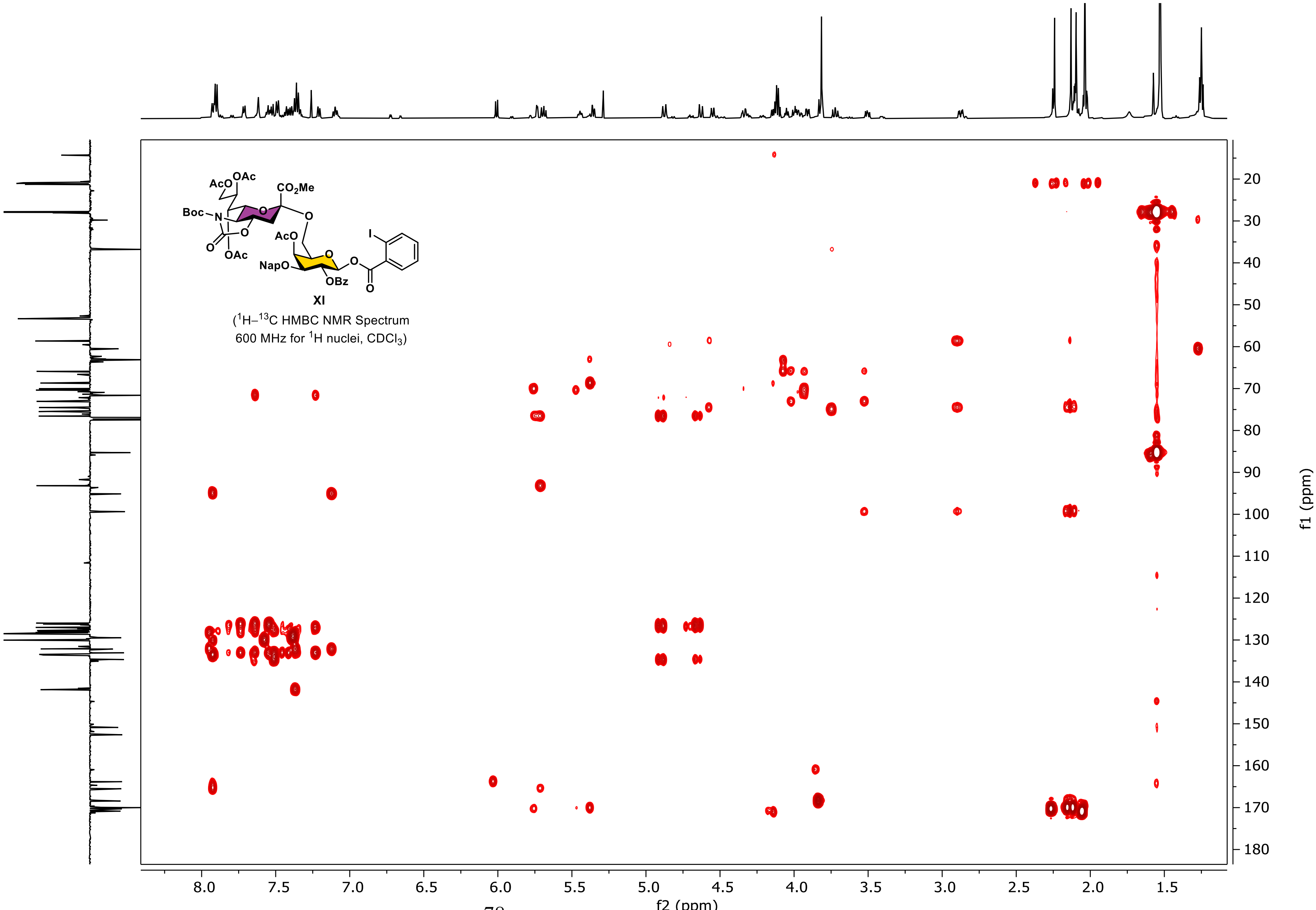
AcO
OAc
CO2Me
Boc
N
O
O
AcO
O
I
O
OAc
NapO
O
OBz
O
XI
(1H–13C HMBC NMR Spectrum
600 MHz for 1H nuclei, CDCl3)
f2 (ppm)
f1 (ppm)

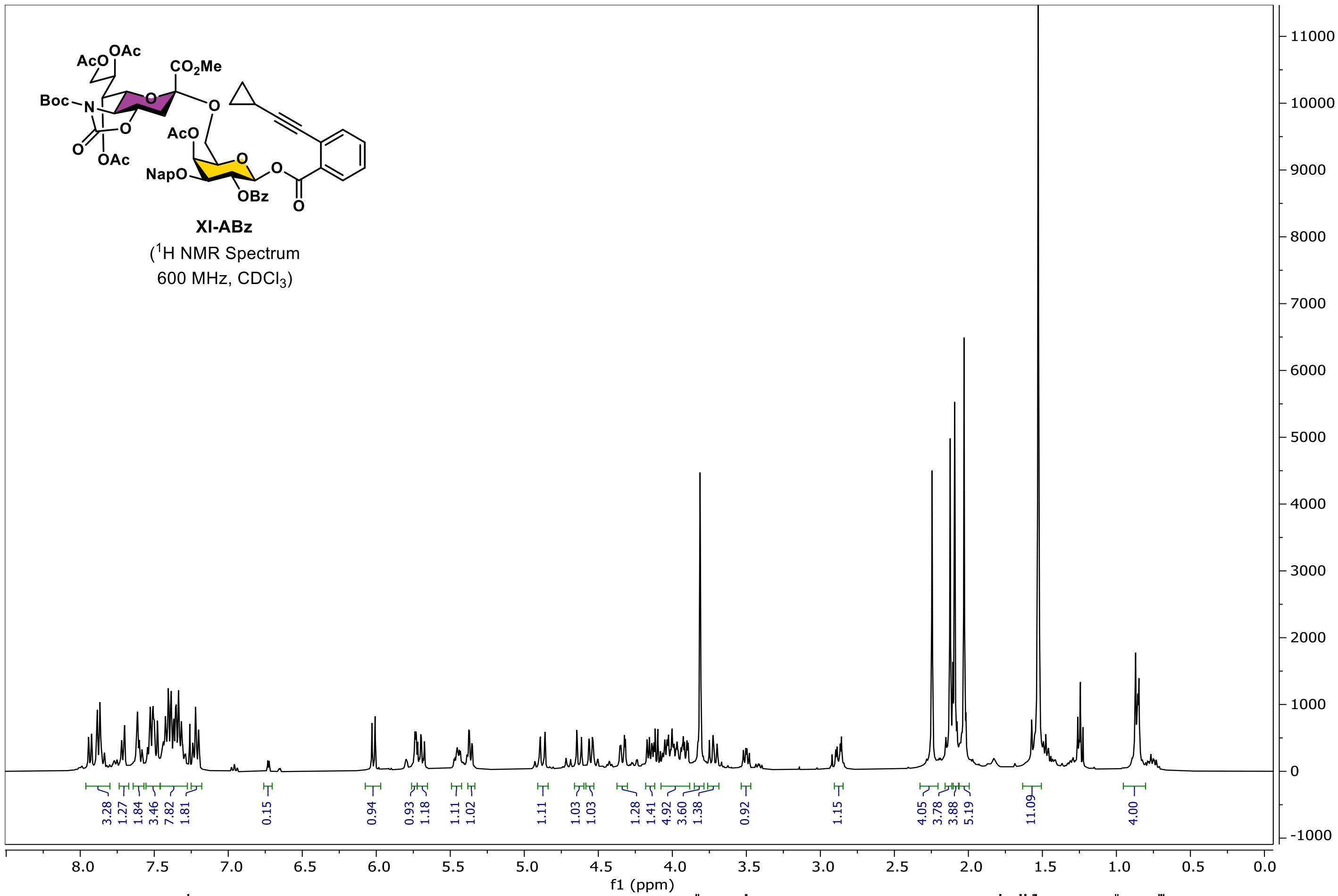

XI-ABz
(¹H NMR Spectrum
600 MHz, CDCl3)
f1 (ppm)

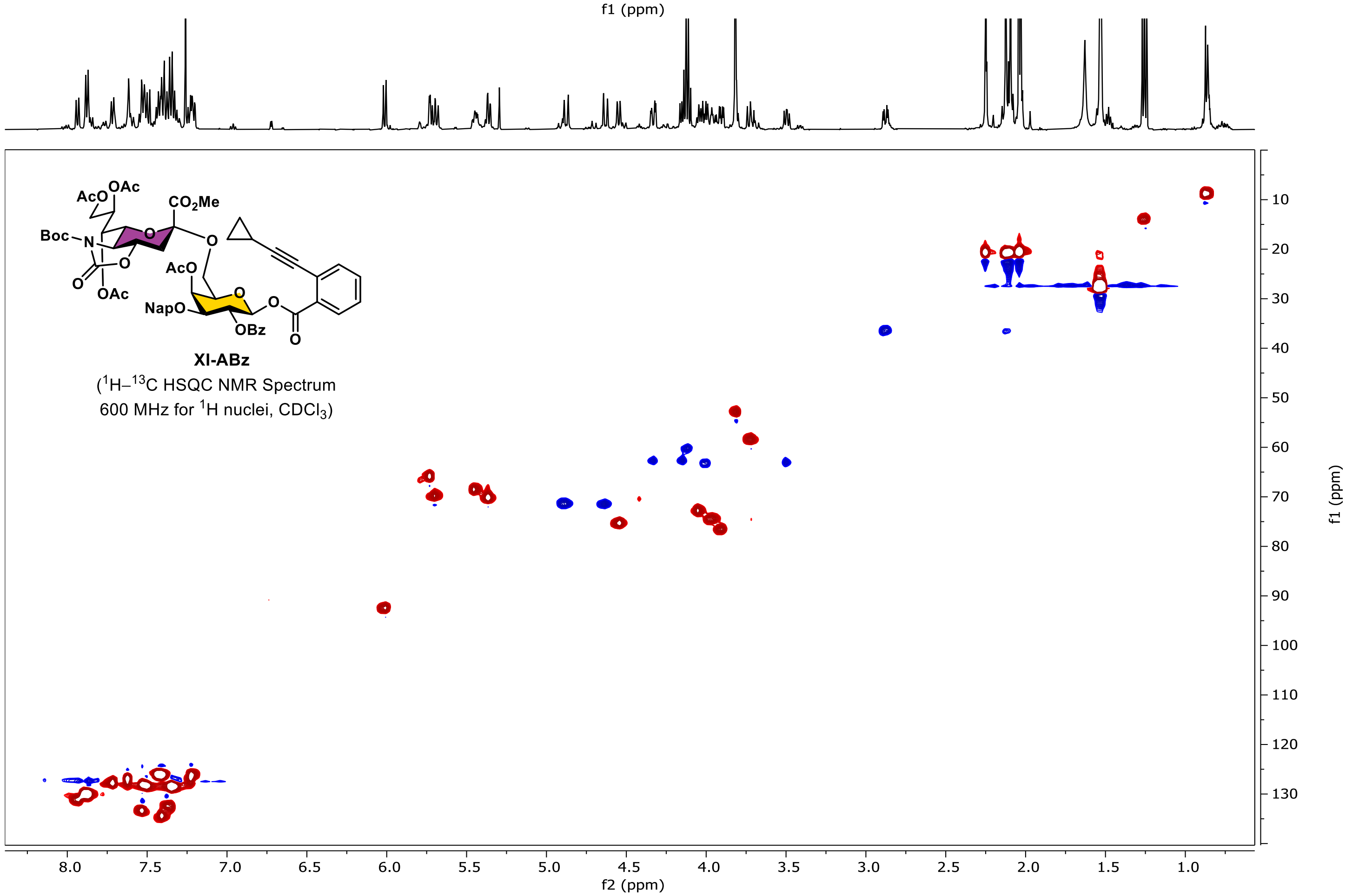

XI-ABz
(¹H–¹³C HSQC NMR Spectrum
600 MHz for ¹H nuclei, CDCl3)
f2 (ppm)
f1 (ppm)

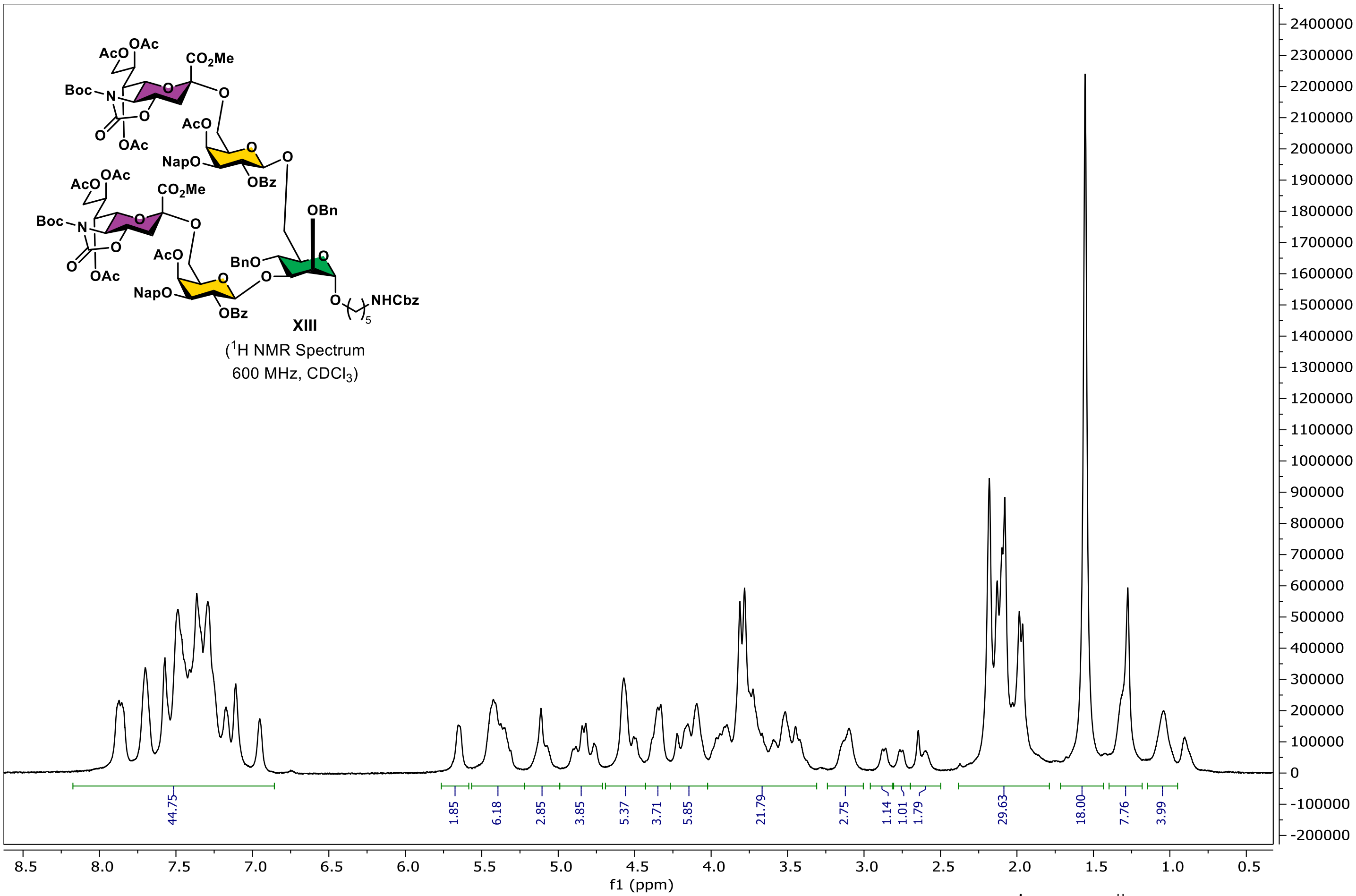
AcO
OAc
CO2Me
Boc
N
O
OAc
AcO
NapO
OBz
OBn
BnO
NHCbz
5
XIII
(1H NMR Spectrum
600 MHz, CDCl3)
44.75
1.85
6.18
2.85
3.85
5.37
3.71
5.85
21.79
2.75
1.14
1.01
1.79
29.63
18.00
7.76
3.99
f1 (ppm)

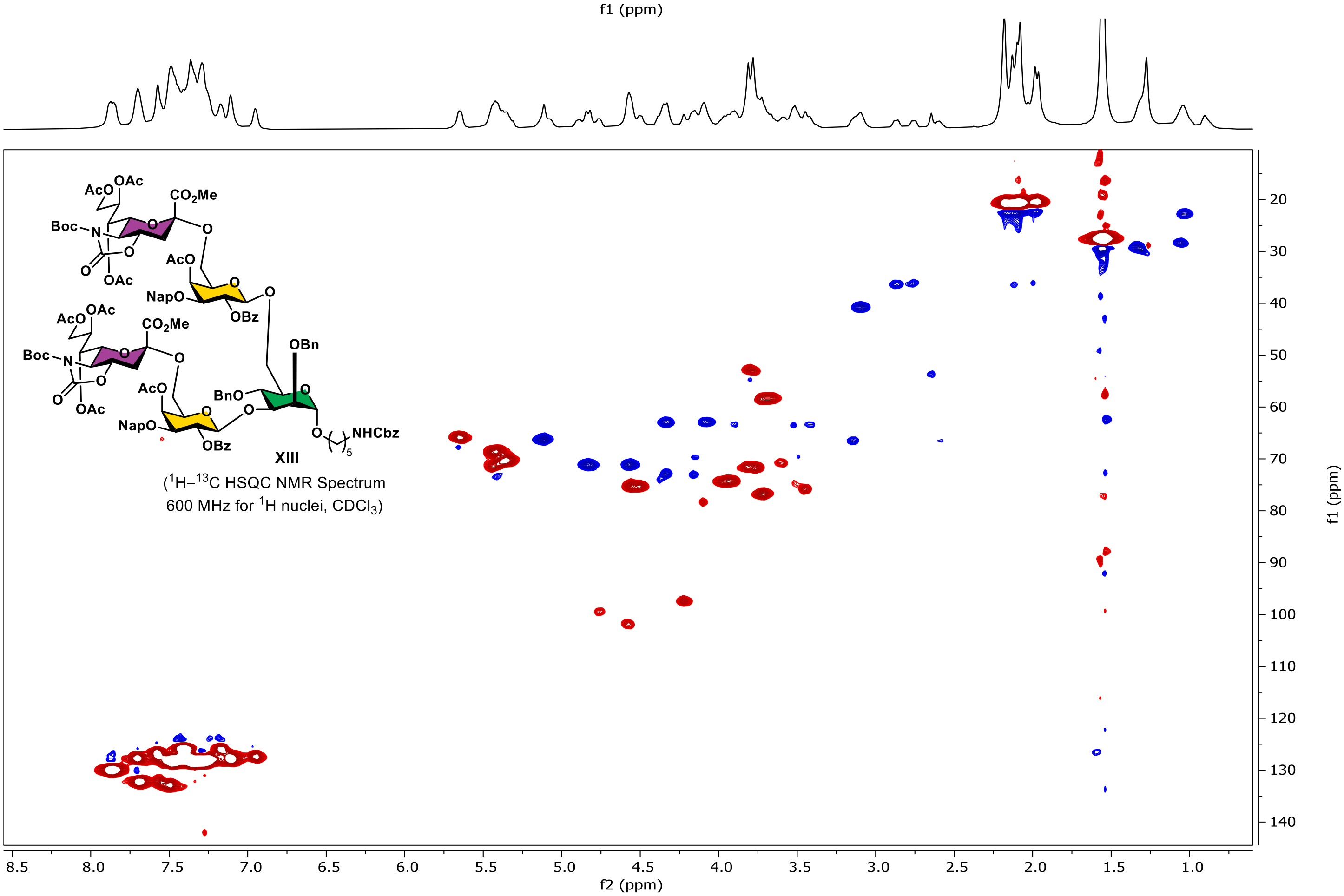
AcO
OAc
CO2Me
Boc
N
O
OAc
AcO
NapO
OBz
OBn
BnO
NHCbz
5
XIII
(1H–13C HSQC NMR Spectrum
600 MHz for 1H nuclei, CDCl3)
f2 (ppm)
f1 (ppm)

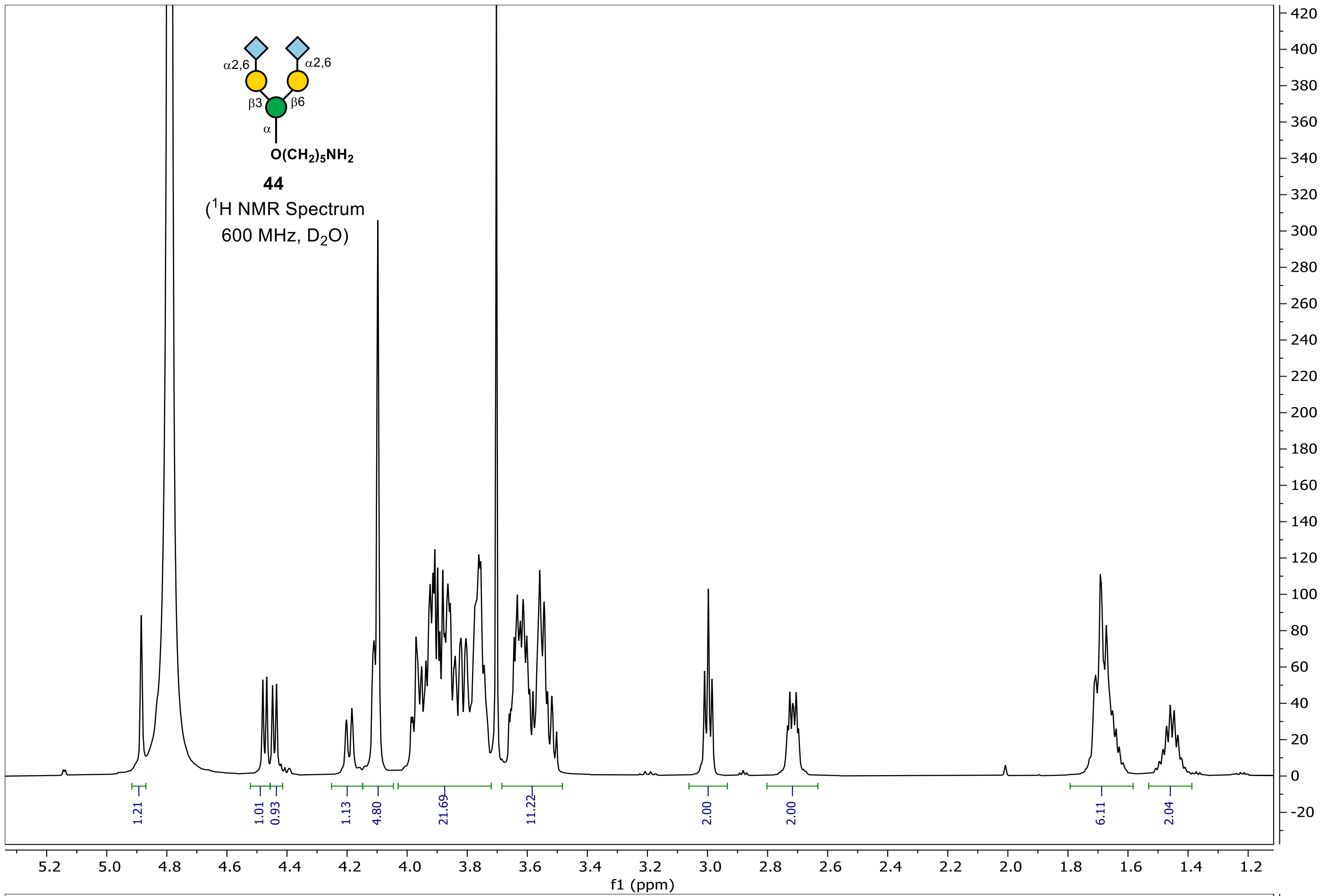

α2,6
α2,6
β3
β6
α
O(CH2)5NH2
44
(1H NMR Spectrum
600 MHz, D2O)
f1 (ppm)

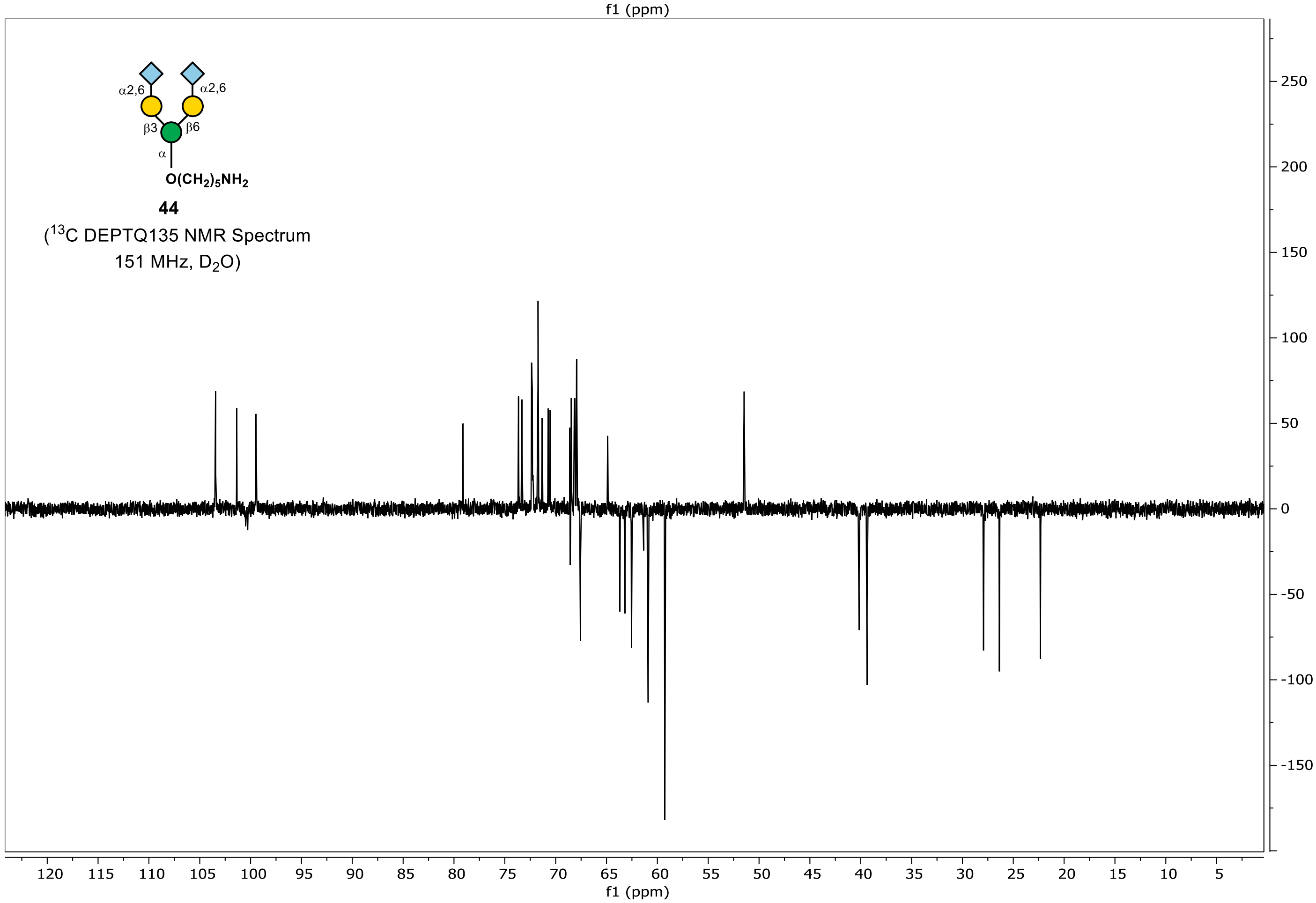

α2,6
α2,6
β3
β6
α
O(CH2)5NH2
44
(13C DEPTQ135 NMR Spectrum
151 MHz, D2O)
f1 (ppm)

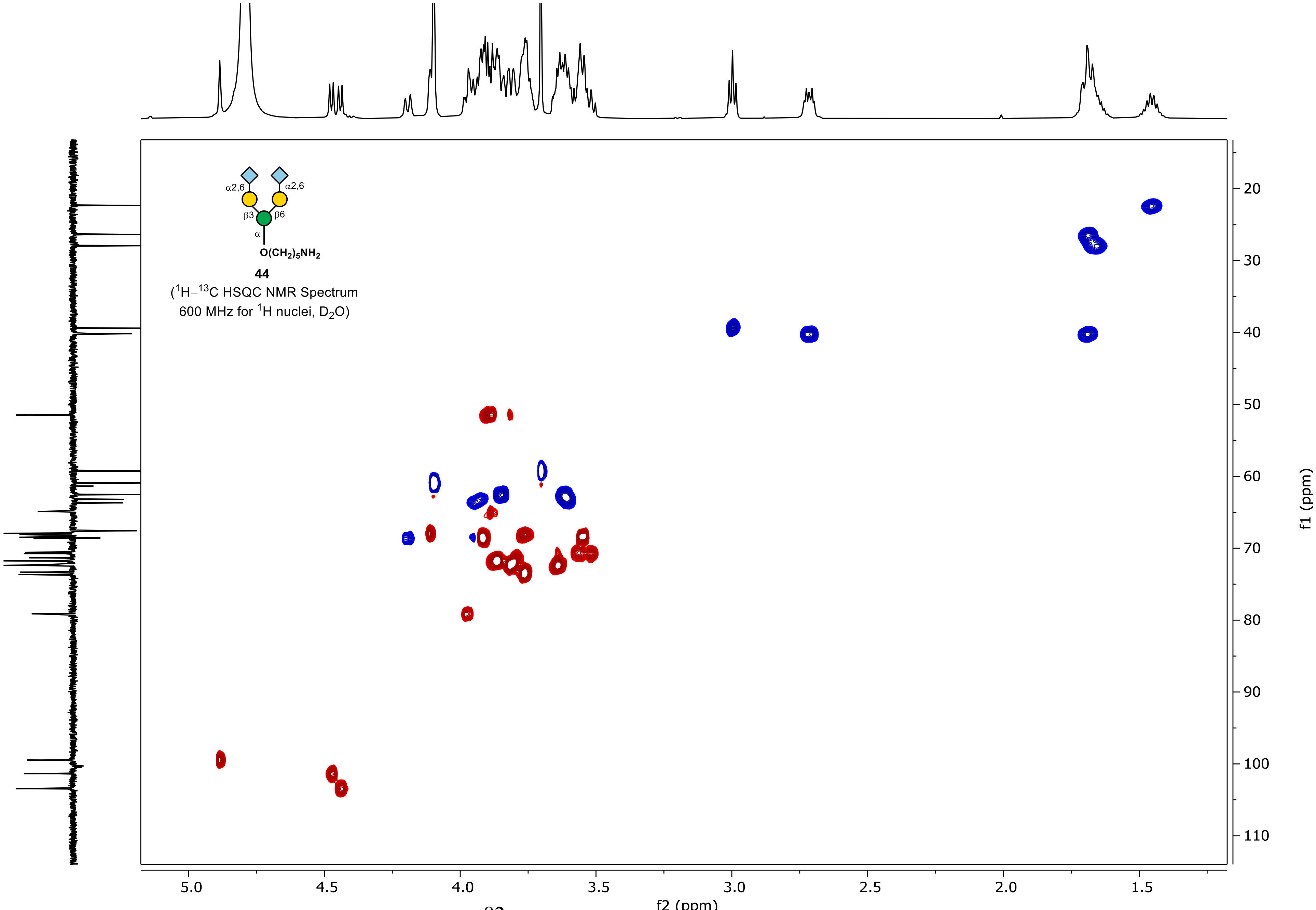
α2,6
α2,6
β3
β6
α
$O(CH_2)_5NH_2$
44
(^1H–^{13}C HSQC NMR Spectrum
600 MHz for ^1H nuclei, D_2O)
5.0
4.5
4.0
3.5
3.0
2.5
2.0
1.5
f2 (ppm)
20
30
40
50
60
70
80
90
100
110
f1 (ppm)

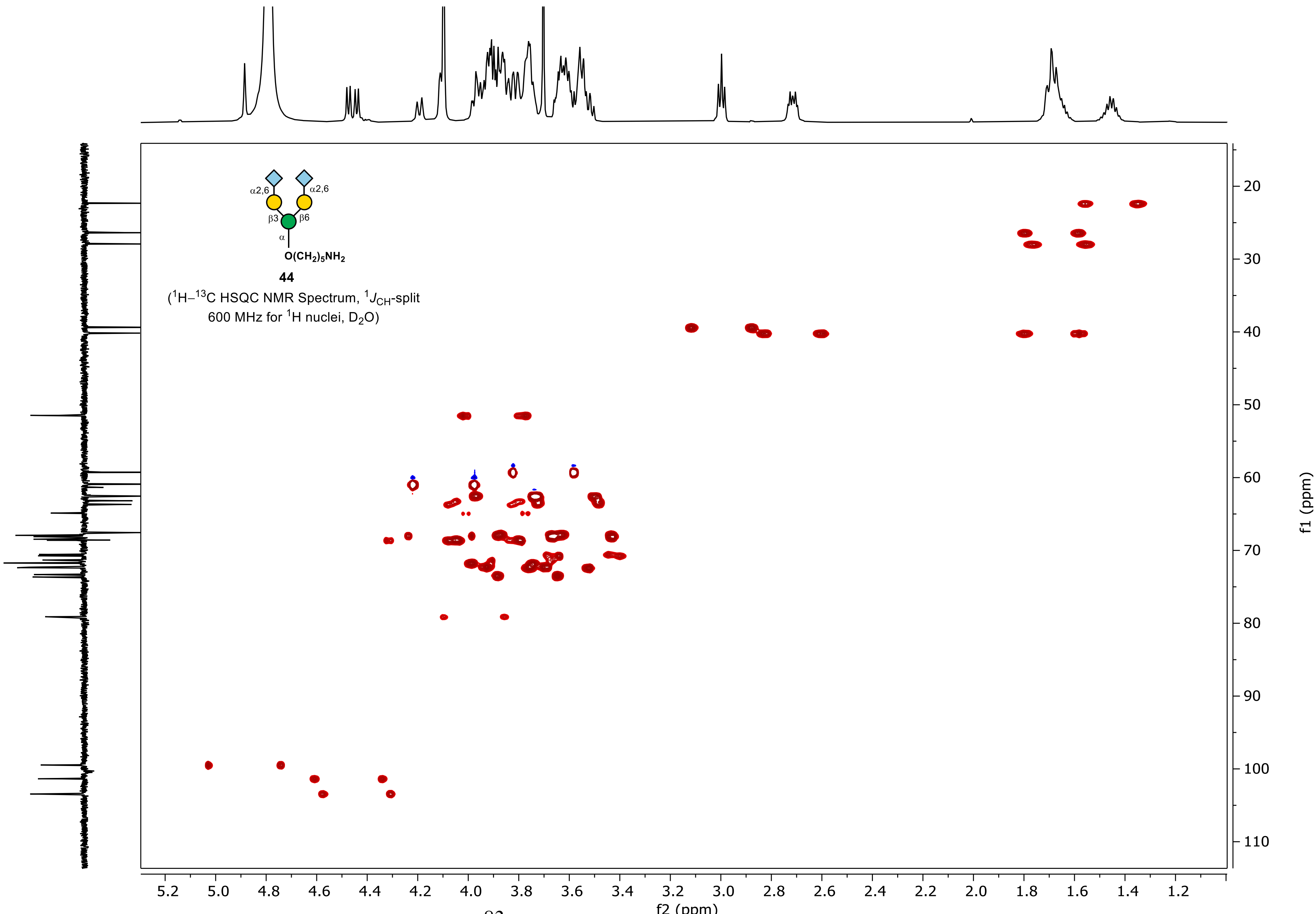
α2,6
α2,6
β3
β6
α
O(CH2)5NH2
44
(1H–13C HSQC NMR Spectrum, 1JCH-split
600 MHz for 1H nuclei, D2O)
f2 (ppm)
f1 (ppm)

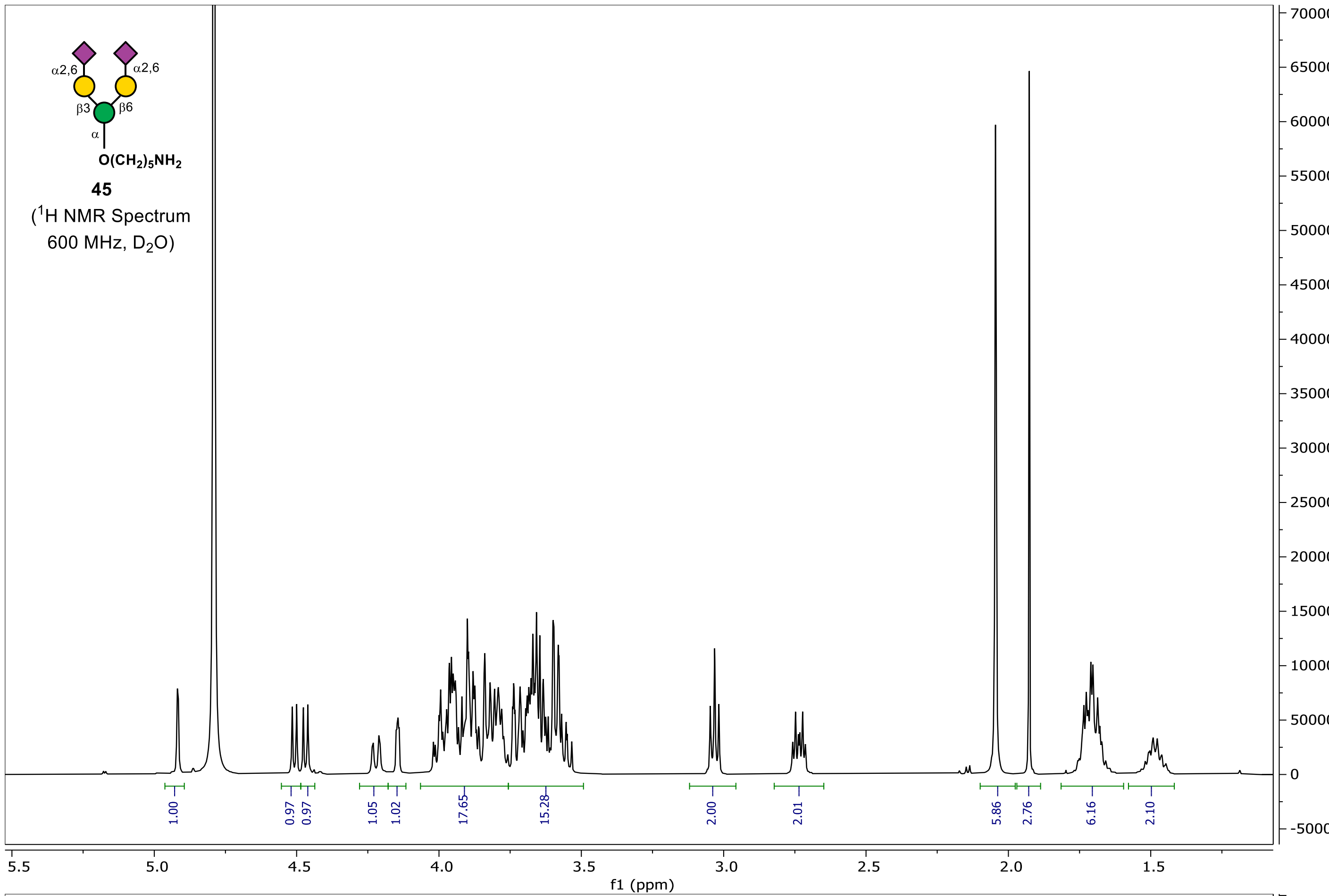
α2,6
α2,6
β3
β6
α
O(CH2)5NH2
45
(1H NMR Spectrum
600 MHz, D2O)
f1 (ppm)

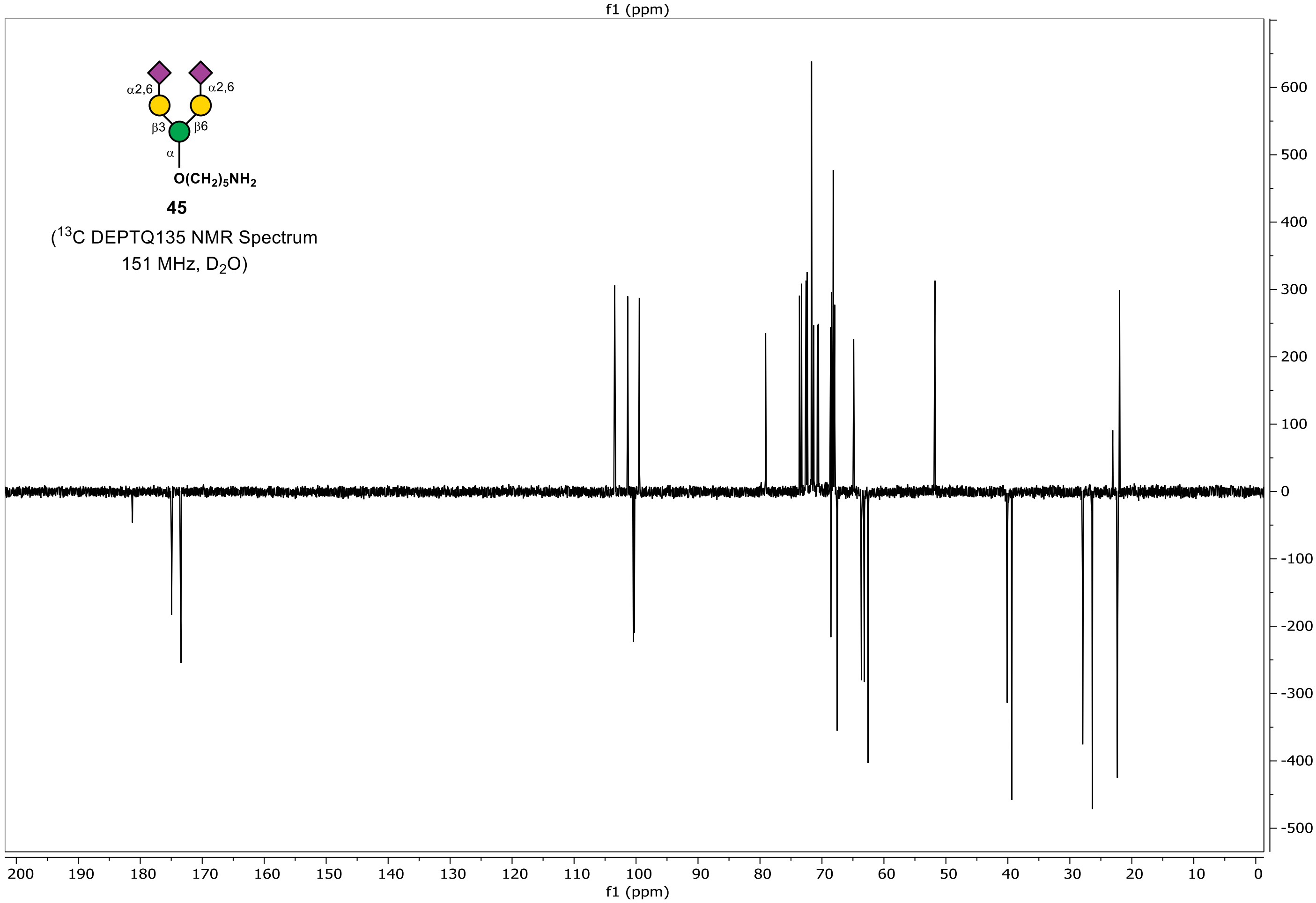
α2,6
α2,6
β3
β6
α
O(CH2)5NH2
45
(13C DEPTQ135 NMR Spectrum
151 MHz, D2O)
f1 (ppm)

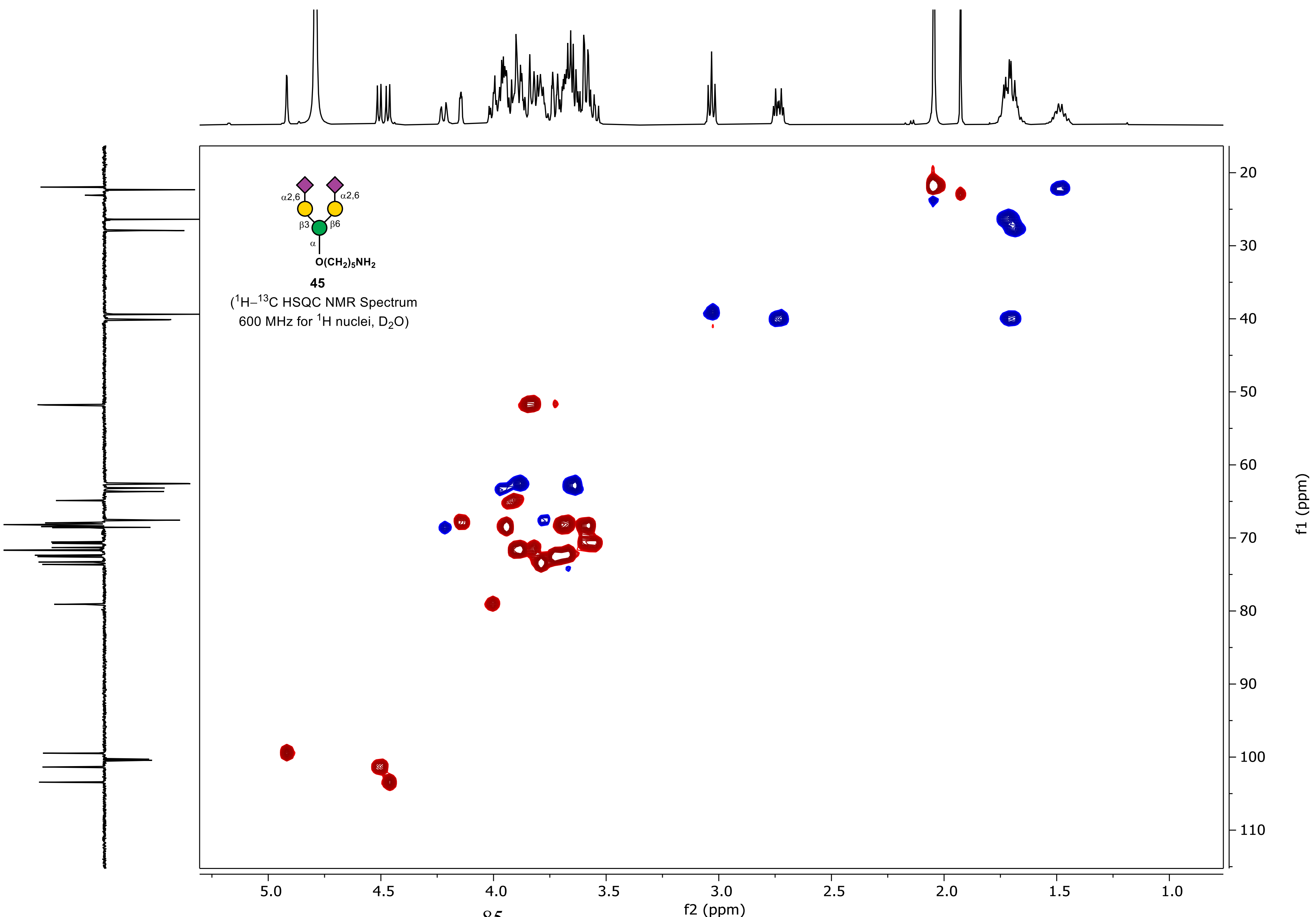
α2,6
α2,6
β3
β6
α
$O(CH_2)_5NH_2$
45
(^1H–^{13}C HSQC NMR Spectrum
600 MHz for ^1H nuclei, D_2O)
5.0
4.5
4.0
3.5
3.0
2.5
2.0
1.5
1.0
f2 (ppm)
20
30
40
50
60
70
80
90
100
110
f1 (ppm)

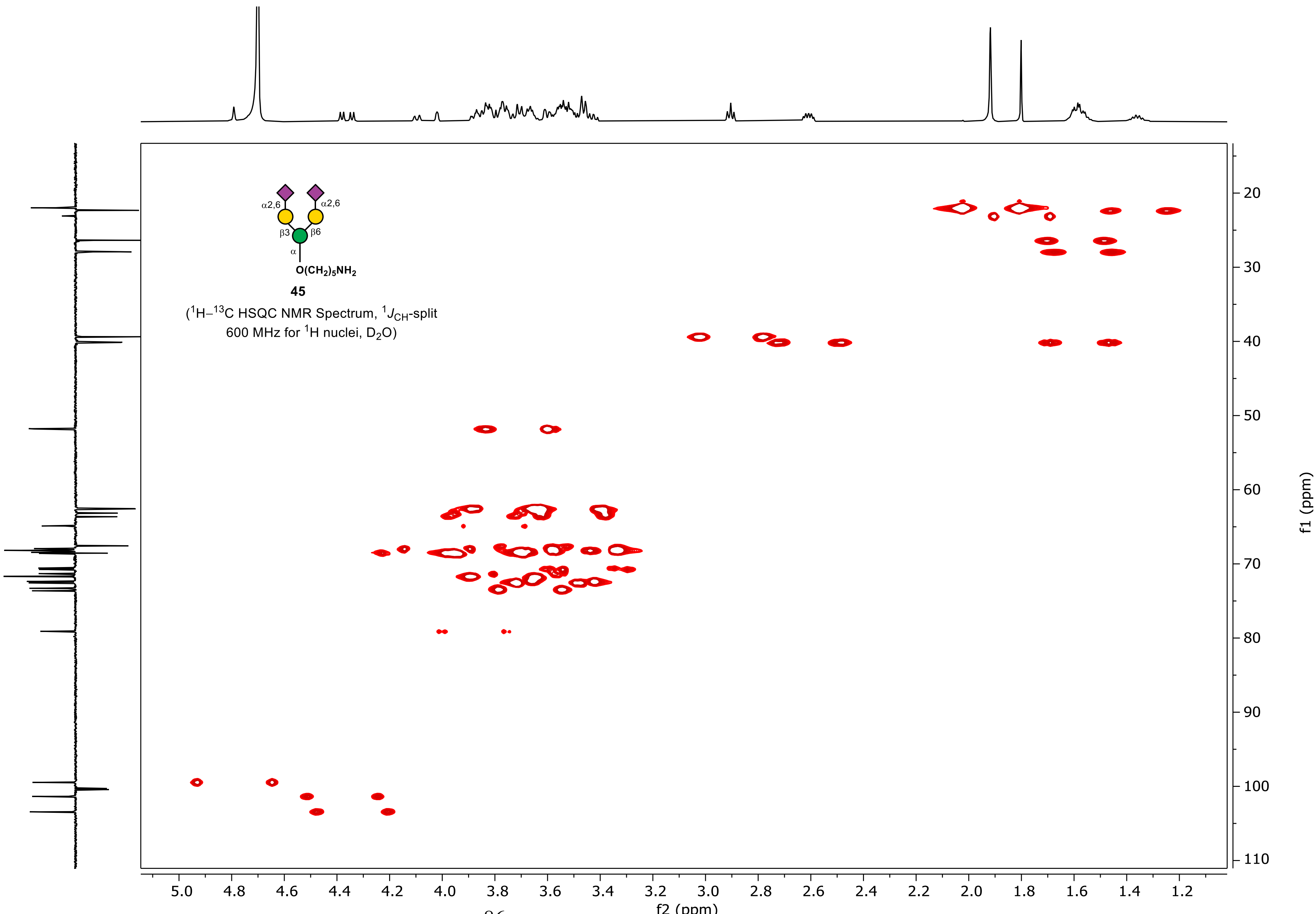
α2,6
α2,6
β3
β6
α
O(CH2)5NH2
45
(1H–13C HSQC NMR Spectrum, 1JCH-split
600 MHz for 1H nuclei, D2O)
f2 (ppm)
f1 (ppm)

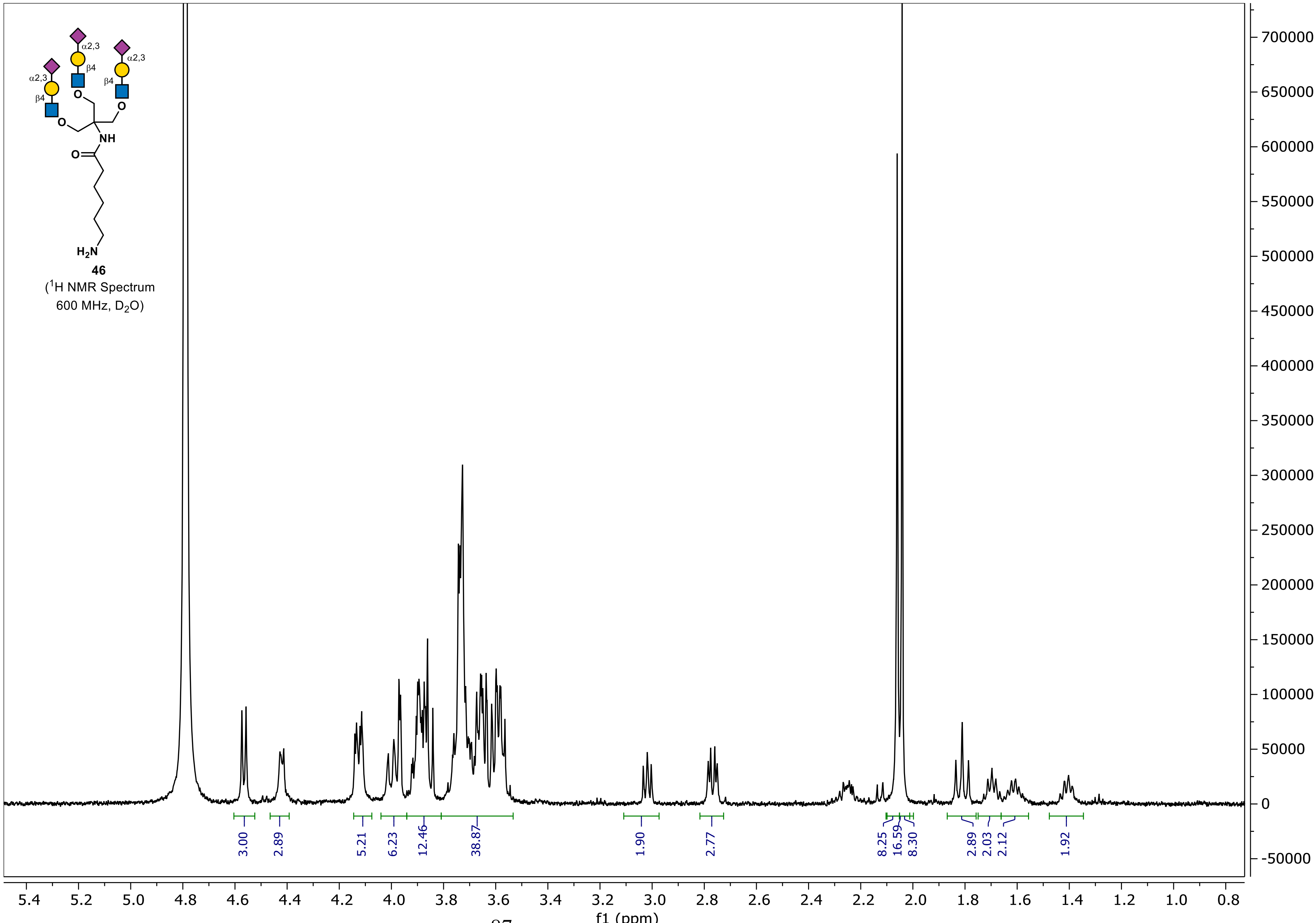
α2,3
β4
O
NH
H2N
46
(1H NMR Spectrum
600 MHz, D2O)
3.00
2.89
5.21
6.23
12.46
38.87
1.90
2.77
8.25
16.59
8.30
2.89
2.03
2.12
1.92
f1 (ppm)

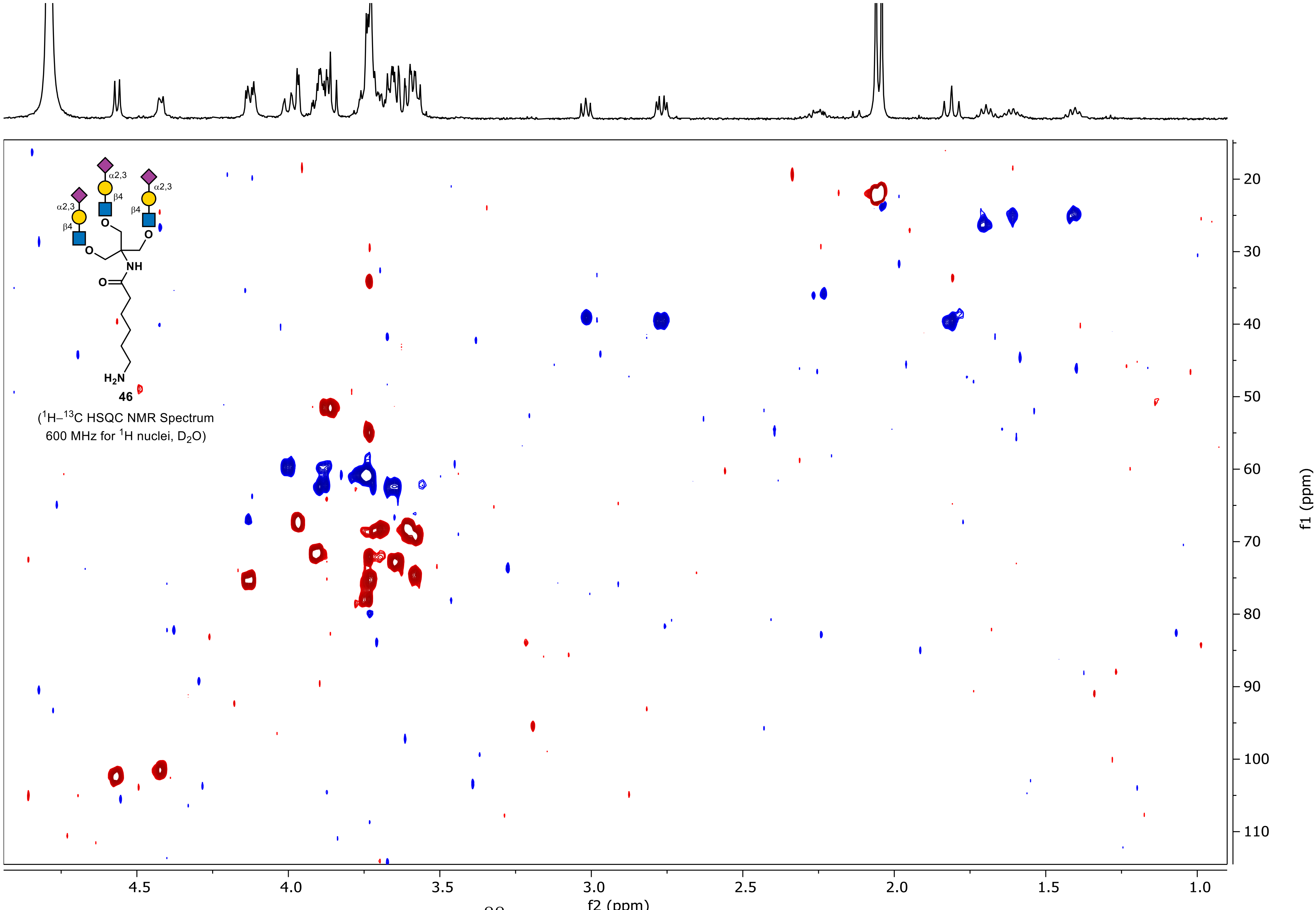
α2,3
β4
O
NH
O
H2N
46
(1H–13C HSQC NMR Spectrum
600 MHz for 1H nuclei, D2O)
f2 (ppm)
f1 (ppm)
4.5
4.0
3.5
3.0
2.5
2.0
1.5
1.0
20
30
40
50
60
70
80
90
100
110

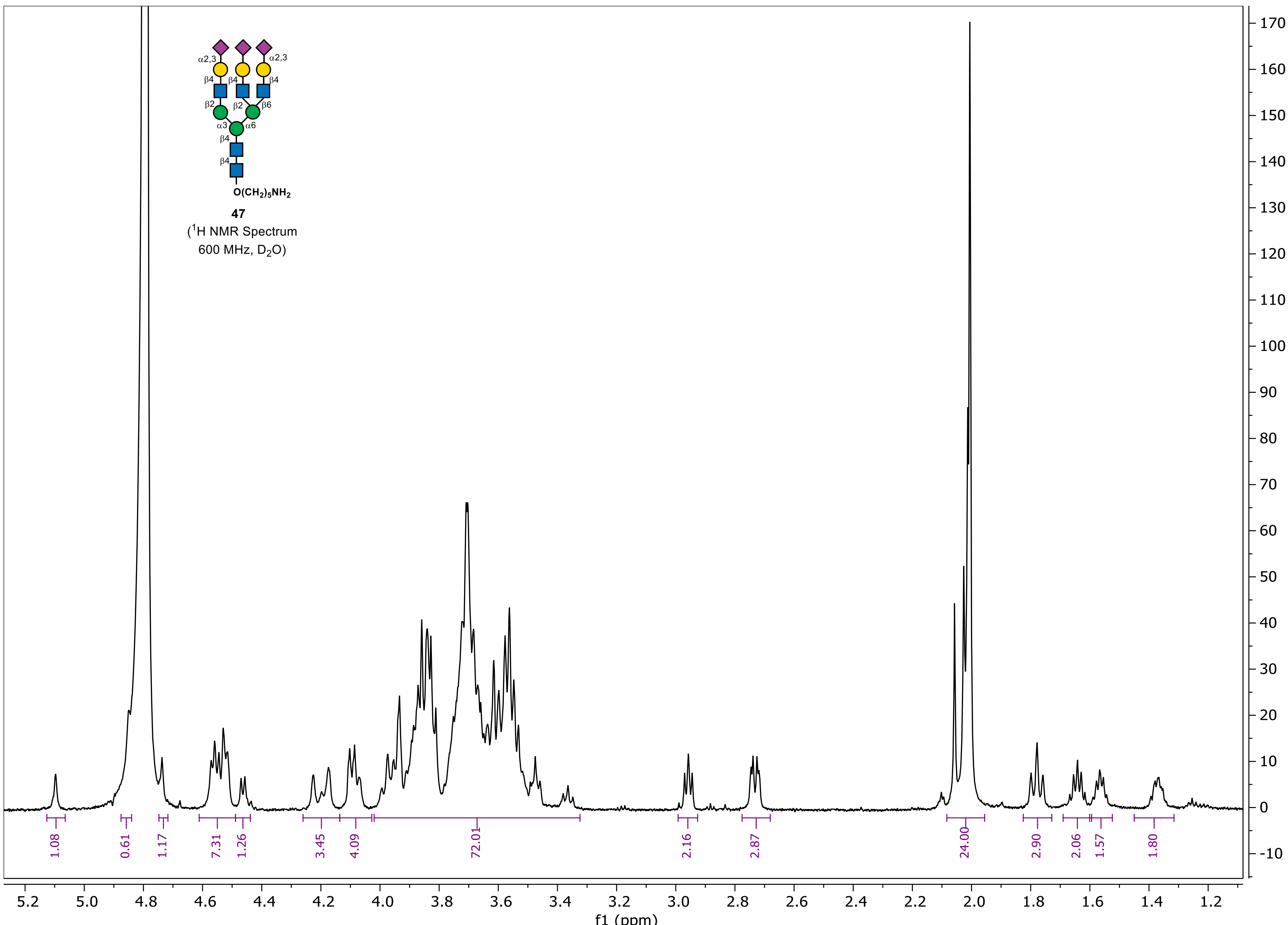
α2,3
β4
β2
β6
α3
α6
O(CH2)5NH2
47
(1H NMR Spectrum
600 MHz, D2O)
1.08
0.61
1.17
7.31
1.26
3.45
4.09
72.01
2.16
2.87
24.00
2.90
2.06
1.57
1.80
f1 (ppm)

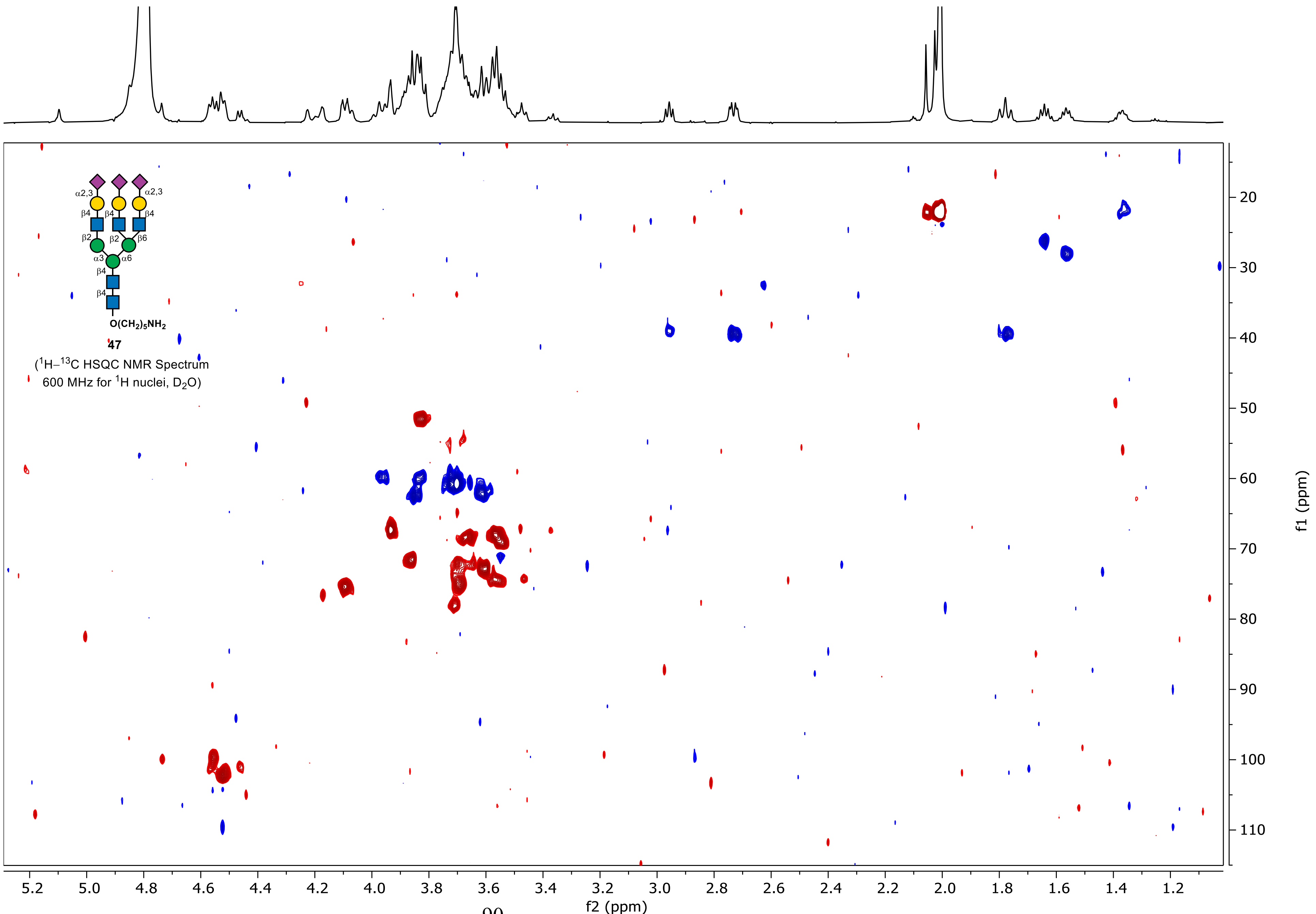
α2,3
α2,3
β4
β4
β4
β2
β2
β6
α3
α6
β4
β4
O(CH2)5NH2
47
(1H–13C HSQC NMR Spectrum
600 MHz for 1H nuclei, D2O)
f2 (ppm)
f1 (ppm)

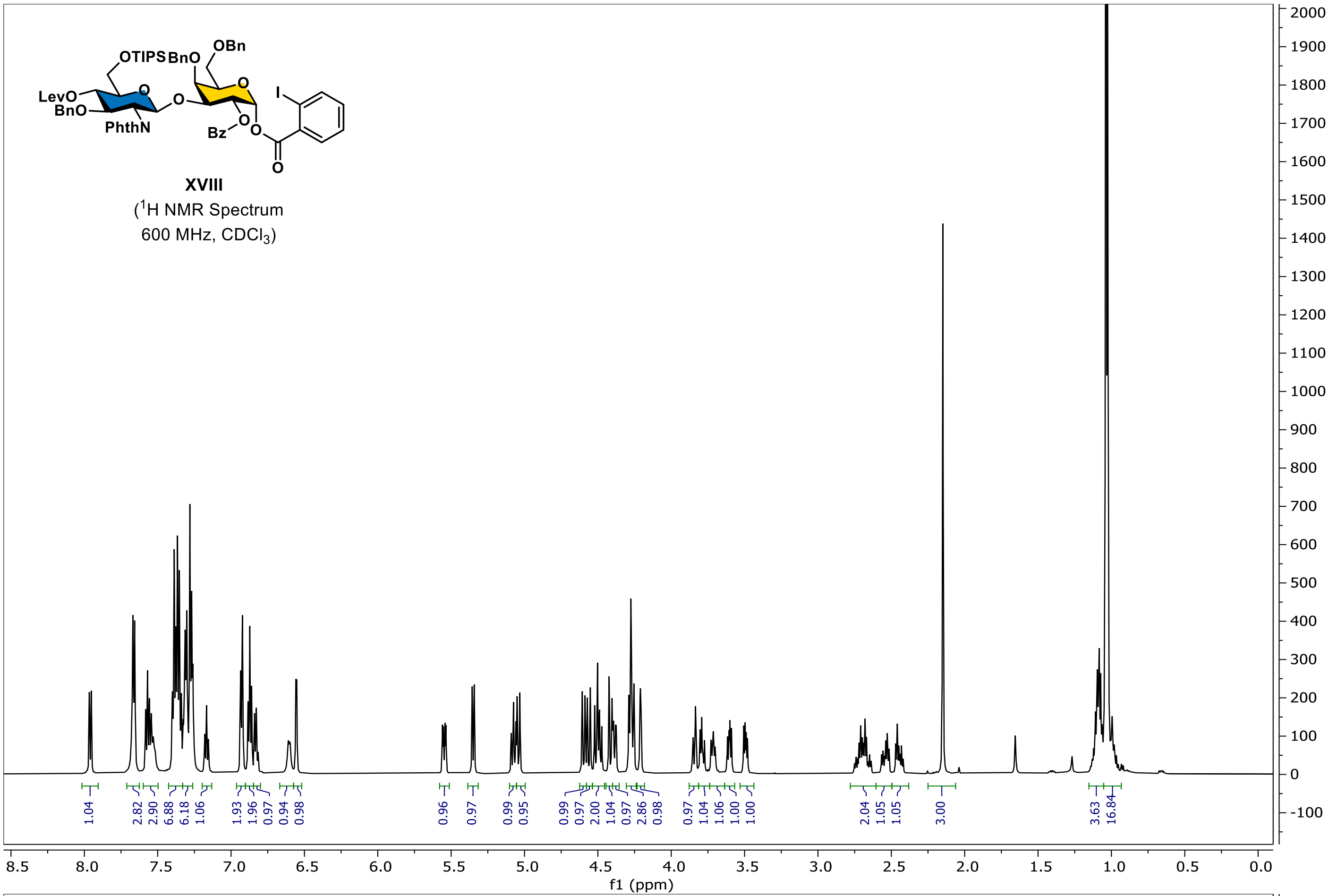
OTIPS
BnO
OBn
LevO
BnO
PhthN
Bz
I
XVIII
(1H NMR Spectrum
600 MHz, CDCl3)
f1 (ppm)

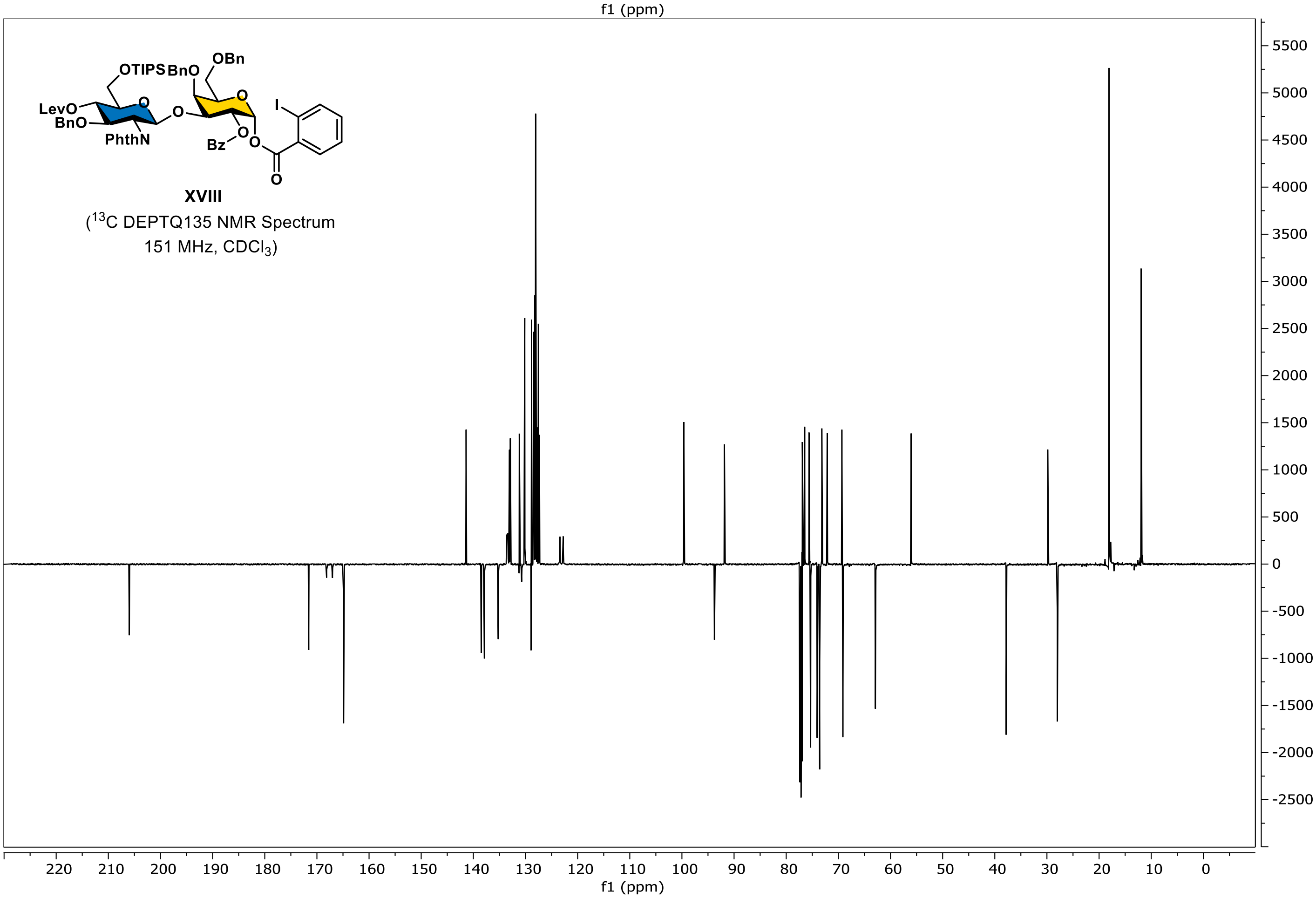
OTIPS
BnO
OBn
LevO
BnO
PhthN
Bz
I
XVIII
(13C DEPTQ135 NMR Spectrum
151 MHz, CDCl3)
f1 (ppm)

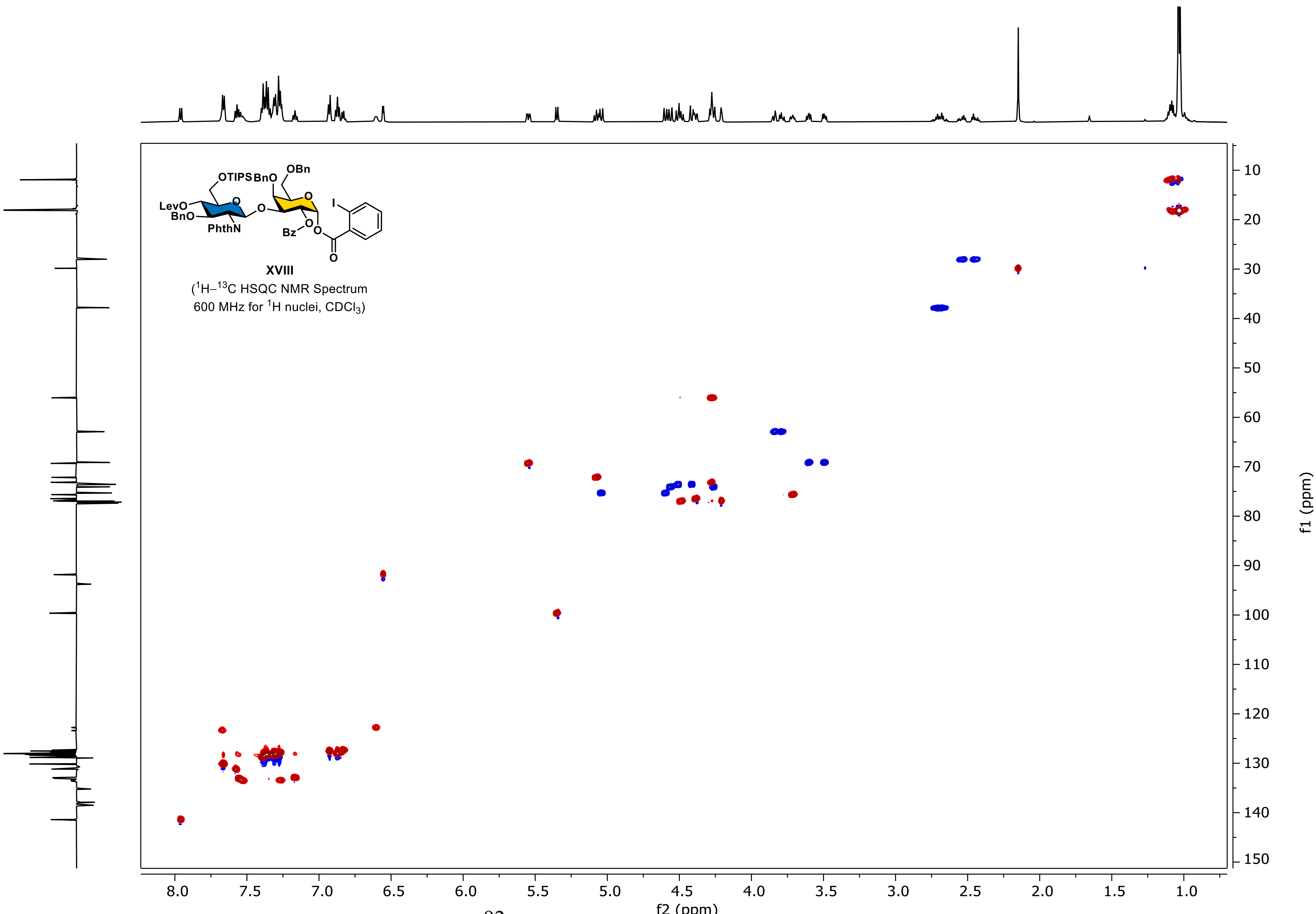
OTIPS
BnO
OBn
LevO
BnO
PhthN
Bz
I
XVIII
(^{1}H–^{13}C HSQC NMR Spectrum
600 MHz for ^{1}H nuclei, CDCl$_3$)
f2 (ppm)
f1 (ppm)

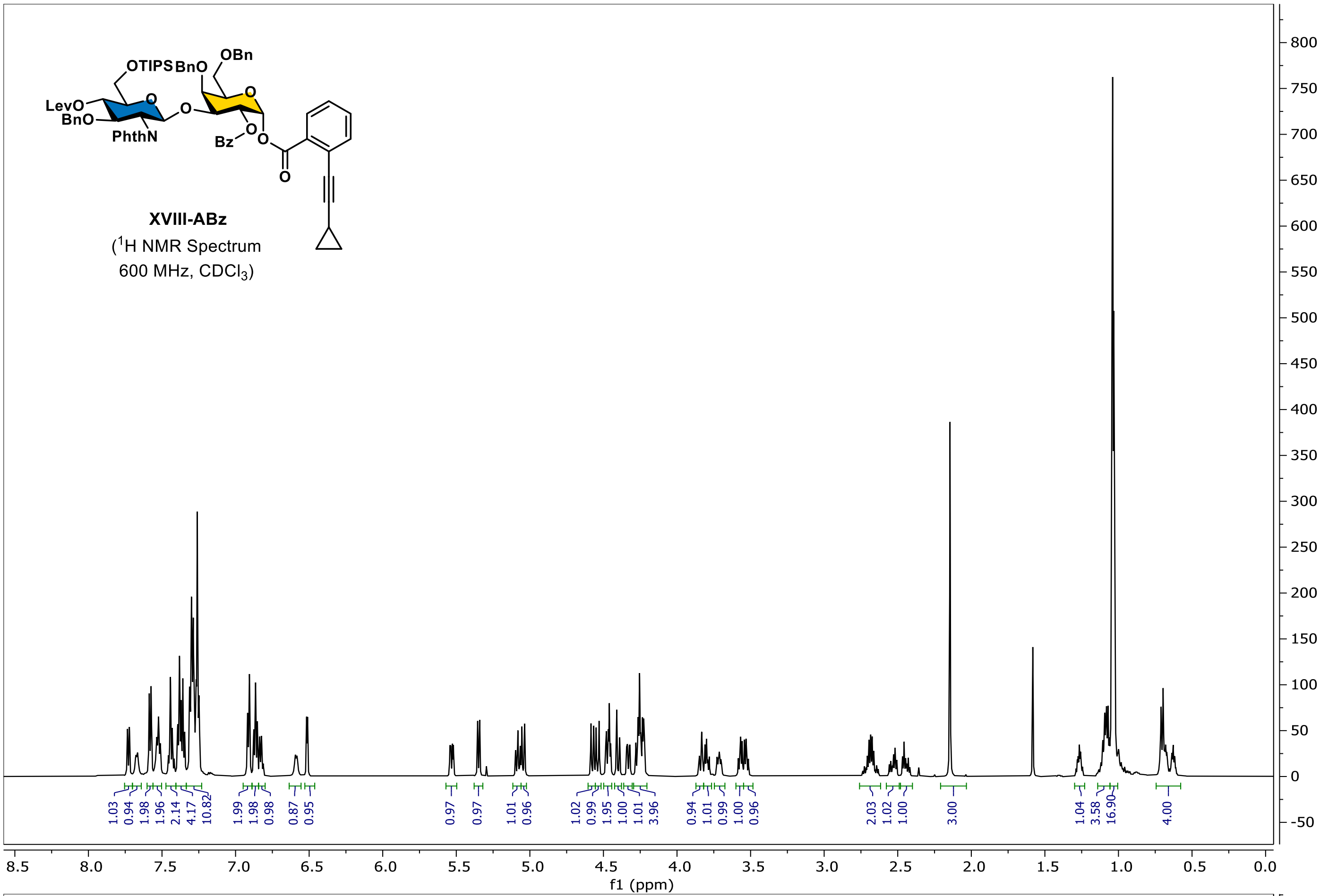
OTIPS
BnO
OBn
LevO
BnO
PhthN
Bz
XVIII-ABz
(1H NMR Spectrum
600 MHz, CDCl3)
f1 (ppm)

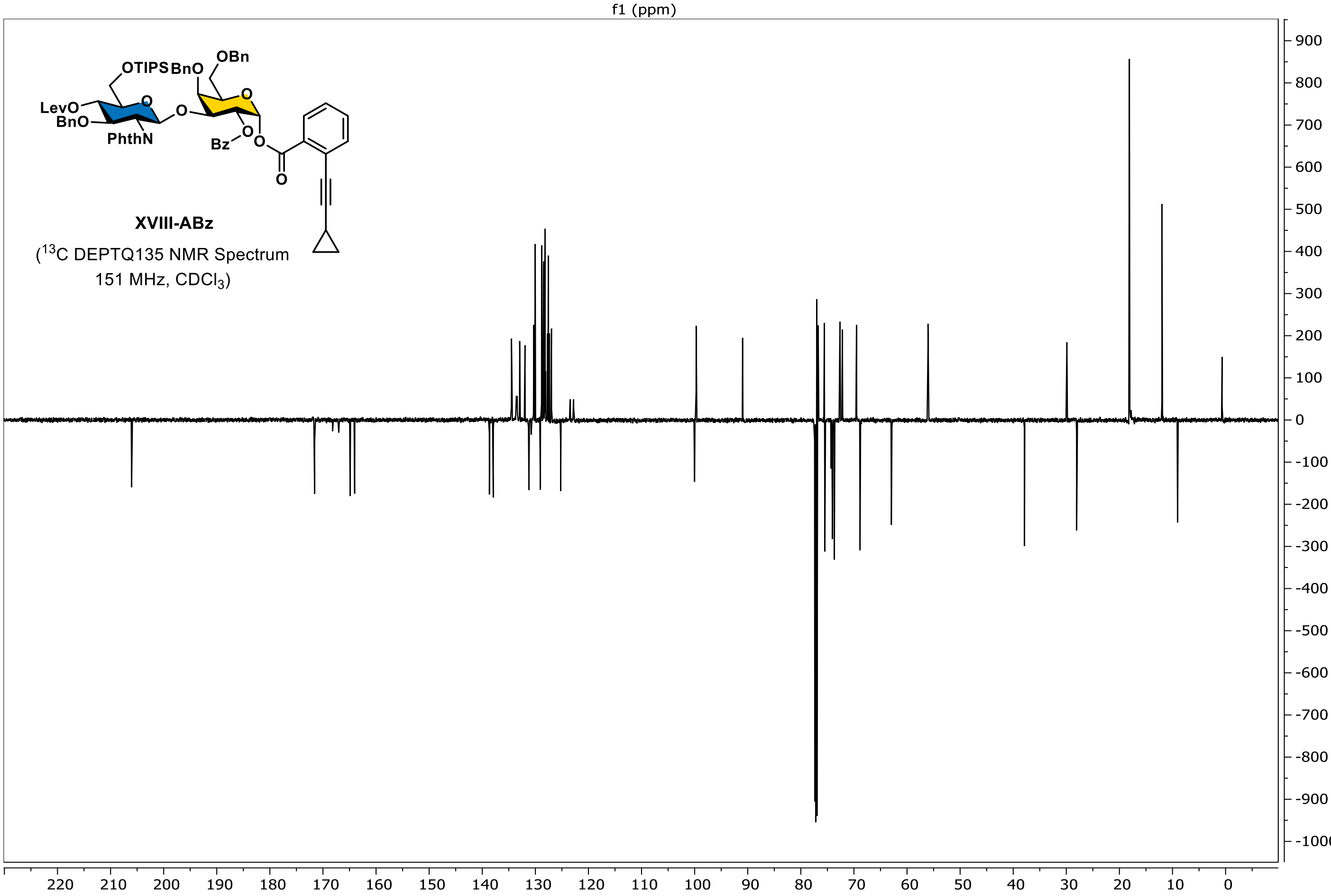
OTIPS
BnO
OBn
LevO
BnO
PhthN
Bz
XVIII-ABz
(13C DEPTQ135 NMR Spectrum
151 MHz, CDCl3)
f1 (ppm)

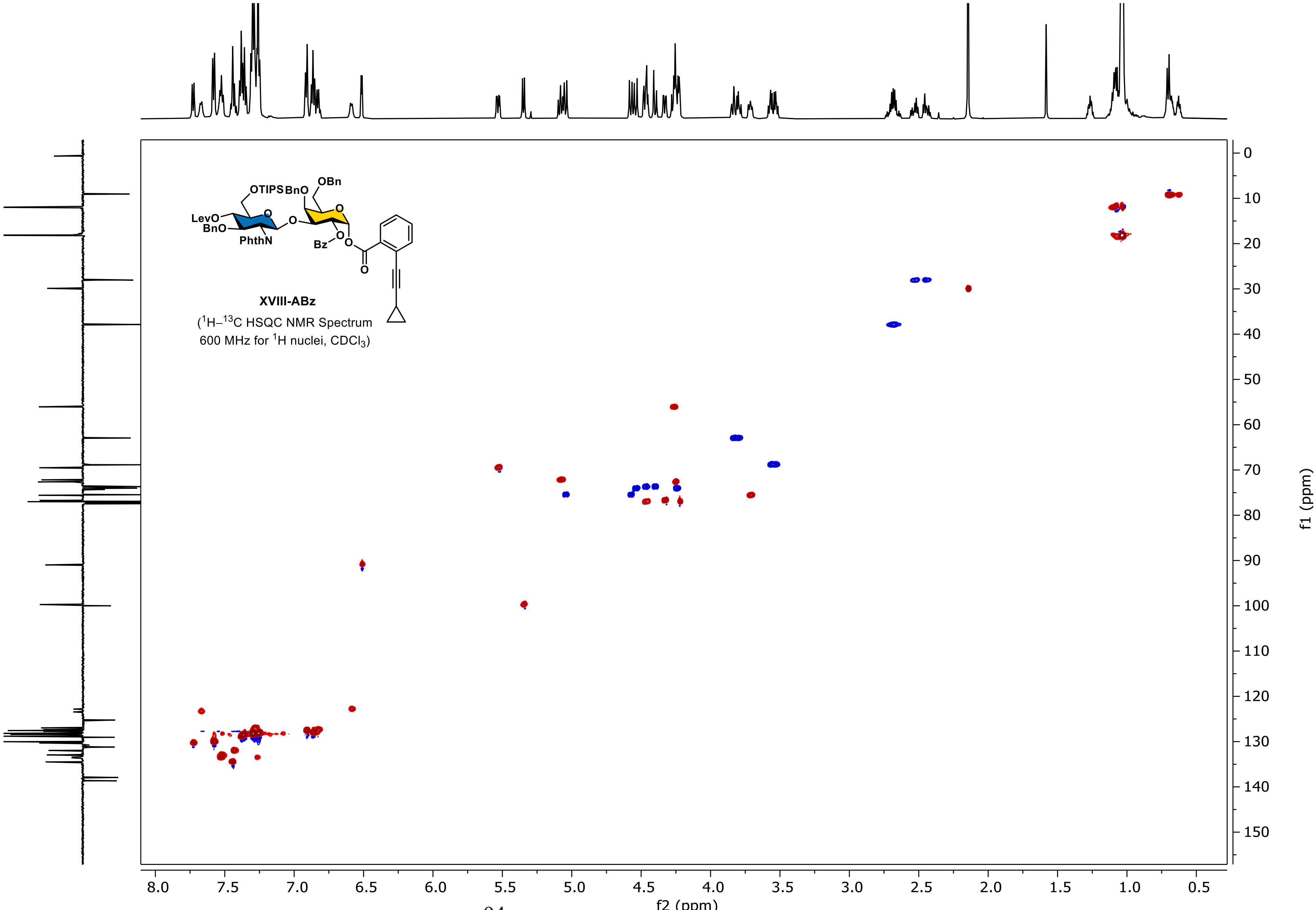
OTIPS
BnO
OBn
LevO
BnO
PhthN
Bz
XVIII-ABz
(¹H–¹³C HSQC NMR Spectrum
600 MHz for ¹H nuclei, CDCl3)
f2 (ppm)
f1 (ppm)

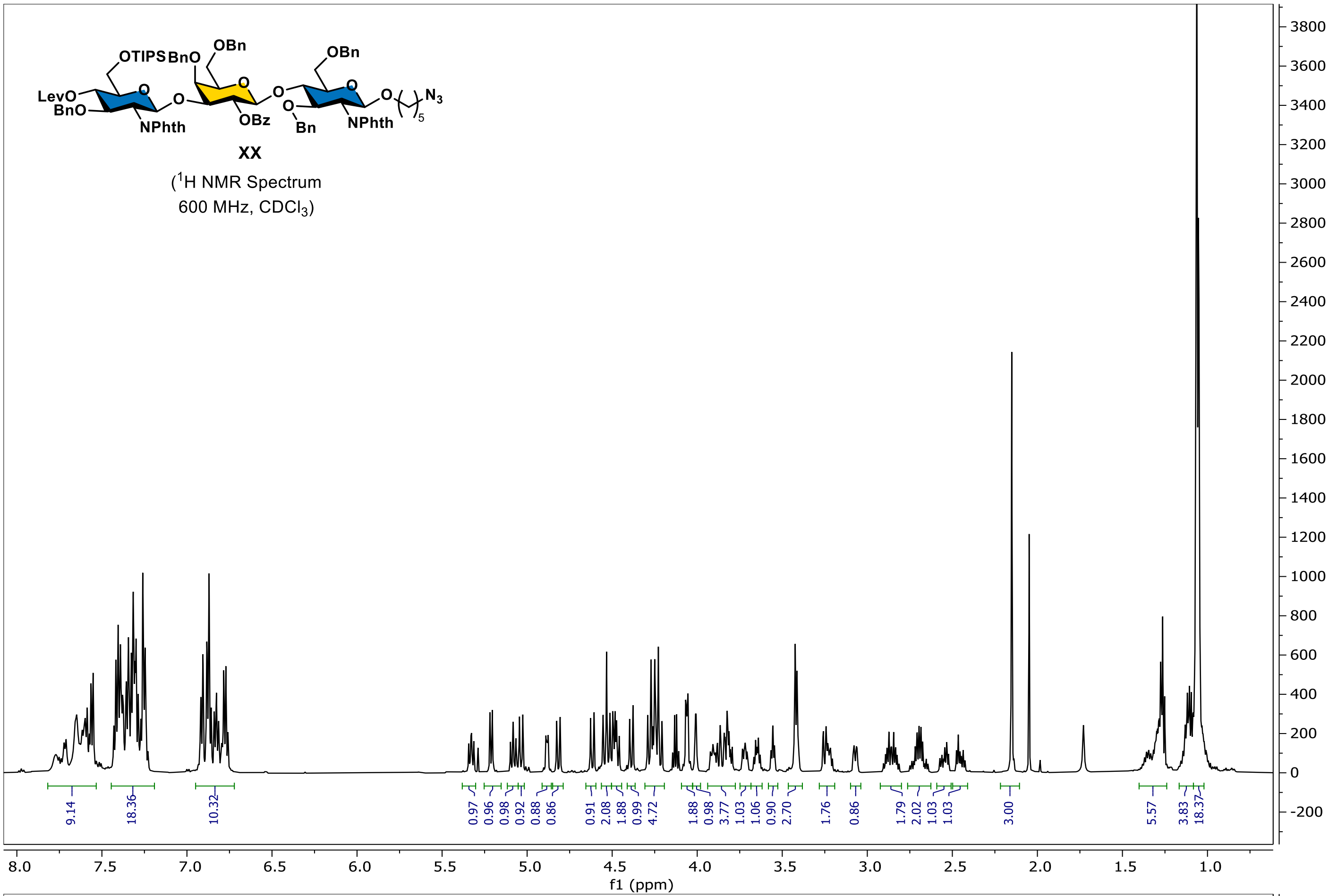
OTIPS
BnO
OBn
OBn
LevO
BnO
O
NPhth
OBz
Bn
NPhth
N3
5
XX
(1H NMR Spectrum
600 MHz, CDCl3)
f1 (ppm)

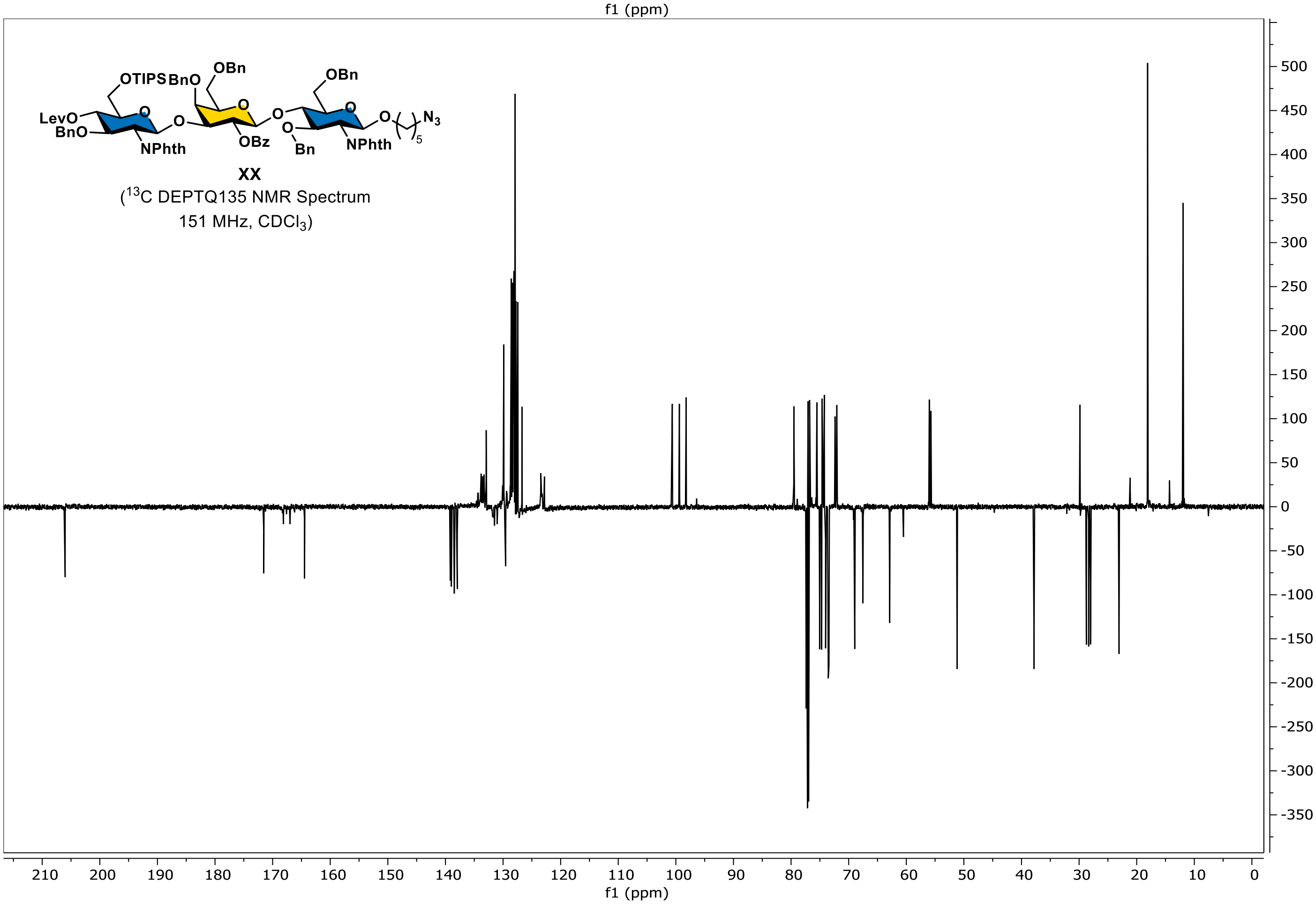
OTIPS
BnO
OBn
OBn
LevO
BnO
O
NPhth
OBz
Bn
NPhth
N3
5
XX
(13C DEPTQ135 NMR Spectrum
151 MHz, CDCl3)
f1 (ppm)

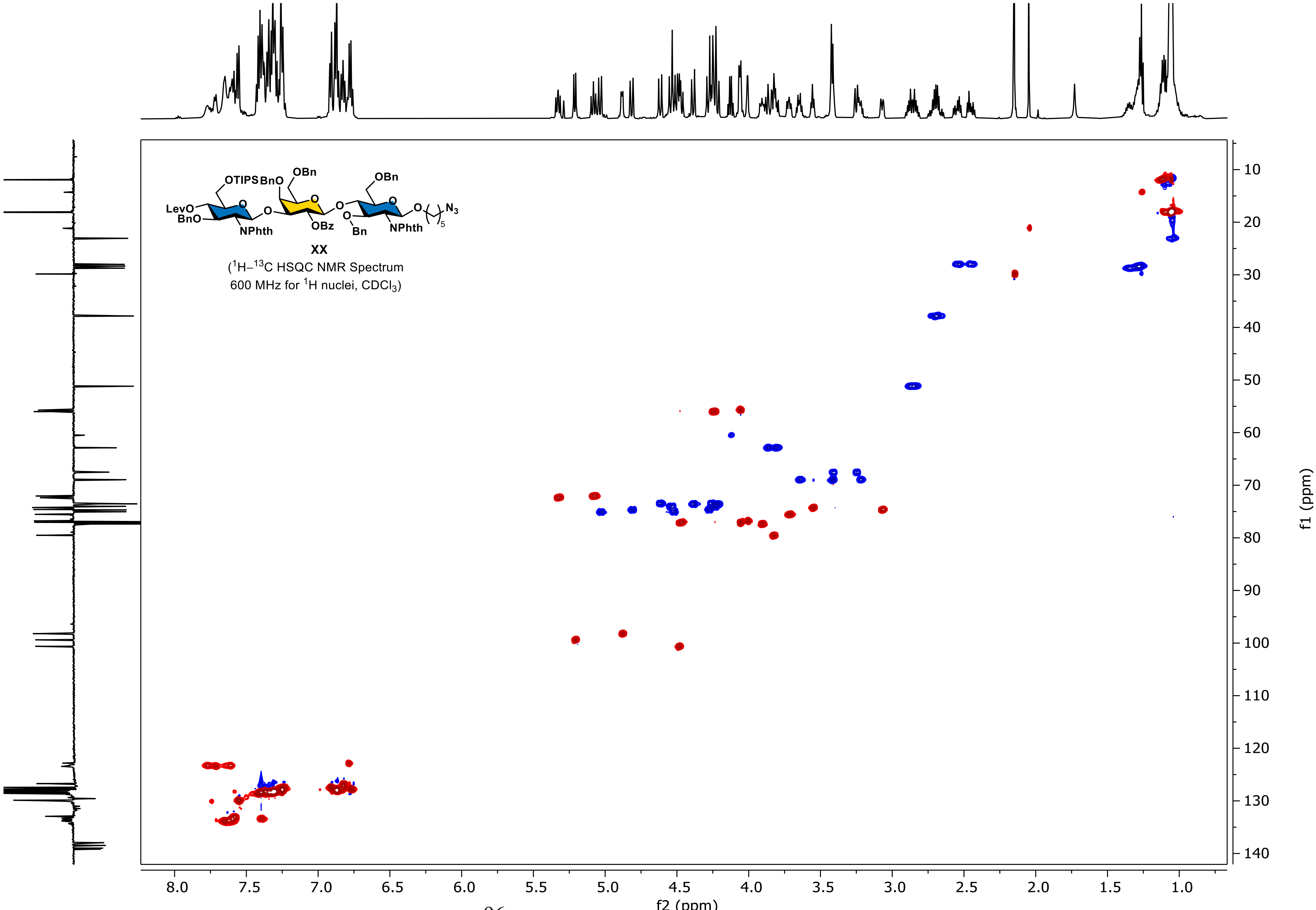
OTIPS
BnO
OBn
OBn
LevO
BnO
NPhth
OBz
Bn
NPhth
N3
XX
(1H–13C HSQC NMR Spectrum
600 MHz for 1H nuclei, CDCl3)
f2 (ppm)
f1 (ppm)

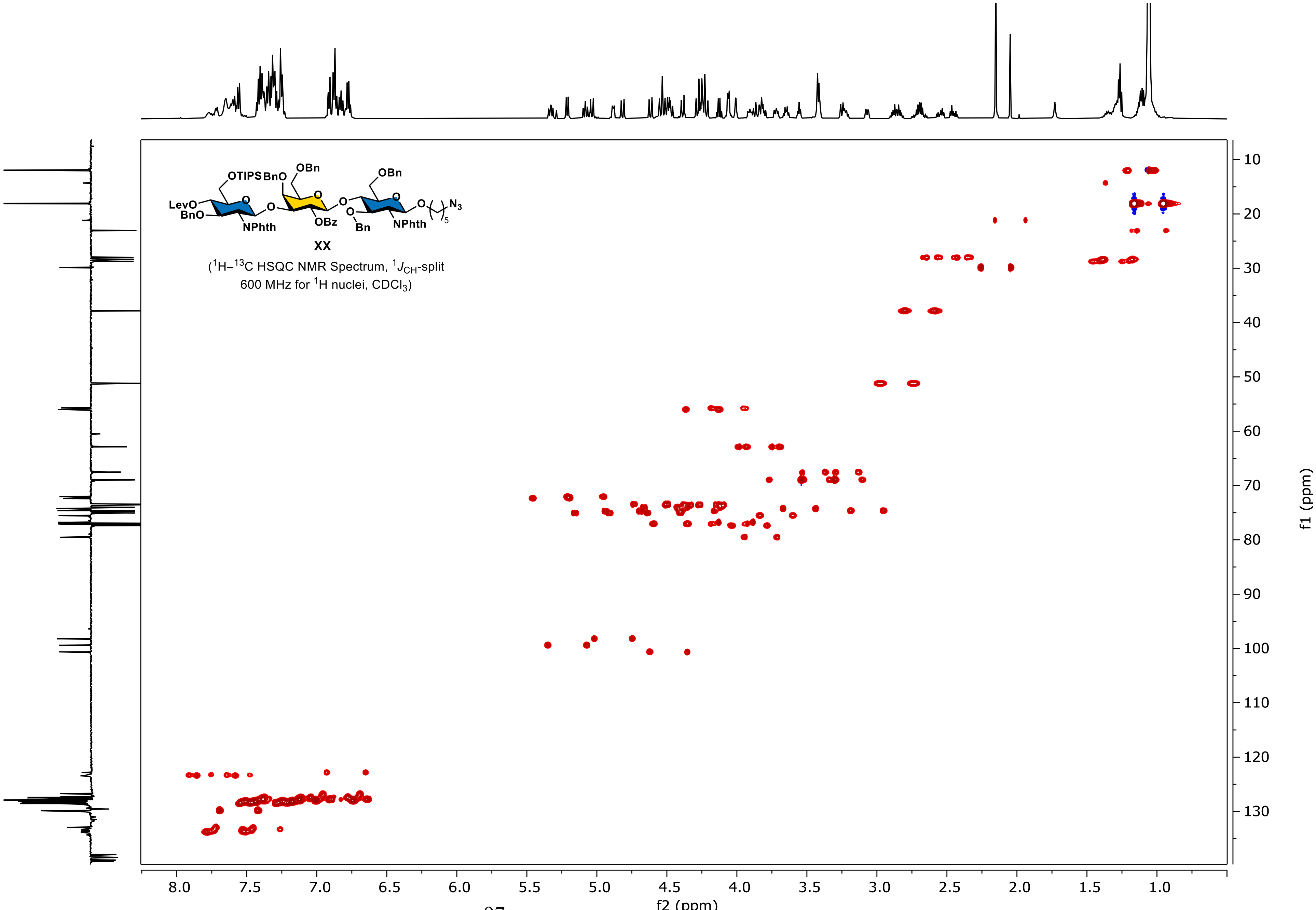

OTIPS
BnO
OBn
OBn
LevO
BnO
O
NPhth
OBz
Bn
NPhth
N3
5
XX
(1H–13C HSQC NMR Spectrum, 1JCH-split
600 MHz for 1H nuclei, CDCl3)
f2 (ppm)
f1 (ppm)

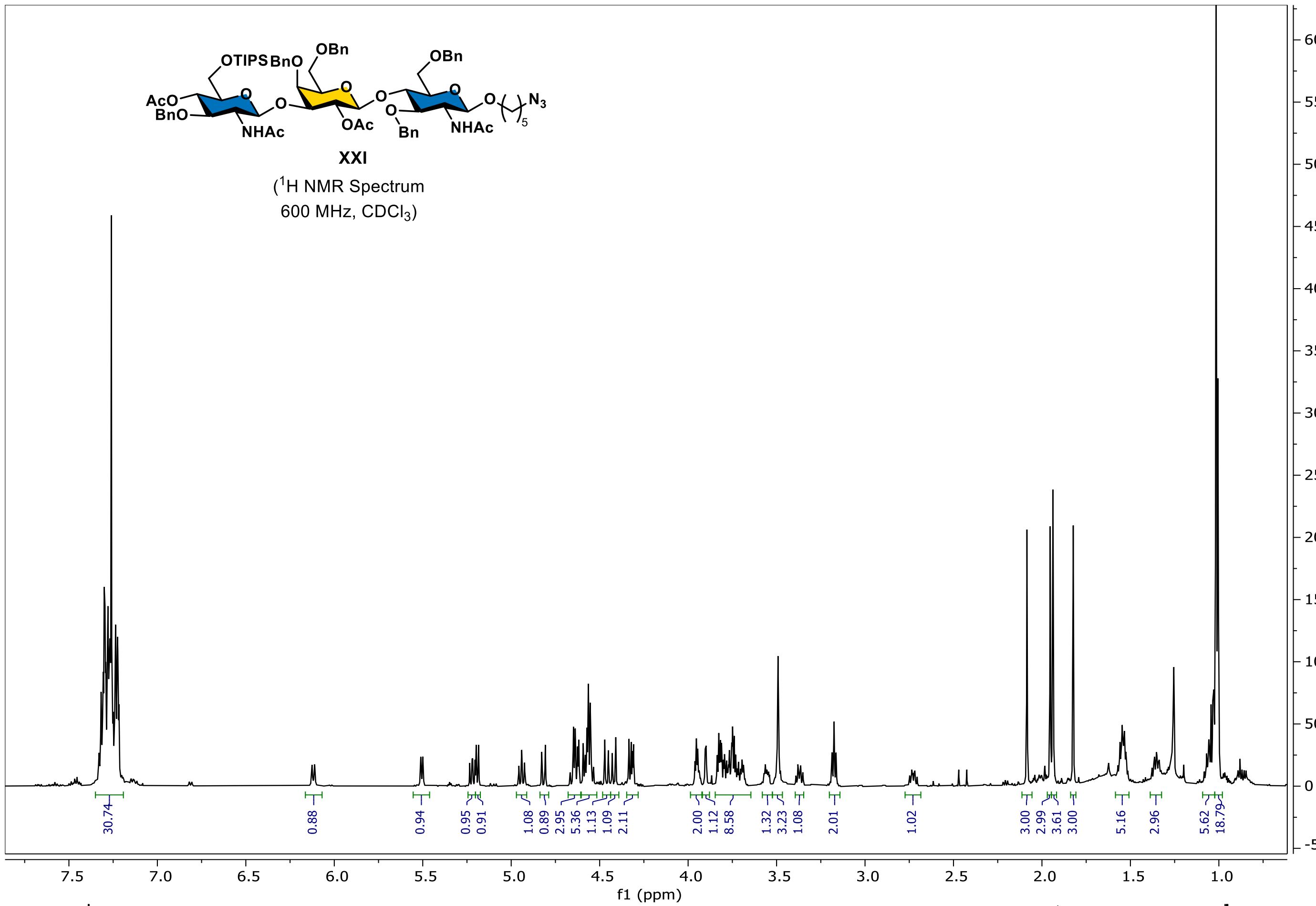

OTIPS
BnO
OBn
OBn
AcO
BnO
NHAc
OAc
Bn
NHAc
N3
5
XXI
(1H NMR Spectrum
600 MHz, CDCl3)
30.74
0.88
0.94
0.95
0.91
1.08
0.89
2.95
5.36
1.13
1.09
2.11
2.00
1.12
8.58
1.32
3.23
1.08
2.01
1.02
3.00
2.99
3.61
3.00
5.16
2.96
5.62
18.79
f1 (ppm)

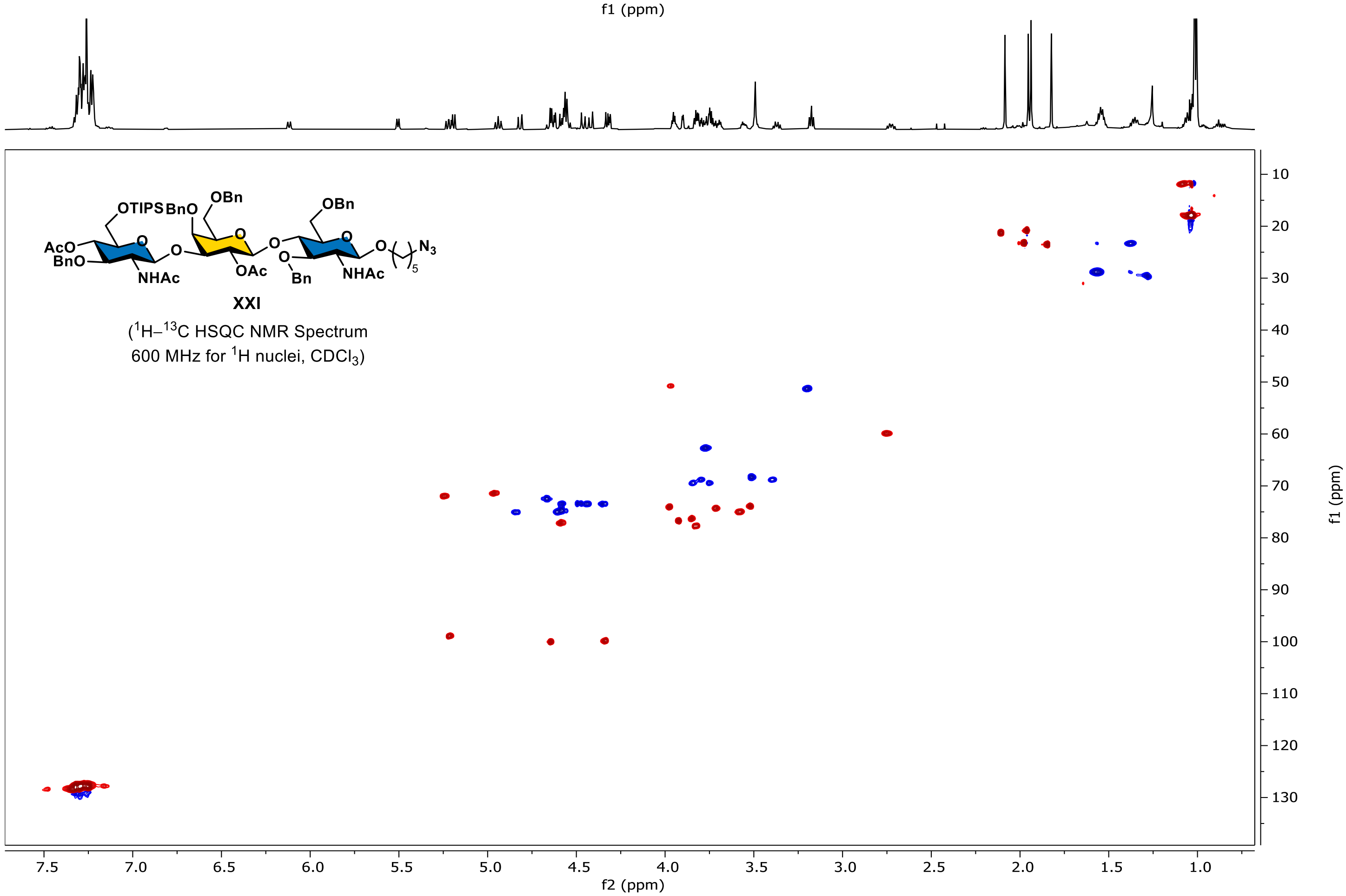

OTIPS
BnO
OBn
OBn
AcO
BnO
NHAc
OAc
Bn
NHAc
N3
5
XXI
(1H–13C HSQC NMR Spectrum
600 MHz for 1H nuclei, CDCl3)
f2 (ppm)
f1 (ppm)

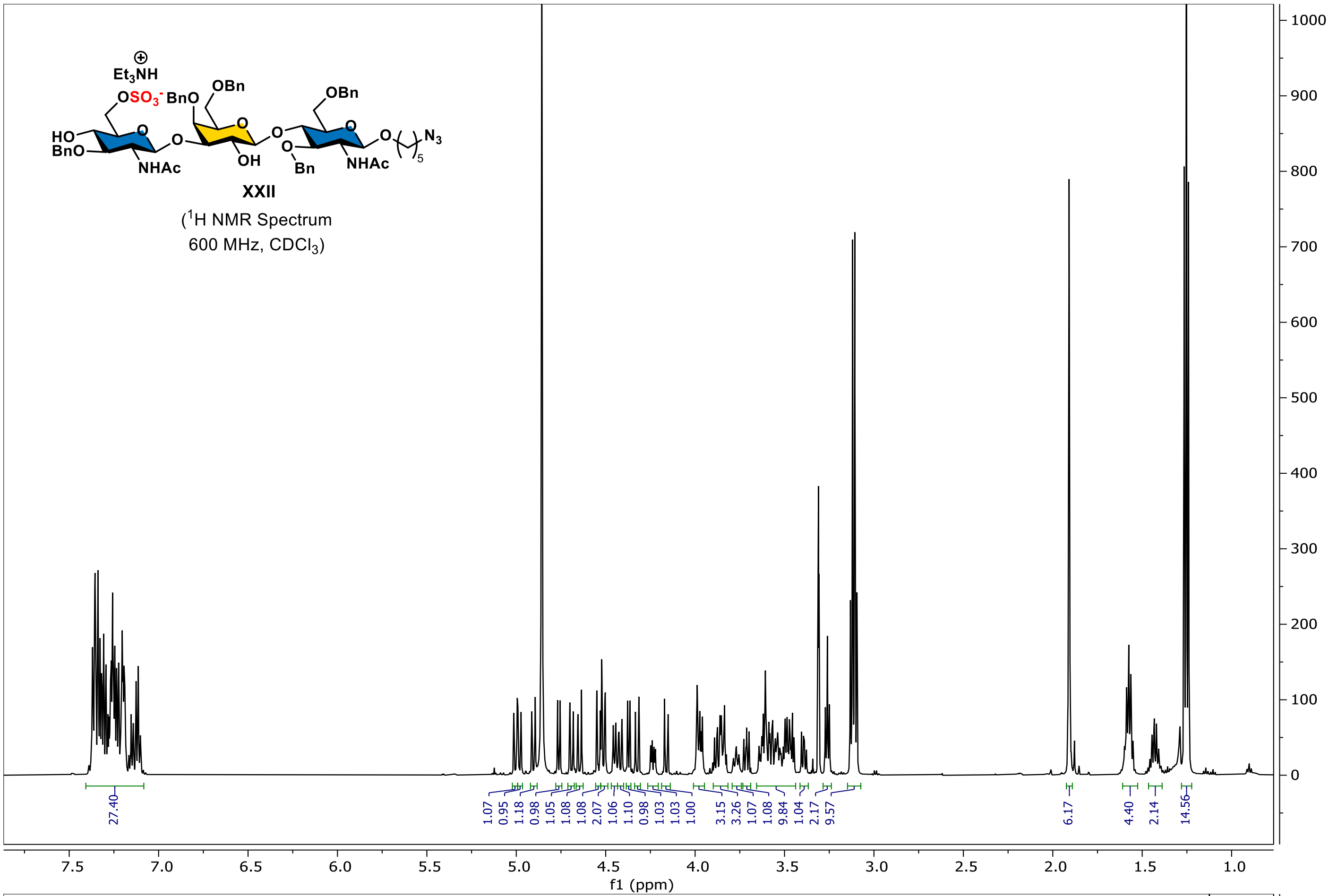

Et3NH
OSO3- BnO
OBn
OBn
HO
BnO
O
NHAc
OH
O
Bn
NHAc
N3
5
XXII
(1H NMR Spectrum
600 MHz, CDCl3)
f1 (ppm)

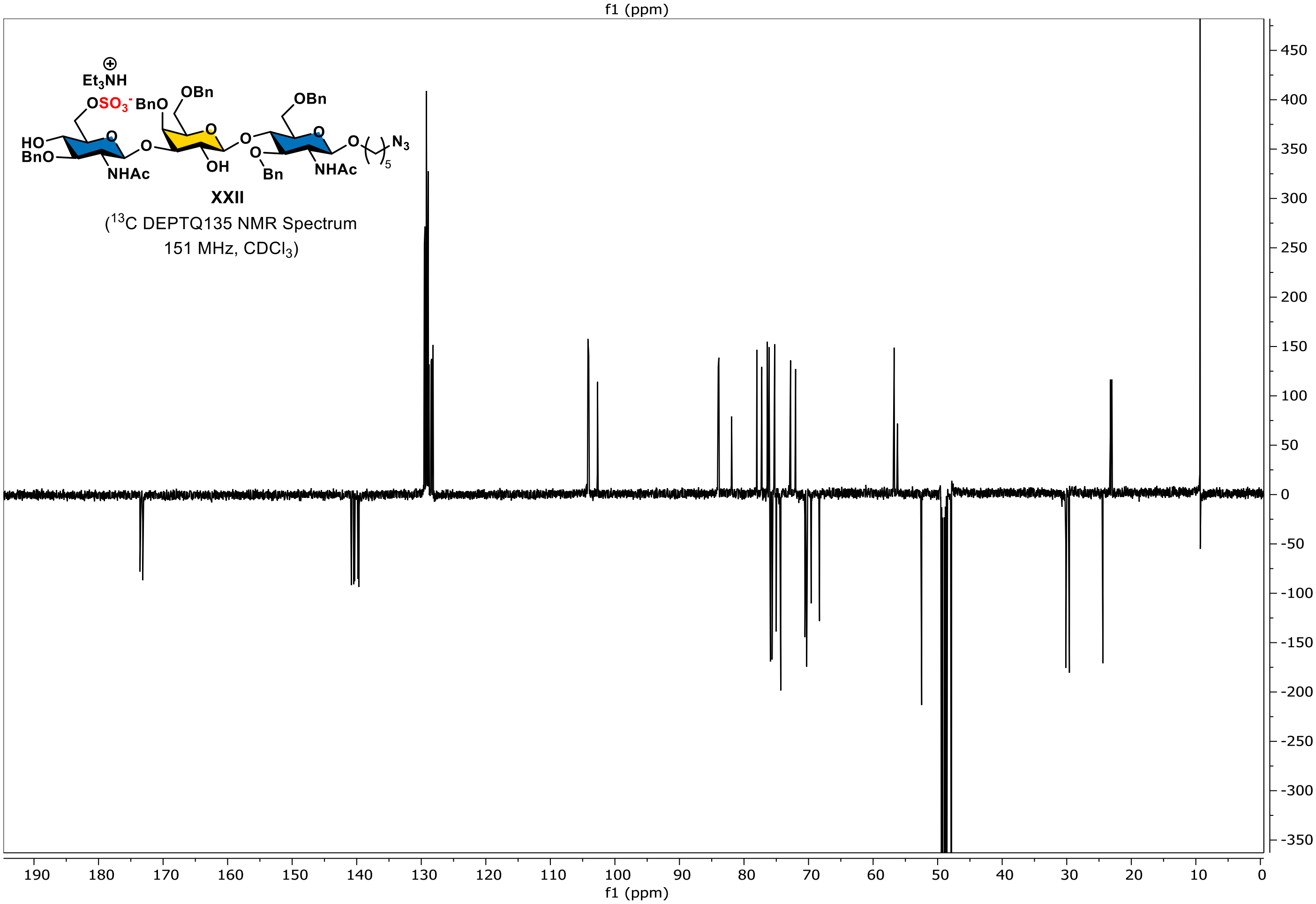

Et3NH
OSO3- BnO
OBn
OBn
HO
BnO
O
NHAc
OH
O
Bn
NHAc
N3
5
XXII
(13C DEPTQ135 NMR Spectrum
151 MHz, CDCl3)
f1 (ppm)

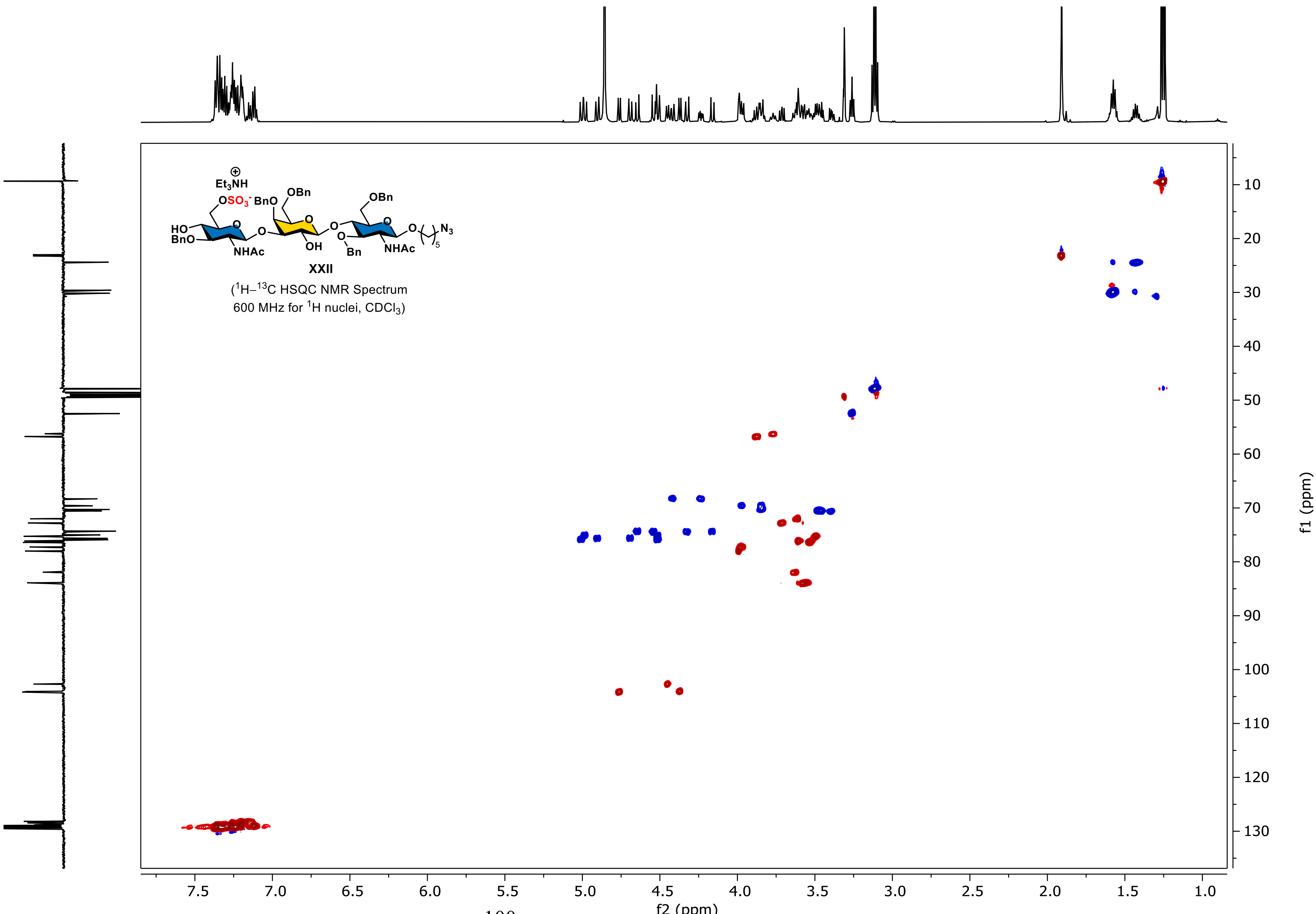
Et3NH
OSO3- BnO
OBn
OBn
HO
BnO
NHAc
OH
Bn
NHAc
N3
5
XXII
(1H–13C HSQC NMR Spectrum
600 MHz for 1H nuclei, CDCl3)
f2 (ppm)
f1 (ppm)

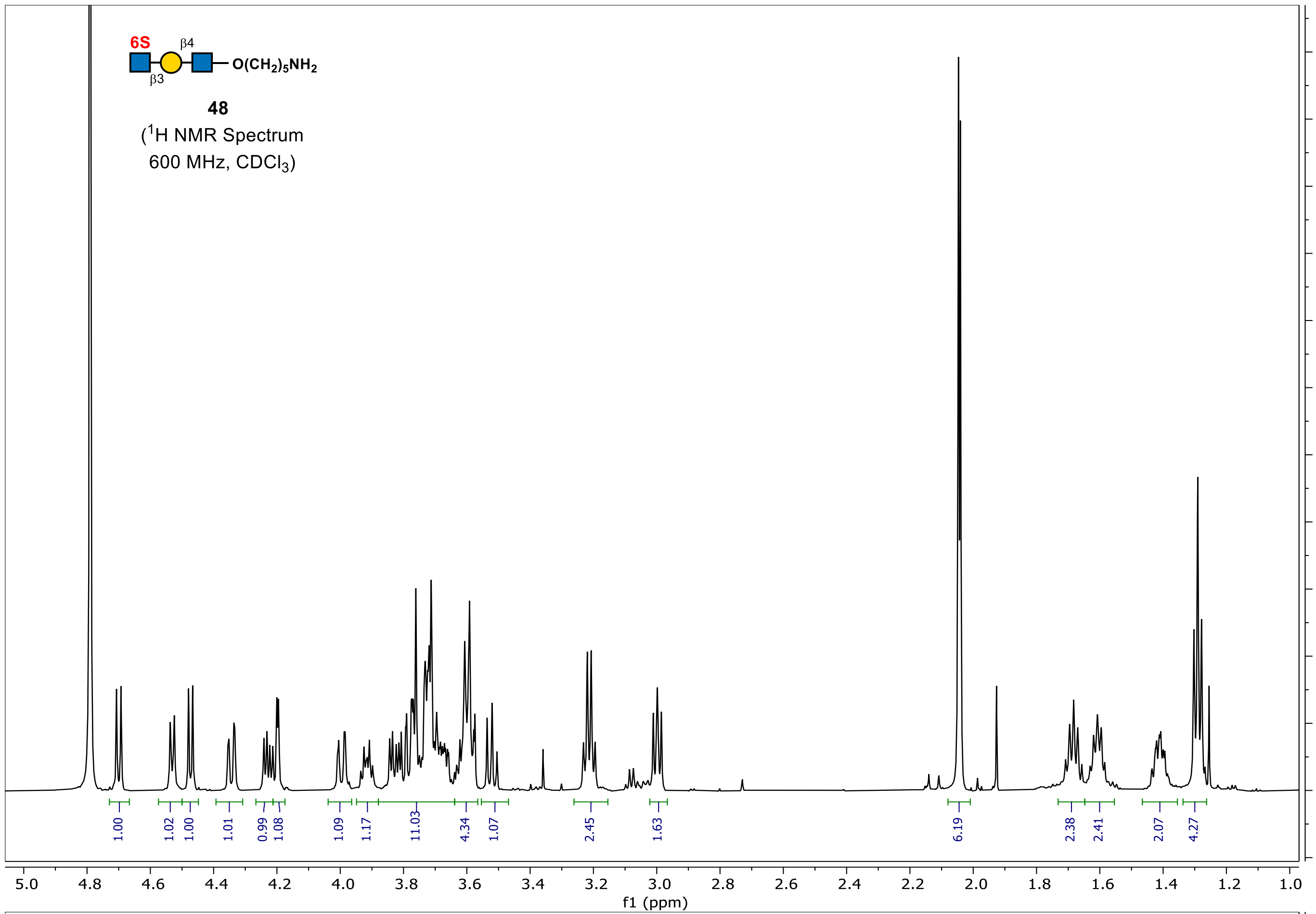
6S
β4
β3
O(CH2)5NH2
48
(1H NMR Spectrum
600 MHz, CDCl3)
f1 (ppm)

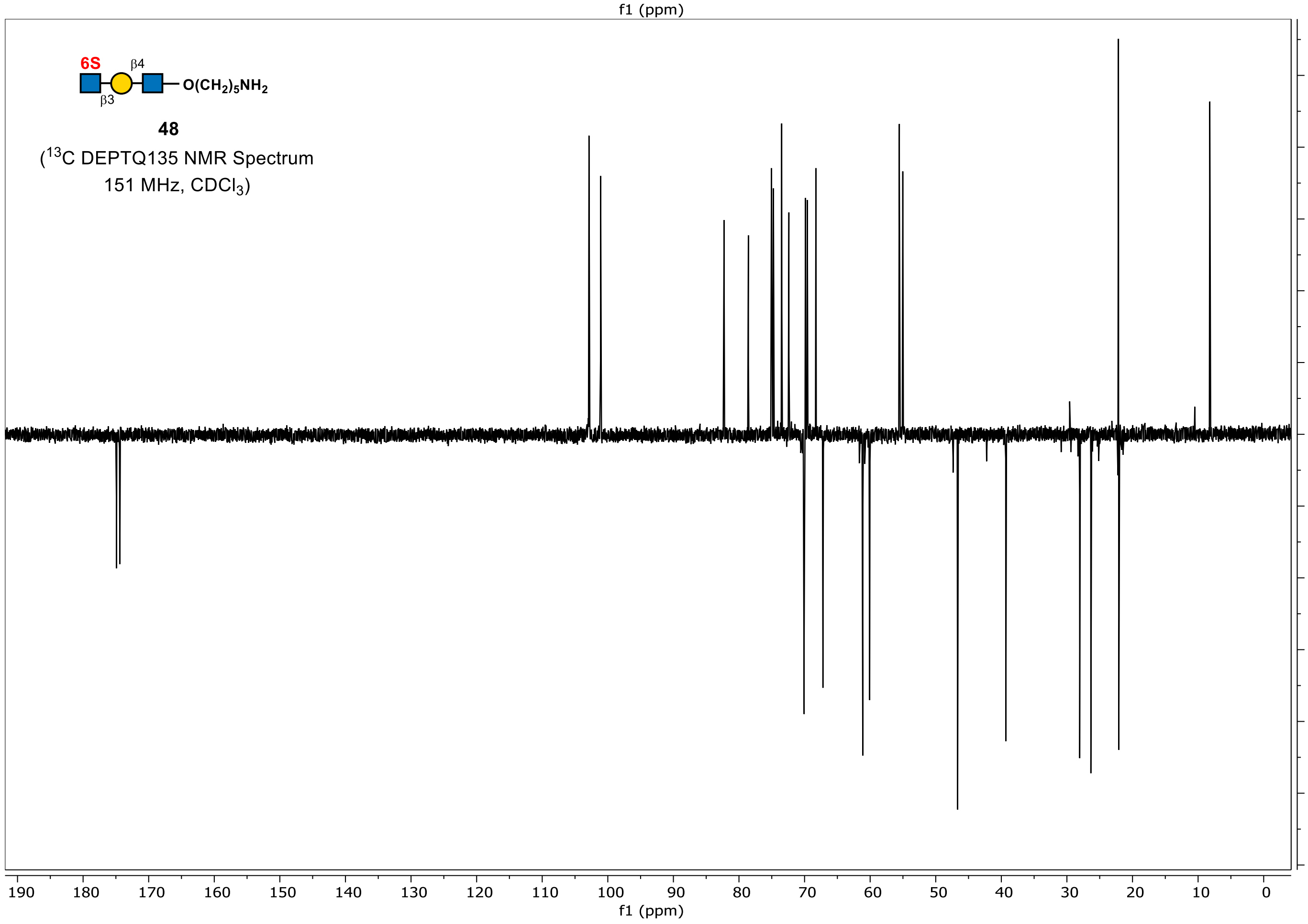
6S
β4
β3
O(CH2)5NH2
48
(13C DEPTQ135 NMR Spectrum
151 MHz, CDCl3)
f1 (ppm)

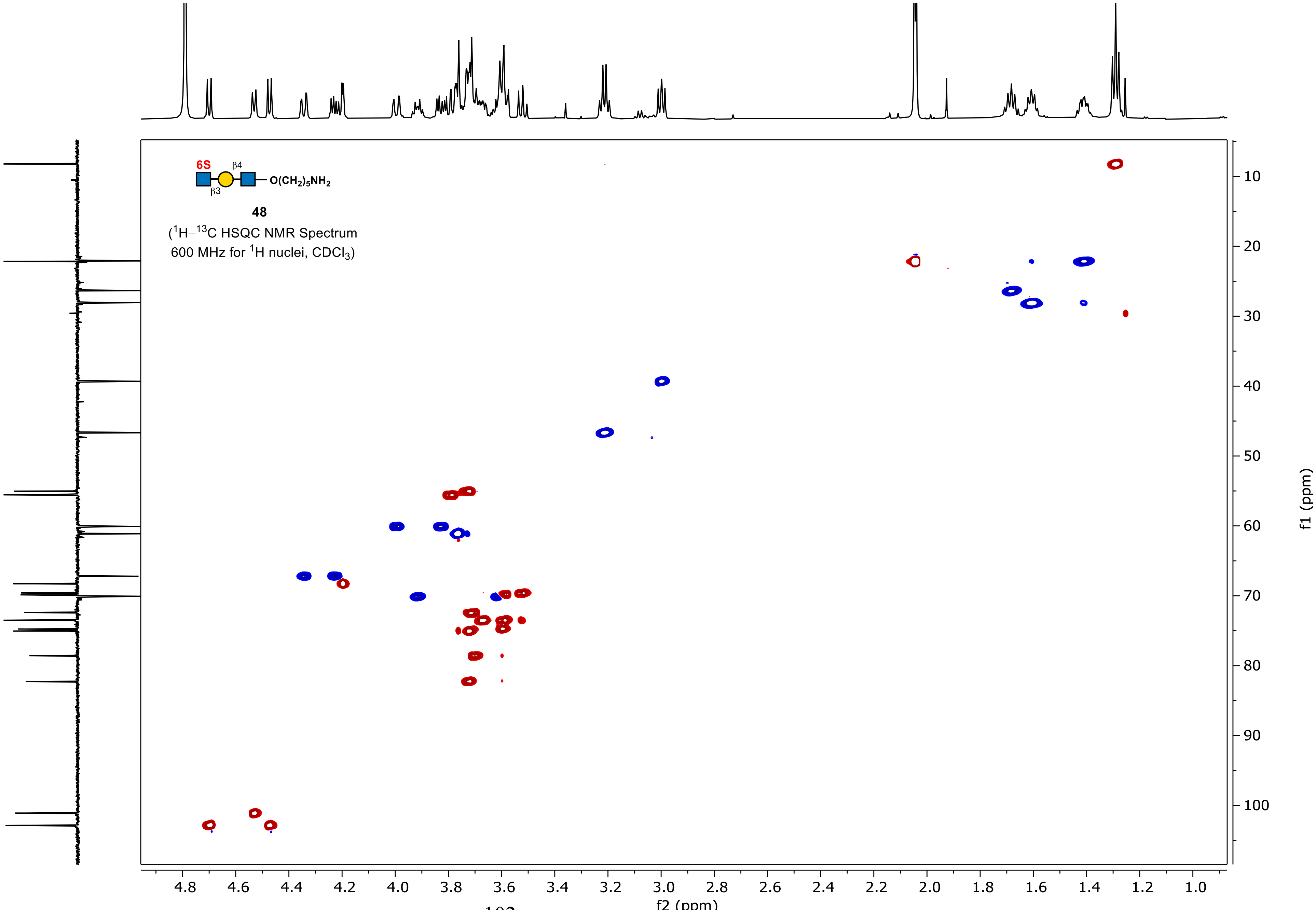
6S
β4
β3
O(CH2)5NH2
48
(1H–13C HSQC NMR Spectrum
600 MHz for 1H nuclei, CDCl3)
f2 (ppm)
f1 (ppm)

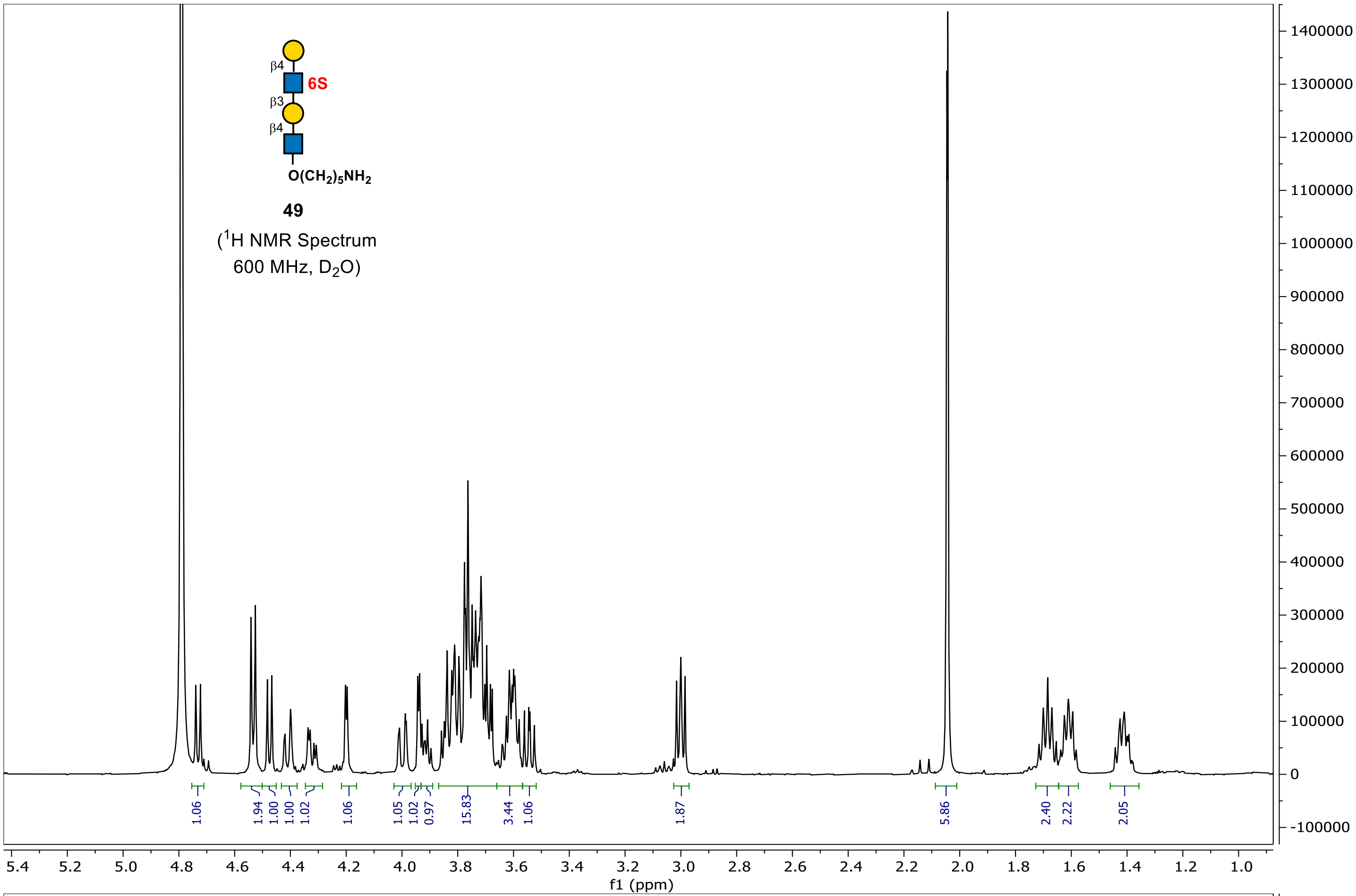
β4
6S
β3
β4
O(CH2)5NH2
49
(1H NMR Spectrum
600 MHz, D2O)
f1 (ppm)

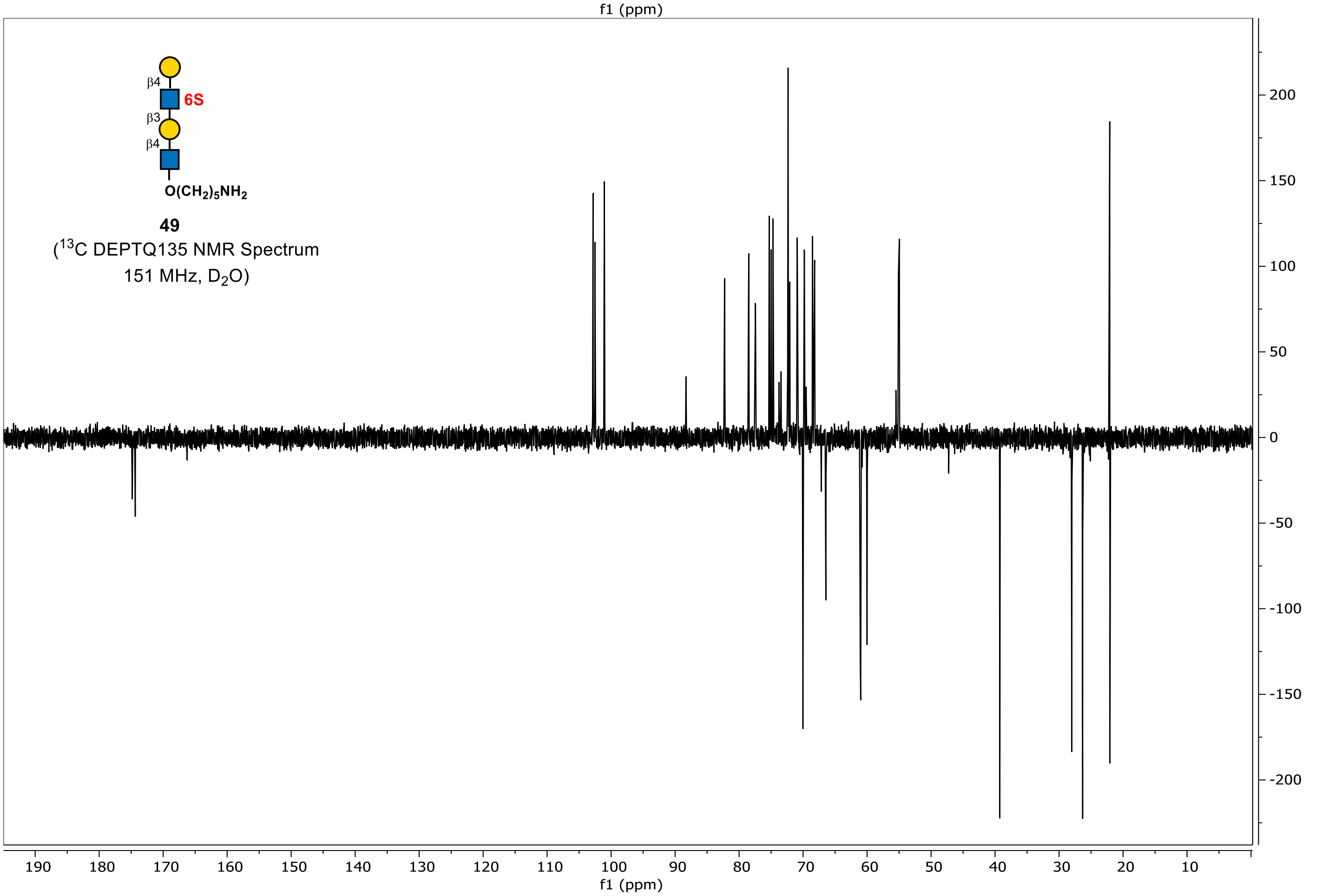
β4
6S
β3
β4
O(CH2)5NH2
49
(13C DEPTQ135 NMR Spectrum
151 MHz, D2O)
f1 (ppm)

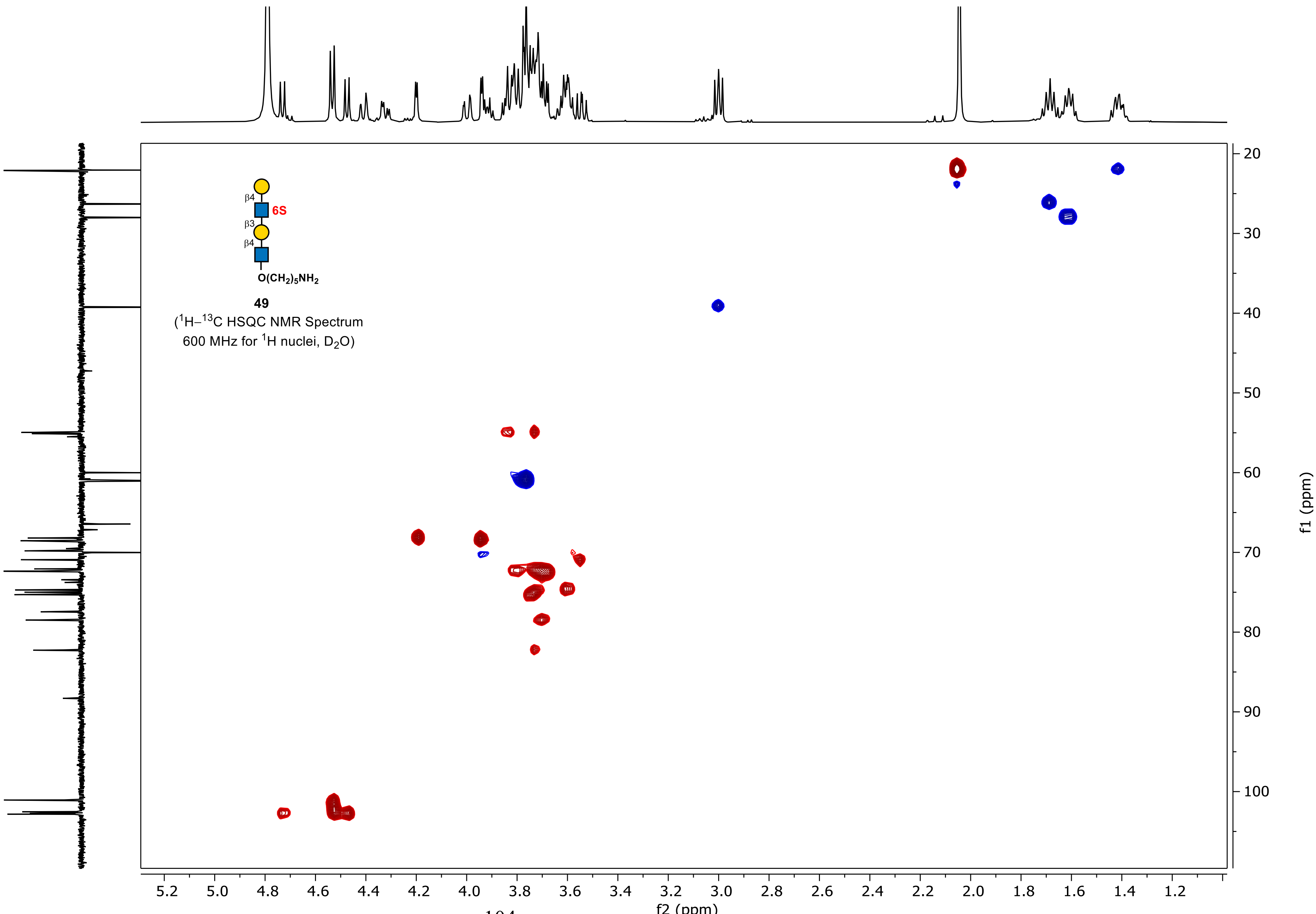
β4
6S
β3
β4
O(CH2)5NH2
49
(1H–13C HSQC NMR Spectrum
600 MHz for 1H nuclei, D2O)
f2 (ppm)
f1 (ppm)

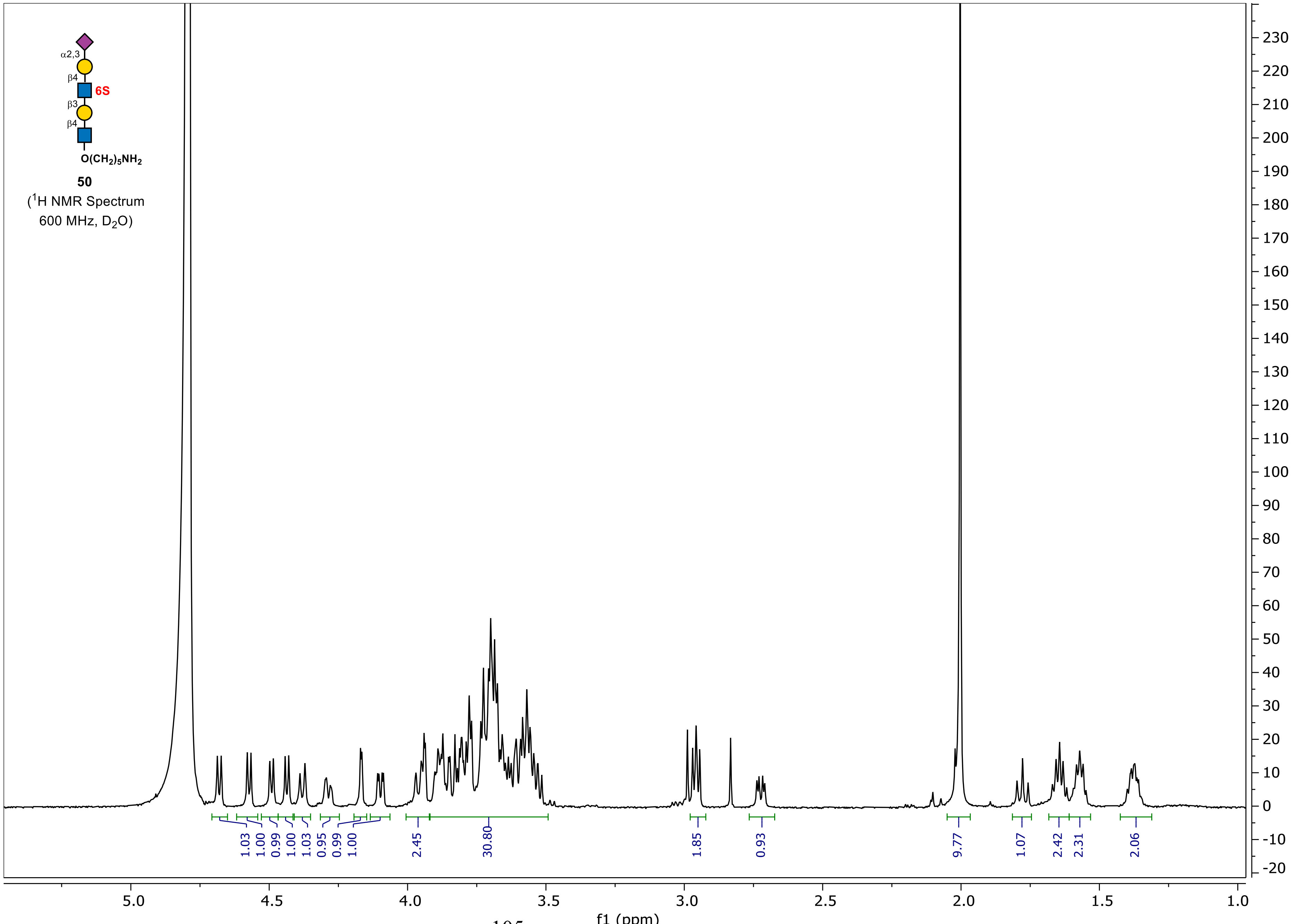
α2,3
β4
6S
β3
β4
O(CH2)5NH2
50
(1H NMR Spectrum
600 MHz, D2O)
1.03
1.00
0.99
1.00
1.03
0.95
0.99
1.00
2.45
30.80
1.85
0.93
9.77
1.07
2.42
2.31
2.06
f1 (ppm)

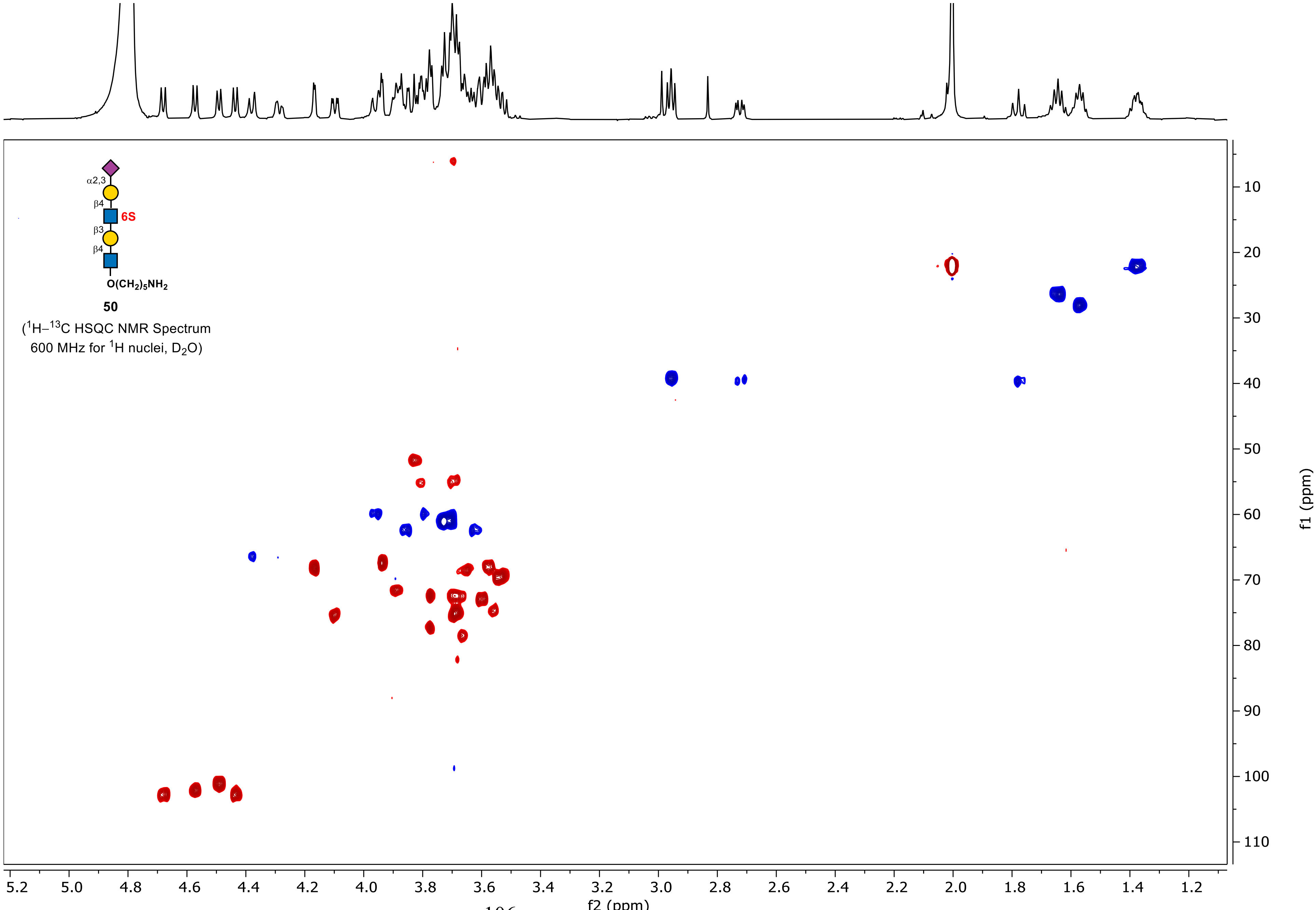

α2,3
β4
6S
β3
β4
O(CH2)5NH2
50
(1H–13C HSQC NMR Spectrum
600 MHz for 1H nuclei, D2O)
f2 (ppm)
f1 (ppm)

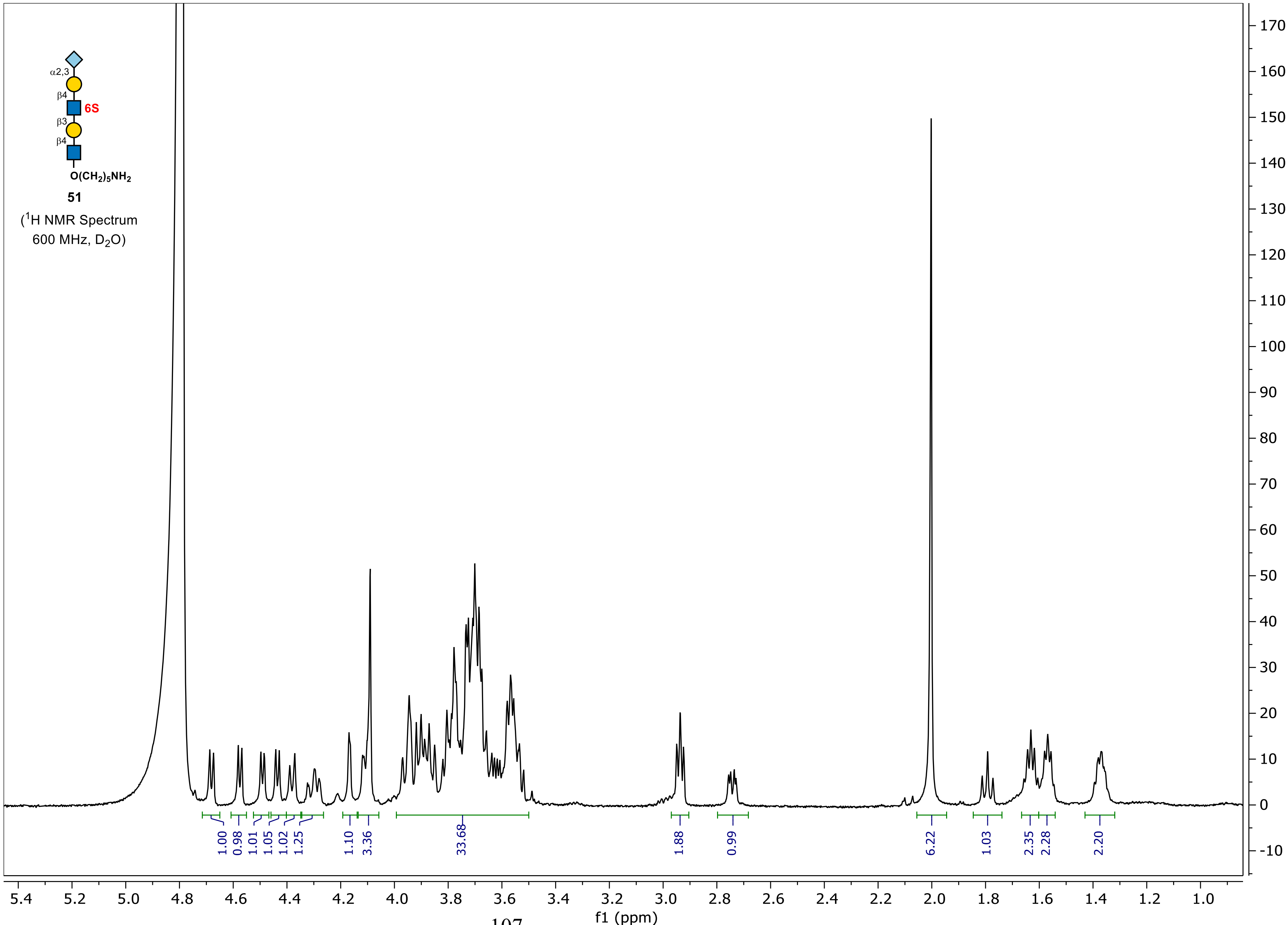
α2,3
β4
6S
β3
β4
O(CH2)5NH2
51
(1H NMR Spectrum
600 MHz, D2O)
1.00
0.98
1.01
1.05
1.02
1.25
1.10
3.36
33.68
1.88
0.99
6.22
1.03
2.35
2.28
2.20
f1 (ppm)

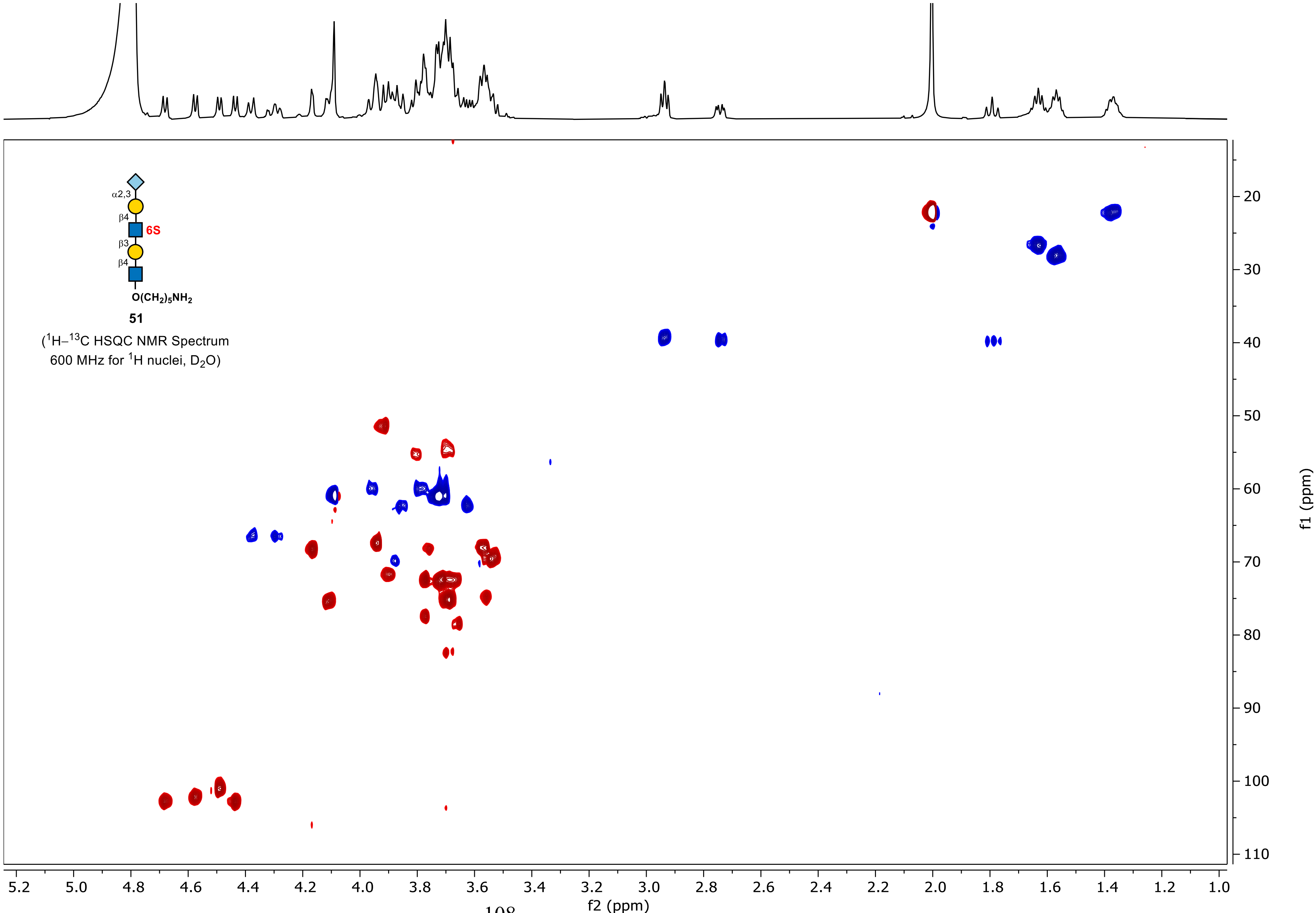
α2,3
β4
6S
β3
β4
$O(CH_2)_5NH_2$
51
($^1H-^{13}C$ HSQC NMR Spectrum
600 MHz for ^1H nuclei, D_2O)
f2 (ppm)
f1 (ppm)

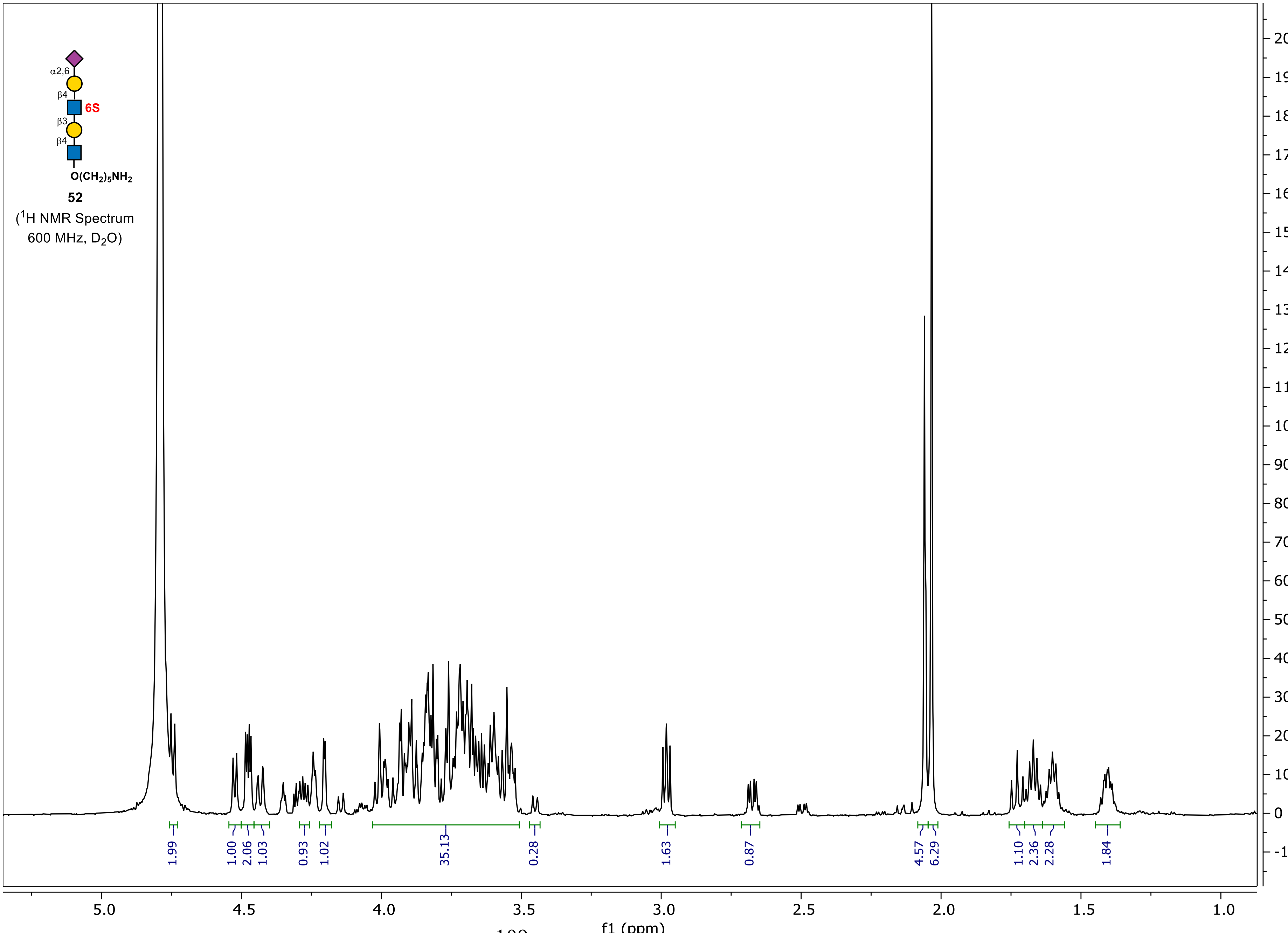
α2,6
β4
6S
β3
β4
O(CH2)5NH2
52
(1H NMR Spectrum
600 MHz, D2O)
f1 (ppm)

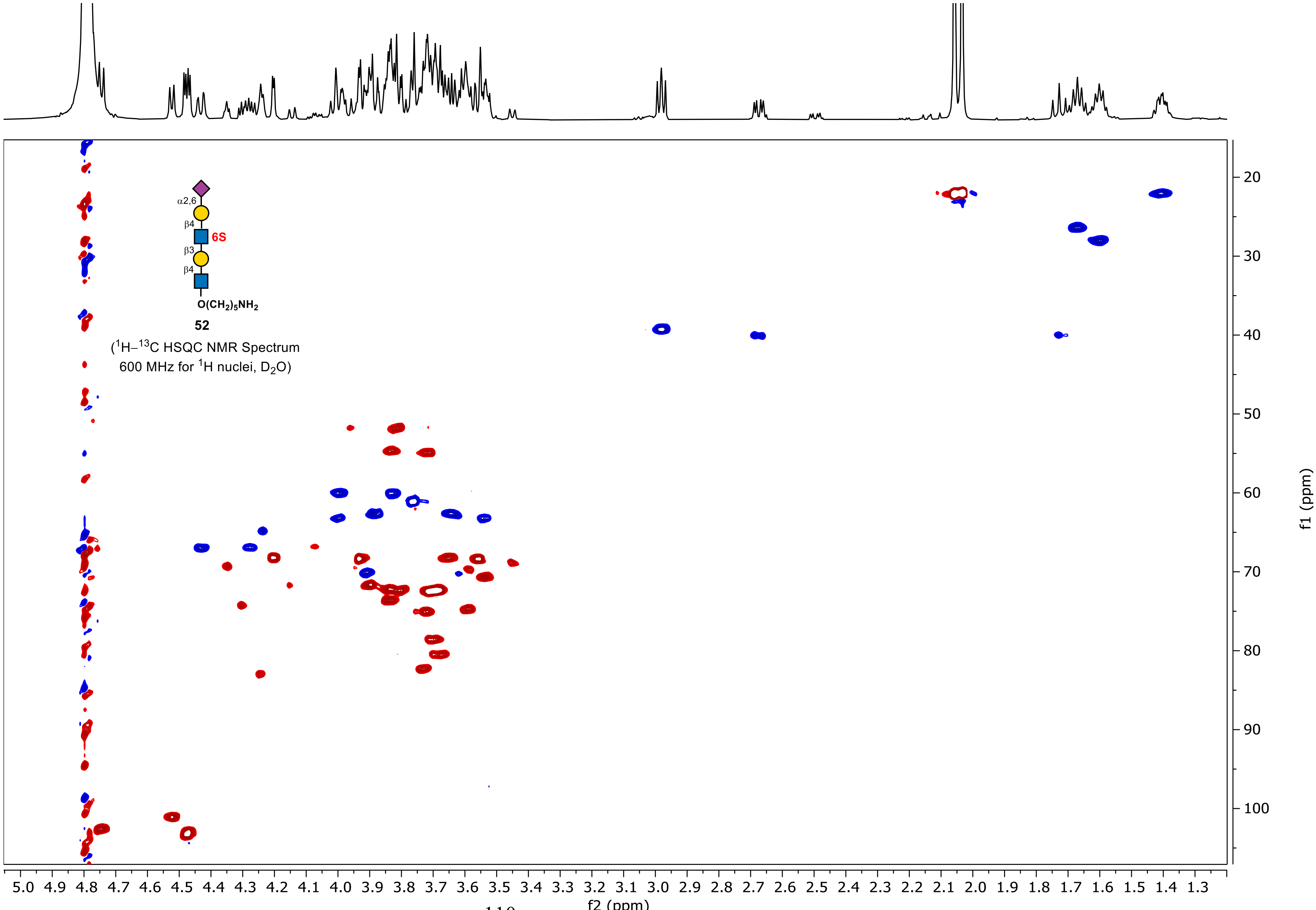
α2,6
β4
6S
β3
β4
O(CH2)5NH2
52
(1H–13C HSQC NMR Spectrum
600 MHz for 1H nuclei, D2O)
f1 (ppm)
f2 (ppm)

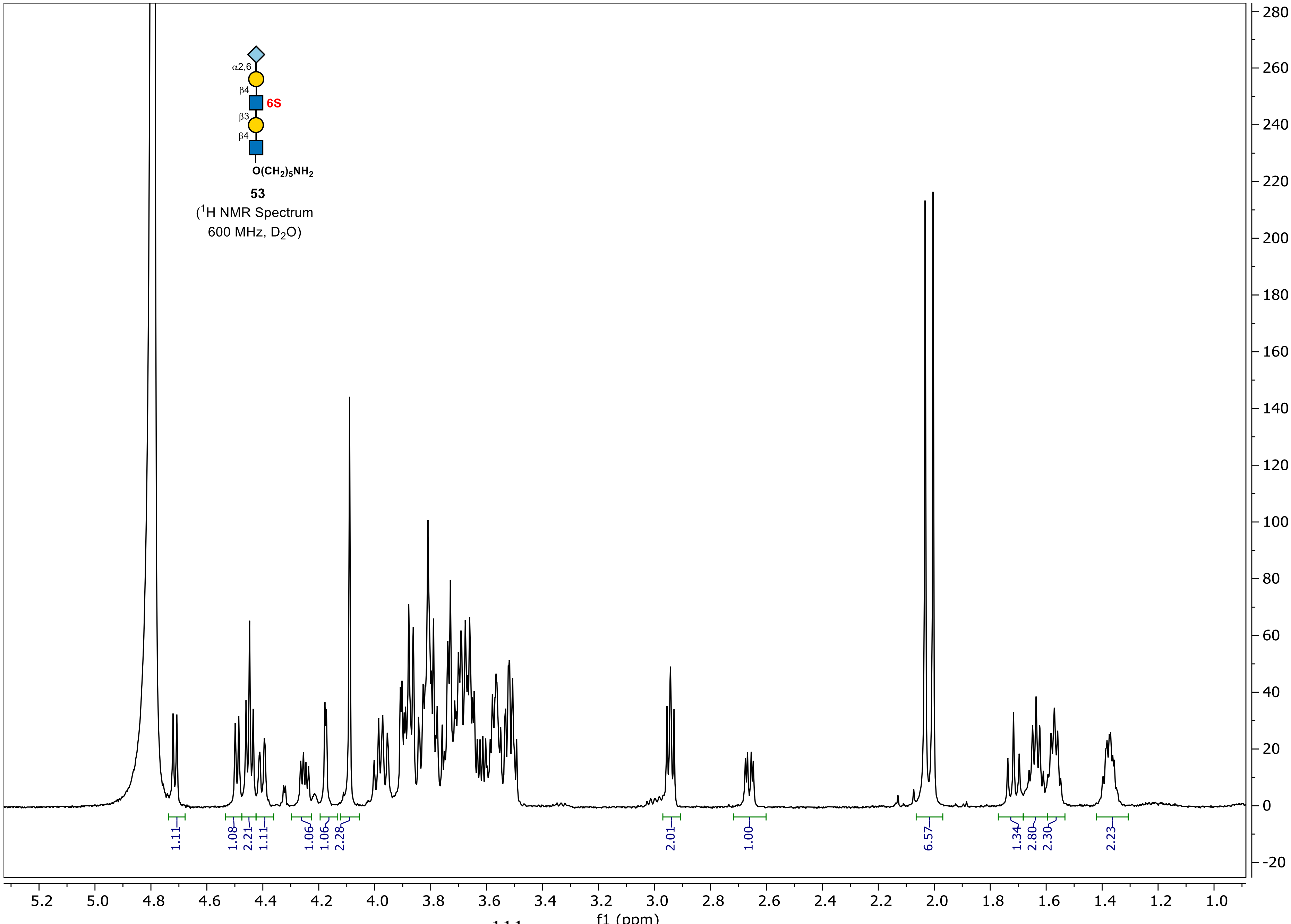
α2,6
β4
6S
β3
β4
O(CH2)5NH2
53
(1H NMR Spectrum
600 MHz, D2O)
1.11
1.08
2.21
1.11
1.06
1.06
2.28
2.01
1.00
6.57
1.34
2.80
2.30
2.23
f1 (ppm)

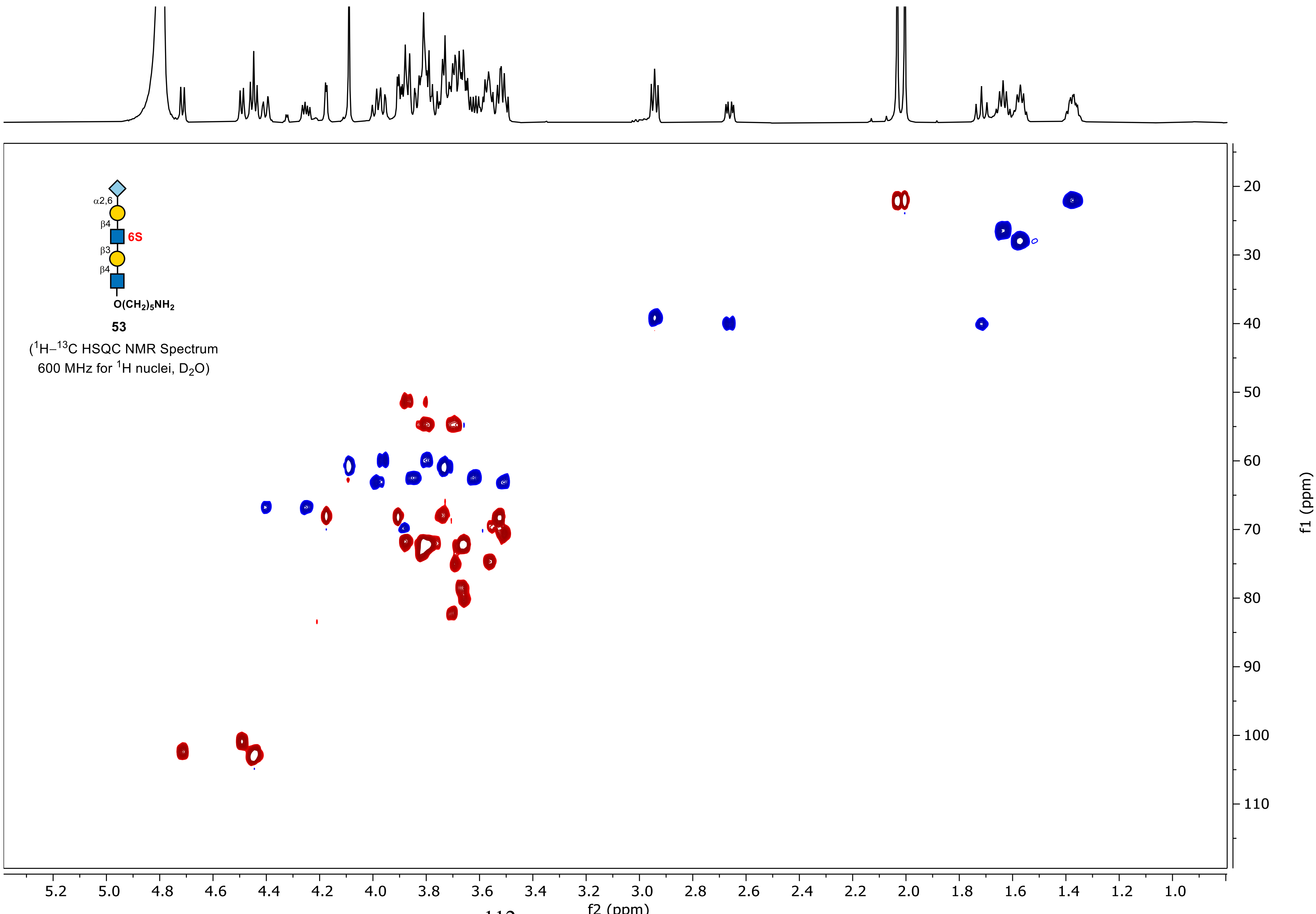
α2,6
β4
6S
β3
β4
O(CH2)5NH2
53
(1H–13C HSQC NMR Spectrum
600 MHz for 1H nuclei, D2O)
f2 (ppm)
f1 (ppm)

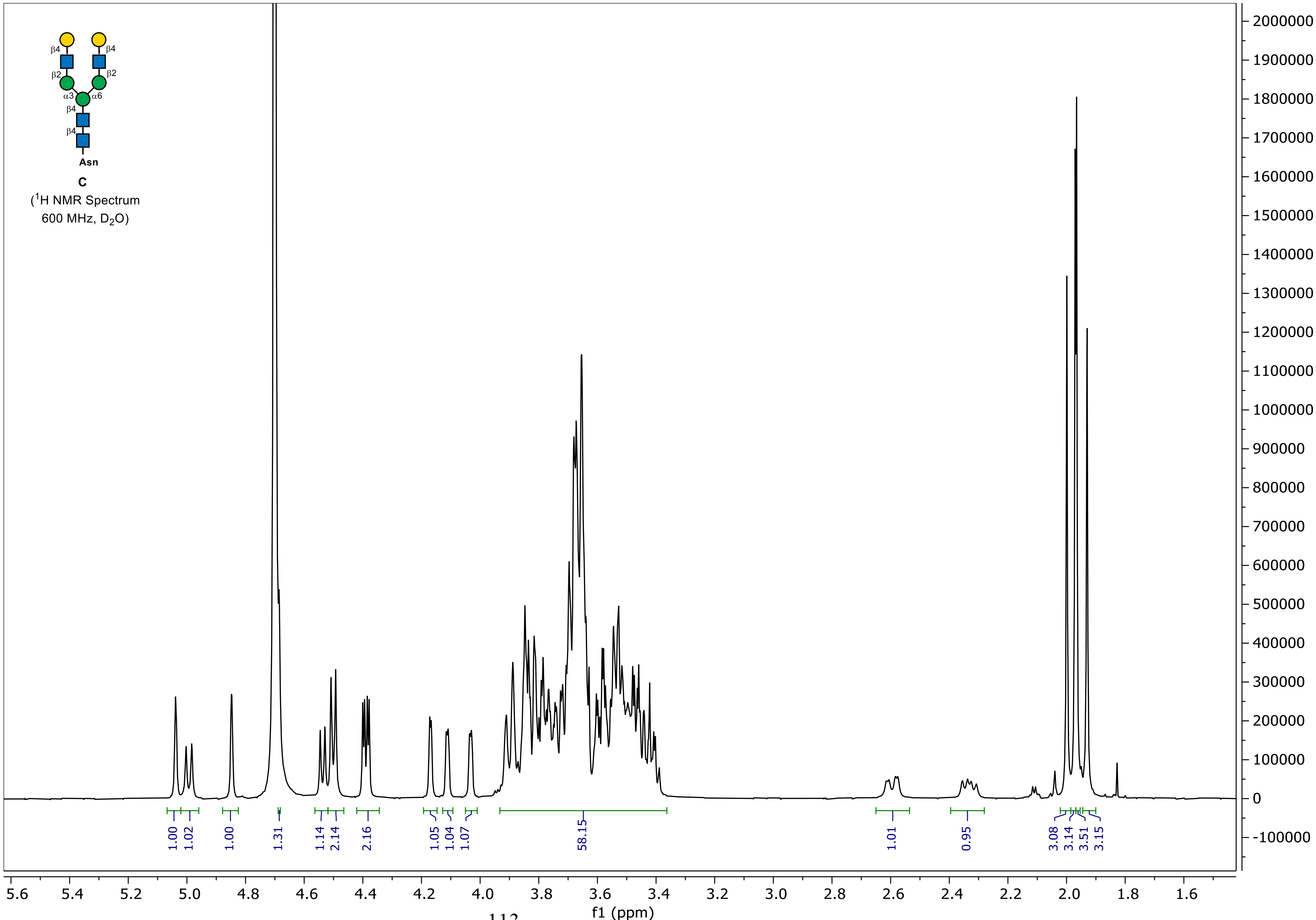

Asn
C
(^{1}H NMR Spectrum
600 MHz, D_2O)
f1 (ppm)

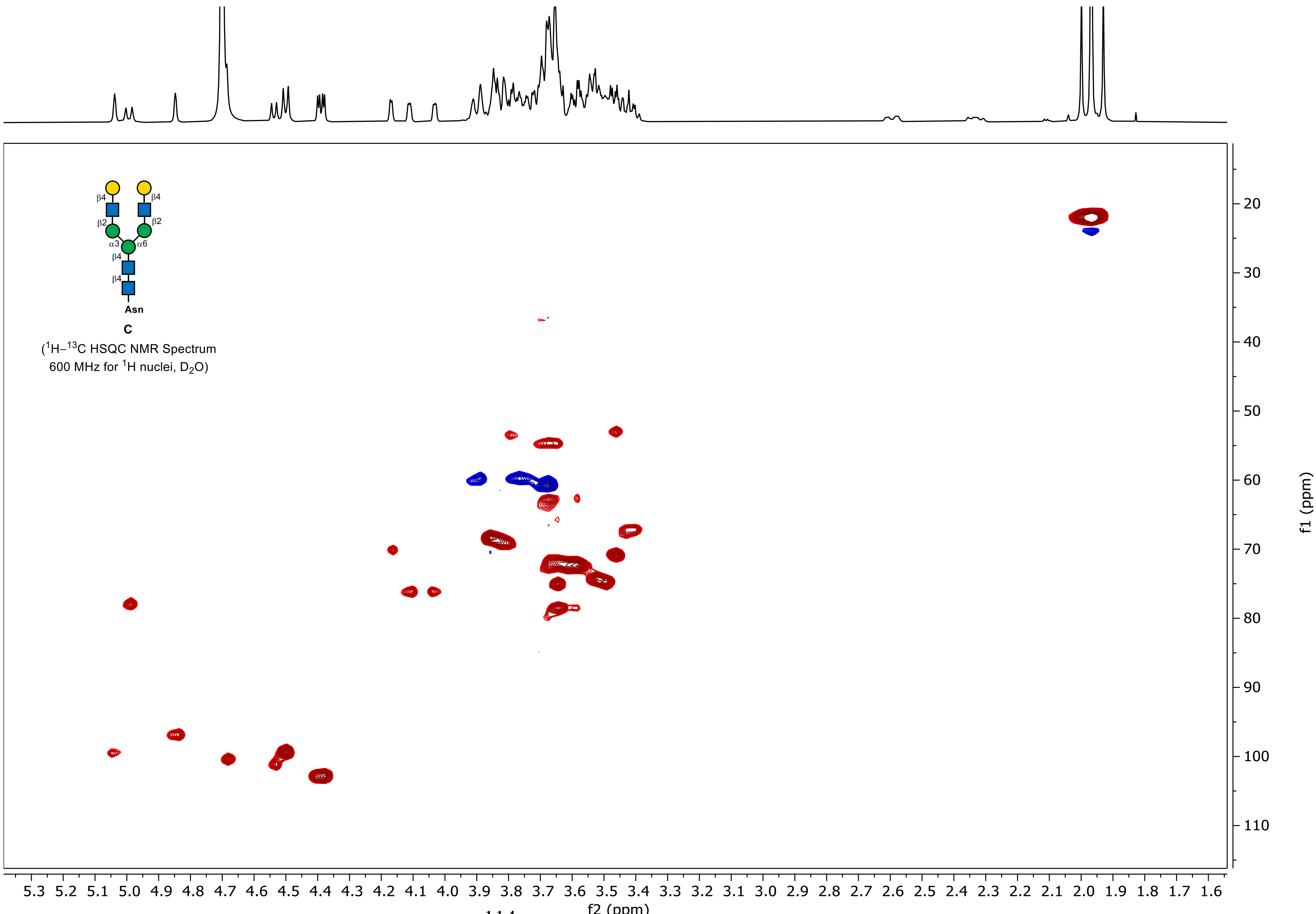
β4
β4
β2
β2
α3
α6
β4
β4
Asn
C
(^{1}H–^{13}C HSQC NMR Spectrum
600 MHz for ^{1}H nuclei, D_2O)
f1 (ppm)
f2 (ppm)

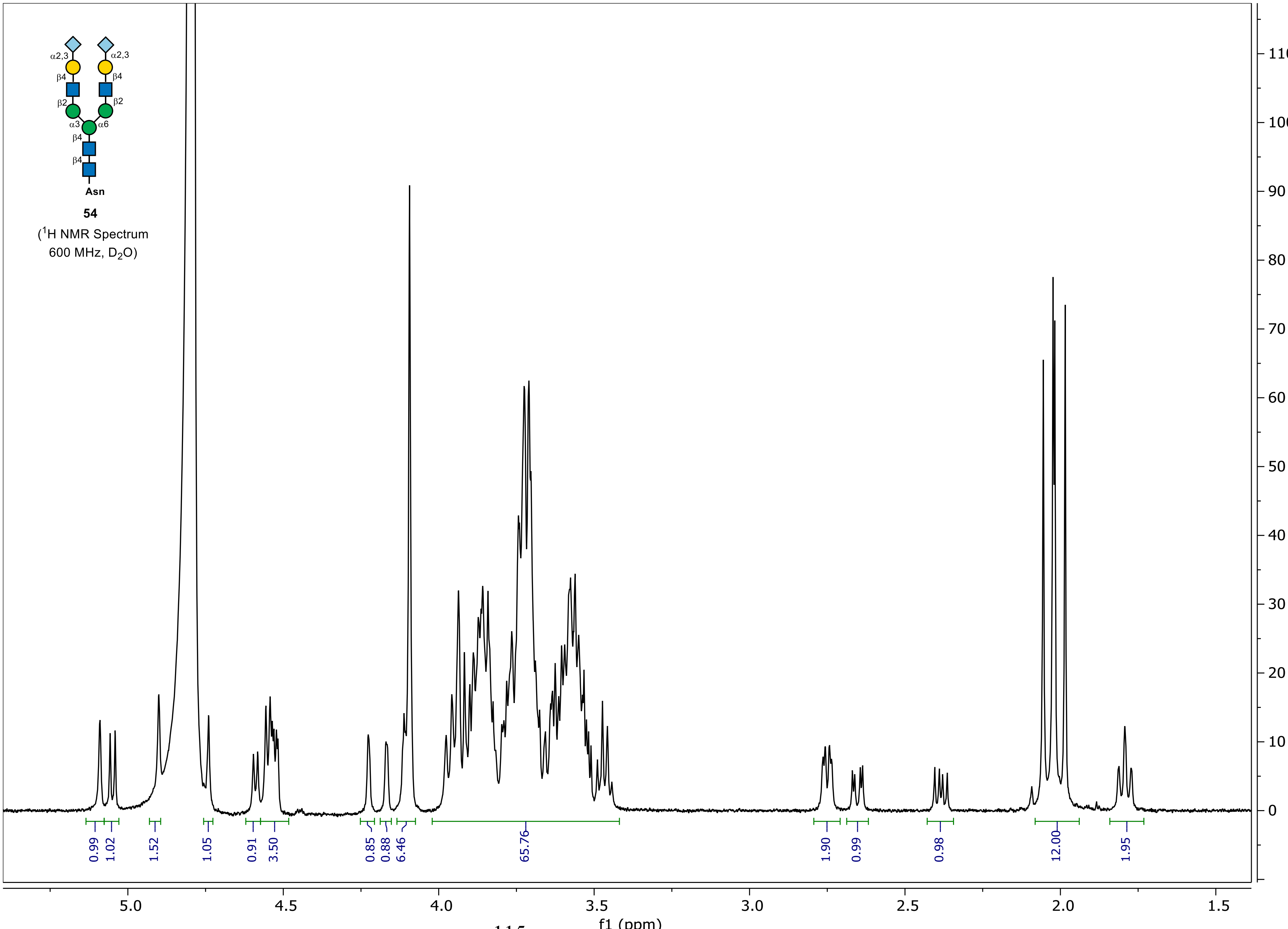
α2,3
α2,3
β4
β4
β2
β2
α3
α6
β4
β4
Asn
54
(1H NMR Spectrum
600 MHz, D2O)
0.99
1.02
1.52
1.05
0.91
3.50
0.85
0.88
6.46
65.76
1.90
0.99
0.98
12.00
1.95
5.0
4.5
4.0
3.5
3.0
2.5
2.0
1.5
f1 (ppm)
110
100
90
80
70
60
50
40
30
20
10
0

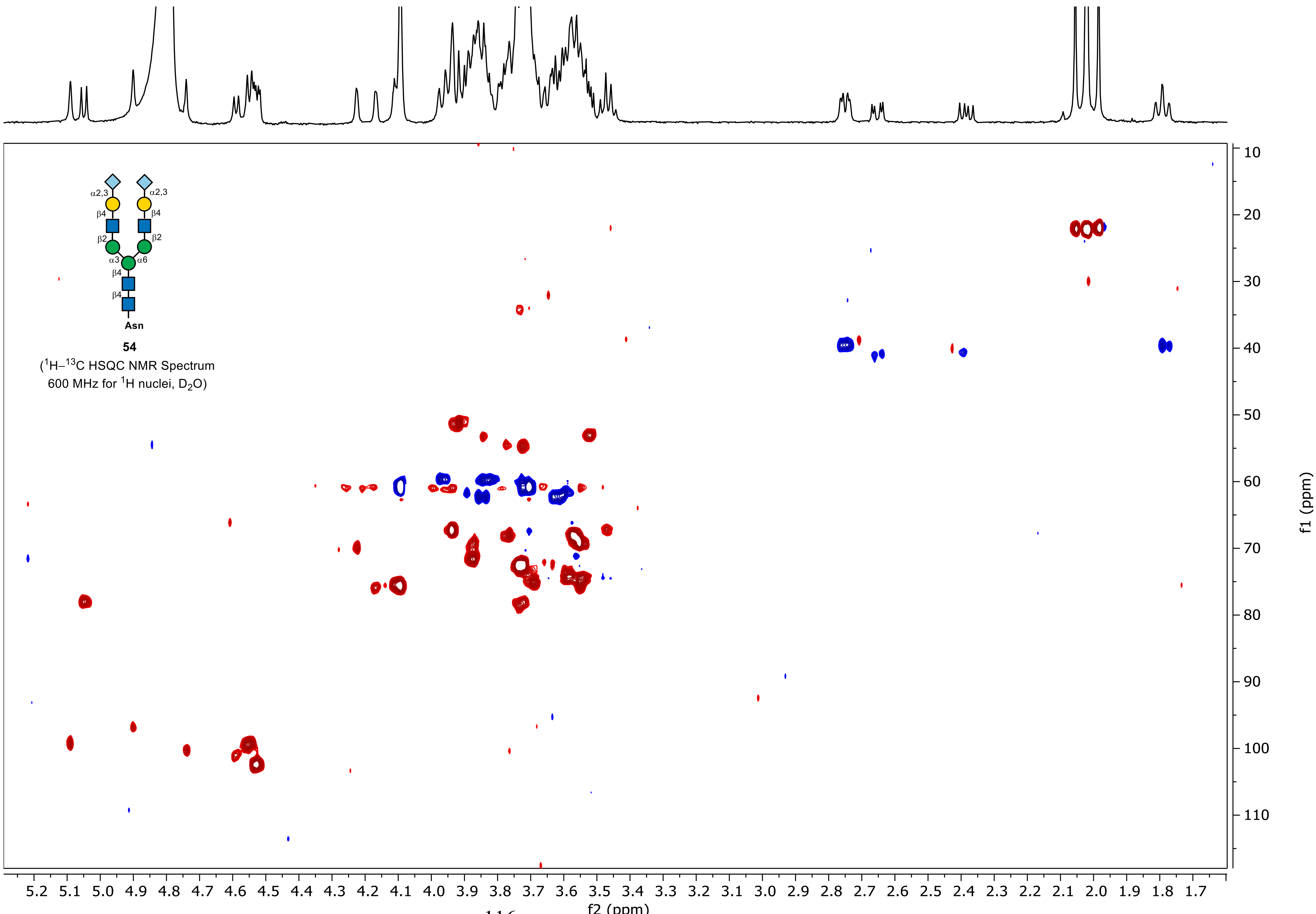
α2,3
α2,3
β4
β4
β2
β2
α3
α6
β4
β4
Asn
54
(^{1}H–^{13}C HSQC NMR Spectrum
600 MHz for ^{1}H nuclei, D_2O)
f1 (ppm)
f2 (ppm)

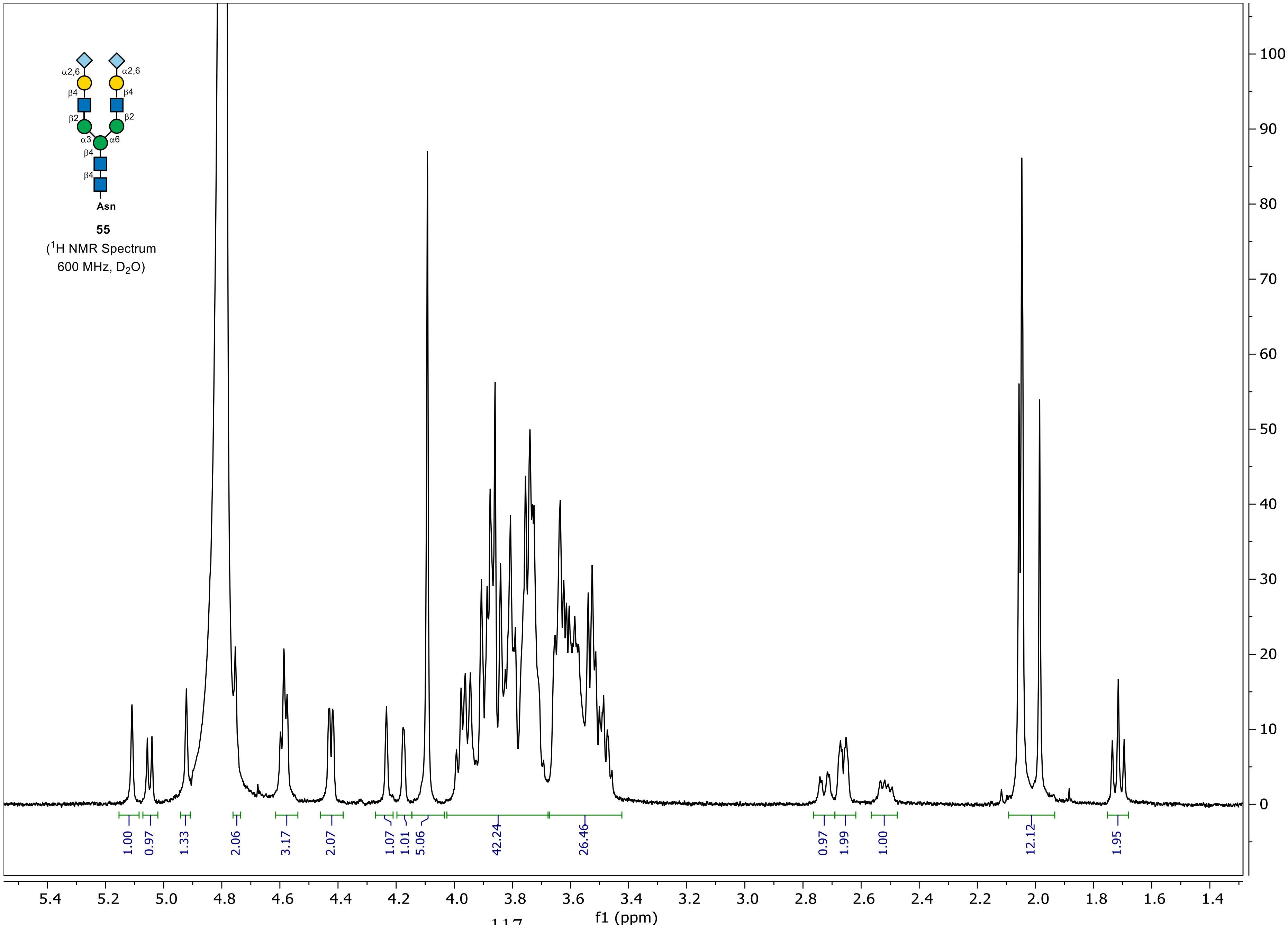
α2,6
α2,6
β4
β4
β2
β2
α3
α6
β4
β4
Asn
55
(¹H NMR Spectrum
600 MHz, D2O)
1.00
0.97
1.33
2.06
3.17
2.07
1.07
1.01
5.06
42.24
26.46
0.97
1.99
1.00
12.12
1.95
f1 (ppm)

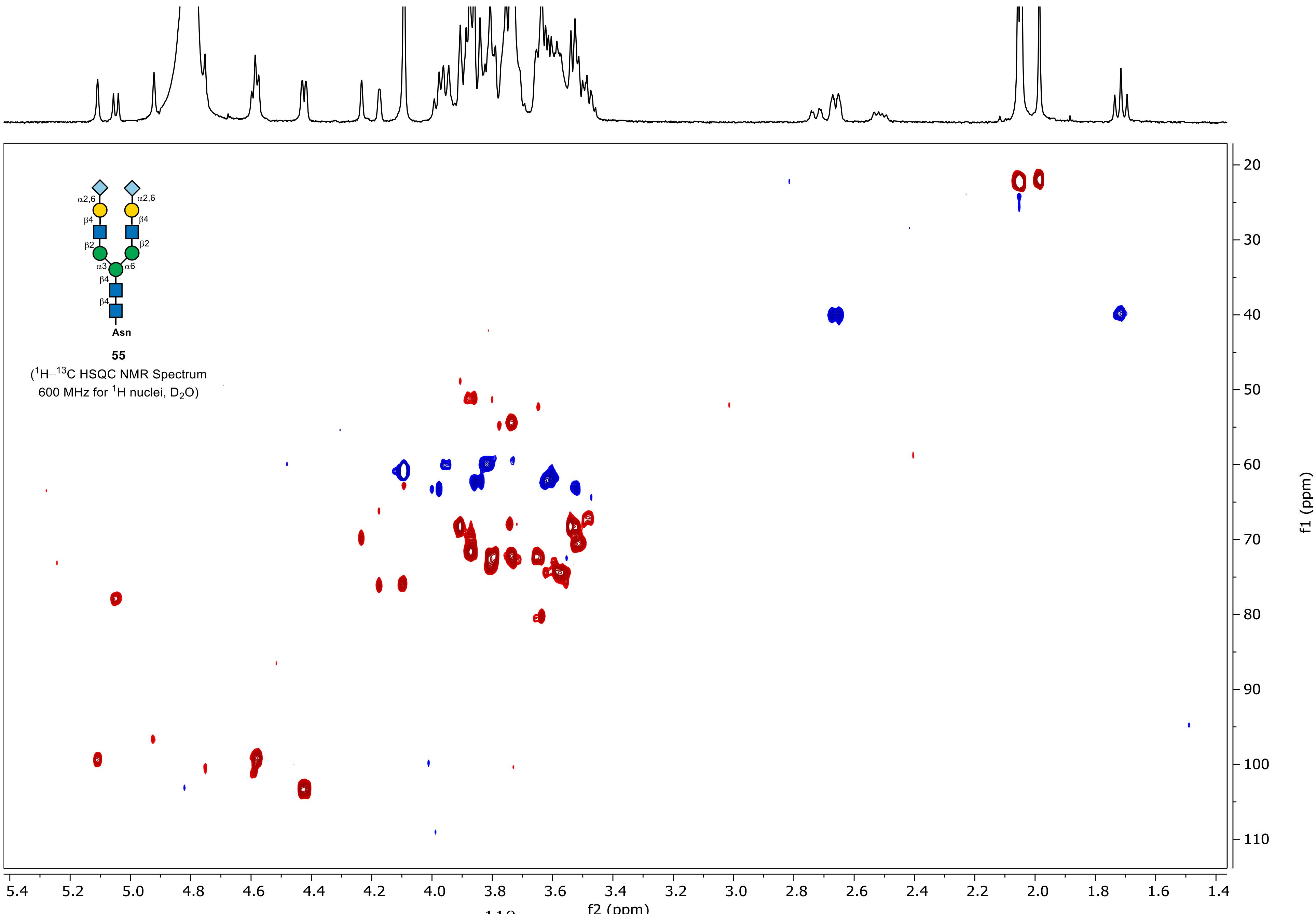

α2,6
α2,6
β4
β4
β2
β2
α3
α6
β4
β4
Asn
55
(^{1}H–^{13}C HSQC NMR Spectrum
600 MHz for ^{1}H nuclei, D$_2$O)
f2 (ppm)
f1 (ppm)

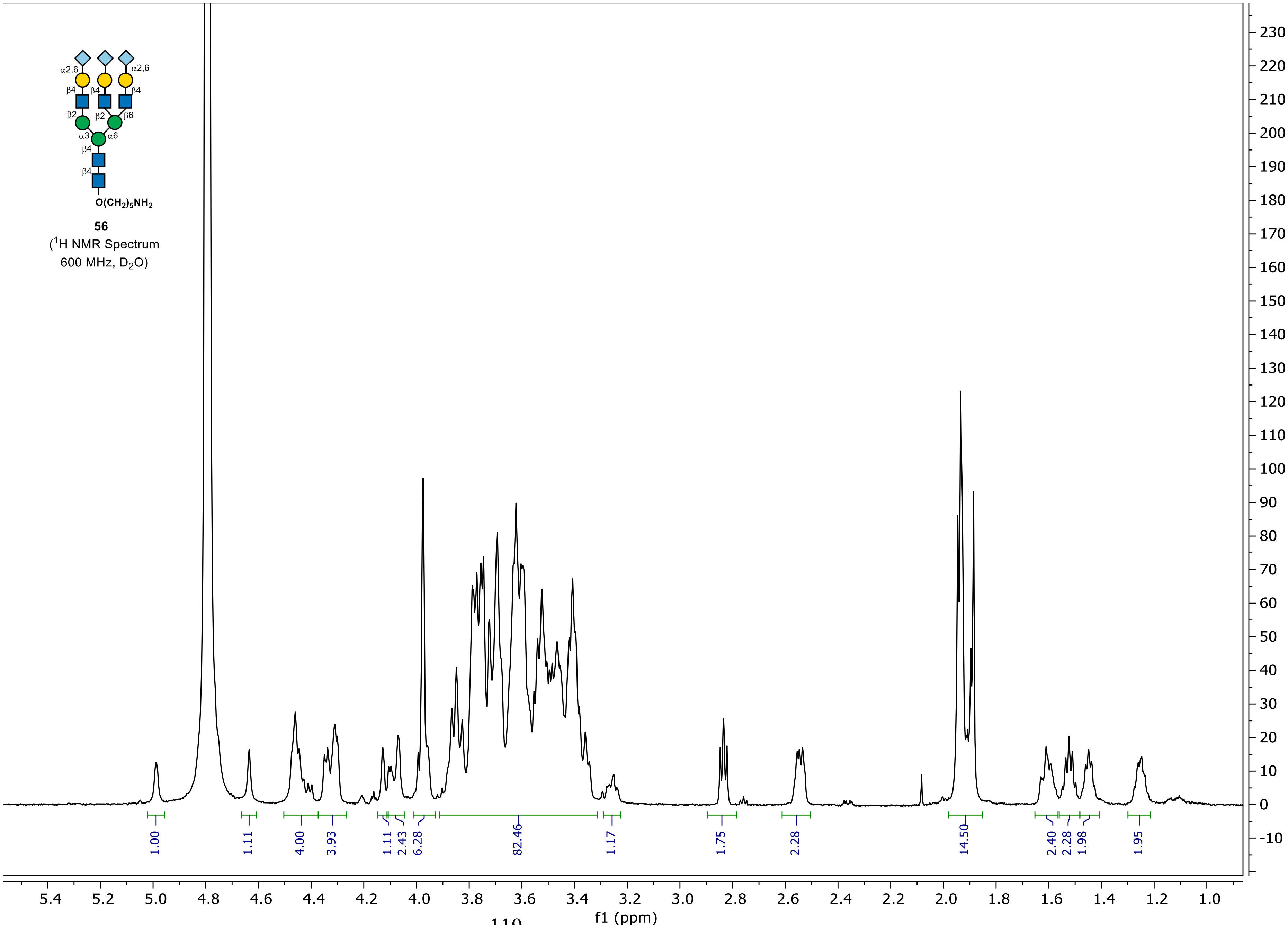
α2,6
α2,6
β4
β4
β4
β2
β2
β6
α3
α6
β4
β4
O(CH2)5NH2
56
(1H NMR Spectrum
600 MHz, D2O)
1.00
1.11
4.00
3.93
1.11
2.43
6.28
82.46
1.17
1.75
2.28
14.50
2.40
2.28
1.98
1.95
f1 (ppm)

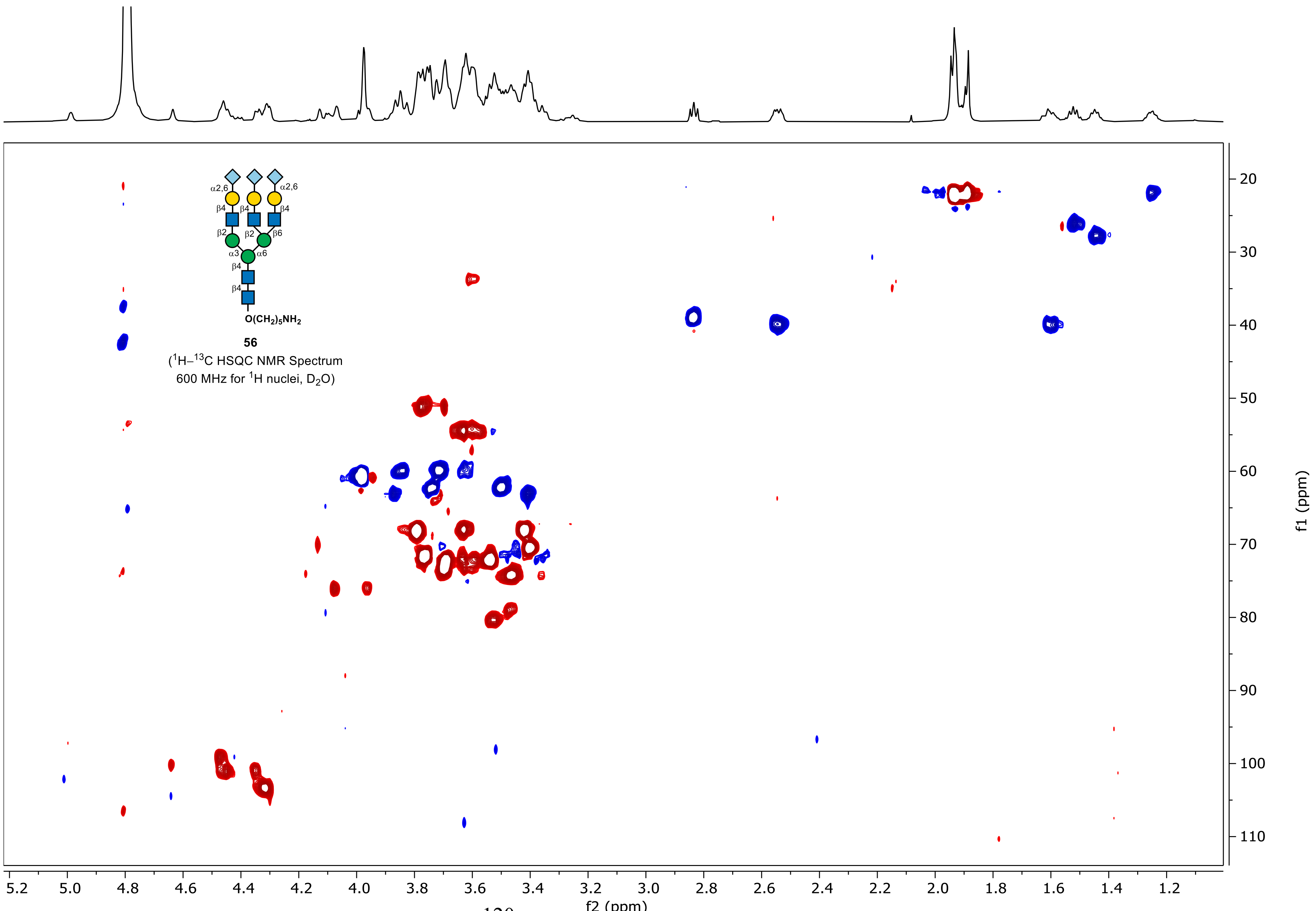

α2,6
β4
β2
β6
α3
α6
O(CH2)5NH2
56
(1H–13C HSQC NMR Spectrum
600 MHz for 1H nuclei, D2O)
f1 (ppm)
f2 (ppm)
